# Horizontal transfer of BovB and L1 retrotransposons in eukaryotes

**DOI:** 10.1186/s13059-018-1456-7

**Published:** 2018-07-09

**Authors:** Atma M. Ivancevic, R. Daniel Kortschak, Terry Bertozzi, David L. Adelson

**Affiliations:** 1 Department of Genetics and Evolution, Biological Sciences, The University of Adelaide, Adelaide, SA Australia; 2 Neurogenetics Research Program, Adelaide Medical School, The University of Adelaide, Adelaide, SA Australia; 3 Evolutionary Biology Unit, South Australian Museum, Adelaide, SA Australia

**Keywords:** Genome evolution, Horizontal transfer, Transposon, Eukaryote, Mammal

## Abstract

**Background:**

Transposable elements (TEs) are mobile DNA sequences, colloquially known as jumping genes because of their ability to replicate to new genomic locations. TEs can jump between organisms or species when given a vector of transfer, such as a tick or virus, in a process known as horizontal transfer. Here, we propose that LINE-1 (L1) and Bovine-B (BovB), the two most abundant TE families in mammals, were initially introduced as foreign DNA via ancient horizontal transfer events.

**Results:**

Using analyses of 759 plant, fungal and animal genomes, we identify multiple possible L1 horizontal transfer events in eukaryotic species, primarily involving Tx-like L1s in marine eukaryotes. We also extend the BovB paradigm by increasing the number of estimated transfer events compared to previous studies, finding new parasite vectors of transfer such as bed bug, leech and locust, and BovB occurrences in new lineages such as bat and frog. Given that these transposable elements have colonised more than half of the genome sequence in today’s mammals, our results support a role for horizontal transfer in causing long-term genomic change in new host organisms.

**Conclusions:**

We describe extensive horizontal transfer of BovB retrotransposons and provide the first evidence that L1 elements can also undergo horizontal transfer. With the advancement of genome sequencing technologies and bioinformatics tools, we anticipate our study to be a valuable resource for inferring horizontal transfer from large-scale genomic data.

**Electronic supplementary material:**

The online version of this article (10.1186/s13059-018-1456-7) contains supplementary material, which is available to authorized users.

## Background

Transposable elements (TEs) are mobile segments of DNA which occupy large portions of eukaryotic genomes, including more than half of the human genome [1]. Long interspersed element (LINE) retrotransposons are TEs which move from site to site using a ‘copy and paste’ mechanism, facilitating their amplification throughout the genome [2, 3]. The insertion of retrotransposons can interrupt existing genetic structures, resulting in gene disruptions, chromosomal breaks and rearrangements, and numerous diseases such as cancer [4–6]. Two of the most abundant retrotransposon families in eukaryotes are LINE-1 (L1) and Bovine-B (BovB) [7, 8].

Horizontal transfer (HT) is the transmission of genetic material by means other than parent-to-offspring. Given a vector of transfer (e.g. virus, parasite), retrotransposons have the innate ability to jump between species as they do within genomes [2, 9–11]. Studies investigating the possibility of retrotransposon HT are limited, mainly including CR1s and RTEs [8, 12–15]. Using over 700 publicly available genomes from plants, fungi and animals, we tested the hypothesis that HT is a ubiquitous process not restricted to certain species or retrotransposons. We used L1 and BovB elements as exemplars because of their contrasting dynamics and predominance in mammalian genomes. BovB retrotransposons provide an excellent example of HT: divergent species contain highly similar BovB sequences and the analysis of various insect species has revealed plausible vectors of transfer [8, 11]. In contrast, L1 elements are believed to be only vertically inherited [16]. We hypothesise that the very presence of L1s in today’s mammals is due to an ancient HT event. In this study, we use BovBs as a comparison to identify common characteristics of horizontally transferred elements in contemporary eukaryotic species.

Three criteria are typically used to detect HT candidates: (1) a patchy distribution of the TE across the tree of life; (2) unusually high TE sequence similarity between divergent taxa; and (3) phylogenetic inconsistencies between TE tree topology and species relationships [17]. To comprehensively test these criteria, we performed large-scale phylogenomic analyses over 700 eukaryotic genomes (plants, fungi and animals) using iterative protein and nucleotide similarity searches of BovB and L1 sequences.

## Results

### Distribution and abundance of TEs across species

**Fig. 1:**
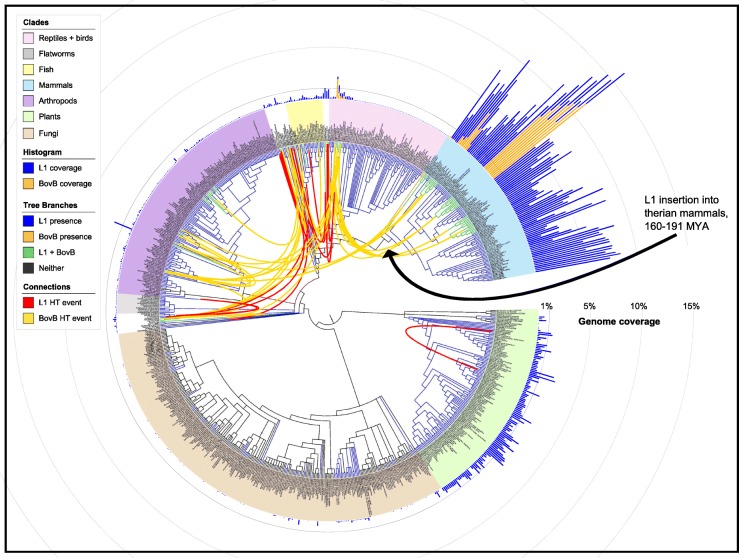
Presence and coverage of L1 and BovB elements across eukaryotes. The Tree of Life [59] was used to infer a tree of the 759 species used in this study; iTOL [58] was used to generate the *bar graph* and final graphic. The *black arrow* marks the proposed L1 HT event into therian mammals 160–191 MYA. *Branches* are coloured to indicate which species have both BovB and L1 (*green*), only BovB (*orange*), only L1 (*blue*) or neither (*black*). *Bar graph colours* correspond to BovB (*orange*) and L1 (*blue*). Connections indicate possible HT events involving BovB (*yellow*) or L1 (*red*) elements. An interactive and downloadable version of this figure is available at: http://itol.embl.de/shared/atma

Our findings show that there are two phases in HT: effective insertion of the TE, followed by expansion throughout the genome. Figure 1 shows that both BovB and L1 elements are present in a diverse array of species including mammals, reptiles, fish, amphibians, arthropods and primitive species such as sea urchins and sea squirts. Both retrotransposons also have a patchy distribution across our sampled eukaryotes. The main difference between BovB and L1 lies in the number of colonised species. BovBs are only present in 72 of the 759 species analysed, strictly within animals, so it is easy to trace their HT between the distinct clades (e.g. squamates, ruminants). In contrast, L1s encompass a total of 559 species, including plants, animals and several fungal species, and they are ubiquitous across the well-studied therian mammals. The only species which appear to have BovBs yet lack L1s are the two monotremes, platypus and echidna.

The abundance of TEs differs greatly between species. As shown in Fig. 1, mammalian genomes are incredibly susceptible to BovB and L1 expansion. More than 17% of the cow genome comprises these TEs (12% BovB, 5% L1; see Additional file 1: Table S4). This is without considering the contribution of TE fragments [18] or derived short interspersed elements (SINEs), boosting retrotransposon coverage to > 50% in some mammals [1]. Even within mammals, there are noticeable differences in copy number; for example, bats and equids have a very low number of full-length BovBs (< 50 per genome) compared to the thousands found in ruminants and Afrotherian mammals. The low copy number here is TE-specific rather than species-specific; there are many L1s in bats and equids. Hence, the rate of TE propagation is determined both by the genome environment (e.g. mammal versus non-mammal) and the type of retrotransposon (e.g. BovB vs L1).

### Widespread HT of BovB in animals

**Fig. 2:**
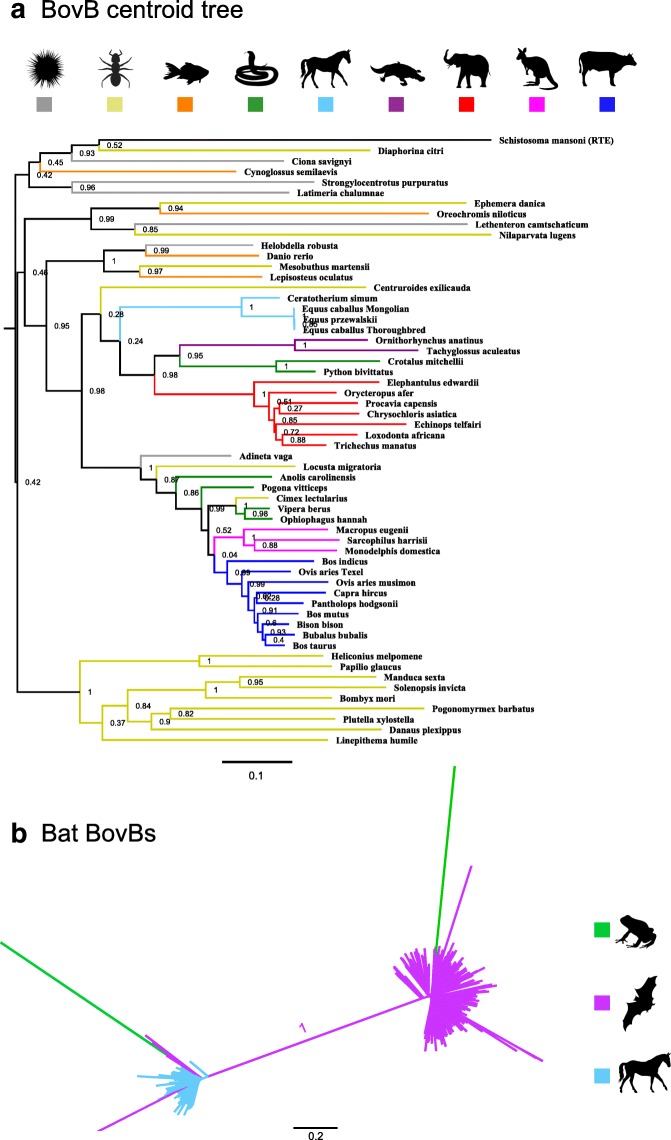
HT of BovB retrotransposons. **a** Representative BovB tree inferred using nucleotide centroid sequences, where the centroid was the longest intact BovB sequence for each species (min cut-off length 2 kb, max cut-off length 4 kb for species with chimeric BovBs). MUSCLE [50] was used to align sequences, Gblocks [51] was used to extract conserved blocks, FastTree [52] was used to infer a maximum likelihood phylogeny. FastTree branch support values are shown and branches are coloured taxonomically. RTE sequence from *Schistosoma mansoni* was included as an expected outgroup. **b** BovB subgroups in bats and frogs. All bat, frog, horse and rhino BovB nucleotide sequences in the range of 2–4 kb were grouped into a file. As above, MUSCLE [50] was used to align sequences, Gblocks [51] was used to extract conserved blocks, FastTree [52] was used to infer a maximum likelihood phylogeny and branches are coloured taxonomically. The *Xenopus laevis* subgroup (*left*) contains sequences from Perissodactyla (horses and rhino) and Chiroptera (bats). The *Xenopus tropicalis* subgroup (*right*) forms a distinct clade, clustering with the majority of the bat BovB sequences

To develop a method for identifying HT events, we used BovB, a TE known to undergo HT. First, we generated a representative BovB phylogeny using consensus and centroid approaches (see ‘Methods’ for details). Figure 2a shows the centroid BovB tree, where the centroid for each species was the longest intact BovB sequence. The phylogeny supports previous results [8]—with the topology noticeably different from the tree of life (Fig. 1)— although we were able to refine our estimates for the times of insertion. For example, the cluster of equids includes the white rhinoceros, *Ceratotherium simum*, suggesting that BovBs were introduced into the most recent common ancestor before these species diverged. The low copy number in equids and rhinoceros, observed in Fig. 1, is not because of a recent insertion event; the most likely explanation is that the donor BovB inserted into an ancestral genome, was briefly active, but lost its ability to retrotranspose and was subsequently vertically transmitted.

The placement of arthropods in the BovB tree is intriguing, revealing potential HT vectors and the origin of BovB retrotransposons. For example, the RTE-like BovBs from butterflies, moths and ants appear as sister groups to the main BovB clade. This suggests that BovB TEs may have arisen as a subclass of ancient RTEs, countering the belief that they originated in squamates [14]. Within the central clade, we see a scattering of possible vector species including a leech (*Helobdella robusta*), two scorpion species (*Mesobuthus martensii* and *Centruroides exilicauda*) and a locust (*Locusta migratoria*). But the most interesting arthropod species is *Cimex lectularius*, the common bed bug, known to feed on animal blood. The full-length BovB sequence from *Cimex* shares > 80% identity to viper and cobra BovBs; their reverse transcriptase domains share > 90% identity at the amino acid level. Together, the bed bug and leech support the idea [8, 19] that blood-sucking parasites can transfer retrotransposons between the animals they feed on.

Our mining of BovB sequences further revealed two concurrent BovB subgroups in bats and frogs. Two frog species (*Xenopus laevis* and *Xenopus tropicalis*) each contain a single intact BovB sequence > 2 kb in length (and numerous fragments), but these two sequences are very different and correlate with the two distinct BovB subgroups observed in bats (Fig. 2b). This seems to indicate at least two independent insertion events, somehow connecting *Xenopus laevis* with the ‘horse-like’ BovB group, and *Xenopus tropicalis* with the bat-specific BovB group (most similar to the BovBa-1_EF consensus from RepBase [20]). Without intermediary species, it is difficult to infer the chain of events that led to these patterns.

Finally, to exhaustively search for all cases of BovB HT, we tested several all-against-all clustering approaches to detect individual HT candidate sequences. We first replicated the method described in El Baidouri et al. [21], which uses BLAST [22] to compare all sequences within a database, and SiLiX [23] to extract discordant clusters. This worked well for recent BovB transfers (e.g. *Cimex lectularius*—snakes) but failed to identify ancient transfer events and required considerable computational time and power. Next, we tested VSEARCH [24], which is orders of magnitude faster than BLAST [22]. A total of 174,510 BovB sequences were clustered in < 15 min on a high-performance computing cluster with 16 cores. We clustered full-length nucleotide sequences, nucleotide sequences from just the open reading frames (ORFs) and amino acid sequences from extracted reverse-transcriptase (RT) domains (see ‘Methods’).

Many of the resulting clusters contained BovBs from closely related species, e.g. cow and yak. To find the most compelling HT events, we imposed the restriction that clusters had to contain BovBs from species that belonged to different eukaryotic Classes (e.g. Mammalia and Insecta). We performed *a machina* validation for each candidate HT cluster: pairwise alignments of the flanking regions to rule out possible contamination or orthologous regions; phylogenetic reconstructions to confirm discordant relationships; and reciprocal best hit checks to confirm correct clustering (see ‘Methods’). Combining both nucleotide and amino acid results, a total of 67 HT clusters were detected (visualised as connections in Fig. 1, described in detail in Additional file 1: Table S5). This includes recent transfers between ruminants and reptiles, often grouped with bed bug or locust BovBs (as shown in Fig. 2a), and older transfers between scorpions and fish, mayfly and a *Myotis* bat, rotifer and leech. The *Pogona vitticeps* lizard appears in numerous different animal groupings, suggesting a high level of active retrotransposition (Additional file 2: Figure S38) and subsequent HT.

Altogether, our results demonstrate that the HT of BovB elements is even more widespread than previously reported, providing one of the most compelling examples of non-LTR HT across animals.

### Possible L1 HT in aquatic species and plants

We carried out the same exhaustive search in L1s, which presented a challenge because of greater divergence and the sheer number of vertically inherited copies. Producing a consensus for each species was impractical as most species contained a mixture of old degraded L1s and young intact L1s. Instead, we used the all-against-all clustering methods on the collated dataset of L1 nucleotide sequences > 3 kb in length (> 1 million sequences total). Once again, VSEARCH [24] was substantially faster and identified more potential HT candidates than the BLAST+SiLiX method [21–23]. This is likely due to a crucial difference in clustering algorithms; SiLiX uses single linkage to draw connections between sequences, which is effective for recent HT events but clusters all ‘degraded’ elements into a single group. In contrast, VSEARCH relies on centroid/average linkage, and is thus more appropriate for ancient HT events (where the centroid is ideally the transferred TE).

**Fig. 3:**
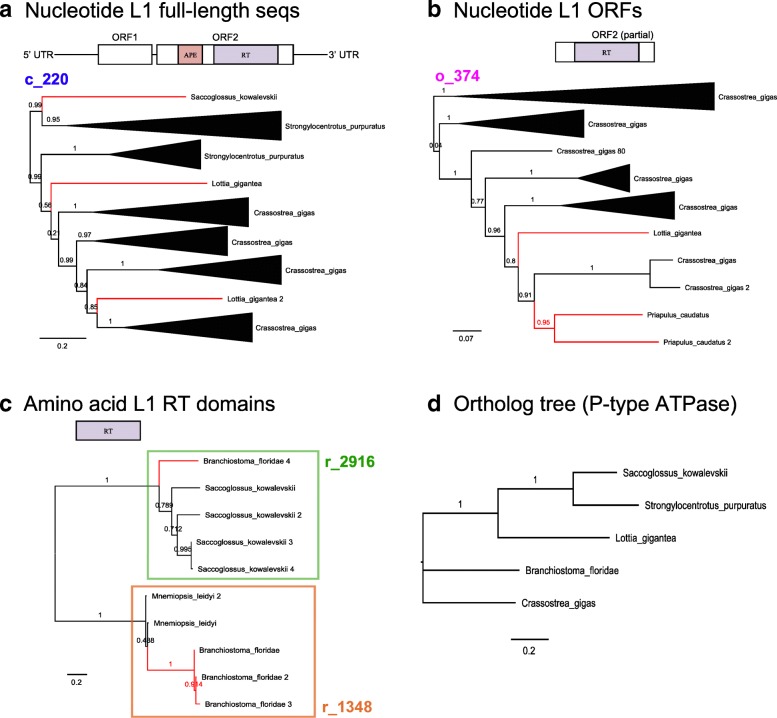
L1: TE trees of cross-Phylum HT clusters. **a** Full-length nucleotide L1s. Phylogeny of a putative cross-Phylum L1 HT event involving ancient Tx-like L1 elements in aquatic species. MUSCLE [50] was used to align the sequences, Gblocks [51] was used to extract conserved blocks from the alignment, FastTree [52] was used to infer a phylogeny and FigTree was used for tree rendering. **b** Nucleotide ORFs. Same as (**a**), but this family was found from the all-against-all clustering of ORF sequences rather than full-length L1s. **c** Amino acid RT domains. Same as (**a**, **b**), but these families were found from the all-against-all clustering of amino acid RT domains. **d** Species tree inferred from gene orthologs (specifically, P-type ATPase). Sequences were obtained from OrthoDB [60], aligned with MUSCLE and the maximum likelihood phylogeny was inferred using FastTree. Note that there was no information available for *Priapulus* or *Mnemiopsis*

**Fig. 4:**
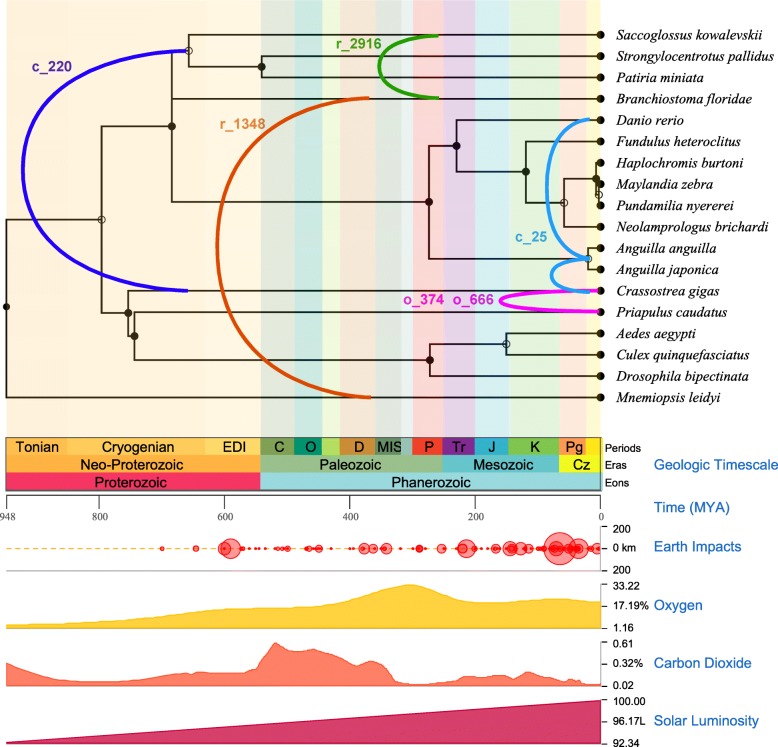
L1: expected species trees. TimeTree [25] using published data from thousands of studies to infer the relationships of the species involved in all six putative L1 cross-Phylum HT events. Background is coloured to match the ages in the geological timescale. Note that there was no information available for *Lottia gigantea*

Over 9000 clusters contained L1s from at least two different species: these were our HT candidates. The vast majority of these clusters contained L1s from closely related species. As before, to improve recognition of HT vs vertical inheritance, we looked for families displaying cross-Class or cross-Phylum transfer. We clustered full-length nucleotide sequences (Fig. 3a), nucleotide ORFs (Fig. 3b) and amino acid RT domains (Fig. 3c). We checked for discordance compared to orthologs (Fig. 3d), absence in neighbouring species and elevated sequence identity compared to flanking regions. To confirm the ortholog trees (particularly for species with no known ortholog data), we used TimeTree [25] to estimate species divergence times and infer species relationships from previous studies and fossil records (Fig. 4). By using the procedure we had established for BovB elements (see ‘Methods’), we were able to retain 18 L1 clusters as potential HT events that span different eukaryotic Classes or Phyla (Additional file 1: Table S6). Additional clusters which looked promising but could not be confirmed due to short scaffolds in the draft assembly or lack of functional domains in the ORFs are also listed in Additional file 1: Table S6 (marked as likely contamination or likely artefacts, respectively).

All of the cross-Phylum clusters involve marine eukaryotes, with potential vector species such as the Pacific oyster (*Crassostrea gigas*), the catus worm (*Priapulus catus*) and a sea worm usually found in coastal mud or sand (*Saccoglossus kowalevskii*) (Fig. 4). Notably, all of the cross-Phylum clusters contain the diverse Tx-like L1s originally discovered in *Xenopus* frogs [26, 27]. Likewise with the cross-Class clusters, with the exception of one plant cluster based on RT domains (r_1111 in Additional file 1: Table S6). In contrast to BovBs, there is no strong evidence to suggest ongoing L1 HT in mammals. Relaxing our clustering criteria (e.g. to include cross-Order or cross-species HT candidates) resulted in sporadic groupings of different mammals – most likely clustered together because they all contained ‘dead’, inactive L1s.

**Fig. 5:**
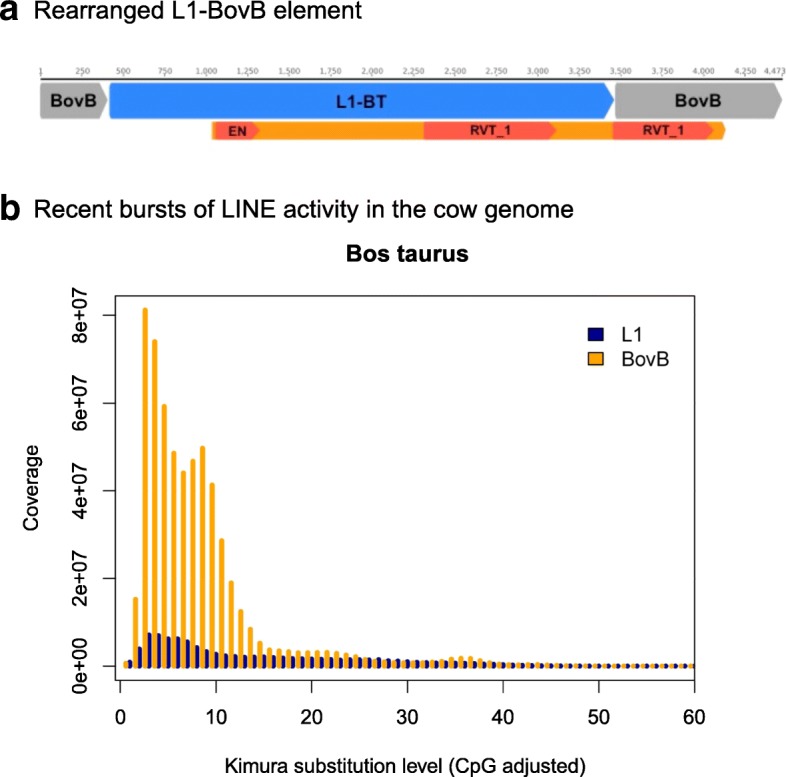
Chimeric L1-BovB element in cattle genomes. **a** Chimeric L1-BovB retrotransposon found in cattle genomes (*Bos taurus* and *Bos indicus*). L1-BT and BovB correspond to RepBase names [20], representing repeats which are known to have been recently active. The *orange bar* is the length of the entire ORF. **b** Kimura divergence plot of BovB and L1 elements in *Bos taurus*. RepeatMasker [54] was used to mask all BovB and L1 nucleotide sequences in the cow genome and calculate divergence from alignments to RepBase [20] consensus sequences. The *y-axis* represents coverage against the RepBase super consensus library; the *x-axis* indicates the Kimura divergence estimate. Ruminants are the only mammals which have currently active lineages of both BovB and L1 elements, explaining how the chimeric BovB-L1 element may have arisen. RVT_1 reverse transcriptase, EN endonuclease domain

Finally, our mining of L1 HT candidates led to the serendipitous discovery of a chimeric L1-BovB element present in cattle genomes (*Bos taurus* and *Bos indicus*), shown in Fig. 5a. This rearranged copy likely arose from a recently active L1 element (98% identical to the canonical *Bos* L1-BT [20]) inserting into an active BovB (97% identical to *Bos* BovB [20]). Ruminants are the only mammals that currently have active lineages of both BovB and L1 elements (Fig. 5b), creating the ideal genomic environment for the genesis of chimeric repetitive elements. The hybrid element contains two RT domains and high similarity to active L1/BovB elements, although there is little evidence to suggest transcription (Additional file 2: Figure S55). Nonetheless, it raises an important question: can L1 elements to be transferred throughout mammals by being transduced in other, more prolific TEs, such as BovBs?

## Discussion

### The curious case of L1 absence in monotremes

Figure 1 shows the similarly patchy distributions of BovB and L1 elements across our inferred tree of life. Monotremes are a particularly interesting discrepancy because they contain BovBs, yet lack L1s. There are several possible explanations for this: either L1s could not be detected due to the draft status of the genomic data; or L1s were expunged shortly after the monotreme-therian split, before they had a chance to accumulate; or monotremes never had L1s. To control for genome quality, we used two independent searching strategies to mine for L1s in both full genome data (Illumina and PacBio platypus assemblies) and all available nucleotide databases, as well as a third method to act as a reciprocal best-hit check (see ‘Methods’). Species were annotated ‘L1-present’ if there was any evidence of fragments or full-length copies from at least one of the methods. There was no hit at all for echidna; the few isolated fragments found in the platypus assembly were known contaminants from wallaby [27] or more likely to be ancestral L2/CR1 fragments. We could easily identify other TE families in both species, including an abundance of L2s and BovBs.

The second scenario is also unlikely in the context of L1 distributions in other eukaryotes. TE removal from a genome is thought to occur through a series of mid-size to large segmental deletions (31 bp to 10 kb) [28]. However, this process is not absolute; it is difficult to remove all evidence of a TE family, especially since the extinct and degraded copies are unlikely to carry a selective disadvantage. Consider the 60 analysed bird genomes: full-length L1s have been eradicated from the avian lineage, yet every bird species bears evidence of ancient/ancestral L1 activity through the presence of fragments that contain recognisable RT domains. Similarly, L1s have been functionally inactive in megabats for at least 24 million years [29], yet their genomic history is preserved via degraded L1 remains. This is not the case for platypus or echidna. We therefore conclude that L1s were probably never present in the monotreme lineage. To emphasise this, the L1 explosion in therian mammals mimics the rapid BovB expansions in ruminants and Afrotherian mammals (Fig. 1). Our results indicate that L1s were inserted into a common ancestor of therian mammals 160–191 million years ago (MYA) and have since been vertically inherited.

### Both BovB and (Tx-like) L1 satisfy the criteria for HT

The typical criteria used to infer HT are a patchy distribution across taxa, phylogenetic inconsistencies in the TE topology and high TE sequence similarity between divergent species. As discussed above, both BovB and L1 have a patchy distribution across the eukaryotic tree of life – for L1, this is best seen by including fungi and diverse plant species. Both BovB and L1 also show phylogenetic inconsistencies in TE topology. For BovB, this is evident immediately from the initial tree (Fig. 2a); for L1, this is shown in the individual phylogenies constructed from the HT clusters (Fig. 3; Additional file 2: Figures S1 and S2). In each case, the species involved in the HT event appear too closely related on the TE tree and neighbouring species lack evidence of similar copies.

In terms of high sequence similarity, the level of identity between transferred elements seems largely dependent on how recently the HT event occurred. For example, consider the BovB HT events. The BovB element from bed bug *Cimex lectularis* shares > 80% similarity to BovBs from three snake species (Fig. 2a), suggestive of a recent event. Ancient HT events are unlikely to have such a high degree of similarity, due to accumulated mutations over time. In BovB, the ancient HT events were found by reducing the clustering identity to 50–60% and using a centroid-based clustering strategy [24] rather than single linkage [23].

The L1 HT families satisfy these same criteria. Using stringent identity parameters of ≥ 80%, we could not find any promising candidates; there have been no recent L1 HT events in our subset of species. However, the cross-Phylum L1 transfers between aquatic species mimic the ancient BovB events, with sequence similarity restricted to the TE sequence or RT domain. This contradicts the belief that L1s are exclusively vertically inherited and supports our hypothesis that a similar event introduced L1s to therian mammals.

### Transfer frequency and mechanisms differ between TE classes

The main argument against L1 HT is the frequency of transfer vs number of colonised species. For example, consider the number of cross-Phylum HT events found for each TE. We detected more cross-Phylum transfers involving BovB elements (especially between widely divergent groups such as reptiles and mammals), yet they are only present in 72 of the 759 analysed species. In contrast, we were only able to find evidence for six cross-Phylum L1 HTs (all among sea-dwelling creatures). These six events cannot explain how L1s arose in therian mammals or came to colonise > 500 species. If L1 HT is so rare, how have L1s come to dominate almost all of the major clades of plants and animals, and even appear in fungi?

There are several explanations for these observations. First, L1s are ancient: they have been around for millions of years longer than BovBs, which only emerged recently (possibly as a subclass of ancient RTEs). BovB HT is easy to trace because we can see the likely insertion point for each distinct group of species (Fig. 1). In contrast, L1 HT events potentially occurred before the origin of today’s species. If L1s inserted into an early ancestor of therian mammals, it is also possible they inserted into the ancestor of sauropsids, or fish. The patterns we observe in Fig. 1 could then be explained by subsequent vertical inheritance into descendent species.

Another aspect to consider is the mechanism of transfer. BovB and L1 are similar in structure, but L1 sequences are almost twice as long as BovB. This may reduce the likelihood of successful transfer and integration into the new host genome. Moreover, the cross-Phylum clusters implicate aquatic metazoans such as oysters, molluscs and marine worms as possible vectors of L1 HT. Compared to arthropods, these types of species are heavily underrepresented in our dataset; we are missing numerous potential intermediary species. Further studies should explore different vectors of transfer (microbiological or viral) to provide a more comprehensive representation of the tree of life.

Finally, our analysis only considered TE candidates from cross-Class species, to find the most extreme cases of HT. Several studies have suggested that HT is more likely to occur between closely related species with similar genomic environments [11, 12, 30]. The BovB results (e.g. Fig. 2a, Additional file 1: Table S5) suggest that there has been ongoing HT even between ruminant species. Accurately identifying HT events between similar species or individuals of the same species would give a better approximation for TE transfer frequency, although this is complicated by the noise of vertically inherited and degraded TE copies.

### Suppression of L1 and BovB activity in megabat genomes

Both the BovB and L1 results suggest that transferred TEs can retain activity and expand within their new host. However, the extent to which a TE can propagate in a new organism depends on factors such as a favourable genomic environment and TE replication machinery. Mammals appear to be more susceptible to TE expansion than other species (Fig. 1). However, bats seem exceptional in their ability to quickly suppress LINE activity.

Bats, particularly megabats, are often used as an example of L1 extinction affecting an entire lineage [27, 29]. Bat BovB sequences are similarly degraded. Despite the presence of two BovB subfamilies, indicative of two independent HT events, bat BovBs show little evidence of replication and no intact functional domains. In fact, the megabat group is the only monophyletic clade on our tree showing L1/BovB presence coupled with complete extinction of both TE families. This is important in the context of host suppression mechanisms.

Bats are frequently implicated as vectors of DNA exchange: they transmit numerous viruses and TEs, cause disease epidemics and feed on arthropods [31–33]. As such, they are the ideal intermediate species for HT. Constant exposure to potentially harmful DNA may have led to the evolution of heightened TE silencing mechanisms. This is supported by the observation that bats have a relatively compact genome size and experience dynamic loss and gain of DNA [28]. It is likely that bats act as TE reservoirs [34]: enabling the transmission of foreign DNA while minimising impact to their own genomes.

### HT of L1s potentially influenced the evolution of therian mammals

Over 30 years ago, Barbara McClintock pioneered the discovery of TEs, flagging them as ‘controlling elements’ of the genome [35]. In the last few years, we have finally started seeing evidence of their functional importance. A study of 29 mammals found > 280,000 non-coding elements exapted from TE insertions [36]. TEs have been implicated in the evolution of innate immunity [37, 38] and the placenta [39, 40], as well as transcriptional regulation of mammalian brains [41]. The structural changes arising from horizontally transferred TEs have contributed to the modification of regulatory elements and led to the development of novel traits (recently reviewed by Boto [42]). Recent evidence also shows that Krüppel-associated box domain-containing zinc-finger proteins (KRAB-ZFPs) use TEs, particularly endogenous retroviruses and L1s, to establish species-specific networks of epigenetic regulation [43, 44]. We speculate that the transfer (and consequent expansion) of L1s into therian mammals helped facilitate regulatory network modification, potentially contributing to the rapid speciation that occurred following the split from monotremes.

## Conclusions

Our analyses indicate that both BovB and L1 retrotransposons, particularly Tx-like L1s, have undergone HT events in the past. We extracted millions of retrotransposon sequences from a 759-genome dataset, demonstrating the similarly patchy distributions of these two LINE classes across the eukaryotic tree of life. We further extended the analysis of BovBs to include blood-sucking and migratory arthropods capable of parasitising mammals and squamates, as well as two distinct clades of bat/frog BovBs. Contrary to the belief of exclusive vertical inheritance, our results with L1s suggest multiple ancient HT events in eukaryotes, mainly among aquatic species, and possible HT into the early therian mammal lineage. The rapid speciation following the split of theria and australosphenids (monotremes), 160–191 MYA, coincides with the invasion of L1 elements into therian genomes. We therefore speculate that the speciation of therian mammals was driven in part by the effect of L1 retrotransposition on genome structure and function, including regulatory effects on transcriptional networks. This ancient transfer event allowed expansion of L1s and associated SINEs, transformation of genome structure and regulation in mammals [45], and potentially catalysed the therian radiation.

## Methods

Source code and workflow guide is available on Zenodo [46] and GitHub:

https://github.com/AdelaideBioinfo/horizontalTransfer

The interactive and downloadable tree of life is available at:

http://itol.embl.de/shared/atma

### Extraction of L1 and BovB retrotransposons from genome data

To extract the retrotransposons of interest, we used the methods and genomes previously described in Ivancevic et al. [27], as well as 256 new genomes (Additional file 1: Table S1). Briefly, this involved downloading 755 publicly available genomes (and acquiring four more from collaborations), then using two independent searching strategies (LASTZ [47] and TBLASTN [22]) to identify and characterise L1 and BovB elements. A third program, CENSOR [48], was used with the RepBase library of known repeats [20] to verify hits with a reciprocal best-hit check. The inclusion of fungal L1 queries facilitated the discovery of diverse and ancient L1 elements in metazoans, particularly in animals and insects. Both L1 and BovB results are summarised in the Supplementary Material (Additional file 1: Tables S2 and S3, respectively).

### Inferring a representative BovB tree from consensus/centroid sequences

The canonical BovB retrotransposon is 3.2 kb in length [8, 20], although this varies between species. We wanted to construct a BovB representative for each species. To this end, we tested consensus and centroid approaches to generate one BovB ‘representative’ sequence per species.

First, we tried a consensus sequence approach. For each species, UCLUST [49] was used to cluster full-length BovB sequences at varying identities in the range of 70–90%. A consensus sequence of each cluster was generated using the UCLUST -consout option. This worked well for most species but generated a very long consensus for species with degraded or divergent BovBs (e.g. bats).

Second, we tried a centroid sequence approach, where the ‘centroid’ for each species was the longest intact BovB sequence. We set 2 kb as the minimum length cut-off for intact elements. For species with long stretches of overlapping BovBs (e.g. cow, which has BovB genomic regions > 8 kb), we introduced a 4-kb maximum cut-off length. BovB representative sequences for each species were then aligned using MUSCLE [50] and the multiple alignment was processed with Gblocks [51] to extract conserved blocks, with default parameters except min block size: 5, allowed gaps: all. FastTree [52] was used to infer a maximum likelihood phylogeny using a general time reversible (GTR) model and gamma approximation on substitution rates. FastTree support values are shown on the tree branches in Fig. 2a.

### Distinguishing between RTE and BovB elements

All sequences identified as BovB or RTE were kept and labelled according to their closest RepBase classification [20]. However, there appear to be numerous discrepancies with the naming: e.g. some RTE sequences shared > 90% identity to BovBs and vice versa. BovB retrotransposons were discovered relatively recently; it is likely that several RepBase sequences labelled ‘RTE’ are actually BovBs.

To determine which sequences were BovBs and which were RTEs, we clustered all the sequences in each genome using UCLUST [49] and compared clusters to the BovB consensus and centroid from each species.

### Clustering of nucleotide BovB sequences from bats and frogs

All intact BovB sequences > 2 kb and < 4 kb from bats, frogs and perissodactyls were grouped into a single file. We also added two RepBase equid sequences (RTE-1_EC and BovB_Ec) and 1 RepBase bat sequence (BovBa-1_EF) [20]. After clustering, we expected to find one family of equid BovBs, the equid RTE sequence as an outlier and numerous families containing bat and frog BovBs.

The actual findings are described in the text (Fig. 2b). We first used UCLUST [49] to cluster the sequences (function -cluster_fast with parameters -id, -uc, -clusters). The highest identity at which there were only two clusters/families was 40%. At higher identities, the equid BovBs stayed together but the bat and frog BovBs were lost as singletons. To confirm the clustering, we used MUSCLE [50] to align all the sequences and FastTree [52] to infer a maximum likelihood phylogeny (see Fig. 2b).

### Extraction of nucleotide ORFs and conserved amino acid residues

Starting with BovBs, USEARCH [49] was used to find all possible ORFs, with function -fastx_findorfs and parameters -aaout (for amino acid output), -ntout (for nucleotide output) and -orfstyle 7 (to allow non-standard start codons). Nucleotide ORFs were kept for later clustering. Amino acid ORFs were used to detect RT domains with HMMer [53]. RT domains were extracted using the envelope coordinates from the HMMer domain hits table (-domtblout), with minimum length 200 amino acid residues.

### All-against-all clustering using BLAST + SiLiX

We compiled all confirmed BovB and L1 nucleotide sequences into separate multi-fasta databases. For nucleotide sequences, the length cut-off was ≥ 2.4 kb and < 4 kb for BovBs; ≥ 3 kb and < 9 kb for L1s. BovBs were analysed first to identify characteristics of HT events.

To detect HT candidates, we initially used the all-against-all clustering strategy described in El Baidouri et al. [21]. Briefly, this method uses a nucleotide BLAST [22] to compare every individual sequence in a database against every other sequence; hence the term all-against-all. BLAST parameters were as follows: -r 2 (reward for nucleotide match, setting this to 2 is more adapted for divergent sequences); -e 1e-10 (e-value); -F F (filter query sequence = false); -m 8 (for tabular output). The SiLiX program [23] was then used to filter the BLAST output and produce clusters or families that met the designated identity threshold.

### All-against-all clustering using VSEARCH

The BLAST+SiLiX method worked well for recent HT events (e.g. BovB transfer between bed bug and snakes) but failed to pick up ancient HT events. For comparison, we also tested VSEARCH [24]: an open source version of USEARCH [49] that is orders of magnitude faster than BLAST [22] and uses centroid/average linkage to identify clusters. As before, we used our entire database of BovB nucleotide sequences as input to VSEARCH, at clustering identities of 50–90%.

The majority of clusters contained several copies of the same BovB family from a single species, indicative of vertical inheritance. We found that using a lower identity threshold was more informative for capturing ancient HT events. At 50–60% identity, the clustering preserved the recent, high-identity HT events while also finding the ancient, lower-identity HT events. We concluded that these were the best % identities to use for our particular dataset, considering it includes widely divergent branches of Eukaryota.

Clusters were deemed HT candidates if they contained BovB elements belonging to at least two different species. This left thousands of possible HT candidates. To find the most compelling HT clusters, we went one step further and kept only the clusters which demonstrated cross-Class transfer (e.g. BovBs from Mammalia and Insecta in the same cluster). All potential HT candidates were validated by checking that they were not located on short, unplaced scaffolds or contigs in the genome. The flanking regions of each HT candidate pair were extracted and checked (via pairwise alignment) to ensure that high sequence identity was restricted to the BovB region. This was done to check for contamination or orthologous regions. Phylogenies of HT candidate clusters were inferred using maximum likelihood and neighbour-joining methods (1000 bootstraps).

As an extra step, we used VSEARCH [24] to perform an all-against-all clustering of the extracted nucleotide ORF sequences and USEARCH [49] to perform all-against-all clustering of the extracted amino acid RT domains (note that VSEARCH does not currently support amino acid sequences).

The entire process was then repeated with L1s (for nucleotide L1s, then nucleotide ORFs, then amino acid RT domains). Additional file 1: Tables S5 and S6 show the HT clusters for BovB and L1, respectively. Nucleotide sequence clusters are prefixed with c (e.g. c_*), nucleotide ORF clusters are prefixed with o (e.g. o_*) and amino acid RT clusters are prefixed with r (e.g. r_*).

### Kimura divergence estimates for species containing both TEs

To compare TE dynamics within these species, we used RepeatMasker [54] to compare L1 and BovB nucleotide sequences from each genome against the super consensus library of repeats curated by RepBase [20]. Kimura substitution levels were calculated from the alignments using the provided RepeatMasker utility scripts [54]. Additional file 2: Figures S3–S54 show the resulting plots.

## Additional files

Additional file 1: Tables describing the source and assembly version used for each of the 759 genomes (**Table S1.**) the L1 and BovB content of each genome (**Tables S2.** and **S3.** respectively), the estimated genome coverage of L1 and BovB elements for each species (**Table S4.**) and putative horizontal transfer clusters involving BovB and L1 (**Tables S5. and S6.** respectively). (PDF 466 kb)

Additional file 2: **Figures S1.** and **S2.** show additional discordant L1 clusters. **Figures S3–S54.** show Kimura divergence plots for species containing both L1 and BovB elements. **Figure S55** shows the location of the chimeric L1-BovB element in the cow genome (*Bos taurus*), showing that there is little evidence of transcription. (PDF 823 kb)

## References

[1] de Koning AP, Gu W, Castoe TA, Batzer MA, Pollock DD (2011). Repetitive elements may comprise over two-thirds of the human genome. PLoS Genet.

[2] Piskurek O, Jackson DJ (2012). Transposable elements: from DNA parasites to architects of metazoan evolution. Genes.

[3] Feschotte C (2008). Transposable elements and the evolution of regulatory networks. Nat Rev Genet.

[4] Kemp JR, Longworth MS (2015). Crossing the LINE toward genomic instability: LINE-1 retrotransposition in cancer. Front Chem.

[5] Goodier JL (2016). Restricting retrotransposons: a review. Mob DNA.

[6] Solyom S, Kazazian HH (2012). Mobile elements in the human genome: implications for disease. Genome Med.

[7] Richardson SR, Doucet AJ, Kopera HC, Moldovan JB, Garcia-Perez JL, Moran JV (2015). The influence of LINE-1 and SINE retrotransposons on mammalian genomes. Microbiol Spectr.

[8] Walsh AM, Kortschak RD, Gardner MG, Bertozzi T, Adelson DL (2013). Widespread horizontal transfer of retrotransposons. Proc Natl Acad Sci U S A.

[9] Ivancevic AM, Walsh AM, Kortschak RD, Adelson DL (2013). Jumping the fine LINE between species: horizontal transfer of transposable elements in animals catalyses genome evolution. Bioessays.

[10] Gilbert C, Feschotte C (2018). Horizontal acquisition of transposable elements and viral sequences: patterns and consequences. Curr Opin Genet Dev.

[11] Peccoud J, Loiseau V, Cordaux R, Gilbert C (2017). Massive horizontal transfer of transposable elements in insects. Proc Natl Acad Sci U S A.

[12] Sormacheva I, Smyshlyaev G, Mayorov V, Blinov A, Novikov A, Novikovaz O (2012). Vertical evolution and horizontal transfer of CR1 non-LTR retrotransposons and Tc1/mariner DNA transposons in Lepidoptera species. Mol Biol Evol.

[13] Suh A, Witt CC, Menger J, Sadanandan KR, Podsiadlowski L, Gerth M (2016). Ancient horizontal transfers of retrotransposons between birds and ancestors of human pathogenic nematodes. Nat Commun.

[14] Kordis D, Gubensek F (1999). Horizontal transfer of non-LTR retrotransposons in vertebrates. Genetica.

[15] Gao D, Chu Y, Xia H, Xu C, Heyduk K, Abernathy B (2018). Horizontal transfer of non-LTR retrotransposons from arthropods to flowering plants. Mol Biol Evol.

[16] Waters PD, Dobigny G, Waddell PJ, Robinson TJ (2007). Evolutionary history of LINE-1 in the major clades of placental mammals. PLoS One.

[17] Schaack S, Gilbert C, Feschotte C (2010). Promiscuous DNA: horizontal transfer of transposable elements and why it matters for eukaryotic evolution. Trends Ecol Evol.

[18] Adelson DL, Raison JM, Edgar RC (2009). Characterization and distribution of retrotransposons and simple sequence repeats in the bovine genome. Proc Natl Acad Sci U S A.

[19] Gilbert C, Schaack S, Pace JK, Brindley PJ, Feschotte C (2010). A role for host-parasite interactions in the horizontal transfer of transposons across phyla. Nature.

[20] Jurka J, Kapitonov VV, Pavlicek A, Klonowski P, Kohany O, Walichiewicz J (2005). Repbase update, a database of eukaryotic repetitive elements. Cytogenet Genome Res.

[21] El Baidouri M, Carpentier MC, Cooke R, Gao D, Lasserre E, Llauro C (2014). Widespread and frequent horizontal transfers of transposable elements in plants. Genome Res.

[22] Altschul SF, Gish W, Miller W, Myers EW, Lipman DJ (1990). Basic local alignment search tool. J Mol Biol.

[23] Miele V, Penel S, Duret L (2011). Ultra-fast sequence clustering from similarity networks with SiLiX. BMC Bioinformatics.

[24] Rognes T, Flouri T, Nichols B, Quince C, Mahe F (2016). VSEARCH: a versatile open source tool for metagenomics. PeerJ.

[25] Kumar S, Hedges SB (2011). TimeTree2: species divergence times on the iPhone. Bioinformatics.

[26] Christensen S, Pont-Kingdon G, Carroll D (2000). Comparative studies of the endonucleases from two related Xenopus laevis retrotransposons, Tx1L and Tx2L: target site specificity and evolutionary implications. Genetica.

[27] Ivancevic AM, Kortschak RD, Bertozzi T, Adelson DL (2016). LINEs between species: evolutionary dynamics of LINE-1 retrotransposons across the eukaryotic tree of life. Genome Biol Evol.

[28] Kapusta A, Suh A, Feschotte C (2017). Dynamics of genome size evolution in birds and mammals. Proc Natl Acad Sci U S A.

[29] Cantrell MA, Scott L, Brown CJ, Martinez AR, Wichman HA (2008). Loss of LINE-1 activity in the megabats. Genetics.

[30] Soucy SM, Huang J, Gogarten JP (2015). Horizontal gene transfer: building the web of life. Nat Rev Genet.

[31] Olival KJ, Hosseini PR, Zambrana-Torrelio C, Ross N, Bogich TL, Daszak P (2017). Host and viral traits predict zoonotic spillover from mammals. Nature.

[32] Calisher CH, Childs JE, Field HE, Holmes KV, Schountz T (2006). Bats: important reservoir hosts of emerging viruses. Clin Microbiol Rev.

[33] Tang Z, Zhang HH, Huang K, Zhang XG, Han MJ, Zhang Z (2015). Repeated horizontal transfers of four DNA transposons in invertebrates and bats. Mob DNA.

[34] Venner S, Miele V, Terzian C, Biemont C, Daubin V, Feschotte C (2017). Ecological networks to unravel the routes to horizontal transposon transfers. PLoS Biol.

[35] McClintock B (1984). The significance of responses of the genome to challenge. Science.

[36] Lindblad-Toh K, Garber M, Zuk O, Lin MF, Parker BJ, Washietl S (2011). A high-resolution map of human evolutionary constraint using 29 mammals. Nature.

[37] Chuong EB, Elde NC, Feschotte C (2016). Regulatory evolution of innate immunity through co-option of endogenous retroviruses. Science.

[38] Lynch VJ (2016). GENETICS. A copy-and-paste gene regulatory network. Science.

[39] Lynch VJ, Leclerc RD, May G, Wagner GP (2011). Transposon-mediated rewiring of gene regulatory networks contributed to the evolution of pregnancy in mammals. Nat Genet.

[40] Parrish NF, Tomonaga K (2016). Endogenized viral sequences in mammals. Curr Opin Microbiol.

[41] Sasaki T, Nishihara H, Hirakawa M, Fujimura K, Tanaka M, Kokubo N (2008). Possible involvement of SINEs in mammalian-specific brain formation. Proc Natl Acad Sci U S A.

[42] Boto L (2014). Horizontal gene transfer in the acquisition of novel traits by metazoans. Proc Biol Sci.

[43] Imbeault M, Helleboid PY, Trono D (2017). KRAB zinc-finger proteins contribute to the evolution of gene regulatory networks. Nature.

[44] Ecco G, Cassano M, Kauzlaric A, Duc J, Coluccio A, Offner S (2016). Transposable elements and their KRAB-ZFP controllers regulate gene expression in adult tissues. Dev Cell.

[45] Chuong EB, Elde NC, Feschotte C (2017). Regulatory activities of transposable elements: from conflicts to benefits. Nat Rev Genet.

[46] Ivancevic A, Kortschak RD, Bertozzi T, Adelson DL (2018). Dataset from: Horizontal transfer of BovB and L1 retrotransposons in eukaryotes [Data set] Zenodo.

[47] Harris RS. Improved Pairwise Alignment of Genomic DNA Ph.D. Thesis. Pennsylvania: Pennsylvania State University; 2007.

[48] Kohany O, Gentles AJ, Hankus L, Jurka J (2006). Annotation, submission and screening of repetitive elements in Repbase: RepbaseSubmitter and Censor. BMC Bioinformatics.

[49] Edgar RC (2010). Search and clustering orders of magnitude faster than BLAST. Bioinformatics.

[50] Edgar RC (2004). MUSCLE: multiple sequence alignment with high accuracy and high throughput. Nucleic Acids Res.

[51] Castresana J (2000). Selection of conserved blocks from multiple alignments for their use in phylogenetic analysis. Mol Biol Evol.

[52] Price MN, Dehal PS, Arkin AP (2009). FastTree: computing large minimum evolution trees with profiles instead of a distance matrix. Mol Biol Evol.

[53] Finn RD, Clements J, Eddy SR (2011). HMMER web server: interactive sequence similarity searching. Nucleic Acids Res.

[54] Tarailo-Graovac M, Chen N (2009). Using RepeatMasker to identify repetitive elements in genomic sequences. Curr Protoc Bioinformatics.

[55] Benson DA, Cavanaugh M, Clark K, Karsch-Mizrachi I, Ostell J, Pruitt KD (2018). GenBank. Nucleic Acids Res.

[56] Kent WJ, Sugnet CW, Furey TS, Roskin KM, Pringle TH, Zahler AM (2002). The human genome browser at UCSC. Genome Res.

[57] Ivancevic A, Kortschak RD, Bertozzi T, Adelson DL. AdelaideBioinfo/horizontalTransfer: first release of horizontal transfer code (version v1.0.0). Adelaide: Zenodo; 2018. 10.5281/zenodo.1246999

[58] Letunic I, Bork P (2016). Interactive tree of life (iTOL) v3: an online tool for the display and annotation of phylogenetic and other trees. Nucleic Acids Res.

[59] The Tree of Life Web Project. http://tolweb.org. Accessed Oct 2017.

[60] Zdobnov EM, Tegenfeldt F, Kuznetsov D, Waterhouse RM, Simao FA, Ioannidis P (2017). OrthoDB v9.1: cataloging evolutionary and functional annotations for animal, fungal, plant, archaeal, bacterial and viral orthologs. Nucleic Acids Res.

