# Supplementary material for: Horizontal transfer of BovB and L1 retrotransposons in eukaryotes
Source: Genome Biol. 2018 Jul 9;19:85. doi: 10.1186/s13059-018-1456-7 (PMC6036668; doi:10.1186/s13059-018-1456-7)
Supplement: Supplementary file 1 — Tables describing the source and assembly version used for each of the 759 genomes (Table S1.) the L1 and BovB content of each genome (Tables S2. and S3. respectively), the estimated genome coverage of L1 and BovB elements for each species (Table S4.) and putative horizontal transfer clusters involving BovB and L1 (Tables S5. and S6. respectively). (PDF 466 kb) [file 13059_2018_1456_MOESM1_ESM.pdf]

Supplementary Tables

Table S1: Our dataset of public domain genomes **2**

Table S2: L1s in genomic data **40**

Table S3: BovBs in genomic data **60**

Table S4: Genome coverage of L1 and BovB elements **80**

Table S5: BovB HT candidate clusters **95**

Table S6: L1 HT candidate clusters **102**

Table S1: Our dataset of public domain genomes

Table S1: Shows the systematic name, common name, genome version, source and submitter for the 759 genomes used in this study. Almost all are in the public domain; only four genomes were acquired through collaborators, these are marked 'Private' in the source column. Headings indicate Kingdom (METAZOA, FUNGI or VIRIDIPLANTAE), followed by Class (e.g. MAMMALIA) and Order (e.g. Monotremata).

No	Systematic Name	Common Name	Genome Version	Source	Submitter
**METAZOA**					
**MAMMALIA**					
**Monotremata**					
1	*Tachyglossus aculeatus*	Echidna	Tachyglossus	Private	-
2	*Ornithorhynchus anatinus*	Platypus	ornAna1	UCSC	WashU
**Marsupialia**					
3	*Monodelphis domestica*	Opossum	monDom5	UCSC	GAT
4	*Macropus eugenii*	Tammar wallaby	Meug_1.1	NCBI	Tammar Wallaby Genome Sequencing Consortium
5	*Sarcophilus harrisii*	Tasmanian devil	sarHar1	UCSC	WTSI
**Xenarthra**					
6	*Dasypus novemcinctus*	Armadillo	Dasnov3.0	NCBI	BCM
7	*Choloepus hoffmanni*	Sloth	choHof1	UCSC	Broad
**Afrotheria**					
8	*Chrysochloris asiatica*	Cape golden mole	ChrAsi1.0	NCBI	Broad
9	*Echinops telfairi*	Tenrec	EchTel2.0	NCBI	Broad
10	*Orycteropus afer afer*	Aardvark	OryAfe1.0	NCBI	Broad
11	*Elephantulus edwardii*	Cape elephant shrew	EleEdw1.0	NCBI	Broad
12	*Trichechus manatus latirostris*	Manatee	TriManLat1.0	NCBI	Broad
13	*Procavia capensis*	Rock hyrax	proCap1	UCSC	BCM
14	*Loxodonta africana*	Elephant	LAv4	Private	-
**Insectivora**					
15	*Erinaceus europaeus*	Hedgehog	EriEur2.0	NCBI	Broad
16	*Sorex araneus*	Common shrew	SorAra2.0	NCBI	Broad
17	*Condylura cristata*	Star-nosed mole	ConCri1.0	NCBI	Broad
**Chiroptera**					
18	*Pteropus alecto*	Black flying fox	ASM32557v1	NCBI	BGI
19	*Pteropus vampyrus*	Megabat	pteVam1	UCSC	BCM
20	*Eidolon helvum*	Straw-coloured fruit bat	ASM46528v1	NCBI	QMUL
21	*Megaderma lyra*	Greater false vampire bat	ASM46534v1	NCBI	QMUL
22	*Rhinolophus ferrumequinum*	Greater horseshoe bat	ASM46549v1	NCBI	QMUL

No	Systematic Name	Common Name	Genome Version	Source	Submitter
23	*Pteronotus parnellii*	Parnells mustached bat	ASM46540v1	NCBI	QMUL
24	*Eptesicus fuscus*	Big brown bat	EptFus1.0	NCBI	Broad
25	*Myotis brandtii*	Brandts bat	ASM41265v1	NCBI	BGI
26	*Myotis davidii*	Mouse-eared bat	ASM32734v1	NCBI	BGI
27	*Myotis lucifugus*	Microbat	Myoluc2.0	NCBI	Broad
**Perissodactyla**					
28	*Ceratotherium simum simum*	White rhino	CerSimSim1.0	NCBI	Broad
29	*Equus przewalskii*	Przewalski horse	Burgud	NCBI	IMAU
30	*Equus caballus* (Thoroughbred)	Thoroughbred horse	equCab2	UCSC	GAT
31	*Equus caballus* (Mongolian)	Mongolian horse	Ajinai1.0	NCBI	IMAU
**Pholidota**					
32	*Manis pentadactyla*	Chinese pangolin	M_pentadactyla-1.1.1	NCBI	WashU
**Carnivora**					
33	*Felis catus*	Cat	felCat5	UCSC	International Cat Genome Sequencing Consortium
34	*Panthera tigris altaica*	Siberian tiger	PanTig1.0	NCBI	Personal Genomics Institute
35	*Canis lupus familiaris*	Dog	CanFam3.1	NCBI	Dog Genome Sequencing Consortium
36	*Ursus maritimus*	Polar bear	UrsMar_1.0	NCBI	BGI
37	*Ailuropoda melanoleuca*	Panda	ailMel1	UCSC	BGI
38	*Leptonychotes weddellii*	Weddell seal	LepWed1.0	NCBI	Broad
39	*Odobenus rosmarus divergens*	Walrus	Oros_1.0	NCBI	Marine Mammals
40	*Mustela putorius furo*	Ferret	MusPutFur1.0	NCBI	Ferret Genome Sequencing Consortium
**Cetartiodactyla**					
41	*Camelus dromedarius*	Dromedary	PRJNA234474_ Ca_dromedarius_V1.0	NCBI	King Abdulaziz City for Science and Technology
42	*Camelus ferus*	Bactrian camel	CB1	NCBI	Bactrian Camels Genome Sequencing and Analysis Consortium
43	*Vicugna pacos*	Alpaca	Vicugna_pacos-2.0.1	NCBI	WashU

No	Systematic Name	Common Name	Genome Version	Source	Submitter
44	*Sus scrofa* (Duroc)	Duroc pig	Sscrofa10.2	NCBI	Swine Genome Sequencing Consortium
45	*Sus scrofa* (Tibetan)	Tibetan pig	Tibetan_Pig_v1.0	NCBI	Novogene
46	*Sus scrofa* (Ellegaard Gottingen minipig)	Minipig	SscrofaMinipig	NCBI	GlaxoSmithK-line
47	*Balaenoptera acutorostrata scammoni*	Minke whale	BalAcu1.0	NCBI	Korea Ocean Research & Development Institute
48	*Physeter catodon*	Sperm whale	Physeter_macrocephalus-2.0.2	NCBI	AGM
49	*Lipotes vexillifer*	Baiji (Chinese dolphin)	Lipotes_vexillifer_v1	NCBI	BGI
50	*Tursiops truncatus*	Bottlenose dolphin	Ttru_1.4	NCBI	BCM
51	*Orcinus orca*	Killer whale	Oorc_1.1	NCBI	Marine Mammals
52	*Pantholops hodgsonii*	Tibetan antelope	PHO1.0	NCBI	BGI
53	*Capra hircus*	Goat	CHIR_1.0	NCBI	International Goat Genome Consortium
54	*Ovis aries* (Texel)	Domestic sheep	oviAri3	UCSC	International Sheep Genome Consortium
55	*Ovis aries musimon*	Wild sheep	Oori1	NCBI	EBI
56	*Bubalus bubalis*	Water buffalo	UMD_CASPUR_WB_2.0	NCBI	Mary
57	*Bison bison bison*	Bison	Bison_UMD1.0	NCBI	Mary
58	*Bos mutus*	Yak	BosGru_v2.0	NCBI	BGI
59	*Bos indicus*	Zebu	Bos_indicus_1.0	NCBI	Genoa Biotecnologia SA
60	*Bos taurus*	Cow	bosTau6	UCSC	Mary
**Lagomorpha**					
61	*Ochotona princeps*	American pika	OchPri3.0	NCBI	Broad
62	*Oryctolagus cuniculus*	Rabbit	oryCun2	UCSC	GSP
**Rodentia**					
63	*Ictidomys tridecemlineatus*	Squirrel	SpeTri2.0	NCBI	Broad
64	*Heterocephalus glaber*	Naked molerat	HetGla_female_1.0	NCBI	Broad
65	*Fukomys damarensis*	Damaraland molerat	DMR_v1.0	NCBI	BGI
66	*Cavia aperea*	Brazilian guinea pig	CavAp1.0	NCBI	Leibniz Institute for Zoo and Wildlife research
67	*Cavia porcellus*	Guinea pig	cavPor3	UCSC	GSP
68	*Chinchilla lanigera*	Chinchilla	ChiLan1.0	NCBI	Broad

No	Systematic Name	Common Name	Genome Version	Source	Submitter
69	*Octodon degus*	Degu	OctDeg1.0	NCBI	Broad
70	*Dipodomys ordii*	Kangaroo rat	dipOrd1	UCSC	BCM
71	*Jaculus jaculus*	Lesser Egyptian jerboa	JacJac1.0	NCBI	Broad
72	*Nannospalax galili*	Blind molerat	S.galili_v1.0	NCBI	BGI
73	*Mesocricetus auratus*	Golden hamster	MesAur1.0	NCBI	Broad
74	*Cricetulus griseus*	Chinese hamster	CriGri_1.0	NCBI	BGI
75	*Microtus ochrogaster*	Prairie vole	MicOch1.0	NCBI	Broad
76	*Peromyscus maniculatus bairdii*	Deer mouse	Pman_1.0	NCBI	BCM
77	*Rattus norvegicus*	Rat	Rnor_5.0	NCBI	Rat Genome Sequencing Consortium
78	*Mus musculus*	Mouse	GRCm38.p2	NCBI	GRC
**Scandentia**					
79	*Tupaia belangeri*	Tree shrew	tupBel1	UCSC	Broad
80	*Tupaia chinensis*	Chinese tree shrew	TupChi_1.0	NCBI	BGI
**Primates**					
81	*Galeopterus variegatus*	Flying lemur	G_variegatus-3.0.2	NCBI	WashU
82	*Otolemur garnettii*	Bushbaby	OtoGar3	NCBI	Broad
83	*Microcebus murinus*	Mouse lemur	micMur1	UCSC	GSP
84	*Tarsius syrichta*	Tarsier	Tarsius_syrichta-2.0.1	NCBI	WashU
85	*Callithrix jacchus*	Marmoset	Callithrix_jacchus-3.2	NCBI	WashU
86	*Saimiri boliviensis boliviensis*	Squirrel monkey	SaiBol1.0	NCBI	Broad
87	*Rhinopithecus roxellana*	Snub-nosed monkey	Rrox_v1	NCBI	Novogene
88	*Nasalis larvatus*	Proboscis monkey	Charlie1.0	NCBI	Proboscis Monkey Functional Genome Consortium
89	*Chlorocebus sabaeus*	Green monkey	Chlorocebus_sabeus 1.1	NCBI	Vervet Genomics Consortium
90	*Macaca fascicularis*	Crab-eating macaque	Macaca_fascicularis_5.0	NCBI	WashU
91	*Macaca mulatta*	Rhesus macaque	rheMac3	UCSC	BGI
92	*Papio anubis*	Baboon	Panu_2.0	NCBI	BCM
93	*Nomascus leucogenys*	Gibbon	nomLeu3	UCSC	Gibbon Genome Sequencing Consortium
94	*Pongo abelii*	Sumatran orangutan	P_pygmaeus_2.0.2	NCBI	Orangutan Genome Sequencing Consortium
95	*Gorilla gorilla gorilla*	Gorilla	gorGor3.1	NCBI	WTSI

No	Systematic Name	Common Name	Genome Version	Source	Submitter
96	*Pan paniscus*	Bonobo	panpan1	NCBI	MP
97	*Pan troglodytes*	Chimp	panTro4	UCSC	Chimpanzee Sequencing and Analysis Consortium
98	*Homo sapiens*	Human	GRCh37 (hg19)	NCBI	GRC
**SAUROPSIDA**					
**Testudines**					
99	*Apalone spinifera*	Spiny softshell turtle	ASM38561v1	NCBI	WUGSC
100	*Pelodiscus sinensis*	Chinese softshell turtle	PelSin_1.0	NCBI	P. sinensis Genome Project Consortium
101	*Chelonia mydas*	Green sea turtle	CheMyd_1.0	NCBI	BGI
102	*Chrysemys picta bellii*	Painted turtle	chrPic1	UCSC	Painted Turtle Genome Sequencing Consortium
**Struthioniformes**					
103	*Struthio camelus australis*	Ostrich	ASM69896v1	NCBI	BGI
**Tinamiformes**					
104	*Tinamus guttatus*	Tinamou	ASM70537v2	NCBI	BGI
**Anseriformes**					
105	*Anas platyrhynchos*	Mallard	BGI_duck_1.0	NCBI	State Key Laboratory for Agrobiotechnology, China Agricultural University, Beijing
**Galliformes**					
106	*Lyrurus tetrix tetrix*	Black grouse	tetTet1	NCBI	UU
107	*Gallus gallus*	Chicken	galGal4	UCSC	International Chicken Genome Consortium
108	*Coturnix japonica*	Japanese quail	Coja_1.0	NCBI	Tokyo University of Agriculture
109	*Meleagris gallopavo*	Turkey	melGal1	UCSC	Turkey Genome Consortium
110	*Colinus virginianus*	Bobwhite	NB1.1	NCBI	TAMU
**Passeriformes**					
111	*Acanthisitta chloris*	Rifleman	ASM69581v1	NCBI	BGI
112	*Manacus vitellinus*	Manakin	ASM69201v1	NCBI	BGI

No	Systematic Name	Common Name	Genome Version	Source	Submitter
113	*Zonotrichia albicollis*	White-throated sparrow	Zonotrichia_albicollis-1.0.1	NCBI	White-throated Sparrow Consortium
114	*Geospiza fortis*	Medium ground finch	geoFor1	UCSC	BGI
115	*Serinus canaria*	Atlantic canary	SCA1	NCBI	MP
116	*Taeniopygia guttata*	Zebra finch	taeGut1	UCSC	WashU
117	*Ficedula albicollis*	Collared flycatcher	FicAlb1.5	NCBI	UU
118	*Pseudopodoces humilis*	Ground tit	PseHum1.0	NCBI	BGI
119	*Corvus brachyrhynchos*	American crow	ASM69197v1	NCBI	BGI
120	*Corvus cornix cornix*	Hooded crow	Hooded_Crow_genome	NCBI	UU
**Psittaciformes**					
121	*Ara macao*	Scarlet macaw	SMACv1.1	NCBI	TAMU
122	*Amazona vittata*	Puerto Rican parrot	AV1	NCBI	Puerto Rican Parrot Genome Project
123	*Melopsittacus undulatus*	Budgerigar	melUnd1	UCSC	WashU
124	*Nestor notabilis*	Kea	ASM69687v1	NCBI	BGI
**Falconiformes**					
125	*Falco cherrug*	Saker falcon	F_cherrug_v1.0	NCBI	BGI
126	*Falco peregrinus*	Peregrine falcon	F_peregrinus_v1.0	NCBI	BGI
**Cariamiformes**					
127	*Cariama cristata*	Seriema	ASM69053v1	NCBI	BGI
**Coraciiformes**					
128	*Merops nubicus*	Bee eater	ASM69184v1	NCBI	BGI
**Piciformes**					
129	*Picoides pubescens*	Woodpecker	ASM69900v1	NCBI	BGI
**Bucerotiformes**					
130	*Buceros rhinoceros silvestris*	Hornbill	ASM71030v1	NCBI	BGI
**Trogoniformes**					
131	*Apaloderma vittatum*	Trogon	ASM70340v1	NCBI	BGI
**Leptosomiformes**					
132	*Leptosomus discolour*	Cuckoo roller	ASM69178v1	NCBI	BGI
**Accipitriformes**					
133	*Haliaeetus albicilla*	White-tailed eagle	ASM69140v1	NCBI	BGI
134	*Haliaeetus leucocephalus*	Bald eagle	Haliaeetus_leucocephalus-4.0	NCBI	The Bald Eagle Consortium
135	*Aquila chrysaetos canadensis*	Golden eagle	Aquila_chrysaetos-1.0.2	NCBI	WashU
136	*Cathartes aura*	Turkey vulture	ASM69994v1	NCBI	BGI
**Strigiformes**					
137	*Tyto alba*	Barn owl	ASM68720v1	NCBI	BGI
**Coliiformes**					
138	*Colius striatus*	Mousebird	ASM69071v1	NCBI	BGI
**Charadriiformes**					

No	Systematic Name	Common Name	Genome Version	Source	Submitter
139	*Charadrius vociferus*	Killdeer	ASM70802v2	NCBI	BGI
**Gruiformes**					
140	*Balearica regulorum gibbericeps*	Grey crane	ASM70989v1	NCBI	BGI
141	*Chlamydotis macqueenii*	MacQueen's bustard	ASM69519v1	NCBI	BGI
**Cuculiformes**					
142	*Cuculus canorus*	Common cuckoo	ASM70932v1	NCBI	BGI
**Procellariiformes**					
143	*Fulmarus glacialis*	Fulmar	ASM69083v1	NCBI	BGI
**Sphenisciformes**					
144	*Aptenodytes forsteri*	Emperor penguin	ASM69914v1	NCBI	BGI
145	*Pygoscelis adeliae*	Adelie penguin	ASM69910v1	NCBI	BGI
**Pelecaniformes**					
146	*Phalacrocorax carbo*	Black cormorant	ASM70892v1	NCBI	BGI
147	*Pelecanus crispus*	Pelican	ASM68737v1	NCBI	BGI
148	*Nipponia nippon*	Ibis	ASM70822v1	NCBI	BGI
149	*Egretta garzetta*	Egret	ASM68718v1	NCBI	College of Medicine and Forensics, Xi'an Jiaotong University
150	*Phaethon lepturus*	Tropicbird	ASM68728v1	NCBI	BGI
**Gaviiformes**					
151	*Gavia stellata*	Loon	ASM69087v1	NCBI	BGI
**Musophagiformes**					
152	*Tauraco erythrolophus*	Turaco	ASM70936v1	NCBI	BGI
**Opisthocomiformes**					
153	*Opisthocomus hoazin*	Hoatzin	ASM69207v1	NCBI	BGI
**Columbiformes**					
154	*Columba livia*	Rock dove	Cliv_1.0	NCBI	BGI
**Pteroclidiformes**					
155	*Pterocles gutturalis*	Sandgrouse	ASM69924v1	NCBI	BGI
**Apodiformes**					
156	*Calypte anna*	Anna's hummingbird	ASM69908v1	NCBI	BGI
157	*Chaetura pelagica*	Chimney swift	ChaPel_1.0	NCBI	BGI
**Caprimulgiformes**					
158	*Caprimulgus carolinensis*	Chuck-will's-widow	ASM70074v1	NCBI	BGI
**Eurypygiformes**					
159	*Eurypyga helias*	Sunbittern	ASM69077v1	NCBI	BGI
**Mesitornithiformes**					
160	*Mesitornis unicolor*	Mesite	ASM69576v1	NCBI	BGI
**Podicipediformes**					
161	*Podiceps cristatus*	Grebe	ASM69954v1	NCBI	BGI
**Phoenicopteriformes**					

No	Systematic Name	Common Name	Genome Version	Source	Submitter
162	*Phoenicopterus ruber ruber*	Flamingo	ASM68726v1	NCBI	BGI
**Crocodilia**					
163	*Alligator mississippiensis*	American alligator	allMis1	UCSC	ICGWG
164	*Alligator sinensis*	Chinese alligator	ASM45574v1	NCBI	BGI
165	*Crocodylus porosus*	Saltwater crocodile	Cpor_2.0	NCBI	ICGWG
166	*Gavialis gangeticus*	Gharial crocodile	ggan_v0.2	NCBI	ICGWG
**Squamata**					
167	*Pogona vitticeps*	Bearded dragon	Pogona_vitticeps.male	Private (Terry)	-
168	*Anolis carolinensis*	Anole lizard	anoCar2	UCSC	Broad
169	*Vipera berus berus*	Common European viper	Vber.be_1.0	NCBI	BCM-HGSC
170	*Crotalus mitchellii pyrrhus*	Pit viper	CrotMitch1.0	NCBI	Reed College
171	*Ophiophagus hannah*	King cobra	OphHan1.0	NCBI	Naturalis Biodiversity Center
172	*Python bivittatus*	Burmese python	Python_molurus_bivittatus-5.0.2	NCBI	The Consortium for Comparative Genomics, UC Denver
**AMPHIBIA**					
**Anura**					
173	*Nanorana parkeri*	Tibetan frog	ASM93562v1	NCBI	BGI
174	*Xenopus laevis*	African clawed frog	Xenopus_laevis_v2	NCBI	International Xenopus Sequencing Consortium
175	*Xenopus tropicalis*	Western clawed frog	Xtropicalis_v7	NCBI	JGI
**NEOPTERYGII**					
**Lepisosteiformes**					
176	*Lepisosteus oculatus*	Spotted gar	LepOcu1	NCBI	Broad
**Anguilliformes**					
177	*Anguilla anguilla*	European eel	Anguilla_anguilla_v1_09_nov_10	NCBI	ZF-S
178	*Anguilla japonica*	Japanese eel	japanese_eel_genome_v1_25_oct_2011_japonica_c401b400k25m200_sspacepremium k3a02n24_extra.final.scaffolds	NCBI	ZF-S
**Cypriniformes**					
179	*Danio rerio*	Zebrafish	danRer7	UCSC	WTSI

No	Systematic Name	Common Name	Genome Version	Source	Submitter
**Characiformes**					
180	*Astyanax mexicanus*	Mexican tetra	Astyanax_mexicanus-1.0.2	NCBI	AGM
**Beloniformes**					
181	*Oryzias latipes*	Medaka	ASM31367v1	NCBI	Medaka genome sequencing project
**Cyprinodontiformes**					
182	*Poecilia formosa*	Amazon molly	Poecilia_formosa-5.1.2	NCBI	AGM
183	*Xiphophorus maculatus*	Southern platyfish	Xiphophorus_maculatus-4.4.2	NCBI	The Genome Institute, Washington University at St. Louis
184	*Fundulus heteroclitus*	Mummichog	Fundulus_heteroclitus-3.0.2	NCBI	WashU
**Tetraodontiformes**					
185	*Takifugu flavidus*	Yellowbelly pufferfish	version 1 of Takifugu flavidus genome	NCBI	IOCAS
186	*Takifugu rubripes*	Fugu	fr3	UCSC	The Fugu Genome Sequencing Consortium
187	*Tetraodon nigroviridis*	Tetraodon	tetNig2	UCSC	Genoscope
**Pleuronectiformes**					
188	*Cynoglossus semilaevis*	Tongue sole	Cse_v1.0	NCBI	BGI
**Perciformes**					
189	*Haplochromis burtoni*	Burton's haplo	AstBur1.0	NCBI	Broad
190	*Pundamilia nyererei*	Flameback cichlid	PunNye1.0	NCBI	Broad
191	*Maylandia zebra*	Zebra mbuna	MetZeb1.1	NCBI	Broad
192	*Neolamprologus brichardi*	Fairy cichlid	NeoBri1.0	NCBI	Broad
193	*Oreochromis niloticus*	Nile tilapia	oreNil2	UCSC	Broad
194	*Sebastes nigrocinctus*	Tiger rockfish	Snig1.0	NCBI	USC
195	*Sebastes rubrivinctus*	Flag rockfish	SRub1.0	NCBI	USC
196	*Gasterosteus aculeatus*	Stickleback	gasAcu1	UCSC	Broad
**Gadiformes**					
197	*Gadus morhua*	Atlantic cod	gadMor1	UCSC	Genofisk
**CHONDRICHTHYES**					
**Chimaeriformes**					
198	*Callorhinchus milii*	Australian ghostshark	Callorhinchus_milii-6.1.3	NCBI	IMCB
**Carcharhiniformes**					
199	*Carcharhinus brachyurus*	Copper shark	shark_ass	Private (Terry)	-
**ECDYSOZOA**					
**Ephemeroptera**					

No	Systematic Name	Common Name	Genome Version	Source	Submitter
200	*Ephemera danica*	Green drake	Edan_1.0	NCBI	i5k
**Odonata**					
201	*Ladona fulva*	Dragonfly	Lful_1.0	NCBI	i5k
**Phthiraptera**					
202	*Pediculus humanus corporis*	Body louse	JCVI_LOUSE_1.0	NCBI	JCVI
**Thysanoptera**					
203	*Frankliniella occidentalis*	Flower thrips	Focc_1.0	NCBI	i5k
**Hemiptera**					
204	*Diaphorina citri*	Asian citrus psyllid	Diaci psyllid genome assembly version 1.1	NCBI	International Psyllid Genome Consortium
205	*Pachypsylla venusta*	Petiolegall psyllid	Pven_1.0	NCBI	i5k
206	*Acyrthosiphon pisum*	Pea aphid	Acyr_2.0	NCBI	BCM
207	*Nilaparvata lugens*	Brown planthopper	NilLug1.0	NCBI	Nilaparvata lugens Genome Consortium
208	*Oncopeltus fasciatus*	Milkweed bug	Ofas_1.0	NCBI	BCM-HGSC i5k
209	*Rhodnius prolixus*	Assassin bug	Rhodnius_prolixus-3.0.1	NCBI	WashU
210	*Cimex lectularius*	Bed bug	Clec_1.0	NCBI	i5k
**Coleoptera**					
211	*Onthophagus taurus*	Taurus scarab	Otau_1.0	NCBI	i5k
212	*Agrilus planipennis*	Emerald ash borer	Apla_1.0	NCBI	BCM-HGSC i5k
213	*Tribolium castaneum*	Red flour beetle	Tcas_3.0	NCBI	BCM
214	*Anoplophora glabripennis*	Asian long-horned beetle	Agla_1.0	NCBI	i5k
215	*Leptinotarsa decemlineata*	Colorado potato beetle	Ldec_1.5	NCBI	i5k
216	*Dendroctonus ponderosae*	Mountain pine beetle	DendPond_male_1.0	NCBI	The Tria Project: Mountain Pine Beetle System Genomics
**Strepsiptera**					
217	*Mengenilla moldrzyki*	Twisted-wing parasite	Memo_1.0	NCBI	Zoologisches Forschungsmu-seum Alexander Koenig
**Diptera**					
218	*Aedes aegypti*	Yellow fever mosquito	AaegL2	NCBI	TIGR
219	*Culex quinquefasciatus*	Southern house mosquito	CulPip1.0	NCBI	Broad
220	*Anopheles albimanus*	-	Anop_albi_ALBI9_A_V1	NCBI	Broad

No	Systematic Name	Common Name	Genome Version	Source	Submitter
221	*Anopheles arabiensis*	-	Anop_arab_DONG5_ A_V1	NCBI	Broad
222	*Anopheles atroparvus*	-	Anop_atro_EBRO_V1	NCBI	Broad
223	*Anopheles christyi*	-	Anop_chri_ ACHKN1017_V1	NCBI	Broad
224	*Anopheles culicifacies*	-	Anop_culi_species_A-37_1_V1	NCBI	Broad
225	*Anopheles darlingi*	-	A_darlingi_v1	NCBI	Laboratorio Nacional de Computacao Cientifica
226	*Anopheles dirus*	-	Anop_diru_ WRAIR2_V1	NCBI	Broad
227	*Anopheles epiroticus*	-	Anop_epir_ epiroticus2_V1	NCBI	Broad
228	*Anopheles farauti*	-	Anop_fara_FAR1_V2	NCBI	Broad
229	*Anopheles funestus*	-	Anop_fune_ FUMOZ_V1	NCBI	Broad
230	*Anopheles gambiae*	African malaria mosquito	anoGam1	UCSC	International Anopheles Genome Project
231	*Anopheles maculatus*	-	Anop_macu_ maculatus3_V1	NCBI	Broad
232	*Anopheles melas*	-	Anop_mela_ CM1001059_ A_V2	NCBI	Broad
233	*Anopheles merus*	-	Anop_meru_MAF_V1	NCBI	Broad
234	*Anopheles minimus*	-	Anop_mini_ MINIMUS1_V1	NCBI	Broad
235	*Anopheles quadriannulatus*	-	Anop_quad_ QUAD4_A_V1	NCBI	Broad
236	*Anopheles sinensis*	-	AS2	NCBI	Nanjing Medical University
237	*Anopheles stephensi*	-	ASM30077v2	NCBI	Virginia Tech
238	*Mayetiola destructor*	Hessian fly	Mdes_1.0	NCBI	BCM
239	*Lutzomyia longipalpis*	Sand fly	Llon_1.0	NCBI	BCM
240	*Phlebotomus papatasi*	-	Ppap_1.0	NCBI	WashU
241	*Ceratitis capitata*	Med fly	Ccap_1.0	NCBI	i5k
242	*Drosophila albomicans*	-	DroAlb_1.0	NCBI	Kunming Institute of Zoology, Chinese Academy of Sciences
243	*Drosophila ananassae*	-	droAna3	UCSC	Agen
244	*Drosophila biarmipes*	-	Dbia_2.0	NCBI	BCM
245	*Drosophila bipectinata*	-	Dbip_2.0	NCBI	BCM
246	*Drosophila elegans*	-	Dele_2.0	NCBI	BCM

No	Systematic Name	Common Name	Genome Version	Source	Submitter
247	*Drosophila erecta*	-	droEre2	UCSC	Agen
248	*Drosophila eugracilis*	-	Deug_2.0	NCBI	modENCODE
249	*Drosophila ficusphila*	-	Dfic_2.0	NCBI	BCM
250	*Drosophila grimshawi*	-	droGri2	UCSC	Agen
251	*Drosophila kikkawai*	-	Dkik_2.0	NCBI	BCM
252	*Drosophila melanogaster*	-	Release 6 plus ISO1 MT	NCBI	The FlyBase Consortium/Berkeley Drosophila Genome Project/Celera Genomics
253	*Drosophila miranda*	-	DroMir_2.2	NCBI	University of California, Berkeley
254	*Drosophila mojavensis*	-	droMoj3	UCSC	Agen
255	*Drosophila persimilis*	-	droPer1	UCSC	Broad
256	*Drosophila pseudoobscura pseudoobscura*	-	Dpse_3.0	NCBI	BCM
257	*Drosophila rhopaloa*	-	Drho_2.0	NCBI	modENCODE
258	*Drosophila sechellia*	-	droSec1	UCSC	Broad
259	*Drosophila simulans*	-	dsim_caf1	NCBI	WashU
260	*Drosophila suzukii*	-	Dsuzukii.v01	NCBI	BGI
261	*Drosophila takahashii*	-	Dtak_2.0	NCBI	BCM
262	*Drosophila virilis*	-	droVir3	UCSC	Agen
263	*Drosophila willistoni*	-	dwil_caf1	NCBI	JCVI
264	*Drosophila yakuba*	-	dyak_caf1	NCBI	FlyBase
265	*Musca domestica*	House fly	Musca_domestica-2.0.2	NCBI	GGC
266	*Glossina austeni*	Tsetse fly	Glossina_austeni-1.0.3	NCBI	GGC
267	*Glossina brevipalpis*	-	Glossina_brevipalpis_1.0.3	NCBI	GGC
268	*Glossina fuscipes fuscipes*	-	Glossina_fuscipes-3.0.2	NCBI	GGC
269	*Glossina morsitans morsitans*	-	ASM107743v1	NCBI	WTSI
270	*Glossina pallidipes*	-	Glossina_pallidipes-1.0.3	NCBI	GGC
**Trichoptera**					
271	*Limnephilus lunatus*	Caddis fly	Llun_1.0	NCBI	i5k
**Lepidoptera**					
272	*Papilio glaucus*	Tiger butterfly	pgl_assembly_v1	NCBI	UT Southwestern Medical Center
273	*Papilio polytes*	Mormon butterfly	Ppol_1.0	NCBI	TokyoTech
274	*Papilio xuthus*	Swallowtail butterfly	Pxut_1.0	NCBI	TokyoTech

No	Systematic Name	Common Name	Genome Version	Source	Submitter
275	*Heliconius melpomene melpomene*	Postman butterfly	ASM31383v2	NCBI	Heliconius Genome Sequencing Consortium
276	*Melitaea cinxia*	Glanville fritillary	MelCinx1.0	NCBI	University of Helsinki
277	*Danaus plexippus*	Monarch butterfly	DanPle_1.0	NCBI	The Reppert Lab
278	*Bombyx mori*	Silkworm	ASM15162v1	NCBI	The International Silkworm Genome Sequencing Consortium
279	*Manduca sexta*	Tobacco hornworm	Msex_1.0	NCBI	BCM
280	*Plutella xylostella*	Diamondback moth	DBM_FJ_V1.1	NCBI	Plutella xylostella Genome Consortium
**Hymenoptera**					
281	*Athalia rosae*	Turnip sawfly	Aros_1.0	NCBI	i5k
282	*Cephus cinctus*	Wheat stem sawfly	Ccin1	NCBI	UIUC
283	*Orussus abietinus*	Parasitic wood wasp	Oabi_1.0	NCBI	i5k
284	*Ceratosolen solmsi marchali*	Pollinating wasp	CerSol_1.0	NCBI	Ceratosolen solmsi Genome Consortium
285	*Nasonia giraulti*	Jewel wasp	Ngir_1.0	NCBI	BCM
286	*Nasonia longicornis*	-	Nlon_1.0	NCBI	BCM
287	*Nasonia vitripennis*	-	Nvit_2.1	NCBI	BCM
288	*Copidosoma floridanum*	Looper parasitoid wasp	Cflo_1.0	NCBI	i5k
289	*Trichogramma pretiosum*	-	Tpre_1.0	NCBI	i5k
290	*Microplitis demolitor*	-	Mdem1	NCBI	UIUC
291	*Megachile rotundata*	Alfalfa leafcutter bee	MROT_1.0	NCBI	University of Maryland
292	*Apis dorsata*	Giant honey bee	Apis dorsata 1.3	NCBI	Cold Spring Harbor Laboratory
293	*Apis florea*	Dwarf honey bee	Aflo_1.0	NCBI	BCM
294	*Apis mellifera*	Western honey bee	Amel_4.5	NCBI	Human Genome Sequencing Center

No	Systematic Name	Common Name	Genome Version	Source	Submitter
295	*Bombus impatiens*	Common Eastern bumblebee	BIMP_2.0	NCBI	Biotechnology Center, University of Illinois
296	*Bombus terrestris*	Buff-tailed bumblebee	Bter_1.0	NCBI	BCM
297	*Linepithema humile*	Argentine ant	Lhum_UMD_V04	NCBI	AGC
298	*Camponotus floridanus*	Florida carpenter ant	CamFlo_1.0	NCBI	BGI
299	*Acromyrmex echinatior*	Panamanian leafcutter ant	Aech_3.9	NCBI	BGI
300	*Atta cephalotes*	Leafcutter ant	Attacep1.0	NCBI	WashU
301	*Solenopsis invicta*	Red imported fire ant	Si_gnG	NCBI	UNIL
302	*Pogonomyrmex barbatus*	Red harvester ant	Pbar_UMD_V03	NCBI	AGC
303	*Harpegnathos saltator*	Jumping ant	HarSal_1.0	NCBI	BGI
304	*Cerapachys biroi*	Clonal raider ant	CerBir1.0	NCBI	BGI
**Blattodea**					
305	*Blattella germanica*	German cockroach	Bger_1.0	NCBI	i5k
**Isoptera**					
306	*Zootermopsis nevadensis*	Eusocial termite	ZooNev1.0	NCBI	BGI
**Orthoptera**					
307	*Locusta migratoria*	Migratory locust	LocustGenomeV1	NCBI	Chinese Academy of Sciences
**Diplostraca**					
308	*Daphnia pulex*	Water flea	V1.0	NCBI	JGI
**Calanoida**					
309	*Eurytemora affinis*	Copepod	Eaff_1.0	NCBI	i5k
**Siphonostomatoida**					
310	*Lepeophtheirus salmonis*	Salmon louse	lsal_atl_canada_female_ v1	NCBI	Jong S Leong, Ben F Koop, Eric B Rondeau
**Amphipoda**					
311	*Hyalella azteca*	Scud	Hazt_1.0	NCBI	BCM
**Geophilomorpha**					
312	*Strigamia maritima*	European centipede	Smar_1.0	NCBI	BCM
**Araneae**					
313	*Stegodyphus mimosarum*	Social spider	Stegodyphus_ mimosarum_ v1	NCBI	BGI
314	*Latrodectus hesperus*	Black widow	Lhes_1.0	NCBI	i5k
315	*Parasteatoda tepidariorum*	Common house spider	Ptep_1.0	NCBI	i5k
**Prostigmata**					

No	Systematic Name	Common Name	Genome Version	Source	Submitter
316	*Tetranychus urticae*	Red spider mite	ASM23943v1	NCBI	Spider Mite Consortium
**Astigmata**					
317	*Dermatophagoides farinae*	House dust mite	Dfarinae1.0	NCBI	CUHK
318	*Sarcoptes scabiei type canis*	Dog mite	SarSca1.0	NCBI	Wright State University
**Oribatida**					
319	*Achipteria coleoptrata*	-	SM98876v1	NCBI	UNIL
320	*Hypochthonius rufulus*	-	ASM98884v1	NCBI	UNIL
321	*Platynothrus peltifer*	Oribatid mite	ASM98890v1	NCBI	UNIL
322	*Steganacarus magnus*	-	ASM98888v1	NCBI	UNIL
**Ixodida**					
323	*Ixodes ricinus*	Castor bean tick	ASM97304v1	NCBI	Luxembourg Institute of Health
324	*Ixodes scapularis*	Deer tick	JCVI_ISG_i3_1.0	NCBI	JCVI
325	*Rhipicephalus microplus*	Cattle tick	CCG_Rmi_1.0	NCBI	USDA-ARS
**Mesostigmata**					
326	*Metaseiulus occidentalis*	Western predatory mite	Mocc_1.0	NCBI	BCM
327	*Varroa destructor*	Honeybee mite	BRL_Vdes_1.0	NCBI	Varroa Genome Sequencing Consortium
**Scorpiones**					
328	*Centruroides exilicauda*	Baja california bark scorpion	Cexi_1.0	NCBI	i5k
329	*Mesobuthus martensii*	Chinese scorpion	M_martensii_Version_1	NCBI	Institute of Plant Physiology & Ecology, Shanghai Insitutes for Biology Sciences, CAS
**Xiphosura**					
330	*Limulus polyphemus*	Atlantic horseshoe crab	Limulus_polyphemus-2.1.2	NCBI	WashU
**Trichocephalida**					
331	*Trichinella spiralis*	Pork worm	Trichinella spiralis-3.7.1	NCBI	WashU
**Ascaridida**					
332	*Ascaris suum*	Pig roundworm	AscSuum_1.0	NCBI	BGI
**Spirurida**					
333	*Elaeophora elaphi*	Red deer nematode	EEL001	NCBI	WTSI
334	*Onchocerca volvulus*	-	OVOC001	NCBI	WTSI

No	Systematic Name	Common Name	Genome Version	Source	Submitter
**Rhabditida**					
335	*Steinernema monticolum*	-	S_monti_v1	NCBI	Caltech
336	*Panagrellus redivivus*	Microworm	Pred3	NCBI	Caltech
337	*Haemonchus contortus*	Wireworm	HCON	NCBI	WTSI
338	*Necator americanus*	Human hookworm	N_ americanus_v1	NCBI	WashU
339	*Heterorhabditis bacteriophora*	Beneficial nematode	Heterorhabditis_bacteriophora-7.0	NCBI	WashU
340	*Caenorhabditis angaria*	-	ps1010rel4	NCBI	California Institute of Technology, Division of Biology
341	*Caenorhabditis brenneri*	-	C_brenneri-6.0.1b	NCBI	Caenorhabditis brenneri Sequencing and Analysis Consortium
342	*Caenorhabditis briggsae*	-	ASM455v1	NCBI	The C.briggsae Sequencing Consortium
343	*Caenorhabditis elegans*	-	WBcel235	NCBI	C. elegans Sequencing Consortium
344	*Caenorhabditis japonica*	-	C_japonica-7.0.1	NCBI	WashU
345	*Caenorhabditis sp. 11 MAF-2010*	-	Caenorhabditis_sp11_JU1373-3.0.1	NCBI	WashU
**Priapulimorphida**					
346	*Priapulus caudatus*	Cactus worm	Priapulus_caudatus-4.0.1	NCBI	WashU
**ROTIFERA**					
**Bdelloidea**					
347	*Adineta vaga*	Rotifer	AMS_PRJEB1171_v1	NCBI	Genoscope CEA
**PLATYHELMINTHES**					
**Strigeidida**					
348	*Schistosoma curassoni*	-	S_curassoni_Dakar	NCBI	WTSI
349	*Schistosoma haematobium*	-	SchHae_1.0	NCBI	CHGC
350	*Schistosoma japonicum*	-	ASM15177v1	NCBI	CHGC
351	*Schistosoma mansoni*	-	ASM23792v2	NCBI	Schistosoma Genome Network
352	*Schistosoma margrebowiei*	-	S_margrebowiei_Zambia	NCBI	WTSI
353	*Schistosoma mattheei*	-	S_mattheei_Denwood	NCBI	WTSI

No	Systematic Name	Common Name	Genome Version	Source	Submitter
354	*Schistosoma rodhaini*	-	S_rodhaini_Burundi	NCBI	WTSI
**Opisthorchiida**					
355	*Clonorchis sinensis*	Chinese liver fluke	C_sinensis-2.0	NCBI	Department of Parasitology, Zhongshan School of Medicine, Sun Yat-sen University
**Cyclophyllidea**					
356	*Echinococcus granulosus*	Hyper tapeworm	EGRAN001	NCBI	WTSI
357	*Echinococcus multilocularis*	Fox tapeworm	EMULTI001	NCBI	WTSI
358	*Hymenolepis microstoma*	Rodent tapeworm	HMIC001	NCBI	WTSI
**Polyopisthocotylea**					
359	*Protopolystoma xenopodis*	Frog parasite	P_xenopodis_South_Africa	NCBI	WTSI
**ANNELIDA**					
**Scolecida**					
360	*Capitella teleta*	Polychaete worm	Capca1	NCBI	JGI
**Rhynchobdellida**					
361	*Helobdella robusta*	Leech	Helobdella robusta v1.0	NCBI	JGI
**MOLLUSCA**					
**Ostreoida**					
362	*Crassostrea gigas*	Pacific oyster	oyster_v9	NCBI	BGI
**Gastropoda**					
363	*Lottia gigantea*	Owl limpet	Helro1	NCBI	JGI
364	*Aplysia californica*	California sea hare	AplCal3.0	NCBI	Broad
365	*Biomphalaria glabrata*	Freshwater snail	ASM45736v1	NCBI	WashU
**CNIDARIA**					
**Actiniaria**					
366	*Nematostella vectensis*	Starlet sea anemone	ASM20922v1	NCBI	JGI
**Anthoathecata**					
367	*Hydra vulgaris*	Freshwater polyp	Hydra_RP_1.0	NCBI	JCVI
**TENTACULATA**					
**Lobata**					
368	*Mnemiopsis leidyi*	Warty comb jelly	MneLei_Aug2011	NCBI	National Human Genome Research Institute, National Institutes of Health

No	Systematic Name	Common Name	Genome Version	Source	Submitter
**PLACOZOA**					
369	*Trichoplax adhaerens*	Placozoan	v1.0	NCBI	JGI
**PORIFERA**					
**Haplosclerida**					
370	*Amphimedon queenslandica*	Sea sponge	v1.0	NCBI	JGI
**ECHINOIDEA**					
**Temnopleuroida**					
371	*Lytechinus variegatus*	Green sea urchin	Lvar_0.4	NCBI	BCM
**Echinoida**					
372	*Strongylocentrotus purpuratus*	Purple sea urchin	Spur_3.1	NCBI	BCM
**ASTEROIDEA**					
**Valvatida**					
373	*Patiria miniata*	Bat star	Pmin_1.0	NCBI	Sea Urchin Genome Sequencing Consortium
**ENTEROPNEUSTA**					
374	*Saccoglossus kowalevskii*	Acorn worm	Skow_1.1	NCBI	BCM
**TUNICATA**					
**Enterogona**					
375	*Ciona intestinalis*	Sea squirt	KH	NCBI	Organization: Department of Zoology, Graduate School
376	*Ciona savignyi*	-	ASM14926v1	NCBI	Broad
**Pleurogona**					
377	*Botryllus schlosseri*	Star ascidian	356a-chromosome-assembly	NCBI	Stanford University
**Copelata**					
378	*Oikopleura dioica*	Larvaceans	ASM20953v1	NCBI	Genoscope CEA
**LEPTOCARDII**					
**Amphioxiformes**					
379	*Branchiostoma floridae*	Lancelet	Version 2	NCBI	JGI
**CEPHALASPIDOMORPHI**					
**Petromyzontiformes**					
380	*Lethenteron camtschaticum*	Arctic lamprey	LetJap1.0	NCBI	IMCB
381	*Petromyzon marinus*	Sea lamprey	petMar2	UCSC	WashU
**SARCOPTERYGII**					
**Coelacanthiformes**					
382	*Latimeria chalumnae*	Coelacanth	latCha1	UCSC	Broad
**FUNGI**					

No	Systematic Name	Common Name	Genome Version	Source	Submitter
**AGARICOMYCETES**					
**Agaricales**					
383	*Agaricus bisporus var. bisporus*	Edible basidiomycete mushroom	ASM30057v2	NCBI	JGI-PSF
384	*Agaricus bisporus var. burnettii*	-	Agabi_varbur_1	NCBI	JGI
385	*Schizophyllum commune*	Mushroom	v1.0	NCBI	JGI-PGF
386	*Coprinopsis cinerea*	Small mushroom	CC3	NCBI	Broad
387	*Moniliophthora roreri*	Frosty pod rot	ASM146670v1	NCBI	Purdue University
388	*Laccaria bicolor*	Tan-colored mushroom	V1.0	NCBI	Laccaria Genome Consortium
**Auriculariales**					
389	*Auricularia subglabra*	Jelly fungi	Auricularia subglabra SS-5 V1.0	NCBI	JGI
**Boletales**					
390	*Coniophora puteana*	Wet rot fungus	Conpu1	NCBI	DOE JGI
391	*Serpula lacrymans*	Dry rot fungus	v1.0	NCBI	JGI-PGF
**Corticiales**					
392	*Punctularia strigosozonata*	Steroid fungi	Punctularia strigosozonata v1.0	NCBI	JGI
**Gloeophyllales**					
393	*Gloeophyllum trabeum*	Brown-rot fungi	Glotr1_1	NCBI	JGI
**Hymenochaetales**					
394	*Fomitiporia mediterranea*	-	Fomme1	NCBI	JGI
**Polyporales**					
395	*Dichomitus squalens*	Poroid crust fungi	Dichomitus squalens v1.0	NCBI	JGI
396	*Fibroporia radiculosa*	-	ASM31352v1	NCBI	Forest Products, Mississippi State University
397	*Phanerochaete carnosa*	Crust fungus	Phanerochaete carnosa HHB-10118-Sp v1.0	NCBI	DOE JGI
398	*Postia placenta*	Brown rot fungus	PosplRSB12_1	NCBI	DOE JGI
399	*Trametes versicolor*	Polypore mushroom	Trametes versicolor v1.0	NCBI	JGI
**Russulales**					
400	*Heterobasidion irregulare*	Tree root rotting pathogenic fungus	Heterobasidion irregulare v2.0	NCBI	JGI
401	*Stereum hirsutum*	False turkey tail	Stehi1	NCBI	JGI
**CHYTRIDIOMYCETES**					
**Spizellomycetales**					

No	Systematic Name	Common Name	Genome Version	Source	Submitter
402	*Spizellomyces punctatus*	Chytrid fungus	S_punctatus_V1	NCBI	Broad
**Rhizophydiales**					
403	*Batrachochytrium dendrobatidis*	Amphibian chytrid fungus	v1.0	NCBI	JGI-PGF
**DOTHIDEOMYCETES**					
**Botryosphaeriales**					
404	*Diplodia corticola*	-	ASM188384v1	NCBI	University of Aveiro
405	*Neofusicoccum parvum*	-	UCRNP2V3	NCBI	UC Davis
**Capnodiales**					
406	*Baudoinia panamericana*	-	Bauco1	NCBI	JGI
407	*Cercospora beticola*	Fungal plant pathogen	CB0940_V2	NCBI	VIB
408	*Pseudocercospora fijiensis*	Black leaf streak fungus	Mycfi2	NCBI	DOE JGI
409	*Sphaerulina musiva*	-	Septoria musiva SO2202 v1.0	NCBI	JGI
410	*Zymoseptoria tritici*	Filamentous fungus	MYCGR v2.0	NCBI	DOE JGI
**Dothideales**					
411	*Aureobasidium namibiae*	Black yeast-like fungus	Aureobasidium pullulans var. namibiae CBS 147.97 v1.0	NCBI	DOE JGI
412	*Aureobasidium subglaciale*	-	Aureobasidium pullulans var. subglaciale EXF-2481 v1.0	NCBI	JGI
**Dothideomycetes incertae sedis**					
413	*Coniosporium apollinis*	-	Coni_apol_CBS100218 _V1	NCBI	Broad
**Pleosporales**					
414	*Paraphaeosphaeria sporulosa*	-	Parsp1	NCBI	DOE JGI
415	*Parastagonospora nodorum*	-	ASM14691v2	NCBI	Broad
416	*Leptosphaeria maculans*	Blackleg disease fungus	ASM23037v1	NCBI	Genoscope
417	*Alternaria alternata*	-	Altal1	NCBI	DOE JGI
418	*Bipolaris maydis*	Stalk rot fungus	CocheC4_1	NCBI	JGI
419	*Bipolaris oryzae*	-	Cochliobolus miyabeanus v1.0	NCBI	JGI
420	*Bipolaris sorokiniana*	-	Cocsa1	NCBI	JGI
421	*Bipolaris victoriae*	-	Cochliobolus victoriae v1.0	NCBI	JGI

No	Systematic Name	Common Name	Genome Version	Source	Submitter
422	*Bipolaris zeicola*	-	Cochliobolus carbonum v1.0	NCBI	JGI
423	*Pyrenophora teres*	Necrotrophic fungus	PTT_W1-1	NCBI	CURTIN UNIVERSITY
424	*Pyrenophora tritici-repentis*	-	ASM14998v1	NCBI	Broad
425	*Setosphaeria turcica*	-	Setospaeria trucica Et28A v1.0	NCBI	JGI
**Venturiales**					
426	*Verruconis gallopava*	-	O_gall_CBS43764	NCBI	Broad
**EUROTIOMYCETES**					
**Chaetothyriales**					
427	*Cyphellophora europaea*	-	Phia_euro_CBS_101466_V1	NCBI	Broad
428	*Capronia coronata*	Black yeast	Capr_coro_CB_617_96_V1	NCBI	Broad
429	*Capronia epimyces*	-	Capr_epim_CBS_606_96_V1	NCBI	Broad
430	*Cladophialophora bantiana*	Dematiaceous fungus	Clad_bant_CBS_173_52_V1	NCBI	Broad
431	*Cladophialophora carrionii*	Melanized fungus	Clad_carr_CBS_160_54_V1	NCBI	Broad
432	*Cladophialophora immunda*	-	Clad_immu_CBS83496_V1	NCBI	Broad
433	*Cladophialophora psammophila*	-	Clad_psam_CBS_110553_V1	NCBI	Broad
434	*Cladophialophora yegresii*	-	Clad_yegr_CBS_114405_V1	NCBI	Broad
435	*Exophiala aquamarina*	-	Exop_aqua_CBS_119918_V1	NCBI	Broad
436	*Exophiala dermatitidis*	-	Exop_derm_V1	NCBI	Broad
437	*Exophiala mesophila*	-	Exop_meso_CBS40295_V1	NCBI	Broad
438	*Exophiala oligosperma*	-	Exop_olig_CBS72588_V1	NCBI	Broad
439	*Exophiala spinifera*	-	Exop_spin_CBS89968_V1	NCBI	Broad
440	*Exophiala xenobiotica*	-	Exop_xeno_CBS118157_V1	NCBI	Broad
441	*Fonsecaea erecta*	-	ASM165198v1	NCBI	Federal University of Parana

No	Systematic Name	Common Name	Genome Version	Source	Submitter
442	*Fonsecaea monophora*	-	ASM164247v1	NCBI	Federal University of Parana
443	*Fonsecaea multimorphosa*	-	Fons_mult_CBS_102226_V1	NCBI	Broad
444	*Fonsecaea nubica*	-	ASM164696v1	NCBI	Federal University of Parana
445	*Fonsecaea pedrosoi*	-	Fons_pedr_CBS_271_37_V1	NCBI	Submitter
446	*Phialophora attae*	Black yeast	ASM129925v1	NCBI	CBS-KNAW Fungal Biodiversity Centre
447	*Rhinocladiella mackenziei*	-	Rhin_mack_CBS_650_93_V1	NCBI	Broad
**Eurotiales**					
448	*Aspergillus aculeatus*	-	Aspac1	NCBI	JGI
449	*Aspergillus bombycis*	-	ASM179269v1	NCBI	USDA-ARS-SRRC
450	*Aspergillus clavatus*	-	ASM271v1	NCBI	J. Craig Venter Institute
451	*Aspergillus fischeri*	-	ASM14964v1	NCBI	TIGR
452	*Aspergillus flavus*	-	JCVI-afl1-v2.0	NCBI	J. Craig Venter Institute
453	*Aspergillus fumigatus*	-	ASM265v1	NCBI	J. Craig Venter Institute
454	*Aspergillus glaucus*	-	Aspgl1	NCBI	DOE JGI
455	*Aspergillus nidulans*	-	ASM14920v2	NCBI	Broad
456	*Aspergillus niger*	-	ASM285v2	NCBI	DSM, The Netherlands
457	*Aspergillus nomius*	-	ASM120477v1	NCBI	USDA-ARS-SRRC
458	*Aspergillus oryzae*	-	ASM18445v3	NCBI	NITE
459	*Aspergillus terreus*	Mold fungus	ASM14961v1	NCBI	Broad
460	*Penicilliopsis zonata*	-	Aspzo1	NCBI	DOE JGI
461	*Penicillium arizonense*	-	ASM177332v1	NCBI	Chalmers University of Technology
462	*Penicillium chrysogenum*	-	ASM71027v1	NCBI	Ruhr-Univeristaet Bochum
463	*Penicillium digitatum*	-	PdigPd1_v1	NCBI	CRG
464	*Penicillium expansum*	Psychrophilic blue mold	ASM76974v1	NCBI	CRG
465	*Penicillium marneffei*	-	JCVI-PMFA1-2.0	NCBI	J. Craig Venter Institute

No	Systematic Name	Common Name	Genome Version	Source	Submitter
466	*Rasamsonia emersonii*	-	ASM96859v1	NCBI	DSM Bio-based Products & Services B.V.
467	*Talaromyces atroroseus*	-	ASM190759v1	NCBI	Technical University of Denmark
468	*Talaromyces stipitatus*	-	JCVI-TSTA1-3.0	NCBI	J. Craig Venter Institute
**Onygenales**					
469	*Ajellomyces dermatitidis*	-	BD_ER3_V1	NCBI	Broad
470	*Arthroderma otae*	-	ASM15114v1	NCBI	Broad
471	*Coccidioides immitis*	-	ASM14933v2	NCBI	Broad
472	*Coccidioides posadasii*	-	JCVI-cpa1-1.0	NCBI	TIGR
473	*Histoplasma capsulatum*	-	ASM14958v1	NCBI	Broad
474	*Nannizzia gypsea*	-	MS_CBS118893	NCBI	Broad
475	*Paracoccidioides brasiliensis*	Dimorphic fungus	Paracocci_br_Pb18_ V2	NCBI	Broad
476	*Paracoccidioides lutzii*	-	Paracocci_br_Pb01_ V2	NCBI	Broad
477	*Trichophyton benhamiae*	-	ASM15112v2	NCBI	Arthroderma Genome Sequencing Consortium
478	*Trichophyton rubrum*	-	ASM15142v1	NCBI	Broad
479	*Trichophyton verrucosum*	-	ASM15150v1	NCBI	Arthroderma Genome Sequencing Consortium
480	*Uncinocarpus reesii*	-	ASM351v2	NCBI	Broad
**Verrucariales**					
481	*Endocarpon pusillum*	-	EPUS	NCBI	Chinese Academy of Sciences

**EXOBASIDIOMYCETES**					
**Georgefischeriales**					
482	*Tilletiaria anomala*	-	Tilletiaria anomala UBC 951 v1.0	NCBI	DOE JGI

**LEOTIOMYCETES**					
**Helotiales**					
483	*Botryotinia fuckeliana*	Gray mold fungus	ASM14353v4	NCBI	Syngenta Biotechnology, Inc.
484	*Glarea lozoyensis*	-	GLAREA	NCBI	Chinese Academy of Sciences

No	Systematic Name	Common Name	Genome Version	Source	Submitter
485	*Marssonina brunnea*	-	ASM29877v1	NCBI	Nanjing Forestry University
486	*Phialocephala scopiformis*	-	Phisc1	NCBI	DOE JGI
487	*Sclerotinia sclerotiorum*	White mold fungus	ASM14694v2	NCBI	Broad
**Leotiomycetes incertae sedis**					
488	*Pseudogymnoascus destructans*	Cold-loving fungus	ASM164126v1	NCBI	US Forest Service
489	*Pseudogymnoascus verrucosus*	-	ASM166265v1	NCBI	US Forest Service
**MALASSEZIOMYCETES**					
**Malasseziales**					
490	*Malassezia globosa*	-	ASM18169v1	NCBI	The Procter & Gamble Company
491	*Malassezia pachydermatis*	-	MalaPachy	NCBI	University of Utrecht
492	*Malassezia sympodialis*	-	ASM34930v2	NCBI	SciLifeLab
**MICROBOTRYOMYCETES**					
**Sporidiobolales**					
493	*Rhodotorula graminis*	Pigmented yeast	Rhoba1_1	NCBI	DOE JGI
494	*Rhodotorula toruloides*	-	RHOziaDV1.0	NCBI	Dalian Institute of Chemical Physics
**MIXIOMYCETES**					
**Mixiales**					
495	*Mixia osmundae*	-	Mixia osmundae v1.0	NCBI	DOE JGI
**ORBILIOMYCETES**					
**Orbiliales**					
496	*Arthrobotrys oligospora*	-	AOL24927 1.0	NCBI	P. R. China
497	*Dactylellina haptotyla*	-	MHA_v2	NCBI	Lund University
**PEZIZOMYCETES**					
**Pezizales**					
498	*Tuber melanosporum*	Black truffle	ASM15164v1	NCBI	The French-Italian Tuber Genome Consortium
**PNEUMOCYSTIDOMYCETES**					
**Pneumocystidales**					
499	*Pneumocystis carinii*	Yeast-like fungus	Pneu_cari_B80_ V3	NCBI	Broad
500	*Pneumocystis jirovecii*	-	Pneu_jiro_RU7_ V2	NCBI	Broad
501	*Pneumocystis murina*	-	Pneumo_murina_B123_ V4	NCBI	Broad
**PUCCINIOMYCETES**					

No	Systematic Name	Common Name	Genome Version	Source	Submitter
**Pucciniales**					
502	*Melampsora larici-populina*	Poplar leaf rust fungus	v1.0	NCBI	JGI-PGF
503	*Puccinia graminis*	Wheat stem rust fungus	ASM14992v1	NCBI	Broad
**SACCHAROMYCETES**					
**Saccharomycetales**					
504	*Candida albicans*	Yeast	ASM18296v3	NCBI	Stanford University
505	*Candida auris*	-	ASM118947v1	NCBI	Indian Institute of Science
506	*Candida dubliniensis*	-	ASM2694v1	NCBI	Wellcome
507	*Candida glabrata*	-	ASM254v2	NCBI	Genolevures Consortium
508	*Candida orthopsilosis*	-	ASM31587v1	NCBI	Trinity College Dublin
509	*Candida tanzawaensis*	-	Canta1	NCBI	Submitter
510	*Candida tenuis*	-	Candida tenuis v1.0	NCBI	DOE JGI
511	*Candida tropicalis*	-	ASM633v2	NCBI	Broad
512	*Lodderomyces elongisporus*	-	ASM14968v1	NCBI	Broad
513	*Ascoidea rubescens*	-	Ascru1	NCBI	DOE JGI
514	*Babjeviella inositovora*	-	Babin1	NCBI	DOE JGI
515	*Clavispora lusitaniae*	-	ASM383v1	NCBI	Broad
516	*Cyberlindnera jadinii*	-	Cybja1	NCBI	DOE JGI
517	*Debaryomyces fabryi*	-	debFab1.0	NCBI	University of Natural Resources and Life Sciences
518	*Debaryomyces hansenii*	-	ASM644v2	NCBI	Genolevures Consortium
519	*Eremothecium cymbalariae*	-	ASM23536v1	NCBI	Carlsberg Laboratory
520	*Eremothecium gossypii*	-	ASM9102v4	NCBI	Biozentrum
521	*Eremothecium sinecaudum*	-	ASM154855v1	NCBI	Duke University
522	*Hyphopichia burtonii*	-	Hypbu1	NCBI	DOE JGI
523	*Kazachstania africana*	-	Ka_CBS2517	NCBI	Wolfe Laboratory
524	*Kazachstania naganishii*	-	ASM34898v1	NCBI	Wolfe Laboratory
525	*Kluyveromyces lactis*	-	ASM251v1	NCBI	Genolevures Consortium
526	*Kluyveromyces marxianus*	-	Kmar_1.0	NCBI	Yamaguchi University
527	*Komagataella phaffii*	-	ASM2700v1	NCBI	VIB
528	*Kuraishia capsulata*	-	AUH_PRJEB4427_v1	NCBI	Genoscope CEA

No	Systematic Name	Common Name	Genome Version	Source	Submitter
529	*Lachancea lanzarotensis*	-	LALA0	NCBI	INRA UMR 1319
530	*Lachancea thermotolerans*	-	ASM14280v1	NCBI	Genolevures Consortium
531	*Metschnikowia bicuspidata*	-	Metbi1	NCBI	DOE JGI
532	*Meyerozyma guilliermondii*	-	ASM14942v1	NCBI	Broad
533	*Naumovozyma castellii*	-	ASM23734v1	NCBI	Trinity College Dublin
534	*Naumovozyma dairenensis*	-	ASM22711v2	NCBI	Trinity College Dublin
535	*Ogataea parapolymorpha*	-	Hansenula_2	NCBI	Centre
536	*Ogataea polymorpha*	-	Hanpo2	NCBI	JGI-PGF
537	*Saccharomyces cerevisiae*	-	R64	NCBI	Saccharomyces Genome Database
538	*Saccharomyces eubayanus*	-	SEUB3.0	NCBI	University of Wisconsin-Madison
539	*Spathaspora passalidarum*	-	Spathaspora passalidarum v2.0	NCBI	DOE JGI
540	*Sugiyamaella lignohabitans*	-	ASM164002v2	NCBI	University of Natural Resources and Life Sciences
541	*Tetrapisispora blattae*	-	ASM31591v1	NCBI	Wolfe Laboratory
542	*Tetrapisispora phaffii*	-	ASM23690v1	NCBI	Wolfe Laboratory
543	*Torulaspora delbrueckii*	-	ASM24337v1	NCBI	Wolfe Laboratory
544	*Vanderwaltozyma polyspora*	-	ASM15003v1	NCBI	Trinity College Dublin
545	*Yarrowia lipolytica*	-	ASM252v1	NCBI	Genolevures Consortium
546	*Zygosaccharomyces rouxii*	-	ASM2636v1	NCBI	Genolevures Consortium
547	*Wickerhamomyces anomalus*	-	Wican1	NCBI	JGI-PSF
548	*Wickerhamomyces ciferrii*	-	ASM31348v1	NCBI	Center for Biotechnology
549	*Scheffersomyces stipitis*	-	ASM20916v1	NCBI	DOE JGI
550	*Pichia kudriavzevii*	-	ASM198332v1	NCBI	WUR
551	*Pichia membranifaciens*	-	Picme2	NCBI	JGI-PSF

No	Systematic Name	Common Name	Genome Version	Source	Submitter
**SCHIZOSACCHAROMYCETES**					
**Schizosaccharomycetales**					
552	*Schizosaccharomyces cryophilus*	-	SCY4	NCBI	Broad
553	*Schizosaccharomyces japonicus*	-	SJ5	NCBI	Broad
554	*Schizosaccharomyces octosporus*	-	SO6	NCBI	Broad
555	*Schizosaccharomyces pombe*	-	ASM294v2	NCBI	EUPOM
**SORDARIOMYCETES**					
**Hypocreales**					
556	*Metarhizium acridum*	-	MetAcr_May2010	NCBI	Metarhizium genome sequencing Consortium
557	*Metarhizium brunneum*	-	MBR_1.0	NCBI	Shanghai Insititutes for Biological Sciences
558	*Metarhizium majus*	-	MAJ_1.0	NCBI	Shanghai Insititutes for Biological Sciences
559	*Metarhizium robertsii*	-	MAA 2.0	NCBI	Metarhizium genome sequencing Consortium
560	*Pochonia chlamydosporia*	-	ASM165323v2	NCBI	The Institute of Vegetables and Flowers CAAS
561	*Beauveria bassiana*	-	ASM28067v1	NCBI	Zhejiang University, China
562	*Cordyceps militaris*	-	CmilitarisCM01_v01	NCBI	SIBS
563	*Isaria fumosorosea*	-	ISF 1.0	NCBI	Shanghai Institutes for Biological Sciences
564	*Trichoderma atroviride*	-	TRIAT v2.0	NCBI	DOE JGI
565	*Trichoderma gamsii*	-	TGAM01v2	NCBI	University of Pisa
566	*Trichoderma reesei*	-	v2.0	NCBI	DOE JGI
567	*Trichoderma virens*	-	TRIVI v2.0	NCBI	DOE JGI
568	*Fusarium fujikuroi*	-	Fusarium_fujikuroi_IMI58289_V2	NCBI	HMGU-IBIS

No	Systematic Name	Common Name	Genome Version	Source	Submitter
569	*Fusarium graminearum*	-	ASM24013v3	NCBI	International Gibberella zeae Genomics Consortium
570	*Fusarium oxysporum*	-	ASM14995v2	NCBI	Broad
571	*Fusarium pseudograminearum*	Crown rot fungus	FP7	NCBI	CSIRO
572	*Fusarium verticillioides*	-	ASM14955v1	NCBI	Syngenta Biotechnology, Inc.
573	*Nectria haematococca*	-	v2.0	NCBI	DOE JGI
574	*Purpureocillium lilacinum*	-	ASM 165326v1	NCBI	The Institute of Vegetables and Flowers CAAS
**Glomerellales**					
575	*Colletotrichum fioriniae*	-	ASM293045v1	NCBI	VIB
576	*Colletotrichum gloeosporioides*	-	ASM290110v1	NCBI	Yeungnam University
577	*Colletotrichum graminicola*	-	C_graminicola_M1_001_V1	NCBI	Broad
578	*Colletotrichum higginsianum*	-	ASM167251v1	NCBI	GATC Biotech AG
579	*Colletotrichum orchidophilum*	-	CORC01	NCBI	University of Western Brittany
580	*Verticillium alfalfae*	Verticillium wilt fungus	ASM15082v1	NCBI	Broad
581	*Verticillium dahliae*	-	ASM15067v2	NCBI	Broad
**Magnaporthales**					
582	*Magnaporthe oryzae*	Rice blast fungus	MG8	NCBI	International Rice Blast Genome Consortium
583	*Gaeumannomyces tritici*	-	Gae_graminis_V2	NCBI	Broad
**Microascales**					
584	*Scedosporium apiospermum*	Environmental mold	ScApio1.0	NCBI	LUNAM - Angers University
**Ophiostomatales**					
585	*Grosmannia clavigera*	-	Sanger-454-IlluminaPA_2.0	NCBI	The Tria Project
586	*Sporothrix schenckii*	Dimorphic fungus	S_schenckii_v1	NCBI	LNCC
**Sordariales**					
587	*Chaetomium globosum*	-	ASM14336v1	NCBI	The Genome Assembly Team

No	Systematic Name	Common Name	Genome Version	Source	Submitter
588	*Chaetomium thermophilum*	-	CTHT_3.0	NCBI	EMBL
589	*Neurospora crassa*	-	NC12	NCBI	Broad
590	*Neurospora tetrasperma*	-	v2.0	NCBI	JGI-PGF
591	*Podospora anserina*	-	ASM22654v1	NCBI	Genoscope
592	*Sordaria macrospora*	-	ASM18280v2	NCBI	Ruhr University Bochum
593	*Thermothelomyces thermophila*	-	ASM22609v1	NCBI	DOE JGI
594	*Thielavia terrestris*	-	ASM22611v1	NCBI	DOE JGI
**Togniniales**					
595	*Phaeoacremonium minimum*	-	UCRPA7V03	NCBI	UC Davis
**Xylariales**					
596	*Eutypa lata*	-	UCREL1V03	NCBI	UC Davis
597	*Pestalotiopsis fici*	Endophytic fungus	PFICI	NCBI	Chinese Academy of Sciences
**TREMELLOMYCETES**					
**Tremellales**					
598	*Cryptococcus amylolentus*	-	Cryp_amyl_CBS6039 _V3	NCBI	Broad
599	*Cryptococcus gattii*	-	ASM18594v1	NCBI	Canada Michael Smith Genome Sciences Centre
600	*Cryptococcus neoformans var. grubii*	Encapsulated yeast	CNA3	NCBI	Broad
601	*Cryptococcus neoformans var. neoformans JEC21*	-	ASM9104v1	NCBI	TIGR
602	*Cryptococcus neoformans var. neoformans B-3501A*	-	ASM14938v1	NCBI	Stanford University
603	*Kockovaella imperatae*	-	Kocim1	NCBI	DOE JGI
604	*Kwoniella bestiolae*	-	Cryp_best_CBS10118 _V1	NCBI	Broad
605	*Kwoniella dejecticola*	-	Cryp_deje_CBS10117 _V1	NCBI	Broad
606	*Kwoniella mangroviensis*	-	Kwon_mang_CBS8507 _V2	NCBI	Broad
607	*Kwoniella pini*	-	Cryp_pinu_CBS10737 _V1	NCBI	Broad
608	*Tremella mesenterica*	-	Treme1	NCBI	DOE JGI
609	*Tsuchiyaea wingfieldii*	-	Tsuc_wing_CBS7118 _V1	NCBI	Broad
**Trichosporonales**					

No	Systematic Name	Common Name	Genome Version	Source	Submitter
610	*Cutaneotrichosporon oleaginosus*	-	Triol1	NCBI	DOE JGI
611	*Trichosporon asahii*	-	Trichosporon asahii 1	NCBI	Beijing Military Command General Hospital of PLA
**USTILAGINOMYCETES**					
**Ustilaginales**					
612	*Anthracocystis flocculosa*	-	Pflocc_1.0	NCBI	Universite Laval
613	*Kalmanozyma brasiliensis*	-	PSEUBRA1	NCBI	Laboratorio Nacional de Ciencia e Tecnologia do Bioetanol
614	*Moesziomyces antarcticus*	-	ASM74776v1	NCBI	National Institute of Advanced Industrial Science and Technology
615	*Pseudozyma hubeiensis*	-	ASM40351v1	NCBI	Kitami Institute of Technology
616	*Ustilago maydis*	Common smut fungus	Umaydis521_2.0	NCBI	Broad
**WALLEMIOMYCETES**					
**Wallemiales**					
617	*Wallemia ichthyophaga*	-	Wallemia ichthyophaga version 1.0	NCBI	BGI
618	*Wallemia mellicola*	-	Wallemia sebi v1.0	NCBI	JGI
**XYLONOMYCETES**					
**Xylonales**					
619	*Xylona heveae*	Rubber tree fungus	Xylona heveae TC161 v1.0	NCBI	DOE JGI
**TAPHRINOMYCOTINA INCERTAE SEDIS**					
**Taphrinomycotina**					
620	*Saitoella complicata*	-	Saico1	NCBI	JGI-PSF
**FUNGI INCERTAE SEDIS**					
**Mortierellales**					
621	*Lobosporangium transversale*	-	Lobtra1	NCBI	DOE JGI
**Mucorales**					
622	*Phycomyces blakesleeanus*	-	Phybl2	NCBI	JGI-PSF
623	*Rhizopus microsporus*	Soil fungus	Rhimi1_1	NCBI	DOE JGI
**Microsporidia**					

No	Systematic Name	Common Name	Genome Version	Source	Submitter
624	*Encephalitozoon cuniculi*	Rabbit fungus	ASM9122v1	NCBI	Genoscope
625	*Encephalitozoon hellem*	-	ASM27781v3	NCBI	University of British Columbia
626	*Encephalitozoon intestinalis*	-	ASM14646v1	NCBI	University of British Columbia
627	*Encephalitozoon romaleae*	-	ASM28003v2	NCBI	University of Ottawa
628	*Enterocytozoon bieneusi*	-	ASM20948v1	NCBI	Tufts Cummings School of Veterinary Medicine
629	*Mitosporidium daphniae*	-	UGP1.0	NCBI	Illinois Institute of Technology
630	*Nematocida parisii*	Nematode-killer from Paris	Nema_parisii_ERTm1 _V3	NCBI	Broad
631	*Nosema ceranae*	Microsporidian	ASM98816v1	NCBI	University of Ottawa
632	*Ordospora colligata*	-	ASM80326v1	NCBI	University of British Columbia
633	*Vavraia culicis*	Spore-forming parasite	Vavr_culi_ floridensis_V1	NCBI	Broad
634	*Vittaforma corneae*	-	Vitt_corn_V1	NCBI	Broad
**VIRIDIPLANTAE**					
**Mamiellales**					
635	*Micromonas pusilla CCMP1545*	-	Micromonas pusilla CCMP1545 v2.0	NCBI	Micromonas Genome Consortium
636	*Micromonas sp. RCC299*	-	ASM9098v2	NCBI	Micromonas Genome Consortium
637	*Ostreococcus lucimarinus CCE9901*	-	ASM9206v1	NCBI	JGI
638	*Ostreococcus tauri*	-	version 050606	NCBI	Laboratoire Arago, France
**Chlamydomonadales**					
639	*Chlamydomonas reinhardtii*	-	v3.0	NCBI	JGI
**Volvocales**					
640	*Volvox carteri f. nagariensis*	-	v1.0	NCBI	JGI
**Chlorellales**					
641	*Chlorella variabilis*	-	v 1.0	NCBI	JGI

No	Systematic Name	Common Name	Genome Version	Source	Submitter
642	*Auxenochlorella protothecoides*	Green microalga	ASM73321v1	NCBI	BGI
643	*Helicosporidium sp. ATCC 50920*	-	Helico_v1.0	NCBI	University of British Columbia
**Chlorococcales**					
644	*Coccomyxa subellipsoidea C-169*	-	Coccomyxa subellipsoidae v2.0	NCBI	JGI
**Klebsormidiales**					
645	*Klebsormidium flaccidum*	-	ASM70883v1	NCBI	TokyoTech
**Funariales**					
646	*Physcomitrella patens*	-	V1.1	NCBI	Moss Genome Consortium
**Selaginellales**					
647	*Selaginella moellendorffii*	Spikemoss	v1.0	NCBI	Selaginella Consortium
**Pinales**					
648	*Pinus taeda*	Loblolly pine	PtaedaFosmidLib.0.8	NCBI	UC Davis
**Amborellales**					
649	*Amborella trichopoda*	Amborellaceae	AMTR1.0	NCBI	Amborella Genome Sequencing Project
**Alismatales**					
650	*Spirodela polyrhiza*	Greater duckweed	Spirodela_polyrhiza_v01	NCBI	Waksman Institute
**Arecales**					
651	*Phoenix dactylifera*	Date palm	DPV01	NCBI	Joint Center for Genomics Research
652	*Elaeis oleifera*	American oil palm	EO8	NCBI	Orion Genomics
**Zingiberales**					
653	*Ensete ventricosum*	Ethiopian banana	v1.1	NCBI	University of Exeter
654	*Musa acuminata subsp. malaccensis*	-	ASM31385v1	NCBI	Geno-scope/IG/CEA
**Poales**					
655	*Sorghum bicolor*	Sorghum	Sorbi1	NCBI	Sorghum Consortium
656	*Zea mays*	Maize	B73 RefGen_v3	NCBI	Maize Genome Sequencing Project
657	*Setaria italica*	Foxtail millet	Setaria V1	NCBI	JGI

No	Systematic Name	Common Name	Genome Version	Source	Submitter
658	*Brachypodium distachyon*	Purple false brome	v1.0	NCBI	JGI and The International Brachypodium Initiative
659	*Leersia perrieri*	-	Lperr_V1.4	NCBI	Arizona Genomics Institute
660	*Oryza barthii*	Rice	O.barthii_v1.3	NCBI	Oryza Chr3 Short Arm Comparative Sequencing Project
661	*Oryza brachyantha*	-	Oryza_brachyantha. v1.4b	NCBI	The Institute of Genetics and Developmental Biology
662	*Oryza glumipatula*	-	Oryza_glumaepatula_ v1.5	NCBI	OMAP
663	*Oryza longistaminata*	-	O_longistaminata_v1.0	NCBI	BGI
664	*Oryza meridionalis*	-	Oryza_meridionalis_ v1.3	NCBI	OMAP
665	*Oryza nivara*	-	Oryza_nivara_v1.0	NCBI	OMAP
666	*Oryza punctata*	-	Oryza_punctata_v1.2	NCBI	OMAP
667	*Oryza sativa Japonica Group*	-	Build 4.0	NCBI	International Rice Genome Sequencing Project
668	*Zizania latifolia*	Manchurian wild rice	Zizania_latifolia_v01	NCBI	Zhejiang University
669	*Aegilops tauschii*	Tausch's goatgrass	ASM34733v1	NCBI	BGI
670	*Triticum urartu*	Wild einkorn	ASM34745v1	NCBI	BGI
**Proteales**					
671	*Nelumbo nucifera*	Sacred lotus	Chinese Lotus 1.1	NCBI	UIUC
**Fabales**					
672	*Lupinus angustifolius*	Blue lupin	Lupin genome scaffold	NCBI	Department of Agriculture and Food, WA government, Australia
673	*Phaseolus vulgaris*	Common bean	PhaVulg1_0	NCBI	JGI
674	*Cajanus cajan*	Pigeon pea	Cajanus_cajan_ Asha_ver1.0	NCBI	AKI-PGI
675	*Vigna angularis var. angularis*	Adzuki bean	Vigna angularis	NCBI	SNU
676	*Vigna radiata var. radiata*	-	Vradiata_ver6	NCBI	SNU
677	*Glycine max*	Soybean	V1.1	NCBI	JGI

No	Systematic Name	Common Name	Genome Version	Source	Submitter
678	*Glycine soja*	-	W05v1.0	NCBI	CUHK
679	*Cicer arietinum*	Chickpea	ASM33114v1	NCBI	BGI
680	*Medicago truncatula*	Barrel clover	MedtrA17_3.5	NCBI	International Medicago Genome Annotation Group
681	*Trifolium pratense*	Red clover	Tp1.0	NCBI	Masaryk University
682	*Lotus japonicus*	Birdsfoot trefoil	ASM18111v1	NCBI	KDRI
**Rosales**					
683	*Malus x domestica*	Apple	MalDomGD1.0	NCBI	IASMA research center
684	*Pyrus x bretschneideri*	Chinese white pear	Pbr_v1.0	NCBI	Nanjing Agricultural University
685	*Prunus mume*	Japanese apricot	P.mume_V1.0	NCBI	BGI
686	*Prunus persica*	Peach	Prupe1_0	NCBI	JGI
687	*Fragaria iinumae*	Japanese strawberry	FII_r1.1	NCBI	KDRI
688	*Fragaria nubicola*	Himalayan strawberry	FNU_r1.1	NCBI	KDRI
689	*Fragaria orientalis*	Wild asian strawberry	FOR_r1.1	NCBI	KDRI
690	*Fragaria vesca subsp. vesca*	Woodland strawberry	FraVesHawaii_1.0	NCBI	Virginia Bioinformatics Institute
691	*Fragaria x ananassa*	Garden strawberry	FANhybrid_r1.2	NCBI	KDRI
692	*Morus notabilis*	Mulberry tree	ASM41409v2	NCBI	BGI
693	*Cannabis sativa*	Cannabis	canSat3	NCBI	University of Toronto
**Fagales**					
694	*Castanea mollissima*	Chinese chestnut	ASM76360v1	NCBI	Clemson University Genomics Institute
695	*Betula nana*	Dwarf birch	ASM32700v1	NCBI	QMUL
**Cucurbitales**					
696	*Cucumis melo*	Muskmelon	ASM31304v1	NCBI	Genetica Molecular, Centre de Recerca en Agrigenomica
697	*Cucumis sativus*	Cucumber	CucSat_1.0	NCBI	The Cucumber Genome Initiative

No	Systematic Name	Common Name	Genome Version	Source	Submitter
698	*Citrullus lanatus*	Watermelon	CiLa_1.0	NCBI	National Engineering Research Center for Vegetables, Beijing Academy of Agriculture and Forestry Sciences
699	*Lagenaria siceraria*	Calabash	Bottle_gourd	NCBI	Institute of Vegetables, Zhejiang Academy of Agricultural Sciences
**Malpighiales**					
700	*Populus euphratica*	Desert poplar	PopEup_1.0	NCBI	Lanzhou University
701	*Populus trichocarpa*	Black cottonwood	Poptr2_0	NCBI	JGI
702	*Jatropha curcas*	Physic nut	JatCur_1.0	NCBI	Chinese Academy of Sciences
703	*Manihot esculenta subsp. flabellifolia*	-	MW_v2d	NCBI	The Cassava Genome Consortium
704	*Ricinus communis*	Castor oil plant	JCVI_RCG_1.1	NCBI	JCVI
705	*Linum usitatissimum*	Flax	LinUsi_v1.1	NCBI	TUFGEN
**Myrtales**					
706	*Eucalyptus camaldulensis*	River red gum	EUC_r1.0	NCBI	KDRI
707	*Eucalyptus grandis*	Rose gum	Egrandis1_0	NCBI	Geneglob
**Brassicales**					
708	*Carica papaya*	Papaya	Papaya1.0	NCBI	The Papaya Genome Sequencing Consortium
709	*Arabidopsis halleri subsp. gemmifera*	-	Ahal_1.0	NCBI	TokyoTech
710	*Arabidopsis lyrata subsp. lyrata*	-	v.1.0	NCBI	JGI
711	*Arabidopsis thaliana*	-	TAIR10	NCBI	The Arabidopsis Information Resource
712	*Camelina sativa*	Camelina	Cs	NCBI	Agriculture & AgriFood Canada
713	*Capsella rubella*	Capsella	Caprub1_0	NCBI	JGI

No	Systematic Name	Common Name	Genome Version	Source	Submitter
714	*Brassica napus*	Rapeseed	Brassica_napus_assembly_v1.0	NCBI	BGI
715	*Brassica oleracea var. oleracea*	-	BOL	NCBI	CanSeq
716	*Brassica rapa*	Field mustard	Brapa_1.0	NCBI	Brassica rapa Genome Sequencing Project, BraGSP
717	*Raphanus raphanistrum subsp. raphanistrum*	-	ASM76984v1	NCBI	Michigan State University
718	*Raphanus sativus*	Radish	ASM80110v1	NCBI	Myongji University
719	*Aethionema arabicum*	-	VEGI_AA_v_1.0	NCBI	McGill
720	*Arabis alpina*	Alpine rockcress	A_alpina_V4	NCBI	TRANSNET
721	*Eutrema parvulum*	-	Eutrema_parvulum_v01	NCBI	University of Illinois
722	*Eutrema salsugineum*	Saltwater cress	Eutsalg1_0	NCBI	JGI
723	*Sisymbrium irio*	London rocket	VEGI_SI_v_1.0	NCBI	McGill
724	*Leavenworthia alabamica*	Alabama gladecress	VEGI_LA_v_1.0	NCBI	McGill
725	*Tarenaya hassleriana*	Pink queen	ASM46358v1	NCBI	BGI
**Malvales**					
726	*Gossypium arboreum*	Tree cotton	Gossypium_arboreum_v1.0	NCBI	BGI
727	*Gossypium raimondii*	New world cotton	Graimondii2_0	NCBI	JGI
728	*Theobroma cacao*	Cacao	Theobroma_cacao_20110822	NCBI	Cacao Genome Consortium
729	*Aquilaria agallochum*	-	Aquilaria_agallocha_v1	NCBI	Academia Sinica
**Sapindales**					
730	*Azadirachta indica*	Indian lilac	AzaInd2.0	NCBI	Centre for Cellular and Molecular Plateforms
731	*Citrus clementine*	Clementine	Citrus_clementina_v1.0	NCBI	International Citrus Genome Consortium
732	*Citrus sinensis*	Orange	Csi_valencia_1.0	NCBI	China Sweet Orange Genome Project
**Vitales**					
733	*Vitis vinifera*	Common grape vine	12X	NCBI	International Grape Genome Program
**Caryophyllales**					

No	Systematic Name	Common Name	Genome Version	Source	Submitter
734	*Amaranthus hypochondriacus*	Prince's feather	AHP 1.0	NCBI	Institute of Bioinformatics and Applied Biotechnology
735	*Amaranthus tuberculatus*	Tall waterhemp	ASM18065v1	NCBI	University of Illinois
736	*Beta vulgaris subsp. vulgaris*	-	RefBeet-1.2.1	NCBI	Beta vulgaris Resource
737	*Spinacia oleracea*	Spinach	Viroflay-1.0.1	NCBI	BeetSeq
738	*Dianthus caryophyllus*	Clove pink	DCA_r1.0	NCBI	KDRI
**Ericales**					
739	*Actinidia chinensis*	Kiwi fruit	Kiwifruit_v1	NCBI	Boyce Thompson Institute
740	*Vaccinium macrocarpon*	Large cranberry	ASM77533v1	NCBI	Rutgers University
741	*Diospyros lotus*	Caucasian persimmon	ASM77412v1	NCBI	University of California Davis
742	*Primula veris*	Cowslip	ASM78844v1	NCBI	University of Zurich
**Solanales**					
743	*Solanum arcanum*	-	Soarc10	NCBI	PRI
744	*Solanum habrochaites*	-	Sohab10	NCBI	PRI
745	*Solanum lycopersicum*	Tomato	SL2.40	NCBI	Solanaceae Genomics Project
746	*Solanum melongena*	Eggplant	SME_r2.5.1	NCBI	KDRI
747	*Solanum pennellii*	-	Sopen10	NCBI	PRI
748	*Solanum pimpinellifolium*	Currant tomato	Sol_pimpi_v1.0	NCBI	Sol Genomics Network
749	*Solanum tuberosum*	Potato	SolTub_3.0	NCBI	Potato Genome Sequencing Consortium
750	*Capsicum annuum*	Hot pepper	PGAv.1.5	NCBI	SNU
751	*Nicotiana sylvestris*	Woodland tobacco	Nsyl	NCBI	Philip Morris International R&D
752	*Nicotiana tomentosiformis*	-	Ntom_v01	NCBI	Philip Morris International R&D
**Lamiales**					
753	*Fraxinus excelsior*	European ash	BATG-0.4	NCBI	QMUL
754	*Penstemon centranthifolius*	Scarlet bugler	ASM73743v1	NCBI	Ohio State University
755	*Penstemon grinnellii*	Grinnell's beardtongue	ASM73742v1	NCBI	Ohio State University

No	Systematic Name	Common Name	Genome Version	Source	Submitter
756	*Sesamum indicum*	Sesame	S_indicum_v1.0	NCBI	Oil Crops Research Institute of the Chinese Academy of Agricultural Sciences
757	*Genlisea aurea*	Corkscrew plant	GenAur_1.0	NCBI	Lomonosov Moscow State University, Evolutionary Genomics Laboratory
758	*Mimulus guttatus*	Common monkey-flower	Mimgu1_0	NCBI	JGI
**Asterales**					
759	*Conyza canadensis*	Horseweed	ASM77593v1	NCBI	University of Tennessee

Table S2: L1s in genomic data

Table S2: Shows which genomes contain L1s (559/759) and the number of hits and length distribution of hits. L1 elements were detected with protein based searches using TBLASTN and nucleotide similarity searches using LASTZ. All hits had to satisfy a ‘reciprocal best hit’ check: they were screened with CENSOR against the Repbase library of known repeats, and kept if the best hit was an L1 element (not some other repeat). Overlapping hits were merged to produce a non-redundant set of L1s for each genome. The Notes column highlights unusual observations.

Species	# L1 hits	Length distribution (bp)	Notes
**METAZOA**			
**MAMMALIA**			
Tachyglossus aculeatus	0	-	
Ornithorhynchus anatinus	0	-	3 tiny fragments found in platypus while mining for L1s. Checked against a pacbio assmebly - found 6 similar fragments, but all are flanked by L2 and L3 5’ and 3’ elements. More likely to be of the L2/CR1 clade.
Monodelphis domestica	368300	min 26, median 396, max 12466	
Macropus eugenii	120437	min 29, median 469, max 7787	
Sarcophilus harrisii	132100	min 26, median 264, max 8834	
Dasypus novemcinctus	249548	min 26, median 503, max 13923	
Choloepus hoffmanni	166782	min 35, median 612, max 11920	
Chrysochloris asiatica	188020	min 26, median 320, max 7157	
Echinops telfairi	80067	min 26, median 347, max 7224	
Orycteropus afer afer	280733	min 26, median 338, max 10311	
Elephantulus edwardii	337288	min 26, median 302, max 7108	
Trichechus manatus latirostris	230983	min 26, median 435, max 14101	
Procavia capensis	156595	min 32, median 736, max 11303	
Loxodonta africana	253130	min 26, median 474, max 14036	
Erinaceus europaeus	130943	min 26, median 308, max 10024	
Sorex araneus	133563	min 26, median 332, max 10424	
Condylura cristata	89051	min 26, median 275, max 7199	
Pteropus alecto	148833	min 26, median 401, max 12178	
Pteropus vampyrus	86961	min 32, median 309, max 9856	
Eidolon helvum	67966	min 32, median 219, max 8961	
Megaderma lyra	58222	min 32, median 210, max 9765	
Rhinolophus ferrumequinum	75604	min 33, median 212, max 9717	
Pteronotus parnellii	69432	min 32, median 188, max 9062	
Eptesicus fuscus	110090	min 26, median 315, max 11254	
Myotis brandtii	120200	min 26, median 317, max 10288	
Myotis davidii	95878	min 26, median 267, max 11658	
Myotis lucifugus	118834	min 26, median 366, max 12207	

**Species**	**# L1 hits**	**Length distribution (bp)**	**Notes**
Ceratotherium simum simum	197102	min 26, median 392, max 12095	
Equus przewalskii	123829	min 29, median 377, max 15366	
Equus caballus (Thoroughbred)	200731	min 26, median 410, max 13504	
Equus caballus (Mongolian)	115028	min 32, median 324, max 15282	
Manis pentadactyla	133451	min 29, median 195, max 10267	
Felis catus	206663	min 26, median 371, max 11426	
Panthera tigris altaica	195025	min 26, median 385, max 13604	
Canis lupus familiaris	189577	min 26, median 368, max 11672	
Ursus maritimus	193442	min 26, median 378, max 13373	
Ailuropoda melanoleuca	124602	min 26, median 289, max 11844	
Leptonychotes weddellii	146135	min 26, median 330, max 10738	
Odobenus rosmarus divergens	164744	min 26, median 371, max 11279	
Mustela putorius furo	193128	min 26, median 362, max 10819	
Camelus dromedarius	155118	min 26, median 344, max 11206	
Camelus ferus	132511	min 26, median 328, max 11795	
Vicugna pacos	194213	min 26, median 346, max 10598	
Sus scrofa (Duroc)	251960	min 26, median 350, max 11196	
Sus scrofa (Tibetan)	169743	min 26, median 383, max 13253	
Sus scrofa (Ellegaard Gottingen minipig)	143892	min 29, median 372, max 11185	
Balaenoptera acutorostrata scammoni	239254	min 26, median 401, max 10933	
Physeter catodon	162334	min 29, median 407, max 13070	
Lipotes vexillifer	198338	min 26, median 458, max 15715	
Tursiops truncatus	206606	min 29, median 296, max 14307	
Orcinus orca	243602	min 26, median 415, max 12828	
Pantholops hodgsonii	140895	min 26, median 293, max 10045	
Capra hircus	172850	min 26, median 307, max 11295	
Ovis aries (Texel)	170755	min 26, median 320, max 12550	
Ovis aries musimon	118754	min 26, median 287, max 12652	
Bubalus bubalis	170578	min 26, median 372, max 16417	
Bison bison bison	222104	min 26, median 366, max 12472	
Bos mutus	125229	min 26, median 291, max 12276	
Bos indicus	162944	min 26, median 329, max 12582	
Bos taurus	183282	min 26, median 338, max 12798	
Ochotona princeps	63231	min 26, median 319, max 7183	
Oryctolagus cuniculus	165637	min 26, median 386, max 13252	
Ictidomys tridecemlineatus	153340	min 29, median 359, max 8996	
Heterocephalus glaber	211256	min 26, median 365, max 9489	
Fukomys damarensis	179459	min 26, median 377, max 10470	
Cavia aperea	215721	min 26, median 344, max 12977	

Species	# L1 hits	Length distribution (bp)	Notes
Cavia porcellus	202062	min 26, median 524, max 13197	
Chinchilla lanigera	162543	min 26, median 374, max 9883	
Octodon degus	181971	min 26, median 365, max 7751	
Dipodomys ordii	39663	min 29, median 111, max 7728	
Jaculus jaculus	132427	min 26, median 365, max 7362	
Nannospalax galili	136751	min 26, median 407, max 7868	
Mesocricetus auratus	117603	min 26, median 374, max 9681	
Cricetulus griseus	90706	min 26, median 329, max 11338	
Microtus ochrogaster	91178	min 26, median 332, max 6404	
Peromyscus maniculatus bairdii	72159	min 26, median 335, max 9389	
Rattus norvegicus	193696	min 26, median 518, max 11866	
Mus musculus	197761	min 26, median 502, max 18858	
Tupaia belangeri	68588	min 32, median 463, max 10269	
Tupaia chinensis	110904	min 26, median 393, max 11021	
Galeopterus variegatus	176635	min 26, median 174, max 9846	
Otolemur garnettii	196900	min 26, median 363, max 8925	
Microcebus murinus	106690	min 32, median 286, max 10808	
Tarsius syrichta	186988	min 26, median 385, max 16139	
Callithrix jacchus	271588	min 26, median 430, max 12568	
Saimiri boliviensis boliviensis	203913	min 26, median 397, max 11877	
Rhinopithecus roxellana	185188	min 26, median 443, max 15293	
Nasalis larvatus	202605	min 26, median 395, max 10716	
Chlorocebus sabaeus	233704	min 26, median 386, max 12077	
Macaca fascicularis	252545	min 26, median 400, max 13479	
Macaca mulatta	238315	min 26, median 329, max 10898	
Papio anubis	263380	min 26, median 404, max 14592	
Nomascus leucogenys	244864	min 26, median 416, max 14766	
Pongo abelii	297567	min 26, median 421, max 13087	
Gorilla gorilla gorilla	263332	min 26, median 395, max 27891	
Pan paniscus	233415	min 26, median 440, max 14715	
Pan troglodytes	264123	min 26, median 410, max 16238	
Homo sapiens	257636	min 26, median 406, max 18773	
**SAUROPSIDA**			
Apalone spinifera	2560	min 29, median 372, max 5911	
Pelodiscus sinensis	2031	min 32, median 511, max 7157	
Chelonia mydas	5240	min 32, median 477, max 8320	
Chrysemys picta bellii	3714	min 32, median 551, max 8573	
Struthio camelus australis	61	min 75, median 254, max 3825	
Tinamus guttatus	27	min 83, median 281, max 1197	
Anas platyrhynchos	51	min 98, median 236, max 4578	
Lyrurus tetrix tetrix	21	min 72, median 248, max 780	
Gallus gallus	22	min 87, median 192, max 4412	
Coturnix japonica	8	min 82, median 146, max 893	
Meleagris gallopavo	14	min 86, median 230, max 4020	

Species	# L1 hits	Length distribution (bp)	Notes
Colinus virginianus	127	min 70, median 357, max 4090	
Acanthisitta chloris	42	min 90, median 287, max 4493	
Manacus vitellinus	36	min 102, median 251, max 4602	
Zonotrichia albicollis	37	min 65, median 211, max 4512	
Geospiza fortis	27	min 114, median 272, max 4489	
Serinus canaria	386	min 61, median 103, max 4516	
Taeniopygia guttata	30	min 84, median 258, max 4354	
Ficedula albicollis	34	min 108, median 272, max 4569	
Pseudopodoces humilis	40	min 68, median 227, max 4312	
Corvus brachyrhynchos	57	min 68, median 248, max 4515	
Corvus cornix cornix	43	min 76, median 257, max 4586	
Ara macao	50	min 107, median 278, max 2083	
Amazona vittata	63	min 72, median 268, max 3513	
Melopsittacus undulatus	38	min 73, median 280, max 3737	
Nestor notabilis	54	min 95, median 267, max 3928	
Falco cherrug	59	min 101, median 280, max 4512	
Falco peregrinus	58	min 86, median 276, max 3806	
Cariama cristata	62	min 125, median 273, max 4604	
Merops nubicus	40	min 121, median 269, max 4075	
Picoides pubescens	37	min 72, median 212, max 641	
Buceros rhinoceros silvestris	43	min 71, median 289, max 4513	
Apaloderma vittatum	32	min 117, median 316, max 3858	
Leptosomus discolour	46	min 128, median 267, max 3646	
Haliaeetus albicilla	65	min 116, median 290, max 4593	
Haliaeetus leucocephalus	68	min 85, median 290, max 4600	
Aquila chrysaetos canadensis	64	min 127, median 291, max 4567	
Cathartes aura	81	min 89, median 305, max 4211	
Tyto alba	100	min 75, median 272, max 4540	
Colius striatus	42	min 85, median 275, max 4391	
Charadrius vociferus	64	min 106, median 290, max 4520	
Balearica regulorum gibbericeps	61	min 111, median 316, max 4608	
Chlamydotis macqueenii	57	min 69, median 270, max 4065	
Cuculus canorus	42	min 119, median 252, max 3744	
Fulmarus glacialis	68	min 70, median 280, max 4628	
Aptenodytes forsteri	79	min 76, median 269, max 4724	
Pygoscelis adeliae	75	min 89, median 281, max 4834	
Phalacrocorax carbo	57	min 50, median 289, max 3731	
Pelecanus crispus	57	min 121, median 286, max 4581	
Nipponia nippon	71	min 76, median 296, max 4564	
Egretta garzetta	66	min 92, median 276, max 4598	
Phaethon lepturus	47	min 151, median 308, max 4615	
Gavia stellata	74	min 88, median 262, max 4577	
Tauraco erythrolophus	45	min 115, median 289, max 4514	

Species	# L1 hits	Length distribution (bp)	Notes
Opisthocomus hoazin	80	min 47, median 326, max 4184	
Columba livia	55	min 109, median 281, max 4523	
Pterocles gutturalis	56	min 106, median 279, max 4508	
Calypte anna	31	min 137, median 286, max 3924	
Chaetura pelagica	29	min 100, median 253, max 4553	
Caprimulgus carolinensis	68	min 83, median 268, max 4374	
Eurypyga helias	41	min 102, median 251, max 4514	
Mesitornis unicolor	41	min 130, median 300, max 4484	
Podiceps cristatus	50	min 127, median 282, max 4730	
Phoenicopterus ruber ruber	77	min 124, median 278, max 4626	
Alligator mississippiensis	4412	min 32, median 624, max 13520	
Alligator sinensis	6253	min 29, median 569, max 9201	
Crocodylus porosus	3948	min 32, median 579, max 7709	
Gavialis gangeticus	3989	min 29, median 592, max 13044	
Pogona vitticeps	6767	min 32, median 440, max 4971	
Anolis carolinensis	12551	min 26, median 518, max 9160	
Vipera berus berus	5714	min 41, median 585, max 5375	
Crotalus mitchellii pyrrhus	3591	min 53, median 390, max 4019	
Ophiophagus hannah	9742	min 38, median 364, max 6767	
Python bivittatus	4679	min 38, median 542, max 5737	
**AMPHIBIA**			
Nanorana parkeri	18787	min 38, median 767, max 5619	
Xenopus laevis	26764	min 32, median 623, max 6227	
Xenopus tropicalis	8638	min 32, median 659, max 9353	
**NEOPTERYGII**			
Lepisosteus oculatus	876	min 65, median 596, max 5363	
Anguilla anguilla	1672	min 47, median 422, max 5766	
Anguilla japonica	1833	min 53, median 517, max 5112	
Danio rerio	5140	min 29, median 614, max 8441	
Astyanax mexicanus	975	min 41, median 479, max 4962	
Oryzias latipes	4496	min 41, median 641, max 5918	
Poecilia formosa	1505	min 29, median 659, max 5676	
Xiphophorus maculatus	1217	min 47, median 652, max 5655	
Fundulus heteroclitus	2825	min 38, median 617, max 5485	
Takifugu flavidus	709	min 59, median 564, max 5147	
Takifugu rubripes	825	min 44, median 553, max 5497	
Tetraodon nigroviridis	232	min 32, median 539, max 5422	
Cynoglossus semilaevis	196	min 73, median 383, max 4385	
Haplochromis burtoni	1666	min 44, median 729, max 6085	
Pundamilia nyererei	1852	min 41, median 695, max 5895	
Maylandia zebra	2116	min 44, median 680, max 6900	
Neolamprologus brichardi	1915	min 47, median 620, max 5431	
Oreochromis niloticus	3265	min 47, median 698, max 6629	
Sebastes nigrocinctus	912	min 74, median 494, max 6241	

Species	# L1 hits	Length distribution (bp)	Notes
Sebastes rubrivinctus	878	min 68, median 507, max 6040	
Gasterosteus aculeatus	507	min 71, median 493, max 4992	
Gadus morhua	1635	min 44, median 410, max 5389	
**CHONDRICHTHYES**			
Callorhinchus milii	209	min 32, median 368, max 2778	
Carcharhinus brachyurus	3388	min 44, median 200, max 5019	
**ECDYSOZOA**			
Ephemera danica	114	min 56, median 700, max 3669	
Ladona fulva	477	min 38, median 770, max 3645	
Pediculus humanus corporis	68	min 89, median 308, max 1819	
Frankliniella occidentalis	193	min 70, median 256, max 4011	
Diaphorina citri	1175	min 75, median 289, max 3389	
Pachypsylla venusta	243	min 84, median 217, max 2029	
Acyrthosiphon pisum	155	min 80, median 672, max 2723	
Nilaparvata lugens	2093	min 44, median 452, max 8772	
Oncopeltus fasciatus	244	min 72, median 203, max 1815	
Rhodnius prolixus	165	min 74, median 641, max 3250	
Cimex lectularius	1070	min 81, median 203, max 2489	
Onthophagus taurus	161	min 83, median 669, max 2726	
Agrilus planipennis	197	min 35, median 455, max 2009	
Tribolium castaneum	34	min 53, median 686, max 2591	
Anoplophora glabripennis	121	min 83, median 611, max 2501	
Leptinotarsa decemlineata	484	min 59, median 785, max 2698	
Dendroctonus ponderosae	135	min 53, median 569, max 2322	
Mengenilla moldrzyki	15	min 73, median 189, max 2534	
Aedes aegypti	3589	min 38, median 737, max 5629	
Culex quinquefasciatus	947	min 41, median 743, max 5511	
Anopheles albimanus	45	min 88, median 192, max 4306	
Anopheles arabiensis	179	min 59, median 442, max 3029	
Anopheles atroparvus	191	min 71, median 516, max 2842	
Anopheles christyi	112	min 89, median 446, max 2247	
Anopheles culicifacies	279	min 62, median 407, max 3851	
Anopheles darlingi	35	min 71, median 176, max 3308	
Anopheles dirus	390	min 88, median 575, max 3077	
Anopheles epiroticus	194	min 41, median 486, max 4385	
Anopheles farauti	403	min 59, median 520, max 4720	
Anopheles funestus	192	min 70, median 551, max 4515	
Anopheles gambiae	321	min 35, median 546, max 4880	
Anopheles maculatus	97	min 65, median 533, max 2325	
Anopheles melas	138	min 71, median 376, max 3158	
Anopheles merus	250	min 65, median 425, max 4176	
Anopheles minimus	163	min 107, median 493, max 3995	
Anopheles quadriannulatus	185	min 65, median 399, max 3170	
Anopheles sinensis	104	min 92, median 499, max 4494	

**Species**	**# L1 hits**	**Length distribution (bp)**	**Notes**
Anopheles stephensi	110	min 91, median 494, max 3220	
Mayetiola destructor	29	min 83, median 677, max 2666	
Lutzomyia longipalpis	11	min 150, median 1016, max 2372	
Phlebotomus papatasi	36	min 68, median 1082, max 2675	
Ceratitis capitata	112	min 53, median 335, max 7274	
Drosophila albomicans	50	min 73, median 136, max 1699	
Drosophila ananassae	24	min 68, median 179, max 1091	
Drosophila biarmipes	25	min 78, median 225, max 1759	
Drosophila bipectinata	19	min 68, median 329, max 1503	
Drosophila elegans	32	min 63, median 186, max 1241	
Drosophila erecta	24	min 82, median 200, max 956	
Drosophila eugracilis	25	min 89, median 227, max 1472	
Drosophila ficusphila	77	min 88, median 758, max 3911	
Drosophila grimshawi	70	min 76, median 114, max 882	
Drosophila kikkawai	36	min 87, median 221, max 1311	
Drosophila melanogaster	16	min 97, median 134, max 2001	
Drosophila miranda	30	min 76, median 148, max 4515	
Drosophila mojavensis	113	min 68, median 210, max 1345	
Drosophila persimilis	36	min 67, median 122, max 837	
Drosophila pseudoobscura pseudoobscura	34	min 70, median 157, max 953	
Drosophila rhopaloa	18	min 104, median 179, max 1143	
Drosophila sechellia	17	min 92, median 225, max 629	
Drosophila simulans	19	min 77, median 169, max 531	
Drosophila suzukii	38	min 86, median 174, max 5555	
Drosophila takahashii	26	min 96, median 180, max 1660	
Drosophila virilis	68	min 69, median 125, max 2055	
Drosophila willistoni	41	min 72, median 152, max 1050	
Drosophila yakuba	22	min 66, median 182, max 2240	
Musca domestica	117	min 44, median 393, max 2615	
Glossina austeni	156	min 71, median 187, max 1413	
Glossina brevipalpis	188	min 81, median 232, max 1514	
Glossina fuscipes fuscipes	162	min 69, median 169, max 2801	
Glossina morsitans morsitans	151	min 72, median 176, max 2225	
Glossina pallidipes	139	min 75, median 168, max 1505	
Limnephilus lunatus	105	min 44, median 266, max 1604	
Papilio glaucus	144	min 35, median 410, max 1434	
Papilio polytes	13	min 126, median 275, max 2819	
Papilio xuthus	22	min 138, median 1647, max 2328	
Heliconius melpomene melpomene	12	min 89, median 150, max 2300	
Melitaea cinxia	30	min 85, median 213, max 2459	
Danaus plexippus	27	min 71, median 416, max 3429	
Bombyx mori	4934	min 82, median 306, max 2427	
Manduca sexta	33	min 70, median 772, max 2360	

**Species**	**# L1 hits**	**Length distribution (bp)**	**Notes**
Plutella xylostella	143	min 80, median 1475, max 2747	
Athalia rosae	26	min 64, median 214, max 2468	
Cephus cinctus	52	min 94, median 300, max 2198	
Orussus abietinus	31	min 89, median 362, max 1745	
Ceratosolen solmsi marchali	258	min 75, median 163, max 1425	
Nasonia giraulti	41	min 76, median 206, max 2043	
Nasonia longicornis	32	min 83, median 200, max 2489	
Nasonia vitripennis	84	min 67, median 151, max 2216	
Copidosoma floridanum	157	min 74, median 386, max 2177	
Trichogramma pretiosum	53	min 80, median 440, max 2543	
Microplitis demolitor	139	min 88, median 929, max 2435	
Megachile rotundata	98	min 85, median 616, max 2474	
Apis dorsata	31	min 95, median 247, max 1367	
Apis florea	42	min 73, median 174, max 1045	
Apis mellifera	35	min 85, median 199, max 2018	
Bombus impatiens	69	min 80, median 603, max 2302	
Bombus terrestris	52	min 90, median 301, max 1412	
Linepithema humile	54	min 77, median 209, max 2480	
Camponotus floridanus	37	min 86, median 232, max 9746	
Acromyrmex echinatior	79	min 68, median 163, max 2261	
Atta cephalotes	54	min 71, median 149, max 3899	
Solenopsis invicta	78	min 81, median 177, max 2619	
Pogonomyrmex barbatus	29	min 85, median 196, max 1617	
Harpegnathos saltator	294	min 83, median 187, max 1510	
Cerapachys biroi	47	min 68, median 191, max 1883	
Blattella germanica	2437	min 47, median 688, max 4506	
Zootermopsis nevadensis	91	min 47, median 488, max 2552	
Locusta migratoria	6154	min 29, median 973, max 5039	
Daphnia pulex	284	min 44, median 744, max 5142	
Eurytemora affinis	404	min 85, median 450, max 3719	
Lepeophtheirus salmonis	18478	min 26, median 473, max 4755	
Hyalella azteca	211	min 41, median 287, max 2477	
Strigamia maritima	62	min 90, median 855, max 3003	
Stegodyphus mimosarum	929	min 65, median 1223, max 2759	
Latrodectus hesperus	273	min 56, median 770, max 2636	
Parasteatoda tepidariorum	125	min 98, median 1082, max 2492	
Tetranychus urticae	54	min 104, median 273, max 1964	
Dermatophagoides farinae	14	min 109, median 164, max 1677	
Sarcoptes scabiei type canis	49	min 83, median 233, max 2369	
Achipteria coleoptrata	9	min 90, median 819, max 2525	
Hypochthonius rufulus	22	min 81, median 302, max 2180	
Platynothrus peltifer	17	min 203, median 833, max 1340	
Steganacarus magnus	39	min 107, median 362, max 2664	
Ixodes ricinus	871	min 41, median 992, max 4181	

Species	# L1 hits	Length distribution (bp)	Notes
Ixodes scapularis	2134	min 41, median 830, max 4043	
Rhipicephalus microplus	216	min 134, median 673, max 3144	
Metaseiulus occidentalis	75	min 96, median 1058, max 1820	
Varroa destructor	19	min 95, median 168, max 692	
Centruroides exilicauda	729	min 62, median 1001, max 3491	
Mesobuthus martensii	370	min 75, median 857, max 4684	
Limulus polyphemus	583	min 44, median 674, max 4269	
Trichinella spiralis	15	min 79, median 284, max 1578	
Ascaris suum	41	min 98, median 694, max 2568	
Elaeophora elaphi	0	-	
Onchocerca volvulus	0	-	
Steinernema monticolum	42	min 85, median 405, max 824	
Panagrellus redivivus	16	min 102, median 495, max 2278	
Haemonchus contortus	721	min 38, median 398, max 2289	
Necator americanus	50	min 77, median 553, max 1634	
Heterorhabditis bacteriophora	1	min 244, median 244, max 244	
Caenorhabditis angaria	16	min 139, median 215, max 1606	
Caenorhabditis brenneri	42	min 107, median 457, max 3145	
Caenorhabditis briggsae	19	min 93, median 240, max 1476	
Caenorhabditis elegans	12	min 211, median 401, max 922	
Caenorhabditis japonica	56	min 38, median 662, max 2528	
Caenorhabditis sp. 11 MAF-2010	14	min 93, median 308, max 1032	
Priapulus caudatus	296	min 53, median 329, max 3346	
**ROTIFERA**			
Adineta vaga	45	min 111, median 653, max 3304	
**PLATYHELMINTHES**			
Schistosoma curassoni	173	min 52, median 64, max 2248	
Schistosoma haematobium	159	min 50, median 62, max 2059	
Schistosoma japonicum	136	min 44, median 464, max 6036	
Schistosoma mansoni	98	min 54, median 61, max 2968	
Schistosoma margrebowiei	142	min 50, median 62, max 3942	
Schistosoma mattheei	125	min 51, median 62, max 5065	
Schistosoma rodhaini	95	min 51, median 62, max 4927	
Clonorchis sinensis	411	min 50, median 213, max 2207	
Echinococcus granulosus	4	min 688, median 1110, max 1514	
Echinococcus multilocularis	8	min 128, median 341, max 1246	
Hymenolepis microstoma	7	min 92, median 719, max 1445	
Protopolystoma xenopodis	25	min 140, median 779, max 4759	
**ANNELIDA**			
Capitella teleta	25	min 89, median 452, max 2708	
Helobdella robusta	652	min 35, median 437, max 2483	
**MOLLUSCA**			
Crassostrea gigas	1344	min 47, median 911, max 6771	

**Species**	**# L1 hits**	**Length distribution (bp)**	**Notes**
Lottia gigantea	559	min 59, median 862, max 5151	
Aplysia californica	1819	min 47, median 216, max 7250	
Biomphalaria glabrata	761	min 53, median 253, max 3280	
**CNIDARIA**			
Nematostella vectensis	191	min 74, median 569, max 5309	
Hydra vulgaris	1607	min 35, median 678, max 5900	
**TENTACULATA**			
Mnemiopsis leidyi	68	min 93, median 868, max 2366	
**PLACOZOA**			
Trichoplax adhaerens	0	-	
**ECHINOIDEA**			
Amphimedon queenslandica	12	min 110, median 580, max 1238	
Lytechinus variegatus	2319	min 44, median 470, max 5196	
Strongylocentrotus purpuratus	4014	min 32, median 398, max 6094	
**ASTEROIDEA**			
Patiria miniata	328	min 59, median 704, max 4514	
**ENTEROPNEUSTA**			
Saccoglossus kowalevskii	2412	min 32, median 670, max 6255	
**TUNICATA**			
Ciona intestinalis	110	min 77, median 546, max 4833	
Ciona savignyi	2744	min 38, median 839, max 11990	
Botryllus schlosseri	726	min 68, median 1564, max 4179	
Oikopleura dioica	5	min 282, median 983, max 2117	
**LEPTOCARDII**			
Branchiostoma floridae	759	min 53, median 845, max 5842	
**CEPHALASPIDOMORPHI**			
Lethenteron camtschaticum	918	min 47, median 651, max 6536	
Petromyzon marinus	529	min 44, median 644, max 7409	
**SARCOPTERYGII**			
Latimeria chalumnae	12303	min 38, median 739, max 12520	
**FUNGI**			
Agaricus bisporus var. bisporus	14	min 527, median 1358, max 3596	
Agaricus bisporus var. burnettii	15	min 674, median 1490, max 3473	
Schizophyllum commune	2	min 2963, median 2963, max 3002	
Coprinopsis cinerea	4	min 1052, median 1184, max 2629	
Moniliophthora roreri	2	min 908, median 908, max 3198	
Laccaria bicolor	2	min 1262, median 1262, max 2974	
Auricularia subglabra	73	min 650, median 3038, max 3746	
Coniophora puteana	7	min 515, median 1616, max 3560	

**Species**	**# L1 hits**	**Length distribution (bp)**	**Notes**
Serpula lacrymans	0	-	
Punctularia strigosozonata	15	min 965, median 3182, max 3689	
Gloeophyllum trabeum	11	min 428, median 1256, max 3527	
Fomitiporia mediterranea	1	min 1142, median 1142, max 1142	
Dichomitus squalens	4	min 1656, median 2546, max 3374	
Fibroporia radiculosa	1	min 770, median 770, max 770	
Phanerochaete carnosa	10	min 530, median 1421, max 2897	
Postia placenta	5	min 1187, median 1703, max 2944	
Trametes versicolor	29	min 2426, median 3143, max 3512	
Heterobasidion irregulare	5	min 1529, median 1706, max 3103	
Stereum hirsutum	4	min 2108, median 2324, max 3530	
Spizellomyces punctatus	0	-	
Batrachochytrium dendrobatidis	0	-	
Diplodia corticola	0	-	
Neofusicoccum parvum	0	-	
Baudoinia panamericana	0	-	
Cercospora beticola	0	-	
Pseudocercospora fijiensis	8	min 896, median 2708, max 2753	
Sphaerulina musiva	1	min 1589, median 1589, max 1589	
Zymoseptoria tritici	5	902, median 1595, max 1685	
Aureobasidium namibiae	0	-	
Aureobasidium subglaciale	0	-	
Coniosporium apollinis	0	-	
Paraphaeosphaeria sporulosa	0	-	
Parastagonospora nodorum	2	min 1352, median 1352, max 2459	
Leptosphaeria maculans	12	min 755, median 1685, max 2792	
Alternaria alternata	0	-	
Bipolaris maydis	0	-	
Bipolaris oryzae	0	-	
Bipolaris sorokiniana	0	-	
Bipolaris victoriae	0	-	
Bipolaris zeicola	0	-	
Pyrenophora teres	0	-	
Pyrenophora tritici-repentis	0	-	
Setosphaeria turcica	0	-	

Species	# L1 hits	Length distribution (bp)	Notes
Verruconis gallopava	0	-	
Cyphellophora europaea	0	-	
Capronia coronata	0	-	
Capronia epimyces	0	-	
Cladophialophora bantiana	0	-	
Cladophialophora carrionii	0	-	
Cladophialophora immunda	0	-	
Cladophialophora psammophila	0	-	
Cladophialophora yegresii	0	-	
Exophiala aquamarina	0	-	
Exophiala dermatitidis	0	-	
Exophiala mesophila	0	-	
Exophiala oligosperma	0	-	
Exophiala spinifera	0	-	
Exophiala xenobiotica	0	-	
Fonsecaea erecta	0	-	
Fonsecaea monophora	0	-	
Fonsecaea multimorphosa	0	-	
Fonsecaea nubica	0	-	
Fonsecaea pedrosoi	0	-	
Phialophora attae	0	-	
Rhinocladiella mackenziei	0	-	
Aspergillus aculeatus	0	-	
Aspergillus bombycis	0	-	
Aspergillus clavatus	0	-	
Aspergillus fischeri	0	-	
Aspergillus flavus	0	-	
Aspergillus fumigatus	0	-	
Aspergillus glaucus	0	-	
Aspergillus nidulans	0	-	
Aspergillus niger	0	-	
Aspergillus nomius	0	-	
Aspergillus oryzae	0	-	
Aspergillus terreus	0	-	
Penicilliopsis zonata	0	-	
Penicillium arizonense	0	-	
Penicillium chrysogenum	0	-	
Penicillium digitatum	0	-	
Penicillium expansum	0	-	
Penicillium marneffei	0	-	
Rasamsonia emersonii	0	-	

Species	# L1 hits	Length distribution (bp)	Notes
Talaromyces atroroseus	1	min 1589, median 1589, max 1589	
Talaromyces stipitatus	7	min 239, median 863, max 1274	
Ajellomyces dermatitidis	25	min 494, median 953, max 1439	
Arthroderma otae	1	min 318, median 318, max 318	
Coccidioides immitis	3	min 944, median 944, max 1433	
Coccidioides posadasii	0	-	
Histoplasma capsulatum	1	min 1442, median 1442, max 1442	
Nannizzia gypsea	0	-	
Paracoccidioides brasiliensis	2	min 662, median 662, max 695	
Paracoccidioides lutzii	0	-	
Trichophyton benhamiae	0	-	
Trichophyton rubrum	0	-	
Trichophyton verrucosum	0	-	
Uncinocarpus reesii	2	min 758, median 758, max 794	
Endocarpon pusillum	7	min 215, median 323, max 953	
Tilletiaria anomala	0	-	
Botryotinia fuckeliana	0	-	
Glarea lozoyensis	0	-	
Marssonina brunnea	0	-	
Phialocephala scopiformis	0	-	
Sclerotinia sclerotiorum	0	-	
Pseudogymnoascus destructans	3	min 1009, median 1494, max 2726	
Pseudogymnoascus verrucosus	0	-	
Malassezia globosa	0	-	
Malassezia pachydermatis	0	-	
Malassezia sympodialis	5	min 347, median 947, max 1133	
Rhodotorula graminis	0	-	
Rhodotorula toruloides	1	min 2537, median 2537, max 2537	
Mixia osmundae	0	-	
Arthrobotrys oligospora	0	-	
Dactylellina haptotyla	1	min 1802, median 1802, max 1802	
Tuber melanosporum	2	min 218, median 218, max 1871	
Pneumocystis carinii	0	-	
Pneumocystis jirovecii	0	-	
Pneumocystis murina	0	-	
Melampsora larici-populina	6	min 1373, median 1475, max 2144	
Puccinia graminis	1	min 752, median 752, max 752	
Candida albicans	8	min 221, median 1097, max 3446	

**Species**	**# L1 hits**	**Length distribution (bp)**	**Notes**
Candida auris	1	min 2327, median 2327, max 2327	
Candida dubliniensis	35	min 41, median 390, max 4952	
Candida glabrata	0	-	
Candida orthopsilosis	0	-	
Candida tanzawaensis	1	min 1844, median 1844, max 1844	
Candida tenuis	0	-	
Candida tropicalis	0	-	
Lodderomyces elongisporus	0	-	
Ascoidea rubescens	0	-	
Babjeviella inositovora	0	-	
Clavispora lusitaniae	0	-	
Cyberlindnera jadinii	0	-	
Debaryomyces fabryi	0	-	
Debaryomyces hansenii	0	-	
Eremothecium cymbalariae	0	-	
Eremothecium gossypii	0	-	
Eremothecium sinecaudum	0	-	
Hyphopichia burtonii	0	-	
Kazachstania africana	0	-	
Kazachstania naganishii	0	-	
Kluyveromyces lactis	0	-	
Kluyveromyces marxianus	0	-	
Komagataella phaffii	0	-	
Kuraishia capsulata	0	-	
Lachancea lanzarotensis	0	-	
Lachancea thermotolerans	0	-	
Metschnikowia bicuspidata	3	min 158, median 410, max 1358	
Meyerozyma guilliermondii	0	-	
Naumovozyma castellii	0	-	
Naumovozyma dairenensis	0	-	
Ogataea parapolymorpha	0	-	
Ogataea polymorpha	0	-	
Saccharomyces cerevisiae	0	-	
Saccharomyces eubayanus	0	-	
Spathaspora passalidarum	0	-	
Sugiyamaella lignohabitans	0	-	
Tetrapisispora blattae	0	-	
Tetrapisispora phaffii	0	-	

Species	# L1 hits	Length distribution (bp)	Notes
Torulaspora delbrueckii	0	-	
Vanderwaltozyma polyspora	0	-	
Yarrowia lipolytica	36	min 89, median 926, max 6079	
Zygosaccharomyces rouxii	0	-	
Wickerhamomyces anomalus	4	min 449, median 458, max 2606	
Wickerhamomyces ciferrii	8	min 230, median 1787, max 2927	
Scheffersomyces stipitis	25	min 1679, median 3077, max 3386	
Pichia kudriavzevii	0	-	
Pichia membranifaciens	0	-	
Schizosaccharomyces cryophilus	0	-	
Schizosaccharomyces japonicus	0	-	
Schizosaccharomyces octosporus	0	-	
Schizosaccharomyces pombe	0	-	
Metarhizium acridum	0	-	
Metarhizium brunneum	0	-	
Metarhizium majus	0	-	
Metarhizium robertsii	0	-	
Pochonia chlamydosporia	0	-	
Beauveria bassiana	2	min 404, median 404, max 1391	
Cordyceps militaris	0	-	
Isaria fumosorosea	0	-	
Trichoderma atroviride	0	-	
Trichoderma gamsii	0	-	
Trichoderma reesei	0	-	
Trichoderma virens	0	-	
Fusarium fujikuroi	0	-	
Fusarium graminearum	0	-	
Fusarium oxysporum	4	min 1260, median 1343, max 1523	
Fusarium pseudograminearum	0	-	
Fusarium verticillioides	1	min 1292, median 1292, max 1292	
Nectria haematococca	0	-	
Purpureocillium lilacinum	0	-	
Colletotrichum fioriniae	0	-	
Colletotrichum gloeosporioides	1	min 1235, median 1235, max 1235	
Colletotrichum graminicola	0	-	

Species	# L1 hits	Length distribution (bp)	Notes
Colletotrichum higginsianum	3	min 913, median 1328, max 2177	
Colletotrichum orchidophilum	1	min 2174, median 2174, max 2174	
Verticillium alfalfae	0	-	
Verticillium dahliae	0	-	
Magnaporthe oryzae	0	-	
Gaeumannomyces tritici	0	-	
Scedosporium apiospermum	0	-	
Grosmannia clavigera	0	-	
Sporothrix schenckii	0	-	
Chaetomium globosum	0	-	
Chaetomium thermophilum	0	-	
Neurospora crassa	0	-	
Neurospora tetrasperma	0	-	
Podospora anserina	0	-	
Sordaria macrospora	1	min 1025, median 1025, max 1025	
Thermothelomyces thermophila	2	min 359, median 359, max 722	
Thielavia terrestris	6	min 530, median 554, max 848	
Phaeoacremonium minimum	0	-	
Eutypa lata	0	-	
Pestalotiopsis fici	0	-	
Cryptococcus amylolentus	0	-	
Cryptococcus gattii	0	-	
Cryptococcus neoformans var. grubii	0	-	
Cryptococcus neoformans var. neoformans JEC21	0	-	
Cryptococcus neoformans var. neoformans B-3501A	0	-	
Kockovaella imperatae	0	-	
Kwoniella bestiolae	0	-	
Kwoniella dejecticola	0	-	
Kwoniella mangroviensis	0	-	
Kwoniella pini	0	-	
Tremella mesenterica	0	-	
Tsuchiyaea wingfieldii	0	-	
Cutaneotrichosporon oleaginosus	0	-	
Trichosporon asahii	0	-	
Anthracocystis flocculosa	0	-	
Kalmanozyma brasiliensis	0	-	

Species	# L1 hits	Length distribution (bp)	Notes
Moesziomyces antarcticus	0	-	
Pseudozyma hubeiensis	0	-	
Ustilago maydis	1	min 2996, median 2996, max 2996	
Wallemia ichthyophaga	0	-	
Wallemia mellicola	0	-	
Xylona heveae	0	-	
Saitoella complicata	2	min 912, median 912, max 1379	
Lobosporangium transversale	17	min 485, median 2977, max 3134	
Phycomyces blakesleeanus	146	min 182, median 2471, max 3203	
Rhizopus microsporus	35	min 317, median 1229, max 2936	
Encephalitozoon cuniculi	0	-	
Encephalitozoon hellem	0	-	
Encephalitozoon intestinalis	0	-	
Encephalitozoon romaleae	0	-	
Enterocytozoon bieneusi	0	-	
Mitosporidium daphniae	0	-	
Nematocida parisii	0	-	
Nosema ceranae	2	min 218, median 218, max 694	
Ordospora colligata	0	-	
Vavraia culicis	0	-	
Vittaforma corneae	0	-	
**VIRIDIPLANTAE**			
Micromonas pusilla CCMP1545	0	-	
Micromonas sp. RCC299	0	-	
Ostreococcus lucimarinus CCE9901	0	-	
Ostreococcus tauri	0	-	
Chlamydomonas reinhardtii	675	min 56, median 719, max 6259	
Volvox carteri f. nagariensis	297	min 56, median 1013, max 8376	
Chlorella variabilis	14	min 72, median 788, max 2753	
Auxenochlorella protothecoides	0	-	
Helicosporidium sp. ATCC 50920	0	-	
Coccomyxa subellipsoidea C-169	298	min 44, median 286, max 8994	
Klebsormidium flaccidum	8	min 521, median 1571, max 3187	
Physcomitrella patens	275	min 82, median 707, max 3257	
Selaginella moellendorffii	221	min 82, median 1180, max 17033	
Pinus taeda	1744	min 44, median 1130, max 5806	
Amborella trichopoda	8912	min 32, median 701, max 7437	

Species	# L1 hits	Length distribution (bp)	Notes
Spirodela polyrhiza	135	min 53, median 1130, max 4144	
Phoenix dactylifera	3589	min 35, median 916, max 6802	
Elaeis oleifera	4151	min 35, median 772, max 4405	
Ensete ventricosum	55	min 50, median 716, max 2405	
Musa acuminata subsp. malaccensis	724	min 68, median 964, max 3524	
Sorghum bicolor	4272	min 35, median 801, max 8289	
Zea mays	6618	min 32, median 870, max 7283	
Setaria italica	2918	min 41, median 953, max 7323	
Brachypodium distachyon	3074	min 35, median 893, max 7282	
Leersia perrieri	1954	min 50, median 762, max 6583	
Oryza barthii	1610	min 32, median 800, max 7342	
Oryza brachyantha	485	min 47, median 698, max 5902	
Oryza glumipatula	1552	min 38, median 807, max 7736	
Oryza longistaminata	1301	min 38, median 806, max 7116	
Oryza meridionalis	1448	min 47, median 758, max 7197	
Oryza nivara	1617	min 38, median 830, max 7342	
Oryza punctata	1504	min 38, median 812, max 7473	
Oryza sativa Japonica Group	1700	min 38, median 925, max 7409	
Zizania latifolia	1564	min 53, median 1016, max 5345	
Aegilops tauschii	11027	min 35, median 1767, max 7683	
Triticum urartu	10977	min 44, median 1778, max 8407	
Nelumbo nucifera	19318	min 29, median 614, max 7648	
Lupinus angustifolius	2027	min 41, median 1151, max 5768	
Phaseolus vulgaris	4448	min 35, median 881, max 5861	
Cajanus cajan	1228	min 50, median 1048, max 5476	
Vigna angularis var. angularis	144	min 89, median 349, max 2875	
Vigna radiata var. radiata	223	min 47, median 330, max 3013	
Glycine max	5640	min 41, median 758, max 7151	
Glycine soja	3923	min 41, median 827, max 11768	
Cicer arietinum	1325	min 41, median 419, max 5029	
Medicago truncatula	2829	min 41, median 617, max 7369	
Trifolium pratense	1968	min 65, median 511, max 5762	
Lotus japonicus	2	min 50, median 1071, max 4150	
Malus x domestica	1729	min 56, median 779, max 6604	
Pyrus x bretschneideri	1729	min 41, median 784, max 6922	
Prunus mume	912	min 50, median 704, max 5952	
Prunus persica	797	min 53, median 710, max 5889	
Fragaria iinumae	1019	min 41, median 589, max 6012	
Fragaria nubicola	1168	min 68, median 528, max 4773	
Fragaria orientalis	1446	min 72, median 470, max 5421	
Fragaria vesca subsp. vesca	1241	min 44, median 663, max 6199	
Fragaria x ananassa	929	min 41, median 380, max 6013	

Species	# L1 hits	Length distribution (bp)	Notes
Morus notabilis	980	min 41, median 295, max 5400	
Cannabis sativa	4612	min 38, median 1254, max 5289	
Castanea mollissima	4957	min 35, median 1287, max 4424	
Betula nana	3589	min 50, median 710, max 5054	
Cucumis melo	1118	min 32, median 777, max 3963	
Cucumis sativus	880	min 50, median 708, max 3316	
Citrullus lanatus	1116	min 47, median 785, max 5049	
Lagenaria siceraria	1019	min 59, median 383, max 2613	
Populus euphratica	1127	min 56, median 923, max 6319	
Populus trichocarpa	1547	min 41, median 754, max 6523	
Jatropha curcas	1905	min 32, median 661, max 5478	
Manihot esculenta subsp. flabellifolia	1690	min 44, median 755, max 5606	
Ricinus communis	843	min 62, median 591, max 3924	
Linum usitatissimum	2158	min 44, median 759, max 5346	
Eucalyptus camaldulensis	4127	min 65, median 976, max 6573	
Eucalyptus grandis	5885	min 35, median 1058, max 7266	
Carica papaya	2451	min 47, median 667, max 6410	
Arabidopsis halleri subsp. gemmifera	3059	min 68, median 157, max 7434	
Arabidopsis lyrata subsp. lyrata	1740	min 65, median 1274, max 6932	
Arabidopsis thaliana	542	min 35, median 926, max 6834	
Camelina sativa	8025	min 47, median 1175, max 7902	
Capsella rubella	654	min 41, median 1131, max 6249	
Brassica napus	9753	min 44, median 1580, max 8479	
Brassica oleracea var. oleracea	6991	min 41, median 1040, max 6121	
Brassica rapa	3425	min 44, median 848, max 6125	
Raphanus raphanistrum subsp. raphanistrum	1728	min 76, median 2107, max 5996	
Raphanus sativus	3064	min 53, median 1787, max 6103	
Aethionema arabicum	1308	min 70, median 1628, max 5667	
Arabis alpina	3703	min 44, median 1184, max 6298	
Eutrema parvulum	787	min 47, median 952, max 6073	
Eutrema salsugineum	1892	min 41, median 1502, max 6363	
Sisymbrium irio	1961	min 59, median 1081, max 6116	
Leavenworthia alabamica	739	min 56, median 1139, max 5965	
Tarenaya hassleriana	620	min 62, median 1094, max 4380	
Gossypium arboreum	3758	min 44, median 1038, max 5838	
Gossypium raimondii	5445	min 35, median 730, max 5724	
Theobroma cacao	2190	min 35, median 700, max 6934	
Aquilaria agallochum	2814	min 44, median 725, max 4272	
Azadirachta indica	489	min 47, median 561, max 5194	
Citrus clementine	1771	min 41, median 899, max 5669	
Citrus sinensis	2011	min 44, median 887, max 5712	

Species	# L1 hits	Length distribution (bp)	Notes
Vitis vinifera	6108	min 29, median 1028, max 6808	
Amaranthus hypochondriacus	2669	min 74, median 525, max 4869	
Amaranthus tuberculatus	184	min 62, median 231, max 1352	
Beta vulgaris subsp. vulgaris	9915	min 41, median 989, max 6948	
Spinacia oleracea	2817	min 50, median 1365, max 6337	
Dianthus caryophyllus	2072	min 68, median 1925, max 4459	
Actinidia chinensis	1052	min 47, median 913, max 5954	
Vaccinium macrocarpon	1861	min 47, median 812, max 4536	
Diospyros lotus	2	min 1382, median 1382, max 1950	
Primula veris	978	min 47, median 857, max 4168	
Solanum arcanum	2517	min 62, median 1360, max 16602	
Solanum habrochaites	2891	min 59, median 1273, max 14525	
Solanum lycopersicum	3046	min 41, median 1014, max 28999	
Solanum melongena	3223	min 50, median 1173, max 10878	
Solanum pennellii	2648	min 38, median 1241, max 16789	
Solanum pimpinellifolium	4046	min 43, median 785, max 16052	
Solanum tuberosum	4713	min 44, median 878, max 16344	
Capsicum annuum	10918	min 35, median 1305, max 6394	
Nicotiana sylvestris	6487	min 53, median 1724, max 6385	
Nicotiana tomentosiformis	4987	min 41, median 1597, max 7374	
Fraxinus excelsior	1798	min 41, median 1215, max 4845	
Penstemon centranthifolius	38	min 50, median 516, max 2734	
Penstemon grinnellii	23	min 203, median 596, max 1456	
Sesamum indicum	3571	min 35, median 902, max 4635	
Genlisea aurea	86	min 121, median 1097, max 4055	
Mimulus guttatus	1304	min 65, median 1112, max 5583	
Conyza canadensis	952	min 62, median 1526, max 4530	

Table S3: BovBs in genomic data

Table S3: Shows which genomes contain BovBs (72/759) and the number of hits and length distributions. As before, BovB elements were detected with protein based searches using TBLASTN and nucleotide similarity searches using LASTZ. All hits had to satisfy a 'reciprocal best hit' check to check they were really BovBs (and not other repeats). Overlapping hits were merged to produce a non-redundant set of BovBs for each genome.

Species	# L1 hits	Length distribution (bp)	Notes
**METAZOA**			
**MAMMALIA**			
Tachyglossus aculeatus	3196	min 29, median 371, max 3794	
Ornithorhynchus anatinus	3720	min 26, median 393, max 3849	
Monodelphis domestica	6340	min 29, median 353, max 3511	
Macropus eugenii	2302	min 32, median 503, max 3497	
Sarcophilus harrisii	4948	min 29, median 410, max 3788	
Dasypus novemcinctus	0	-	
Choloepus hoffmanni	0	-	
Chrysochloris asiatica	240967	min 29, median 296, max 6242	
Echinops telfairi	54734	min 29, median 275, max 3603	
Orycteropus afer afer	155026	min 26, median 362, max 8015	
Elephantulus edwardii	392850	min 26, median 329, max 5564	
Trichechus manatus latirostris	47995	min 29, median 326, max 6541	
Procavia capensis	16229	min 41, median 1049, max 5482	
Loxodonta africana	65259	min 26, median 289, max 6341	
Erinaceus europaeus	0	-	
Sorex araneus	0	-	
Condylura cristata	0	-	
Pteropus alecto	112	min 50, median 293, max 3182	
Pteropus vampyrus	83	min 38, median 279, max 3200	
Eidolon helvum	93	min 38, median 338, max 3195	
Megaderma lyra	79	min 41, median 272, max 3092	
Rhinolophus ferrumequinum	101	min 53, median 314, max 3469	
Pteronotus parnellii	75	min 74, median 278, max 3363	
Eptesicus fuscus	88	min 35, median 287, max 3554	
Myotis brandtii	116	min 47, median 268, max 3373	
Myotis davidii	90	min 41, median 320, max 3615	
Myotis lucifugus	100	min 53, median 345, max 3917	
Ceratotherium simum simum	389	min 38, median 547, max 3668	
Equus przewalskii	283	min 46, median 649, max 3737	
Equus caballus (Thoroughbred)	343	min 44, median 359, max 3751	
Equus caballus (Mongolian)	302	min 56, median 655, max 3750	
Manis pentadactyla	0	-	

**Species**	**# L1 hits**	**Length distribution (bp)**	**Notes**
Felis catus	0	-	
Panthera tigris altaica	0	-	
Canis lupus familiaris	0	-	
Ursus maritimus	0	-	
Ailuropoda melanoleuca	0	-	
Leptonychotes weddellii	0	-	
Odobenus rosmarus divergens	0	-	
Mustela putorius furo	0	-	
Camelus dromedarius	0	-	
Camelus ferus	0	-	
Vicugna pacos	0	-	
Sus scrofa (Duroc)	0	-	
Sus scrofa (Tibetan)	0	-	
Sus scrofa (Ellegaard Gottingen minipig)	0	-	
Balaenoptera acutorostrata scammoni	0	-	
Physeter catodon	0	-	
Lipotes vexillifer	0	-	
Tursiops truncatus	0	-	
Orcinus orca	0	-	
Pantholops hodgsonii	335036	min 26, median 437, max 6825	
Capra hircus	367621	min 26, median 405, max 7217	
Ovis aries (Texel)	360487	min 26, median 486, max 7170	
Ovis aries musimon	266125	min 26, median 518, max 7181	
Bubalus bubalis	360889	min 26, median 516, max 7930	
Bison bison bison	393414	min 26, median 498, max 8305	
Bos mutus	304207	min 26, median 500, max 8654	
Bos indicus	310238	min 26, median 609, max 8107	
Bos taurus	334418	min 26, median 608, max 8639	
Ochotona princeps	0	-	
Oryctolagus cuniculus	0	-	
Ictidomys tridecemlineatus	0	-	
Heterocephalus glaber	0	-	
Fukomys damarensis	0	-	
Cavia aperea	0	-	
Cavia porcellus	0	-	
Chinchilla lanigera	0	-	
Octodon degus	0	-	
Dipodomys ordii	0	-	
Jaculus jaculus	0	-	
Nannospalax galili	0	-	
Mesocricetus auratus	0	-	
Cricetulus griseus	0	-	

Species	# L1 hits	Length distribution (bp)	Notes
Microtus ochrogaster	0	-	
Peromyscus maniculatus bairdii	0	-	
Rattus norvegicus	0	-	
Mus musculus	0	-	
Tupaia belangeri	0	-	
Tupaia chinensis	0	-	
Galeopterus variegatus	0	-	
Otolemur garnettii	0	-	
Microcebus murinus	0	-	
Tarsius syrichta	0	-	
Callithrix jacchus	0	-	
Saimiri boliviensis boliviensis	0	-	
Rhinopithecus roxellana	0	-	
Nasalis larvatus	0	-	
Chlorocebus sabaeus	0	-	
Macaca fascicularis	0	-	
Macaca mulatta	0	-	
Papio anubis	0	-	
Nomascus leucogenys	0	-	
Pongo abelii	0	-	
Gorilla gorilla gorilla	0	-	
Pan paniscus	0	-	
Pan troglodytes	0	-	
Homo sapiens	0	-	
**SAUROPSIDA**			
Apalone spinifera	0	-	Contains RTEs
Pelodiscus sinensis	0	-	Contains RTEs
Chelonia mydas	0	-	Contains RTEs
Chrysemys picta bellii	0	-	Contains RTEs
Struthio camelus australis	0	-	
Tinamus guttatus	0	-	
Anas platyrhynchos	0	-	
Lyrurus tetrix tetrix	0	-	
Gallus gallus	0	-	
Coturnix japonica	0	-	
Meleagris gallopavo	0	-	
Colinus virginianus	0	-	
Acanthisitta chloris	0	-	
Manacus vitellinus	0	-	
Zonotrichia albicollis	0	-	
Geospiza fortis	0	-	
Serinus canaria	0	-	
Taeniopygia guttata	0	-	
Ficedula albicollis	0	-	

**Species**	**# L1 hits**	**Length distribution (bp)**	**Notes**
Pseudopodoces humilis	0	-	
Corvus brachyrhynchos	0	-	
Corvus cornix cornix	0	-	
Ara macao	0	-	
Amazona vittata	0	-	
Melopsittacus undulatus	0	-	
Nestor notabilis	0	-	
Falco cherrug	0	-	
Falco peregrinus	0	-	
Cariama cristata	0	-	
Merops nubicus	0	-	
Picoides pubescens	0	-	
Buceros rhinoceros silvestris	0	-	
Apaloderma vittatum	0	-	
Leptosomus discolour	0	-	
Haliaeetus albicilla	0	-	
Haliaeetus leucocephalus	0	-	
Aquila chrysaetos canadensis	0	-	
Cathartes aura	0	-	
Tyto alba	0	-	
Colius striatus	0	-	
Charadrius vociferus	0	-	
Balearica regulorum gibbericeps	0	-	
Chlamydotis macqueenii	0	-	
Cuculus canorus	0	-	
Fulmarus glacialis	0	-	
Aptenodytes forsteri	0	-	
Pygoscelis adeliae	0	-	
Phalacrocorax carbo	0	-	
Pelecanus crispus	0	-	
Nipponia nippon	0	-	
Egretta garzetta	0	-	
Phaethon lepturus	0	-	
Gavia stellata	0	-	
Tauraco erythrolophus	0	-	
Opisthocomus hoazin	0	-	
Columba livia	0	-	
Pterocles gutturalis	0	-	
Calypte anna	0	-	
Chaetura pelagica	0	-	
Caprimulgus carolinensis	0	-	
Eurypyga helias	0	-	
Mesitornis unicolor	0	-	

Species	# L1 hits	Length distribution (bp)	Notes
Podiceps cristatus	0	-	
Phoenicopterus ruber ruber	0	-	
Alligator mississippiensis	0	-	Contains RTEs
Alligator sinensis	0	-	Contains RTEs
Crocodylus porosus	0	-	Contains RTEs
Gavialis gangeticus	0	-	Contains RTEs
Pogona vitticeps	63989	min 26, median 399, max 3821	
Anolis carolinensis	20144	min 29, median 386, max 3562	
Vipera berus berus	8885	min 32, median 394, max 3490	
Crotalus mitchellii pyrrhus	1540	min 35, median 371, max 3360	
Ophiophagus hannah	2858	min 29, median 344, max 3667	
Python bivittatus	6503	min 26, median 384, max 3793	
**AMPHIBIA**			
Nanorana parkeri	99	min 47, median 322, max 1766	
Xenopus laevis	81	min 71, median 278, max 2303	
Xenopus tropicalis	69	min 74, median 281, max 2623	
**NEOPTERYGII**			
Lepisosteus oculatus	102	min 47, median 525, max 3079	
Anguilla anguilla	0	-	
Anguilla japonica	0	-	
Danio rerio	30	min 164, median 608, max 3079	
Astyanax mexicanus	0	-	
Oryzias latipes	0	-	
Poecilia formosa	0	-	
Xiphophorus maculatus	0	-	
Fundulus heteroclitus	0	-	
Takifugu flavidus	0	-	
Takifugu rubripes	0	-	
Tetraodon nigroviridis	0	-	
Cynoglossus semilaevis	6	min 292, median 476, max 2463	
Haplochromis burtoni	0	-	
Pundamilia nyererei	0	-	
Maylandia zebra	0	-	
Neolamprologus brichardi	0	-	
Oreochromis niloticus	11	min 185, median 1066, max 2829	
Sebastes nigrocinctus	0	-	
Sebastes rubrivinctus	0	-	
Gasterosteus aculeatus	0	-	
Gadus morhua	0	-	
**CHONDRICHTHYES**			
Callorhinchus milii	0	-	
Carcharhinus brachyurus	0	-	
**ECDYSOZOA**			
Ephemera danica	4	min 905, median 1259, max 2744	

Species	# L1 hits	Length distribution (bp)	Notes
Ladona fulva	0	-	
Pediculus humanus corporis	0	-	
Frankliniella occidentalis	0	-	
Diaphorina citri	38	min 131, median 623, max 3089	
Pachypsylla venusta	0	-	
Acyrthosiphon pisum	0	-	
Nilaparvata lugens	93	min 182, median 1427, max 2906	
Oncopeltus fasciatus	0	-	
Rhodnius prolixus	0	-	
Cimex lectularius	176	min 38, median 400, max 2625	
Onthophagus taurus	0	-	
Agrilus planipennis	0	-	
Tribolium castaneum	0	-	
Anoplophora glabripennis	0	-	
Leptinotarsa decemlineata	0	-	
Dendroctonus ponderosae	0	-	
Mengenilla moldrzyki	0	-	
Aedes aegypti	0	-	
Culex quinquefasciatus	0	-	
Anopheles albimanus	0	-	
Anopheles arabiensis	0	-	
Anopheles atroparvus	0	-	
Anopheles christyi	0	-	
Anopheles culicifacies	0	-	
Anopheles darlingi	0	-	
Anopheles dirus	0	-	
Anopheles epiroticus	0	-	
Anopheles farauti	0	-	
Anopheles funestus	0	-	
Anopheles gambiae	0	-	
Anopheles maculatus	0	-	
Anopheles melas	0	-	
Anopheles merus	0	-	
Anopheles minimus	0	-	
Anopheles quadriannulatus	0	-	
Anopheles sinensis	0	-	
Anopheles stephensi	0	-	
Mayetiola destructor	0	-	
Lutzomyia longipalpis	0	-	
Phlebotomus papatasi	0	-	
Ceratitis capitata	0	-	
Drosophila albomicans	0	-	
Drosophila ananassae	0	-	
Drosophila biarmipes	0	-	

**Species**	**# L1 hits**	**Length distribution (bp)**	**Notes**
Drosophila bipectinata	0	-	
Drosophila elegans	0	-	
Drosophila erecta	0	-	
Drosophila eugracilis	0	-	
Drosophila ficusphila	0	-	
Drosophila grimshawi	0	-	
Drosophila kikkawai	0	-	
Drosophila melanogaster	0	-	
Drosophila miranda	0	-	
Drosophila mojavensis	0	-	
Drosophila persimilis	0	-	
Drosophila pseudoobscura pseudoobscura	0	-	
Drosophila rhopaloa	0	-	
Drosophila sechellia	0	-	
Drosophila simulans	0	-	
Drosophila suzukii	0	-	
Drosophila takahashii	0	-	
Drosophila virilis	0	-	
Drosophila willistoni	0	-	
Drosophila yakuba	0	-	
Musca domestica	0	-	
Glossina austeni	0	-	
Glossina brevipalpis	0	-	
Glossina fuscipes fuscipes	0	-	
Glossina morsitans morsitans	0	-	
Glossina pallidipes	0	-	
Limnephilus lunatus	0	-	
Papilio glaucus	320	min 77, median 479, max 2981	
Papilio polytes	0	-	Contains RTEs
Papilio xuthus	0	-	Contains RTEs
Heliconius melpomene melpomene	4	min 2124, median 2775, max 2872	
Melitaea cinxia	0	-	
Danaus plexippus	33	min 62, median 470, max 2705	
Bombyx mori	883	min 44, median 455, max 2995	
Manduca sexta	42	min 143, median 553, max 3042	
Plutella xylostella	19	min 368, median 902, max 3194	
Athalia rosae	0	-	
Cephus cinctus	0	-	
Orussus abietinus	0	-	
Ceratosolen solmsi marchali	0	-	
Nasonia giraulti	0	-	
Nasonia longicornis	0	-	

**Species**	**# L1 hits**	**Length distribution (bp)**	**Notes**
Nasonia vitripennis	0	-	
Copidosoma floridanum	0	-	
Trichogramma pretiosum	0	-	
Microplitis demolitor	0	-	
Megachile rotundata	0	-	
Apis dorsata	0	-	
Apis florea	0	-	
Apis mellifera	0	-	
Bombus impatiens	0	-	
Bombus terrestris	0	-	
Linepithema humile	6	min 92, median 320, max 2960	
Camponotus floridanus	0	-	
Acromyrmex echinatior	0	-	
Atta cephalotes	0	-	
Solenopsis invicta	30	min 209, median 549, max 3090	
Pogonomyrmex barbatus	5	min 59, median 422, max 2948	
Harpegnathos saltator	0	-	
Cerapachys biroi	0	-	
Blattella germanica	0	-	
Zootermopsis nevadensis	0	-	
Locusta migratoria	2724	min 26, median 659, max 3258	
Daphnia pulex	0	-	
Eurytemora affinis	0	-	
Lepeophtheirus salmonis	0	-	
Hyalella azteca	0	-	
Strigamia maritima	0	-	
Stegodyphus mimosarum	0	-	
Latrodectus hesperus	0	-	
Parasteatoda tepidariorum	0	-	
Tetranychus urticae	0	-	
Dermatophagoides farinae	0	-	
Sarcoptes scabiei type canis	0	-	
Achipteria coleoptrata	0	-	
Hypochthonius rufulus	0	-	
Platynothrus peltifer	0	-	
Steganacarus magnus	0	-	
Ixodes ricinus	0	-	
Ixodes scapularis	0	-	
Rhipicephalus microplus	0	-	
Metaseiulus occidentalis	0	-	
Varroa destructor	0	-	
Centruroides exilicauda	213	min 59, median 642, max 3251	
Mesobuthus martensii	443	min 59, median 874, max 3737	
Limulus polyphemus	0	-	
Trichinella spiralis	0	-	

Species	# L1 hits	Length distribution (bp)	Notes
Ascaris suum	0	-	
Elaeophora elaphi	0	-	
Onchocerca volvulus	0	-	
Steinernema monticolum	0	-	
Panagrellus redivivus	0	-	
Haemonchus contortus	0	-	
Necator americanus	0	-	
Heterorhabditis bacteriophora	0	-	
Caenorhabditis angaria	0	-	
Caenorhabditis brenneri	0	-	
Caenorhabditis briggsae	0	-	
Caenorhabditis elegans	0	-	
Caenorhabditis japonica	0	-	
Caenorhabditis sp. 11 MAF-2010	0	-	
Priapulus caudatus	0	-	
**ROTIFERA**			
Adineta vaga	26	min 110, median 1160, max 3077	
**PLATYHELMINTHES**			
Schistosoma curassoni	0	-	
Schistosoma haematobium	0	-	
Schistosoma japonicum	0	-	
Schistosoma mansoni	0	-	
Schistosoma margrebowiei	0	-	
Schistosoma mattheei	0	-	
Schistosoma rodhaini	0	-	
Clonorchis sinensis	0	-	
Echinococcus granulosus	0	-	
Echinococcus multilocularis	0	-	
Hymenolepis microstoma	0	-	
Protopolystoma xenopodis	0	-	
**ANNELIDA**			
Capitella teleta	19	min 134, median 329, max 1317	
Helobdella robusta	136	min 59, median 541, max 3005	
**MOLLUSCA**			
Crassostrea gigas	0	-	
Lottia gigantea	0	-	
Aplysia californica	0	-	
Biomphalaria glabrata	0	-	
**CNIDARIA**			
Nematostella vectensis	0	-	
Hydra vulgaris	0	-	
**TENTACULATA**			
Mnemiopsis leidyi	0	-	

Species	# L1 hits	Length distribution (bp)	Notes
**PLACOZOA**			
Trichoplax adhaerens	0	-	
**ECHINOIDEA**			
Amphimedon queenslandica	0	-	
Lytechinus variegatus	8	min 95, median 474, max 970	
Strongylocentrotus purpuratus	70	min 38, median 470, max 3112	
**ASTEROIDEA**			
Patiria miniata	0	-	
**ENTEROPNEUSTA**			
Saccoglossus kowalevskii	0	-	
**TUNICATA**			
Ciona intestinalis	0	-	
Ciona savignyi	19	min 155, median 799, max 2852	RTE-like BovBs
Botryllus schlosseri	0	-	
Oikopleura dioica	0	-	
**LEPTOCARDII**			
Branchiostoma floridae	0	-	
**CEPHALASPIDOMORPHI**			
Lethenteron camtschaticum	31	min 200, median 731, max 2279	
Petromyzon marinus	0	-	Contains RTEs
**SARCOPTERYGII**			
Latimeria chalumnae	166	min 41, median 511, max 2919	
**FUNGI**			
Agaricus bisporus var. bisporus	0	-	
Agaricus bisporus var. burnettii	0	-	
Schizophyllum commune	0	-	
Coprinopsis cinerea	0	-	
Moniliophthora roreri	0	-	
Laccaria bicolor	0	-	
Auricularia subglabra	0	-	
Coniophora puteana	0	-	
Serpula lacrymans	0	-	
Punctularia strigosozonata	0	-	
Gloeophyllum trabeum	0	-	
Fomitiporia mediterranea	0	-	
Dichomitus squalens	0	-	
Fibroporia radiculosa	0	-	
Phanerochaete carnosa	0	-	
Postia placenta	0	-	
Trametes versicolor	0	-	

**Species**	**# L1 hits**	**Length distribution (bp)**	**Notes**
Heterobasidion irregulare	0	-	
Stereum hirsutum	0	-	
Spizellomyces punctatus	0	-	
Batrachochytrium dendrobatidis	0	-	
Diplodia corticola	0	-	
Neofusicoccum parvum	0	-	
Baudoinia panamericana	0	-	
Cercospora beticola	0	-	
Pseudocercospora fijiensis	0	-	
Sphaerulina musiva	0	-	
Zymoseptoria tritici	0	-	
Aureobasidium namibiae	0	-	
Aureobasidium subglaciale	0	-	
Coniosporium apollinis	0	-	
Paraphaeosphaeria sporulosa	0	-	
Parastagonospora nodorum	0	-	
Leptosphaeria maculans	0	-	
Alternaria alternata	0	-	
Bipolaris maydis	0	-	
Bipolaris oryzae	0	-	
Bipolaris sorokiniana	0	-	
Bipolaris victoriae	0	-	
Bipolaris zeicola	0	-	
Pyrenophora teres	0	-	
Pyrenophora tritici-repentis	0	-	
Setosphaeria turcica	0	-	
Verruconis gallopava	0	-	
Cyphellophora europaea	0	-	
Capronia coronata	0	-	
Capronia epimyces	0	-	
Cladophialophora bantiana	0	-	
Cladophialophora carrionii	0	-	
Cladophialophora immunda	0	-	
Cladophialophora psammophila	0	-	
Cladophialophora yegresii	0	-	
Exophiala aquamarina	0	-	
Exophiala dermatitidis	0	-	
Exophiala mesophila	0	-	

Species	# L1 hits	Length distribution (bp)	Notes
Exophiala oligosperma	0	-	
Exophiala spinifera	0	-	
Exophiala xenobiotica	0	-	
Fonsecaea erecta	0	-	
Fonsecaea monophora	0	-	
Fonsecaea multimorphosa	0	-	
Fonsecaea nubica	0	-	
Fonsecaea pedrosoi	0	-	
Phialophora attae	0	-	
Rhinocladiella mackenziei	0	-	
Aspergillus aculeatus	0	-	
Aspergillus bombycis	0	-	
Aspergillus clavatus	0	-	
Aspergillus fischeri	0	-	
Aspergillus flavus	0	-	
Aspergillus fumigatus	0	-	
Aspergillus glaucus	0	-	
Aspergillus nidulans	0	-	
Aspergillus niger	0	-	
Aspergillus nomius	0	-	
Aspergillus oryzae	0	-	
Aspergillus terreus	0	-	
Penicilliopsis zonata	0	-	
Penicillium arizonense	0	-	
Penicillium chrysogenum	0	-	
Penicillium digitatum	0	-	
Penicillium expansum	0	-	
Penicillium marneffei	0	-	
Rasamsonia emersonii	0	-	
Talaromyces atroroseus	0	-	
Talaromyces stipitatus	0	-	
Ajellomyces dermatitidis	0	-	
Arthroderma otae	0	-	
Coccidioides immitis	0	-	
Coccidioides posadasii	0	-	
Histoplasma capsulatum	0	-	
Nannizzia gypsea	0	-	
Paracoccidioides brasiliensis	0	-	
Paracoccidioides lutzii	0	-	
Trichophyton benhamiae	0	-	
Trichophyton rubrum	0	-	
Trichophyton verrucosum	0	-	
Uncinocarpus reesii	0	-	
Endocarpon pusillum	0	-	
Tilletiaria anomala	0	-	

**Species**	**# L1 hits**	**Length distribution (bp)**	**Notes**
Botryotinia fuckeliana	0	-	
Glarea lozoyensis	0	-	
Marssonina brunnea	0	-	
Phialocephala scopiformis	0	-	
Sclerotinia sclerotiorum	0	-	
Pseudogymnoascus destructans	0	-	
Pseudogymnoascus verrucosus	0	-	
Malassezia globosa	0	-	
Malassezia pachydermatis	0	-	
Malassezia sympodialis	0	-	
Rhodotorula graminis	0	-	
Rhodotorula toruloides	0	-	
Mixia osmundae	0	-	
Arthrobotrys oligospora	0	-	
Dactylellina haptotyla	0	-	
Tuber melanosporum	0	-	
Pneumocystis carinii	0	-	
Pneumocystis jirovecii	0	-	
Pneumocystis murina	0	-	
Melampsora larici-populina	0	-	
Puccinia graminis	0	-	
Candida albicans	0	-	
Candida auris	0	-	
Candida dubliniensis	0	-	
Candida glabrata	0	-	
Candida orthopsilosis	0	-	
Candida tanzawaensis	0	-	
Candida tenuis	0	-	
Candida tropicalis	0	-	
Lodderomyces elongisporus	0	-	
Ascoidea rubescens	0	-	
Babjeviella inositovora	0	-	
Clavispora lusitaniae	0	-	
Cyberlindnera jadinii	0	-	
Debaryomyces fabryi	0	-	
Debaryomyces hansenii	0	-	
Eremothecium cymbalariae	0	-	
Eremothecium gossypii	0	-	
Eremothecium sinecaudum	0	-	
Hyphopichia burtonii	0	-	

Species	# L1 hits	Length distribution (bp)	Notes
Kazachstania africana	0	-	
Kazachstania naganishii	0	-	
Kluyveromyces lactis	0	-	
Kluyveromyces marxianus	0	-	
Komagataella phaffii	0	-	
Kuraishia capsulata	0	-	
Lachancea lanzarotensis	0	-	
Lachancea thermotolerans	0	-	
Metschnikowia bicuspidata	0	-	
Meyerozyma guilliermondii	0	-	
Naumovozyma castellii	0	-	
Naumovozyma dairenensis	0	-	
Ogataea parapolymorpha	0	-	
Ogataea polymorpha	0	-	
Saccharomyces cerevisiae	0	-	
Saccharomyces eubayanus	0	-	
Spathaspora passalidarum	0	-	
Sugiyamaella lignohabitans	0	-	
Tetrapisispora blattae	0	-	
Tetrapisispora phaffii	0	-	
Torulaspora delbrueckii	0	-	
Vanderwaltozyma polyspora	0	-	
Yarrowia lipolytica	0	-	
Zygosaccharomyces rouxii	0	-	
Wickerhamomyces anomalus	0	-	
Wickerhamomyces ciferrii	0	-	
Scheffersomyces stipitis	0	-	
Pichia kudriavzevii	0	-	
Pichia membranifaciens	0	-	
Schizosaccharomyces cryophilus	0	-	
Schizosaccharomyces japonicus	0	-	
Schizosaccharomyces octosporus	0	-	
Schizosaccharomyces pombe	0	-	
Metarhizium acridum	0	-	
Metarhizium brunneum	0	-	
Metarhizium majus	0	-	
Metarhizium robertsii	0	-	

Species	# L1 hits	Length distribution (bp)	Notes
Pochonia chlamydosporia	0	-	
Beauveria bassiana	0	-	
Cordyceps militaris	0	-	
Isaria fumosorosea	0	-	
Trichoderma atroviride	0	-	
Trichoderma gamsii	0	-	
Trichoderma reesei	0	-	
Trichoderma virens	0	-	
Fusarium fujikuroi	0	-	
Fusarium graminearum	0	-	
Fusarium oxysporum	0	-	
Fusarium pseudograminearum	0	-	
Fusarium verticillioides	0	-	
Nectria haematococca	0	-	
Purpureocillium lilacinum	0	-	
Colletotrichum fioriniae	0	-	
Colletotrichum gloeosporioides	0	-	
Colletotrichum graminicola	0	-	
Colletotrichum higginsianum	0	-	
Colletotrichum orchidophilum	0	-	
Verticillium alfalfae	0	-	
Verticillium dahliae	0	-	
Magnaporthe oryzae	0	-	
Gaeumannomyces tritici	0	-	
Scedosporium apiospermum	0	-	
Grosmannia clavigera	0	-	
Sporothrix schenckii	0	-	
Chaetomium globosum	0	-	
Chaetomium thermophilum	0	-	
Neurospora crassa	0	-	
Neurospora tetrasperma	0	-	
Podospora anserina	0	-	
Sordaria macrospora	0	-	
Thermothelomyces thermophila	0	-	
Thielavia terrestris	0	-	
Phaeoacremonium minimum	0	-	
Eutypa lata	0	-	

Species	# L1 hits	Length distribution (bp)	Notes
Pestalotiopsis fici	0	-	
Cryptococcus amylolentus	0	-	
Cryptococcus gattii	0	-	
Cryptococcus neoformans var. grubii	0	-	
Cryptococcus neoformans var. neoformans JEC21	0	-	
Cryptococcus neoformans var. neoformans B-3501A	0	-	
Kockovaella imperatae	0	-	
Kwoniella bestiolae	0	-	
Kwoniella dejecticola	0	-	
Kwoniella mangroviensis	0	-	
Kwoniella pini	0	-	
Tremella mesenterica	0	-	
Tsuchiyaea wingfieldii	0	-	
Cutaneotrichosporon oleaginosus	0	-	
Trichosporon asahii	0	-	
Anthracocystis flocculosa	0	-	
Kalmanozyma brasiliensis	0	-	
Moesziomyces antarcticus	0	-	
Pseudozyma hubeiensis	0	-	
Ustilago maydis	0	-	
Wallemia ichthyophaga	0	-	
Wallemia mellicola	0	-	
Xylona heveae	0	-	
Saitoella complicata	0	-	
Lobosporangium transversale	0	-	
Phycomyces blakesleeanus	0	-	
Rhizopus microsporus	0	-	
Encephalitozoon cuniculi	0	-	
Encephalitozoon hellem	0	-	
Encephalitozoon intestinalis	0	-	
Encephalitozoon romaleae	0	-	
Enterocytozoon bieneusi	0	-	
Mitosporidium daphniae	0	-	
Nematocida parisii	0	-	
Nosema ceranae	0	-	
Ordospora colligata	0	-	
Vavraia culicis	0	-	
Vittaforma corneae	0	-	
**VIRIDIPLANTAE**			

Species	# L1 hits	Length distribution (bp)	Notes
Micromonas pusilla CCMP1545	0	-	
Micromonas sp. RCC299	0	-	
Ostreococcus lucimarinus CCE9901	0	-	
Ostreococcus tauri	0	-	
Chlamydomonas reinhardtii	0	-	
Volvox carteri f. nagariensis	0	-	
Chlorella variabilis	0	-	
Auxenochlorella protothecoides	0	-	
Helicosporidium sp. ATCC 50920	0	-	
Coccomyxa subellipsoidea C-169	0	-	
Klebsormidium flaccidum	0	-	
Physcomitrella patens	0	-	
Selaginella moellendorffii	0	-	
Pinus taeda	0	-	
Amborella trichopoda	0	-	
Spirodela polyrhiza	0	-	
Phoenix dactylifera	0	-	
Elaeis oleifera	0	-	
Ensete ventricosum	0	-	
Musa acuminata subsp. malaccensis	0	-	
Sorghum bicolor	0	-	
Zea mays	0	-	
Setaria italica	0	-	
Brachypodium distachyon	0	-	
Leersia perrieri	0	-	
Oryza barthii	0	-	
Oryza brachyantha	0	-	
Oryza glumipatula	0	-	
Oryza longistaminata	0	-	
Oryza meridionalis	0	-	
Oryza nivara	0	-	
Oryza punctata	0	-	
Oryza sativa Japonica Group	0	-	
Zizania latifolia	0	-	
Aegilops tauschii	0	-	
Triticum urartu	0	-	
Nelumbo nucifera	0	-	

Species	# L1 hits	Length distribution (bp)	Notes
Lupinus angustifolius	0	-	
Phaseolus vulgaris	0	-	
Cajanus cajan	0	-	
Vigna angularis var. angularis	0	-	
Vigna radiata var. radiata	0	-	
Glycine max	0	-	
Glycine soja	0	-	
Cicer arietinum	0	-	
Medicago truncatula	0	-	
Trifolium pratense	0	-	
Lotus japonicus	0	-	
Malus x domestica	0	-	
Pyrus x bretschneideri	0	-	
Prunus mume	0	-	
Prunus persica	0	-	
Fragaria iinumae	0	-	
Fragaria nubicola	0	-	
Fragaria orientalis	0	-	
Fragaria vesca subsp. vesca	0	-	
Fragaria x ananassa	0	-	
Morus notabilis	0	-	
Cannabis sativa	0	-	
Castanea mollissima	0	-	
Betula nana	0	-	
Cucumis melo	0	-	
Cucumis sativus	0	-	
Citrullus lanatus	0	-	
Lagenaria siceraria	0	-	
Populus euphratica	0	-	
Populus trichocarpa	0	-	
Jatropha curcas	0	-	
Manihot esculenta subsp. flabellifolia	0	-	
Ricinus communis	0	-	
Linum usitatissimum	0	-	
Eucalyptus camaldulensis	0	-	
Eucalyptus grandis	0	-	
Carica papaya	0	-	
Arabidopsis halleri subsp. gemmifera	0	-	
Arabidopsis lyrata subsp. lyrata	0	-	
Arabidopsis thaliana	0	-	
Camelina sativa	0	-	

**Species**	**# L1 hits**	**Length distribution (bp)**	**Notes**
Capsella rubella	0	-	
Brassica napus	0	-	
Brassica oleracea var. oleracea	0	-	
Brassica rapa	0	-	
Raphanus raphanistrum subsp. raphanistrum	0	-	
Raphanus sativus	0	-	
Aethionema arabicum	0	-	
Arabis alpina	0	-	
Eutrema parvulum	0	-	
Eutrema salsugineum	0	-	
Sisymbrium irio	0	-	
Leavenworthia alabamica	0	-	
Tarenaya hassleriana	0	-	
Gossypium arboreum	0	-	
Gossypium raimondii	0	-	
Theobroma cacao	0	-	
Aquilaria agallochum	0	-	
Azadirachta indica	0	-	
Citrus clementine	0	-	
Citrus sinensis	0	-	
Vitis vinifera	0	-	
Amaranthus hypochondriacus	0	-	
Amaranthus tuberculatus	0	-	
Beta vulgaris subsp. vulgaris	0	-	
Spinacia oleracea	0	-	
Dianthus caryophyllus	0	-	
Actinidia chinensis	0	-	
Vaccinium macrocarpon	0	-	
Diospyros lotus	0	-	
Primula veris	0	-	
Solanum arcanum	0	-	
Solanum habrochaites	0	-	
Solanum lycopersicum	0	-	
Solanum melongena	0	-	
Solanum pennellii	0	-	
Solanum pimpinellifolium	0	-	
Solanum tuberosum	0	-	
Capsicum annuum	0	-	
Nicotiana sylvestris	0	-	
Nicotiana tomentosiformis	0	-	
Fraxinus excelsior	0	-	

**Species**	**# L1 hits**	**Length distribution (bp)**	**Notes**
Penstemon centranthifolius	0	-	
Penstemon grinnellii	0	-	
Sesamum indicum	0	-	
Genlisea aurea	0	-	
Mimulus guttatus	0	-	
Conyza canadensis	0	-	

Table S4: Genome coverage of L1 and BovB elements

Table S4: Shows the calculations used to generate the bargraph in Fig 1 of the manuscript. BovB and L1 base counts are primarily based on full-length elements. Species that don't have L1s nor BovBs are not listed.

**Species**	**Genome base count**	**BovB base count, percentage of genome**	**L1 base count, percentage of genome**
**MAMMALIA**			
Tachyglossus aculeatus	2020007912	1452458, 0.0719036%	0, 0%
Ornithorhynchus anatinus	1996811212	1830086, 0.0916504%	0, 0%
Monodelphis domestica	3605631728	3273062, 0.0907764%	291203623, 8.07636%
Macropus eugenii	3075184024	1715506, 0.0557855%	77958311, 2.53508%
Sarcophilus harrisii	3174693010	3031341, 0.0954845%	95592375, 3.01107%
Dasypus novemcinctus	3631505655	0, 0%	257879875, 7.10118%
Choloepus hoffmanni	2458927620	0, 0%	145367124, 5.91181%
Chrysochloris asiatica	4210093806	108751003, 2.5831%	108660240, 2.58095%
Echinops telfairi	2947007737	20606032, 0.699219%	53945548, 1.83052%
Orycteropus afer	4444063711	102512334, 2.30673%	196663330, 4.4253%
Elephantulus edwardii	3843982861	161028145, 4.1891%	161200386, 4.19358%
Trichechus manatus	3103791524	27624301, 0.890018%	198750171, 6.40346%
Procavia capensis	2985258999	19116374, 0.640359%	177090807, 5.93218%
Loxodonta africana	3271792967	34432824, 1.05241%	313797498, 9.59099%
Erinaceus europaeus	2715703478	0, 0%	80293621, 2.95664%
Sorex araneus	2423158183	0, 0%	79834929, 3.29466%
Condylura cristata	1769662895	0, 0%	42147711, 2.38168%
Pteropus alecto	1985958707	112864, 0.0056831%	109229895, 5.50011%
Pteropus vampyrus	1996076410	79090, 0.00396227%	67971403, 3.40525%
Eidolon helvum	1837754460	100637, 0.00547609%	50920531, 2.7708%
Megaderma lyra	1735931796	72504, 0.00417666%	43660935, 2.51513%
Rhinolophus ferrumequinum	1926439238	119310, 0.00619329%	50326653, 2.61242%
Pteronotus parnellii	1960317893	78360, 0.00399731%	56150083, 2.86434%
Eptesicus fuscus	2026629342	58336, 0.00287847%	59441803, 2.93304%
Myotis brandtii	2107242811	84290, 0.00400001%	72446482, 3.43798%
Myotis davidii	2059799708	71276, 0.00346034%	51012406, 2.47657%
Myotis lucifugus	2034575300	87666, 0.00430881%	84847408, 4.17028%
Ceratotherium simum simum	2464350348	292804, 0.0118816%	160676871, 6.52005%
Equus przewalskii	2395937679	247857, 0.0103449%	135134972, 5.64017%
Equus caballus Thoroughbred	2484532062	223242, 0.00898527%	191150416, 7.69362%
Equus caballus Mongolian	2377506909	265688, 0.0111751%	123172165, 5.18073%
Manis pentadactyla	2204732179	0, 0%	112483171, 5.1019%
Felis catus	2455541136	0, 0%	163774030, 6.66957%
Panthera tigris altaica	2391065193	0, 0%	155431501, 6.50051%
Canis lupus familiaris	2410960148	0, 0%	163347315, 6.7752%
Ursus maritimus	2301362327	0, 0%	154988843, 6.73466%

**Species**	**Genome base count**	**BovB base count, percentage of genome**	**L1 base count, percentage of genome**
Ailuropoda melanoleuca	2299509015	0, 0%	105301329, 4.5793%
Leptonychotes weddellii	3156886159	0, 0%	112493456, 3.56343%
Odobenus rosmarus divergens	2400133628	0, 0%	137079744, 5.71134%
Mustela putorius furo	2410863155	0, 0%	143960591, 5.97133%
Camelus dromedarius	2004047047	0, 0%	101966754, 5.08804%
Camelus ferus	2009177929	0, 0%	86491041, 4.3048%
Vicugna pacos	2172191320	0, 0%	113049718, 5.20441%
Sus scrofa Duroc	2808509378	0, 0%	176658966, 6.29013%
Sus scrofa Tibetan	2489155924	0, 0%	134715498, 5.4121%
Sus scrofa Ellegaard Gottingen	2358017222	0, 0%	117750484, 4.99362%
Balaenoptera acutorostrata scammoni	2431671281	0, 0%	190619715, 7.83904%
Physeter catodon	2280711356	0, 0%	146560263, 6.42608%
Lipotes vexillifer	2429195737	0, 0%	271942192, 11.1947%
Tursiops truncatus	2551401796	0, 0%	119886176, 4.69884%
Orcinus orca	2372903489	0, 0%	205346717, 8.65382%
Pantholops hodgsonii	2696869832	200623622, 7.43913%	90462265, 3.35434%
Capra hircus	2635832257	225462902, 8.55377%	95239112, 3.61325%
Ovis aries Texel	2619054388	249955158, 9.54372%	108872698, 4.15695%
Ovis aries musimon	2589834840	200496380, 7.74167%	71959650, 2.77854%
Bubalus bubalis	2836150610	286296759, 10.0946%	104875469, 3.69781%
Bison bison bison	2953606000	315448446, 10.6801%	127745325, 4.32506%
Bos mutus	2645145588	217297691, 8.21496%	82289329, 3.11096%
Bos indicus	2673949103	287691777, 10.7591%	110741149, 4.14148%
Bos taurus	2670422299	309871737, 11.6038%	126156398, 4.72421%
Ochotona princeps	2229824103	0, 0%	34195120, 1.53353%
Oryctolagus cuniculus	2737490501	0, 0%	139192850, 5.08469%
Ictidomys tridecemlineatus	2478393770	0, 0%	107499669, 4.33747%
Heterocephalus glaber	2618188253	0, 0%	155993240, 5.95806%
Fukomys damarensis	2333892479	0, 0%	161817287, 6.93337%
Cavia aperea	2716396567	0, 0%	136757020, 5.0345%
Cavia porcellus	2723219641	0, 0%	235803425, 8.65899%
Chinchilla lanigera	2390852391	0, 0%	128385455, 5.36986%
Octodon degus	2995872505	0, 0%	138452612, 4.62145%
Dipodomys ordii	2158502098	0, 0%	34330234, 1.59047%
Jaculus jaculus	2835233679	0, 0%	96017503, 3.38658%
Nannospalax galili	3061408210	0, 0%	136788334, 4.46815%
Mesocricetus auratus	2504908775	0, 0%	80503182, 3.21382%
Cricetulus griseus	2399770464	0, 0%	79279532, 3.30363%
Microtus ochrogaster	2287340943	0, 0%	52224812, 2.28321%

Species	Genome base count	BovB base count, percentage of genome	L1 base count, percentage of genome
Peromyscus maniculatus bairdii	2630541020	0, 0%	53188010, 2.02194%
Rattus norvegicus	2909682625	0, 0%	207049946, 7.11589%
Mus musculus	2730855475	0, 0%	231145319, 8.46421%
Tupaia belangeri	3660774957	0, 0%	51474914, 1.40612%
Tupaia chinensis	2846580235	0, 0%	89655790, 3.1496%
Galeopterus variegatus	3187643824	0, 0%	139985621, 4.39151%
Otolemur garnettii	2519724550	0, 0%	146546821, 5.81599%
Microcebus murinus	2902270736	0, 0%	68180677, 2.34922%
Tarsius syrichta	3453847770	0, 0%	240202647, 6.95464%
Callithrix jacchus	2914958544	0, 0%	246251310, 8.44785%
Saimiri boliviensis	2608572064	0, 0%	179339572, 6.87501%
Rhinopithecus roxellana	2899535590	0, 0%	207691102, 7.16291%
Nasalis larvatus	3011966170	0, 0%	154710505, 5.13653%
Chlorocebus sabaeus	2789639778	0, 0%	204188513, 7.31953%
Macaca fascicularis	2946827162	0, 0%	224863209, 7.63069%
Macaca mulatta	2969988180	0, 0%	154566974, 5.2043%
Papio anubis	2948380710	0, 0%	223973218, 7.59648%
Nomascus leucogenys	2962077449	0, 0%	230918510, 7.79583%
Pongo abelii	3441227734	0, 0%	263895353, 7.66864%
Gorilla gorilla	3029537234	0, 0%	212654289, 7.01937%
Pan paniscus	2869173508	0, 0%	241958348, 8.43303%
Pan troglodytes	3309577922	0, 0%	243238952, 7.34955%
Homo sapiens	3101788170	0, 0%	247845149, 7.9904%
**SAUROPSIDA**			
Apalone spinifera	1931078847	0, 0%	1378934, 0.0714074%
Pelodiscus sinensis	2202466388	0, 0%	1581495, 0.0718056%
Chelonia mydas	2208393880	0, 0%	4027549, 0.182375%
Chrysemys picta bellii	2365749696	0, 0%	3441306, 0.145464%
Struthio camelus australis	1225025301	0, 0%	23472, 0.00191604%
Tinamus guttatus	1047056493	0, 0%	8938, 0.000853631%
Anas platyrhynchos	1105035747	0, 0%	21072, 0.00190691%
Lyrurus tetrix	657025294	0, 0%	6332, 0.000963738%
Gallus gallus	1046932099	0, 0%	9721, 0.000928522%
Coturnix japonica	531959683	0, 0%	2234, 0.000419957%
Meleagris gallopavo	1061817101	0, 0%	6761, 0.000636739%
Colinus virginianus	1171855925	0, 0%	62926, 0.00536977%
Acanthisitta chloris	1035876403	0, 0%	25733, 0.00248418%
Manacus vitellinus	1145871783	0, 0%	19832, 0.00173073%
Zonotrichia albicollis	1052600561	0, 0%	12478, 0.00118544%
Geospiza fortis	1065292181	0, 0%	16040, 0.00150569%
Serinus canaria	1152083301	0, 0%	59037, 0.00512437%
Taeniopygia guttata	1233186341	0, 0%	15804, 0.00128156%
Ficedula albicollis	1118326800	0, 0%	16340, 0.00146111%

Species	Genome base count	BovB base count, percentage of genome	L1 base count, percentage of genome
Pseudopodoces humilis	1042980823	0, 0%	18864, 0.00180866%
Corvus brachyrhynchos	1091312783	0, 0%	23878, 0.00218801%
Corvus cornix cornix	1049947905	0, 0%	20517, 0.0019541%
Ara macao	1204683257	0, 0%	18584, 0.00154265%
Amazona vittata	1175404042	0, 0%	23188, 0.00197277%
Melopsittacus undulatus	1117373619	0, 0%	20189, 0.00180683%
Nestor notabilis	1053559886	0, 0%	26182, 0.0024851%
Falco cherrug	1174811715	0, 0%	27430, 0.00233484%
Falco peregrinus	1171955363	0, 0%	26702, 0.00227841%
Cariama cristata	1132245425	0, 0%	34112, 0.00301277%
Merops nubicus	1062961556	0, 0%	23329, 0.00219472%
Picoides pubescens	1167323935	0, 0%	9100, 0.000779561%
Buceros rhinoceros silvestris	1065782791	0, 0%	18845, 0.00176818%
Apaloderma vittatum	1070836417	0, 0%	24052, 0.00224609%
Leptosomus discolor	1136244952	0, 0%	23034, 0.0020272%
Haliaeetus albicilla	1133549865	0, 0%	35410, 0.00312381%
Haliaeetus leucocephalus	1178409481	0, 0%	35701, 0.00302959%
Aquila chrysaetos canadensis	1192725744	0, 0%	33910, 0.00284307%
Cathartes aura	1152571117	0, 0%	38120, 0.00330739%
Tyto alba	1120143088	0, 0%	38958, 0.00347795%
Colius striatus	1075931597	0, 0%	24811, 0.002306%
Charadrius vociferus	1219859583	0, 0%	33056, 0.00270982%
Balearica regulorum gibbericeps	1127605500	0, 0%	35488, 0.0031472%
Chlamydotis macqueenii	1086566339	0, 0%	31476, 0.00289683%
Cuculus canorus	1153894225	0, 0%	19702, 0.00170744%
Fulmarus glacialis	1141395646	0, 0%	35689, 0.00312679%
Aptenodytes forsteri	1254347440	0, 0%	43865, 0.00349704%
Pygoscelis adeliae	1216600033	0, 0%	43236, 0.00355384%
Phalacrocorax carbo	1138967842	0, 0%	29871, 0.00262264%
Pelecanus crispus	1160924693	0, 0%	34119, 0.00293895%
Nipponia nippon	1223846297	0, 0%	37927, 0.003099%
Egretta garzetta	1206484573	0, 0%	36603, 0.00303386%
Gavia stellata	1129677294	0, 0%	38510, 0.00340894%
Tauraco erythrolophus	1155540733	0, 0%	27530, 0.00238243%
Opisthocomus hoazin	1203712246	0, 0%	45662, 0.00379343%
Columba livia	1107971856	0, 0%	28752, 0.00259501%
Pterocles gutturalis	1069324295	0, 0%	32937, 0.00308017%
Phaethon lepturus	1152958507	0, 0%	29672, 0.00257355%
Calypte anna	1105676412	0, 0%	17154, 0.00155145%
Chaetura pelagica	1119188094	0, 0%	16739, 0.00149564%
Caprimulgus carolinensis	1119683066	0, 0%	32635, 0.00291466%

Species	Genome base count	BovB base count, percentage of genome	L1 base count, percentage of genome
Eurypyga helias	1088019637	0, 0%	20244, 0.00186063%
Mesitornis unicolor	1087290853	0, 0%	22907, 0.0021068%
Podiceps cristatus	1134922578	0, 0%	23144, 0.00203926%
Phoenicopterus ruber	1132184511	0, 0%	38050, 0.00336076%
Alligator mississippiensis	2174259888	0, 0%	5988372, 0.275421%
Alligator sinensis	2270550999	0, 0%	6999721, 0.308283%
Crocodylus porosus	2120573303	0, 0%	4601841, 0.217009%
Gavialis gangeticus	2188353730	0, 0%	4914507, 0.224576%
Pogona vitticeps	1816116151	34892879, 1.92129%	3992505, 0.219838%
Anolis carolinensis	1799143587	10307612, 0.572918%	13231926, 0.735457%
Vipera berus berus	1532390814	5053647, 0.329788%	4980408, 0.325009%
Crotalus mitchellii pyrrhus	1126791715	827313, 0.073422%	1914415, 0.1699%
Ophiophagus hannah	1594074654	1525335, 0.0956878%	6627163, 0.415737%
Python bivittatus	1435034535	3932416, 0.274029%	3913976, 0.272744%
**AMPHIBIA**			
Xenopus tropicalis	1437513269	22381, 0.00155692%	10038037, 0.698292%
Xenopus laevis	2718416252	24071, 0.000885479%	23728273, 0.872871%
Nanorana parkeri	2053849526	39534, 0.00192487%	20631712, 1.00454%
**NEOPTERYGII**			
Lepisosteus oculatus	945861706	74445, 0.0078706%	805428, 0.0851528%
Anguilla anguilla	1018701900	0, 0%	1178915, 0.115727%
Anguilla japonica	1151120721	0, 0%	1520279, 0.132069%
Danio rerio	1412464843	20681, 0.00146418%	6341494, 0.448967%
Astyanax mexicanus	1191242572	0, 0%	655594, 0.0550345%
Oryzias latipes	869801494	0, 0%	3737740, 0.429723%
Poecilia formosa	748923461	0, 0%	1514195, 0.202183%
Xiphophorus maculatus	729647787	0, 0%	1081259, 0.148189%
Fundulus heteroclitus	1021882034	0, 0%	2463073, 0.241033%
Takifugu flavidus	378032400	0, 0%	587772, 0.155482%
Takifugu rubripes	391484715	0, 0%	683087, 0.174486%
Tetraodon nigroviridis	358618246	0, 0%	224868, 0.062704%
Cynoglossus semilaevis	470182763	6328, 0.00134586%	129721, 0.0275895%
Haplochromis burtoni	831411547	0, 0%	1596803, 0.192059%
Pundamilia nyererei	830133247	0, 0%	1663150, 0.200347%
Maylandia zebra	849595388	0, 0%	1930306, 0.227203%
Neolamprologus brichardi	847893845	0, 0%	1660506, 0.195839%
Oreochromis niloticus	927696114	12320, 0.00132802%	3182269, 0.343029%
Sebastes nigrocinctus	687549873	0, 0%	665754, 0.0968299%
Sebastes rubrivinctus	756296653	0, 0%	636171, 0.0841166%
Gasterosteus aculeatus	463354448	0, 0%	416171, 0.089817%
Gadus morhua	824327835	0, 0%	1086653, 0.131823%
**ECDYSOZOA**			
Ephemera danica	475911277	6758, 0.00142001%	101494, 0.0213262%

**Species**	**Genome base count**	**BovB base count, percentage of genome**	**L1 base count, percentage of genome**
Ladona fulva	1158111285	0, 0%	448102, 0.0386925%
Pediculus humanus corporis	110781312	0, 0%	33247, 0.0300114%
Frankliniella occidentalis	415781860	0, 0%	123702, 0.0297517%
Diaphorina citri	485705082	44563, 0.00917491%	521323, 0.107333%
Pachypsylla venusta	701763251	0, 0%	90121, 0.0128421%
Acyrthosiphon pisum	541675471	0, 0%	125434, 0.0231567%
Nilaparvata lugens	1140786310	150418, 0.0131855%	1477747, 0.129538%
Oncopeltus fasciatus	1098671531	0, 0%	98206, 0.129538%
Rhodnius prolixus	702645054	0, 0%	132347, 0.0188355%
Cimex lectularius	650477627	88661, 0.0136301%	312967, 0.0481134%
Onthophagus taurus	270533845	0, 0%	131103, 0.0484608%
Agrilus planipennis	353546665	0, 0%	102367, 0.0289543%
Tribolium castaneum	210248733	0, 0%	29847, 0.014196%
Anoplophora glabripennis	707712193	0, 0%	89797, 0.0126883%
Leptinotarsa decemlineata	1170241964	0, 0%	381273, 0.0325807%
Dendroctonus ponderosae	252847629	0, 0%	93067, 0.0368075%
Mengenilla moldrzyki	155727465	0, 0%	7178, 0.00460933%
Aedes aegypti	1383957531	0, 0%	5075165, 0.366714%
Culex quinquefasciatus	579042118	0, 0%	1308898, 0.226045%
Anopheles albimanus	170508315	0, 0%	20956, 0.0122903%
Anopheles arabiensis	246567867	0, 0%	100922, 0.0409307%
Anopheles atroparvus	224290125	0, 0%	128395, 0.0572451%
Anopheles christyi	172658580	0, 0%	60272, 0.0349082%
Anopheles culicifacies	202998806	0, 0%	142545, 0.0702196%
Anopheles darlingi	136935538	0, 0%	13318, 0.00972574%
Anopheles dirus	216307690	0, 0%	268454, 0.124107%
Anopheles epiroticus	223486714	0, 0%	123461, 0.0552431%
Anopheles farauti	183103254	0, 0%	275859, 0.150658%
Anopheles funestus	225223604	0, 0%	141113, 0.0626546%
Anopheles gambiae	287805703	0, 0%	259922, 0.0903116%
Anopheles maculatus	141894015	0, 0%	66565, 0.0469118%
Anopheles melas	224162116	0, 0%	66478, 0.0296562%
Anopheles merus	288048996	0, 0%	155301, 0.0539148%
Anopheles minimus	201793324	0, 0%	107274, 0.0531603%
Anopheles quadriannulatus	283828998	0, 0%	96223, 0.0339018%
Anopheles sinensis	220777669	0, 0%	86072, 0.0389858%
Anopheles stephensi	221324304	0, 0%	70137, 0.0316897%
Mayetiola destructor	185827756	0, 0%	25099, 0.0135066%
Lutzomyia longipalpis	154229266	0, 0%	11504, 0.00745903%
Phlebotomus papatasi	363767980	0, 0%	44465, 0.0122235%
Ceratitis capitata	484773492	0, 0%	62638, 0.0129211%
Drosophila albomicans	253560284	0, 0%	12982, 0.00511989%

**Species**	**Genome base count**	**BovB base count, percentage of genome**	**L1 base count, percentage of genome**
Drosophila ananassae	230993012	0, 0%	6612, 0.00286242%
Drosophila biarmipes	169378599	0, 0%	9683, 0.00571678%
Drosophila bipectinata	167263958	0, 0%	10275, 0.00614299%
Drosophila elegans	171267669	0, 0%	7777, 0.00454085%
Drosophila erecta	152712140	0, 0%	6451, 0.00422429%
Drosophila eugracilis	156942009	0, 0%	8434, 0.00537396%
Drosophila ficusphila	152439475	0, 0%	84442, 0.0553938%
Drosophila grimshawi	200467819	0, 0%	13335, 0.00665194%
Drosophila kikkawai	164292578	0, 0%	12034, 0.00732474%
Drosophila melanogaster	143706478	0, 0%	4915, 0.00342017%
Drosophila miranda	136728780	0, 0%	10424, 0.00762385%
Drosophila mojavensis	193826310	0, 0%	35669, 0.0184026%
Drosophila persimilis	188374079	0, 0%	7072, 0.00375423%
Drosophila pseudoobscura pseudoobscura	152696384	0, 0%	7046, 0.00461439%
Drosophila rhopaloa	197375704	0, 0%	4465, 0.00226218%
Drosophila sechellia	166577145	0, 0%	4293, 0.00257718%
Drosophila simulans	137828247	0, 0%	3689, 0.00267652%
Drosophila suzukii	232923092	0, 0%	18122, 0.00778025%
Drosophila takahashii	182106768	0, 0%	7223, 0.00396635%
Drosophila virilis	206026697	0, 0%	15396, 0.00747282%
Drosophila willistoni	235516348	0, 0%	8718, 0.00370165%
Drosophila yakuba	165693946	0, 0%	7898, 0.00476662%
Musca domestica	750403944	0, 0%	65208, 0.00868972%
Glossina austeni	370264922	0, 0%	47253, 0.0127619%
Glossina brevipalpis	315360362	0, 0%	59919, 0.0190002%
Glossina fuscipes fuscipes	374774708	0, 0%	49182, 0.0131231%
Glossina morsitans morsitans	363107242	0, 0%	48753, 0.0134266%
Glossina pallidipes	357332231	0, 0%	36772, 0.0102907%
Limnephilus lunatus	1345859447	0, 0%	43713, 0.00324796%
Papilio glaucus	374815656	179060, 0.0477728%	68581, 0.0182973%
Papilio polytes	227005758	0, 0%	10239, 0.00451046%
Papilio xuthus	243890167	0, 0%	33700, 0.0138177%
Heliconius melpomene	273786188	10600, 0.00387163%	5968, 0.0021798%
Melitaea cinxia	389892349	0, 0%	13137, 0.00336939%
Danaus plexippus	272853388	20020, 0.00733727%	18718, 0.00686009%
Bombyx mori	481803763	435424, 0.0903737%	1615213, 0.335243%
Manduca sexta	419412261	31594, 0.00753292%	26668, 0.00635842%
Plutella xylostella	393454548	28327, 0.00719956%	209156, 0.0531589%
Athalia rosae	163837890	0, 0%	13934, 0.00850475%
Cephus cinctus	162226239	0, 0%	33326, 0.0205429%
Orussus abietinus	201220334	0, 0%	18324, 0.00910644%

**Species**	**Genome base count**	**BovB base count, percentage of genome**	**L1 base count, percentage of genome**
Ceratosolen solmsi marchali	277059195	0, 0%	68603, 0.0247611%
Nasonia giraulti	283606953	0, 0%	17238, 0.00607813%
Nasonia longicornis	285726340	0, 0%	14085, 0.00492954%
Nasonia vitripennis	295780872	0, 0%	28451, 0.00961895%
Copidosoma floridanum	555044930	0, 0%	80844, 0.0145653%
Trichogramma pretiosum	196221301	0, 0%	37097, 0.0189057%
Microplitis demolitor	250525215	0, 0%	133621, 0.0533363%
Megachile rotundata	272660569	0, 0%	83736, 0.0307107%
Apis dorsata	230340171	0, 0%	11849, 0.00514413%
Apis florea	230467781	0, 0%	11235, 0.00487487%
Apis mellifera	250270657	0, 0%	12542, 0.00501137%
Bombus impatiens	249185056	0, 0%	43105, 0.0172984%
Bombus terrestris	248654244	0, 0%	20613, 0.00828982%
Linepithema humile	219500750	6510, 0.00296582%	34557, 0.0157435%
Camponotus floridanus	232685334	0, 0%	27476, 0.0118082%
Acromyrmex echinatior	295944863	0, 0%	21595, 0.00729697%
Atta cephalotes	317671980	0, 0%	14002, 0.00440769%
Solenopsis invicta	396009169	28437, 0.00718089%	31306, 0.00790537%
Pogonomyrmex barbatus	235645958	4063, 0.0017242%	10237, 0.00434423%
Harpegnathos saltator	294465601	0, 0%	78553, 0.0266765%
Cerapachys biroi	212825769	0, 0%	21905, 0.0102925%
Blattella germanica	2037201033	0, 0%	2091710, 0.102676%
Zootermopsis nevadensis	485009472	0, 0%	64328, 0.0132632%
Locusta migratoria	5759798599	2236765, 0.0388341%	7904827, 0.137241%
Daphnia pulex	197206209	0, 0%	270547, 0.13719%
Eurytemora affinis	494890867	0, 0%	290790, 0.0587584%
Lepeophtheirus salmonis	665129626	0, 0%	12393542, 1.86333%
Hyalella azteca	1178848281	0, 0%	100730, 0.00854478%
Strigamia maritima	176210797	0, 0%	64283, 0.0364807%
Stegodyphus mimosarum	2738704917	0, 0%	1142876, 0.0417305%
Latrodectus hesperus	1137104656	0, 0%	236269, 0.0207781%
Parasteatoda tepidariorum	1443909906	0, 0%	139984, 0.00969479%
Tetranychus urticae	90815494	0, 0%	34385, 0.0378625%
Dermatophagoides farinae	53545338	0, 0%	4466, 0.0083406%
Sarcoptes scabiei type canis	56262437	0, 0%	16880, 0.0300023%
Achipteria coleoptrata	88443889	0, 0%	9799, 0.0110793%
Hypochthonius rufulus	172365132	0, 0%	11855, 0.00687784%
Platynothrus peltifer	100532934	0, 0%	12889, 0.0128207%
Steganacarus magnus	113565242	0, 0%	20382, 0.0179474%
Ixodes ricinus	391986416	0, 0%	914108, 0.233199%
Ixodes scapularis	1765382190	0, 0%	2014997, 0.114139%
Rhipicephalus microplus	144692238	0, 0%	181040, 0.125121%

Species	Genome base count	BovB base count, percentage of genome	L1 base count, percentage of genome
Metaseiulus occidentalis	151699350	0, 0%	72727, 0.0479415%
Varroa destructor	294133941	0, 0%	3597, 0.00122291%
Centruroides exilicauda	926399729	184507, 0.0199166%	855306, 0.0923258%
Mesobuthus martensii	925546267	524438, 0.0566625%	396089, 0.0427952%
Limulus polyphemus	1828256766	0, 0%	496277, 0.0271448%
Trichinella spiralis	63525422	0, 0%	8033, 0.0126453%
Ascaris suum	269559681	0, 0%	30473, 0.0113047%
Steinernema monticolum	89158954	0, 0%	17504, 0.0196324%
Panagrellus redivivus	65093147	0, 0%	13032, 0.0200205%
Haemonchus contortus	319757902	0, 0%	352460, 0.110227%
Necator americanus	244075060	0, 0%	29269, 0.0119918%
Heterorhabditis bacteriophora	76974349	0, 0%	244, 0.000316989%
Caenorhabditis angaria	79761545	0, 0%	7885, 0.00988572%
Caenorhabditis brenneri	190369721	0, 0%	38619, 0.0202863%
Caenorhabditis briggsae	108478526	0, 0%	7158, 0.00659854%
Caenorhabditis elegans	100272607	0, 0%	5988, 0.00597172%
Caenorhabditis japonica	166256191	0, 0%	41298, 0.02484%
Caenorhabditis sp. 11 MAF-2010	79321433	0, 0%	5899, 0.00743683%
Priapulus caudatus	420178403	0, 0%	179152, 0.0426371%
**PLATYHELMINTHES**			
Schistosoma curassoni	344202215	0, 0%	63816, 0.0185403%
Schistosoma haematobium	375894156	0, 0%	42415, 0.0112838%
Schistosoma japonicum	402743189	0, 0%	94449, 0.0234514%
Schistosoma mansoni	364518427	0, 0%	18207, 0.00499481%
Schistosoma margrebowiei	367396528	0, 0%	30287, 0.00824368%
Schistosoma mattheei	340818150	0, 0%	52043, 0.01527%
Schistosoma rodhaini	343294136	0, 0%	29809, 0.00868322%
Clonorchis sinensis	547288241	0, 0%	137669, 0.0251548%
Echinococcus granulosus	112350571	0, 0%	4822, 0.00429192%
Echinococcus multilocularis	7371474	0, 0%	4135, 0.0560946%
Hymenolepis microstoma	126773784	0, 0%	5297, 0.00417831%
Protopolystoma xenopodis	617344661	0, 0%	28573, 0.00462837%
**OTHER**			
Callorhinchus milii	974481817	0, 0%	96001, 0.00985149%
Carcharhinus brachyurus	2832541493	0, 0%	1383347, 0.0488377%
Lytechinus variegatus	951759914	4192, 0.000440447%	1562525, 0.164172%
Strongylocentrotus purpuratus	936564995	55567, 0.00593306%	2699297, 0.288212%
Patiria miniata	811028858	0, 0%	350468, 0.0432128%
Saccoglossus kowalevskii	775840678	0, 0%	2440029, 0.314501%
Ciona intestinalis	115212710	0, 0%	78094, 0.0677825%

Species	Genome base count	BovB base count, percentage of genome	L1 base count, percentage of genome
Ciona savignyi	587352817	29071, 0.0049495%	4044971, 0.688678%
Botryllus schlosseri	579633380	0, 0%	1256055, 0.216698%
Oikopleura dioica	70471451	0, 0%	5276, 0.00748672%
Branchiostoma floridae	521895125	0, 0%	884348, 0.169449%
Lethenteron camtschaticum	1030662718	27180, 0.00263714%	1011092, 0.0981012%
Petromyzon marinus	885550958	0, 0%	680080, 0.0767974%
Latimeria chalumnae	2860591921	113934, 0.00398288%	14770485, 0.516344%
Adineta vaga	217933776	32135, 0.0147453%	44968, 0.0206338%
Capitella teleta	333283208	9147, 0.00274451%	17739, 0.0053225%
Helobdella robusta	235376169	139023, 0.0590642%	400522, 0.170163%
Crassostrea gigas	557717710	0, 0%	1746428, 0.313138%
Lottia gigantea	359505668	0, 0%	628379, 0.17479%
Aplysia californica	927296314	0, 0%	645572, 0.0696187%
Biomphalaria glabrata	916374414	0, 0%	335123, 0.0365705%
Nematostella vectensis	356613585	0, 0%	186002, 0.0521579%
Hydra vulgaris	852155112	0, 0%	1764624, 0.207078%
Mnemiopsis leidyi	155865547	0, 0%	60221, 0.0386365%
Trichoplax adhaerens	105631681	0, 0%	0, 0%
Amphimedon queenslandica	166679601	0, 0%	7730, 0.00463764%
**FUNGI**			
Coprinopsis cinerea	36192590	0, 0%	7417, 0.0204931%
Agaricus bisporus var. burnettii	32614392	0, 0%	31084, 0.0953076%
Agaricus bisporus var. bisporus	30233745	0, 0%	28062, 0.0928168%
Laccaria bicolor	64877444	0, 0%	4236, 0.00652923%
Schizophyllum commune	38482026	0, 0%	5965, 0.0155007%
Moniliophthora roreri	52204869	0, 0%	4106, 0.00786517%
Serpula lacrymans	42797233	0, 0%	0, 0%
Coniophora puteana	42968544	0, 0%	15013, 0.0349395%
Heterobasidion irregulare	33649967	0, 0%	10227, 0.0303923%
Stereum hirsutum	46511623	0, 0%	11201, 0.0240822%
Dichomitus squalens	42748430	0, 0%	10461, 0.0244711%
Trametes versicolor	44794008	0, 0%	90802, 0.20271%
Fibroporia radiculosa	28378039	0, 0%	770, 0.00271337%
Phanerochaete carnosa	46293325	0, 0%	18096, 0.0390899%
Postia placenta	90891856	0, 0%	8820, 0.00970384%
Gloeophyllum trabeum	37178225	0, 0%	20507, 0.0551586%
Punctularia strigosozonata	34171901	0, 0%	44534, 0.130323%
Fomitiporia mediterranea	63354419	0, 0%	1142, 0.00180256%
Auricularia subglabra	74920202	0, 0%	195051, 0.260345%

Species	Genome base count	BovB base count, percentage of genome	L1 base count, percentage of genome
Malassezia sympodialis	7669689	0, 0%	3964, 0.051684%
Ustilago maydis	19664356	0, 0%	2996, 0.0152357%
Puccinia graminis	88724376	0, 0%	752, 0.000847569%
Melampsora larici-populina	101129028	0, 0%	10131, 0.0100179%
Rhodotorula toruloides	20223942	0, 0%	2537, 0.0125445%
Dactylellina haptotyla	39531920	0, 0%	1802, 0.00455834%
Tuber melanosporum	124945702	0, 0%	2089, 0.00167193%
Talaromyces atroroseus	30858562	0, 0%	1589, 0.0051493%
Talaromyces stipitatus	35685443	0, 0%	6125, 0.0171639%
Ajellomyces dermatitidis	75404732	0, 0%	24833, 0.0329329%
Histoplasma capsulatum	33030326	0, 0%	1442, 0.00436569%
Arthroderma otae	23263091	0, 0%	318, 0.00136697%
Coccidioides immitis	29016019	0, 0%	3321, 0.0114454%
Paracoccidioides brasiliensis	29952540	0, 0%	1357, 0.0045305%
Uncinocarpus reesii	22349738	0, 0%	1552, 0.00694415%
Endocarpon pusillum	37130611	0, 0%	2963, 0.00797994%
Pseudogymnoascus destructans	30684937	0, 0%	5229, 0.0170409%
Beauveria bassiana	33697794	0, 0%	1795, 0.00532676%
Fusarium oxysporum	61386934	0, 0%	5556, 0.00905079%
Fusarium verticillioides	41844914	0, 0%	1292, 0.00308759%
Colletotrichum gloeosporioides	55607143	0, 0%	1235, 0.00222094%
Colletotrichum higginsianum	50716103	0, 0%	4418, 0.00871124%
Colletotrichum orchidophilum	48556462	0, 0%	2174, 0.00447726%
Thermothelomyces thermophila	38744216	0, 0%	1081, 0.00279009%
Thielavia terrestris	36912256	0, 0%	3957, 0.01072%
Sordaria macrospora	39955017	0, 0%	1025, 0.00256538%
Pseudocercospora fijiensis	74141167	0, 0%	18239, 0.0246004%
Sphaerulina musiva	29352103	0, 0%	1589, 0.00541358%
Zymoseptoria tritici	39686251	0, 0%	7312, 0.0184245%
Parastagonospora nodorum	37213987	0, 0%	3811, 0.0102408%
Leptosphaeria maculans	45124619	0, 0%	20091, 0.0445234%
Metschnikowia bicuspidata	16055203	0, 0%	1926, 0.0119961%
Scheffersomyces stipitis	15441179	0, 0%	70712, 0.457944%
Candida albicans	14282666	0, 0%	10100, 0.0707151%
Candida auris	12498763	0, 0%	2327, 0.0186178%
Candida dubliniensis	14618422	0, 0%	33605, 0.229881%

Species	Genome base count	BovB base count, percentage of genome	L1 base count, percentage of genome
Candida tanzawaensis	13136204	0, 0%	1844, 0.0140375%
Wickerhamomyces anomalus	14145566	0, 0%	5054, 0.0357285%
Wickerhamomyces ciferrii	15901152	0, 0%	13860, 0.0871635%
Yarrowia lipolytica	20550897	0, 0%	84838, 0.412819%
Saitoella complicata	14136186	0, 0%	2291, 0.0162066%
Phycomyces blakesleeanus	53939167	0, 0%	335552, 0.622093%
Rhizopus microsporus	25972395	0, 0%	45192, 0.174%
Lobosporangium transversale	42768949	0, 0%	35232, 0.0823775%
Nosema ceranae	7860219	0, 0%	912, 0.0116027%
**VIRIDIPLANTAE**			
Chlamydomonas reinhardtii	120185366	0, 0%	606792, 0.50488%
Volvox carteri f. nagariensis	137684403	0, 0%	396743, 0.288154%
Chlorella variabilis	46159512	0, 0%	12154, 0.0263304%
Coccomyxa subellipsoidea C-169	48826616	0, 0%	342720, 0.701912%
Klebsormidium flaccidum	103921766	0, 0%	14535, 0.0139865%
Physcomitrella patens	479985347	0, 0%	209444, 0.0436355%
Selaginella moellendorffii	212315224	0, 0%	432326, 0.203625%
Pinus taeda	265480119	0, 0%	3355235, 1.26384%
Amborella trichopoda	706332640	0, 0%	10079924, 1.42708%
Spirodela polyrhiza	132009443	0, 0%	217054, 0.164423%
Phoenix dactylifera	555607186	0, 0%	4794765, 0.862977%
Elaeis oleifera	1402725009	0, 0%	4644120, 0.331078%
Ensete ventricosum	172241963	0, 0%	45089, 0.0261777%
Musa acuminata subsp. malaccensis	472235617	0, 0%	861006, 0.182326%
Sorghum bicolor	738540932	0, 0%	6065256, 0.821248%
Zea mays	2066912289	0, 0%	9490541, 0.459165%
Setaria italica	405737341	0, 0%	4216652, 1.03926%
Brachypodium distachyon	271923306	0, 0%	4154965, 1.52799%
Leersia perrieri	266687832	0, 0%	2368362, 0.888065%
Oryza barthii	308272304	0, 0%	2043982, 0.663044%
Oryza brachyantha	259907595	0, 0%	570749, 0.219597%
Oryza glumipatula	372860283	0, 0%	1891897, 0.507401%
Oryza longistaminata	326442508	0, 0%	1729078, 0.529673%
Oryza meridionalis	335668232	0, 0%	1621453, 0.483052%
Oryza nivara	337950324	0, 0%	2024556, 0.599069%
Oryza punctata	393816603	0, 0%	1970705, 0.500412%
Oryza sativa Japonica group	382150945	0, 0%	2353016, 0.615729%

Species	Genome base count	BovB base count, percentage of genome	L1 base count, percentage of genome
Zizania latifolia	603989347	0, 0%	2151532, 0.35622%
Aegilops tauschii	3313650219	0, 0%	21648619, 0.653316%
Triticum urartu	3747047519	0, 0%	21997415, 0.58706%
Nelumbo nucifera	804484421	0, 0%	19049323, 2.36789%
Lupinus angustifolius	523297923	0, 0%	3311489, 0.632811%
Phaseolus vulgaris	521076696	0, 0%	5674591, 1.08901%
Cajanus cajan	510809477	0, 0%	1658557, 0.324692%
Vigna angularis var. angularis	291823841	0, 0%	58656, 0.0200998%
Vigna radiata var. radiata	463085359	0, 0%	91202, 0.0196944%
Glycine max	973224514	0, 0%	7291899, 0.749251%
Glycine soja	863568428	0, 0%	5855382, 0.678045%
Cicer arietinum	530768543	0, 0%	864712, 0.162917%
Medicago truncatula	314353944	0, 0%	2757877, 0.877316%
Trifolium pratense	304979311	0, 0%	1723289, 0.565051%
Lotus japonicus	147812252	0, 0%	1521949, 1.02965%
Malus x domestica	526197889	0, 0%	2239129, 0.42553%
Pyrus x bretschneideri	508550595	0, 0%	2367713, 0.465581%
Prunus mume	233872527	0, 0%	1080390, 0.461957%
Prunus persica	227251827	0, 0%	977650, 0.430206%
Fragaria iinumae	199627509	0, 0%	848595, 0.425089%
Fragaria nubicola	203686407	0, 0%	852989, 0.418776%
Fragaria orientalis	214184023	0, 0%	861282, 0.402122%
Fragaria vesca subsp. vesca	214217322	0, 0%	1330049, 0.620888%
Fragaria x ananassa	173229572	0, 0%	576965, 0.333064%
Morus notabilis	320378613	0, 0%	540366, 0.168665%
Cannabis sativa	757438891	0, 0%	7007555, 0.925164%
Castanea mollissima	833240550	0, 0%	7968700, 0.95635%
Betula nana	564011153	0, 0%	4178216, 0.740804%
Cucumis melo	374772472	0, 0%	1244169, 0.33198%
Cucumis sativus	242977439	0, 0%	799120, 0.328887%
Citrullus lanatus	321046825	0, 0%	1165484, 0.363026%
Lagenaria siceraria	176727258	0, 0%	507954, 0.287423%
Populus euphratica	495875768	0, 0%	1763571, 0.355648%
Populus trichocarpa	417129638	0, 0%	1895269, 0.45436%
Jatropha curcas	318363250	0, 0%	1992515, 0.625862%
Manihot esculenta subsp. flabellifolia	390835892	0, 0%	1771302, 0.453209%
Ricinus communis	350458699	0, 0%	664578, 0.189631%
Linum usitatissimum	282201865	0, 0%	2053058, 0.727514%
Eucalyptus camaldulensis	654922307	0, 0%	5906037, 0.901792%
Eucalyptus grandis	691269672	0, 0%	10125615, 1.46479%
Carica papaya	369781828	0, 0%	2516437, 0.680519%

**Species**	**Genome base count**	**BovB base count, percentage of genome**	**L1 base count, percentage of genome**
Arabidopsis halleri subsp. gemmifera	221139660	0, 0%	2629722, 1.18917%
Arabidopsis lyrata subsp. lyrata	206667935	0, 0%	3211589, 1.55399%
Arabidopsis thaliana	119146348	0, 0%	907867, 0.761976%
Camelina sativa	641356059	0, 0%	13704182, 2.13675%
Capsella rubella	133063876	0, 0%	1196138, 0.89892%
Brassica napus	930121500	0, 0%	19061867, 2.0494%
Brassica rapa	283975909	0, 0%	5186525, 1.8264%
Brassica oleracea var. oleracea	488593889	0, 0%	10794431, 2.20928%
Raphanus raphanistrum subsp. raphanistrum	253833977	0, 0%	3953706, 1.5576%
Raphanus sativus	341476849	0, 0%	6558491, 1.92063%
Aethionema arabicum	192487774	0, 0%	2497220, 1.29734%
Arabis alpina	308032609	0, 0%	6413649, 2.08213%
Eutrema parvulum	137073209	0, 0%	1218003, 0.888578%
Eutrema salsugineum	243110105	0, 0%	3730545, 1.53451%
Sisymbrium irio	245550082	0, 0%	3420058, 1.39281%
Leavenworthia alabamica	173431618	0, 0%	1145977, 0.660766%
Tarenaya hassleriana	249929577	0, 0%	898531, 0.359514%
Gossypium arboreum	1560831891	0, 0%	5673504, 0.363492%
Gossypium raimondii	761405269	0, 0%	6287854, 0.825822%
Theobroma cacao	345993675	0, 0%	2489010, 0.71938%
Aquilaria agallochum	726710453	0, 0%	2696090, 0.370999%
Azadirachta indica	261457759	0, 0%	450277, 0.172218%
Citrus clementina	301364702	0, 0%	2772560, 0.920002%
Citrus sinensis	327669411	0, 0%	2960816, 0.903599%
Vitis vinifera	485326422	0, 0%	10020983, 2.06479%
Amaranthus hypochondriacus	502147592	0, 0%	1858411, 0.370093%
Amaranthus tuberculatus	4347977	0, 0%	47856, 1.10065%
Beta vulgaris subsp. vulgaris	566198563	0, 0%	14816651, 2.61686%
Spinacia oleracea	474077292	0, 0%	4811255, 1.01487%
Dianthus caryophyllus	567661845	0, 0%	3994415, 0.703661%
Actinidia chinensis	604217145	0, 0%	1447675, 0.239595%
Vaccinium macrocarpon	414621889	0, 0%	2174742, 0.524512%
Diospyros lotus	1104189	0, 0%	3332, 0.30176%
Primula veris	309692940	0, 0%	1089758, 0.351883%
Solanum arcanum	665186956	0, 0%	5259355, 0.790658%
Solanum habrochaites	724284658	0, 0%	5928289, 0.818503%
Solanum lycopersicum	781353311	0, 0%	5086040, 0.650927%
Solanum melongena	833080760	0, 0%	5215063, 0.625997%

**Species**	**Genome base count**	**BovB base count, percentage of genome**	**L1 base count, percentage of genome**
Solanum pennellii	720458090	0, 0%	5173838, 0.718132%
Solanum pimpinellifolium	688247059	0, 0%	5291330, 0.768813%
Solanum tuberosum	705779115	0, 0%	7014366, 0.993847%
Capsicum annuum	3063642317	0, 0%	18450182, 0.60223%
Nicotiana sylvestris	2221831232	0, 0%	12836977, 0.577766%
Nicotiana tomentosiformis	1688312294	0, 0%	9735272, 0.576627%
Fraxinus excelsior	875243685	0, 0%	2738111, 0.31284%
Penstemon centranthifolius	4471593	0, 0%	24561, 0.549267%
Penstemon grinnellii	3663524	0, 0%	14843, 0.405156%
Sesamum indicum	274906174	0, 0%	4526917, 1.64671%
Genlisea aurea	43357795	0, 0%	124160, 0.286361%
Mimulus guttatus	321641293	0, 0%	2116247, 0.657953%
Conyza canadensis	326165195	0, 0%	1471338, 0.451102%

Table S5: BovB HT candidate clusters

Table S5: BLASTALL and VSEARCH were used to find BovB HT candidates. Clusters were kept if they contained L1s from two or more different Eukaryotic Classes and passed the *in silico* validation tests. The clustering was run three times: first, using nucleotide BovB sequences between 2.4-4kb in length; second, using nucleotide open reading frames found within BovBs; and third, using amino acid reverse-transcriptase domains only. See Methods for details.

**Family**	**Number of BovBs from each species**	**Eukaryotic Classes**	**Eukaryotic Phyla**
**Nucleotide BovB sequences**			
**CROSS-PHYLUM clusters**			
c_0	135 Anolis carolinensis, 14504 Bison bison, 1 Bombyx mori, 18932 Bos indicus, 5335 Bos mutus, 20798 Bos taurus, 11603 Bubalus bubalis, 5827 Capra hircus, 2 Centruroides exilicauda, 1 Cimex lectularius, 1 Ciona savignyi, 1 Cynoglossus semilaevis, 1 Helobdella robusta, 1 Latimeria chalumnae, 10 Locusta migratoria, 19 Macropus eugenii, 8 Mesobuthus martensii, 20 Monodelphis domestica, 7 Ophiophagus hannah, 8178 Ovis aries Texel, 6315 Ovis aries musimon, 3026 Pantholops hodgsonii, 308 Pogona vitticeps, 5 Python bivittatus, 36 Sarcophilus harrisii, 21 Vipera berus	Actinopterygii, Arachnida, Ascidiacea, Clitellata, Insecta, Mammalia, Reptilia, Sarcopterygii	Annelida, Arthropoda, Chordata
c_1	197 Chrysochloris asiatica, 86 Echinops telfairi, 478 Elephantulus edwardii, 136 Loxodonta africana, 1 Mesobuthus martensii, 7724 Orycteropus afer, 576 Procavia capensis, 79 Trichechus manatus	Arachnida, Mammalia	Arthropoda, Chordata
c_21	2912 Chrysochloris asiatica, 43 Echinops telfairi, 41 Elephantulus edwardii, 3 Helobdella robusta, 30 Loxodonta africana, 2 Mesobuthus martensii, 40 Orycteropus afer, 65 Procavia capensis, 25 Trichechus manatus	Arachnida, Clitellata, Mammalia	Annelida, Arthropoda, Chordata
c_23	3 Crotalus mitchellii, 1 Danio rerio, 2 Helobdella robusta, 3 Lepisosteus oculatus, 1 Mesobuthus martensii, 4 Ophiophagus hannah, 29 Python bivittatus	Actinopterygii, Arachnida, Clitellata, Reptilia	Annelida, Arthropoda, Chordata
c_33	2 Adineta vaga, 6 Python bivittatus	Bdelloidea, Reptilia	Chordata, Rotifera
c_4	7 Chrysochloris asiatica, 443 Elephantulus edwardii, 1 Mesobuthus martensii, 3 Orycteropus afer	Arachnida, Mammalia	Arthropoda, Chordata
c_41	2 Centruroides exilicauda, 1 Locusta migratoria, 54 Macropus eugenii, 51 Monodelphis domestica, 1 Pogona vitticeps, 96 Sarcophilus harrisii	Arachnida, Insecta, Mammalia, Reptilia	Arthropoda, Chordata
c_46	3 Ciona savignyi, 4 Strongylocentrotus purpuratus	Ascidiacea, Echinoidea	Chordata, Echinodermata
c_53	6 Centruroides exilicauda, 1 Crotalus mitchellii, 2 Ophiophagus hannah	Arachnida, Reptilia	Arthropoda, Chordata
c_55	1 Adineta vaga, 1 Helobdella robusta	Bdelloidea, Clitellata	Annelida, Rotifera

Family	Number of BovBs from each species	Eukaryotic Classes	Eukaryotic Phyla
c_7	61 Anolis carolinensis, 1593 Bison bison, 1151 Bos indicus, 1301 Bos mutus, 1192 Bos taurus, 1488 Bubalus bubalis, 1117 Capra hircus, 2 Locusta migratoria, 1 Macropus eugenii, 1021 Ovis aries Texel, 1040 Ovis aries musimon, 800 Pantholops hodgsonii, 100 Pogona vitticeps, 1 Sarcophilus harrisii, 11 Vipera berus	Insecta, Mammalia, Reptilia	Arthropoda, Chordata
**CROSS-CLASS clusters**			
c_106	36 Bison bison, 8 Bos indicus, 28 Bos mutus, 15 Bos taurus, 44 Bubalus bubalis, 18 Capra hircus, 19 Ovis aries Texel, 14 Ovis aries musimon, 20 Pantholops hodgsonii, 2 Pogona vitticeps	Mammalia, Reptilia	Chordata
c_14	5 Anolis carolinensis, 1425 Bison bison, 1373 Bos indicus, 1566 Bos mutus, 1553 Bos taurus, 1519 Bubalus bubalis, 1390 Capra hircus, 11 Ophiophagus hannah, 1440 Ovis aries Texel, 1343 Ovis aries musimon, 1319 Pantholops hodgsonii, 398 Pogona vitticeps, 1 Sarcophilus harrisii, 53 Vipera berus	Mammalia, Reptilia	Chordata
c_15	22 Ceratotherium simum, 22 Equus caballus Mongolian, 22 Equus caballus Thoroughbred, 24 Equus przewalskii, 1 Megaderma lyra, 4 Pogona vitticeps, 2 Rhinolophus ferrumequinum	Mammalia, Reptilia	Chordata
c_19	1332 Bison bison, 1321 Bos indicus, 700 Bos mutus, 1371 Bos taurus, 1285 Bubalus bubalis, 830 Capra hircus, 810 Ovis aries Texel, 603 Ovis aries musimon, 272 Pantholops hodgsonii, 1 Vipera berus	Mammalia, Reptilia	Chordata
c_2	2 Anolis carolinensis, 2674 Bison bison, 2088 Bos indicus, 1778 Bos mutus, 2061 Bos taurus, 2380 Bubalus bubalis, 1424 Capra hircus, 4 Macropus eugenii, 1 Monodelphis domestica, 10 Ophiophagus hannah, 1250 Ovis aries Texel, 1573 Ovis aries musimon, 1194 Pantholops hodgsonii, 154 Pogona vitticeps, 1 Sarcophilus harrisii, 6 Vipera berus	Mammalia, Reptilia	Chordata
c_49	6 Diaphorina citri, 1 Mesobuthus martensii, 1 Nilaparvata lugens	Arachnida, Insecta	Arthropoda
c_5	354 Bison bison, 78 Bos indicus, 287 Bos mutus, 65 Bos taurus, 343 Bubalus bubalis, 143 Capra hircus, 80 Ovis aries Texel, 265 Ovis aries musimon, 134 Pantholops hodgsonii, 1 Pogona vitticeps	Mammalia, Reptilia	Chordata
c_80	1 Centruroides exilicauda, 1 Mesobuthus martensii, 2 Nilaparvata lugens	Arachnida, Insecta	Arthropoda

Nucleotide ORFs

CROSS-PHYLUM clusters

Family	Number of BovBs from each species	Eukaryotic Classes	Eukaryotic Phyla
o_0	11 Adineta vaga, 245 Anolis carolinensis, 59268 Bos indicus, 15219 Bos mutus, 63518 Bos taurus, 29581 Bubalus bubalis, 8688 Capra hircus, 6 Centruroides exilicauda, 1 Chrysochloris asiatica, 5 Cimex lectularius, 2 Helobdella robusta, 1 Lepisosteus oculatus, 53 Locusta migratoria, 1 Loxodonta africana, 8 Macropus eugenii, 6 Mesobuthus martensii, 3 Monodelphis domestica, 17 Ophiophagus hannah, 24910 Ovis aries Texel, 15373 Ovis aries musimon, 7554 Pantholops hodgsonii, 1177 Pogona vitticeps, 2 Procavia capensis, 34 Python bivittatus, 1 Sarcophilus harrisii, 1 Trichechus manatus, 424 Vipera berus	Actinopterygii, Arachnida, Bdelloidea, Clitellata, Insecta, Mammalia, Reptilia	Annelida, Arthropoda, Chordata, Rotifera
o_106	2 Centruroides exilicauda, 5 Lethenteron camtschaticum	Arachnida, Hyperoartia	Arthropoda, Chordata
o_120	1 Bison bison, 2 Bos indicus, 2 Bos taurus, 1 Bubalus bubalis, 2 Capra hircus, 5 Cimex lectularius, 2 Locusta migratoria, 6 Ophiophagus hannah, 1 Ovis aries Texel, 2 Pantholops hodgsonii, 3825 Pogona vitticeps, 257 Vipera berus	Insecta, Mammalia, Reptilia	Arthropoda, Chordata
o_122	1 Anolis carolinensis, 275 Bison bison, 260 Bos indicus, 282 Bos mutus, 251 Bos taurus, 297 Bubalus bubalis, 239 Capra hircus, 2 Cimex lectularius, 9 Ophiophagus hannah, 223 Ovis aries Texel, 246 Ovis aries musimon, 214 Pantholops hodgsonii, 1 Python bivittatus, 61 Vipera berus	Insecta, Mammalia, Reptilia	Arthropoda, Chordata
o_16	4 Ephemera danica, 1 Myotis brandtii	Insecta, Mammalia	Arthropoda, Chordata
o_326	1 Bos indicus, 1 Cimex lectularius, 18 Ophiophagus hannah, 2 Pogona vitticeps, 1 Python bivittatus, 348 Vipera berus	Insecta, Mammalia, Reptilia	Arthropoda, Chordata
o_33	4 Danio rerio, 13 Helobdella robusta, 1 Lepisosteus oculatus, 16 Mesobuthus martensii	Actinopterygii, Arachnida, Clitellata	Annelida, Arthropoda, Chordata
o_38	6 Anolis carolinensis, 2 Bos indicus, 2 Bos mutus, 3 Bos taurus, 2 Capra hircus, 3 Locusta migratoria, 2 Ophiophagus hannah, 5 Pantholops hodgsonii, 3305 Pogona vitticeps, 6 Python bivittatus, 14 Vipera berus	Insecta, Mammalia, Reptilia	Arthropoda, Chordata
o_43	198 Chrysochloris asiatica, 18 Echinops telfairi, 71 Elephantulus edwardii, 399 Loxodonta africana, 1 Mesobuthus martensii, 1981 Orycteropus afer, 129 Procavia capensis, 410 Trichechus manatus	Arachnida, Mammalia	Arthropoda, Chordata
o_47	1 Bison bison, 1 Capra hircus, 10 Cimex lectularius, 1 Crotalus mitchellii, 49 Ophiophagus hannah, 1 Ovis aries musimon, 1 Pantholops hodgsonii, 24 Pogona vitticeps, 8 Python bivittatus, 813 Vipera berus	Insecta, Mammalia, Reptilia	Arthropoda, Chordata
o_609	1 Lepisosteus oculatus, 4 Mesobuthus martensii	Actinopterygii, Arachnida	Arthropoda, Chordata

Family	Number of BovBs from each species	Eukaryotic Classes	Eukaryotic Phyla
o_95	2 Anolis carolinensis, 2944 Bison bison, 1982 Bos indicus, 974 Bos mutus, 2002 Bos taurus, 1685 Bubalus bubalis, 883 Capra hircus, 1 Cimex lectularius, 1 Monodelphis domestica, 1 Ophiophagus hannah, 1535 Ovis aries Texel, 1150 Ovis aries musimon, 623 Pantholops hodgsonii, 1 Pogona vitticeps, 8 Vipera berus	Insecta, Mammalia, Reptilia	Arthropoda, Chordata
**CROSS-CLASS clusters**			
o_112	72 Bison bison, 52 Bos indicus, 56 Bos mutus, 52 Bos taurus, 51 Bubalus bubalis, 63 Capra hircus, 2 Macropus eugenii, 69 Ovis aries Texel, 54 Ovis aries musimon, 49 Pantholops hodgsonii, 1 Pogona vitticeps	Mammalia, Reptilia	Chordata
o_118	81 Bison bison, 32 Bos indicus, 19 Bos mutus, 29 Bos taurus, 63 Bubalus bubalis, 19 Capra hircus, 12 Ovis aries Texel, 9 Ovis aries musimon, 11 Pantholops hodgsonii, 1 Python bivittatus	Mammalia, Reptilia	Chordata
o_123	151 Bison bison, 61 Bos indicus, 37 Bos mutus, 61 Bos taurus, 124 Bubalus bubalis, 27 Capra hircus, 28 Ovis aries Texel, 12 Ovis aries musimon, 16 Pantholops hodgsonii, 2 Python bivittatus	Mammalia, Reptilia	Chordata
o_132	1298 Bison bison, 1319 Bos indicus, 860 Bos mutus, 1325 Bos taurus, 1233 Bubalus bubalis, 673 Capra hircus, 3 Ophiophagus hannah, 800 Ovis aries Texel, 762 Ovis aries musimon, 465 Pantholops hodgsonii, 40 Vipera berus	Mammalia, Reptilia	Chordata
o_134	20 Bison bison, 6 Bos indicus, 24 Bos mutus, 13 Bos taurus, 27 Bubalus bubalis, 18 Capra hircus, 12 Ovis aries Texel, 9 Ovis aries musimon, 14 Pantholops hodgsonii, 1 Pogona vitticeps	Mammalia, Reptilia	Chordata
o_139	38 Bison bison, 4 Bos indicus, 20 Bos mutus, 7 Bos taurus, 33 Bubalus bubalis, 26 Capra hircus, 15 Ovis aries Texel, 3 Ovis aries musimon, 14 Pantholops hodgsonii, 1 Pogona vitticeps, 1 Vipera berus	Mammalia, Reptilia	Chordata
o_142	6 Bison bison, 4 Bos mutus, 9 Bubalus bubalis, 5 Capra hircus, 2 Ovis aries Texel, 1 Ovis aries musimon, 6 Pantholops hodgsonii, 1 Python bivittatus	Mammalia, Reptilia	Chordata
o_146	11 Bison bison, 1 Bos indicus, 7 Bos mutus, 1 Bos taurus, 13 Bubalus bubalis, 3 Capra hircus, 3 Ovis aries Texel, 7 Pantholops hodgsonii, 1 Python bivittatus	Mammalia, Reptilia	Chordata
o_156	2 Lethenteron camtschaticum, 1 Oreochromis niloticus	Actinopterygii, Hyperoartia	Chordata
o_168	10 Bison bison, 1 Bos indicus, 3 Bos mutus, 1 Bos taurus, 8 Bubalus bubalis, 5 Capra hircus, 4 Pantholops hodgsonii, 1 Python bivittatus	Mammalia, Reptilia	Chordata

Family	Number of BovBs from each species	Eukaryotic Classes	Eukaryotic Phyla
o_194	130 Bison bison, 100 Bos indicus, 108 Bos mutus, 94 Bos taurus, 122 Bubalus bubalis, 85 Capra hircus, 1 Ophiophagus hannah, 75 Ovis aries Texel, 88 Ovis aries musimon, 91 Pantholops hodgsonii, 7 Vipera berus	Mammalia, Reptilia	Chordata
o_201	316 Bison bison, 250 Bos indicus, 69 Bos mutus, 267 Bos taurus, 206 Bubalus bubalis, 48 Capra hircus, 104 Ovis aries Texel, 94 Ovis aries musimon, 32 Pantholops hodgsonii, 1 Python bivittatus	Mammalia, Reptilia	Chordata
o_226	12 Bison bison, 2 Bos indicus, 3 Bos mutus, 3 Bos taurus, 15 Bubalus bubalis, 6 Capra hircus, 3 Ovis aries Texel, 1 Ovis aries musimon, 9 Pantholops hodgsonii, 1 Pogona vitticeps	Mammalia, Reptilia	Chordata
o_258	40 Bison bison, 26 Bos indicus, 10 Bos mutus, 25 Bos taurus, 31 Bubalus bubalis, 5 Capra hircus, 10 Ovis aries Texel, 19 Ovis aries musimon, 7 Pantholops hodgsonii, 1 Vipera berus	Mammalia, Reptilia	Chordata
o_293	2 Macropus eugenii, 1 Ophiophagus hannah, 81 Pogona vitticeps, 1 Vipera berus	Mammalia, Reptilia	Chordata
o_316	1 Bubalus bubalis, 354 Pogona vitticeps, 1 Vipera berus	Mammalia, Reptilia	Chordata
o_397	8 Ceratotherium simum, 6 Equus caballus Mongolian, 4 Equus caballus Thoroughbred, 5 Equus przewalskii, 1 Megaderma lyra, 1 Myotis davidii, 2 Pogona vitticeps, 2 Rhinolophus ferrumequinum	Mammalia, Reptilia	Chordata
o_492	2 Anolis carolinensis, 1 Bos mutus, 3 Bubalus bubalis, 1 Capra hircus, 1 Ovis aries Texel, 1 Pantholops hodgsonii, 89 Pogona vitticeps, 3 Python bivittatus, 3 Vipera berus	Mammalia, Reptilia	Chordata
o_570	1 Bos taurus, 2 Bubalus bubalis, 2 Pantholops hodgsonii, 1 Python bivittatus	Mammalia, Reptilia	Chordata
o_576	4 Bison bison, 3 Bos indicus, 2 Bos mutus, 3 Bos taurus, 6 Bubalus bubalis, 2 Capra hircus, 4 Ovis aries Texel, 4 Ovis aries musimon, 1 Pantholops hodgsonii, 1 Vipera berus	Mammalia, Reptilia	Chordata
o_74	46 Bison bison, 4 Bos indicus, 5 Bos mutus, 3 Bos taurus, 34 Bubalus bubalis, 22 Capra hircus, 17 Ovis aries Texel, 3 Ovis aries musimon, 12 Pantholops hodgsonii, 1 Vipera berus	Mammalia, Reptilia	Chordata
o_804	6 Capra hircus, 4 Ovis aries Texel, 2 Ovis aries musimon, 4 Pantholops hodgsonii, 1 Python bivittatus	Mammalia, Reptilia	Chordata
o_83	883 Bison bison, 1133 Bos indicus, 478 Bos mutus, 1207 Bos taurus, 643 Bubalus bubalis, 255 Capra hircus, 517 Ovis aries Texel, 477 Ovis aries musimon, 246 Pantholops hodgsonii, 2 Pogona vitticeps	Mammalia, Reptilia	Chordata

Family	Number of BovBs from each species	Eukaryotic Classes	Eukaryotic Phyla
o_99	662 Bison bison, 632 Bos indicus, 204 Bos mutus, 680 Bos taurus, 547 Bubalus bubalis, 151 Capra hircus, 297 Ovis aries Texel, 230 Ovis aries musimon, 143 Pantholops hodgsonii, 1 Python bivittatus	Mammalia, Reptilia	Chordata

Amino acid RT domains

CROSS-PHYLUM clusters

r_0	1 Adineta vaga, 29 Chrysochloris asiatica, 365 Elephantulus edwardii, 99 Loxodonta africana, 3 Mesobuthus martensii, 1 Ornithorhynchus anatinus, 163 Orycteropus afer, 6 Procavia capensis, 81 Trichechus manatus	Arachnida, Bdelloidea, Mammalia	Arthropoda, Chordata, Rotifera
r_10	359 Anolis carolinensis, 2 Bos indicus, 1 Bos mutus, 6 Bos taurus, 1 Bubalus bubalis, 1 Capra hircus, 1 Lepisosteus oculatus, 1 Locusta migratoria, 3 Ovis aries Texel, 3 Ovis aries musimon, 3 Pantholops hodgsonii, 8 Pogona vitticeps, 3 Vipera berus	Actinopterygii, Insecta, Mammalia, Reptilia	Arthropoda, Chordata
r_12	1 Centruroides exilicauda, 3 Helobdella robusta, 14 Mesobuthus martensii	Arachnida, Clitellata	Annelida, Arthropoda
r_2	5919 Bison bison, 13181 Bos indicus, 1463 Bos mutus, 14379 Bos taurus, 3150 Bubalus bubalis, 336 Capra hircus, 1 Cimex lectularius, 2 Ophiophagus hannah, 6467 Ovis aries Texel, 2092 Ovis aries musimon, 1437 Pantholops hodgsonii, 38 Pogona vitticeps, 31 Vipera berus	Insecta, Mammalia, Reptilia	Arthropoda, Chordata
r_80	330 Bison bison, 498 Bos indicus, 54 Bos mutus, 525 Bos taurus, 177 Bubalus bubalis, 26 Capra hircus, 3 Cimex lectularius, 5 Ophiophagus hannah, 97 Ovis aries Texel, 69 Ovis aries musimon, 18 Pantholops hodgsonii, 682 Pogona vitticeps, 221 Vipera berus	Insecta, Mammalia, Reptilia	Arthropoda, Chordata

CROSS-CLASS clusters

r_141	1 Lepisosteus oculatus, 1 Pogona vitticeps	Actinopterygii, Reptilia	Chordata
r_153	1 Macropus eugenii, 1 Ophiophagus hannah, 3 Pogona vitticeps, 2 Vipera berus	Mammalia, Reptilia	Chordata
r_161	1 Bison bison, 2 Bos indicus, 1 Capra hircus, 1 Ovis aries Texel, 1 Vipera berus	Mammalia, Reptilia	Chordata
r_21	296 Bison bison, 518 Bos indicus, 65 Bos mutus, 509 Bos taurus, 144 Bubalus bubalis, 14 Capra hircus, 277 Ovis aries Texel, 102 Ovis aries musimon, 90 Pantholops hodgsonii, 1 Vipera berus	Mammalia, Reptilia	Chordata
r_25	22 Bison bison, 29 Bos indicus, 16 Bos mutus, 19 Bos taurus, 21 Bubalus bubalis, 6 Capra hircus, 16 Ovis aries Texel, 16 Ovis aries musimon, 4 Pantholops hodgsonii, 2 Pogona vitticeps	Mammalia, Reptilia	Chordata

**Family**	**Number of BovBs from each species**	**Eukaryotic Classes**	**Eukaryotic Phyla**
r_36	1 Bison bison, 1 Bos mutus, 3 Bos taurus, 1 Capra hircus, 1 Ovis aries Texel, 2 Ovis aries musimon, 1 Pantholops hodgsonii, 159 Pogona vitticeps, 1 Python bivittatus, 14 Vipera berus	Mammalia, Reptilia	Chordata
r_58	44 Bison bison, 39 Bos indicus, 14 Bos mutus, 40 Bos taurus, 17 Bubalus bubalis, 13 Capra hircus, 22 Ovis aries Texel, 28 Ovis aries musimon, 5 Pantholops hodgsonii, 1 Vipera berus	Mammalia, Reptilia	Chordata

Table S6: L1 HT candidate clusters

Table S6: The same method was used to perform all-against-all clustering of L1 elements and find discordant groupings. Clusters were kept if they contained L1s from two or more different Eukaryotic Classes and passed the *in silico* validation tests. The clustering was run three times: first, using nucleotide L1 sequences between 3-9kb in length; second, using nucleotide open reading frames found within the L1s; and third, using amino acid reverse-transcriptase domains only. Some clusters looked interesting but were dismissed either because the L1s were located on short scaffolds (marked as likely contamination), or because the ORFs did not contain any known functional domains (marked as likely artifacts).

**Family**	**Number of L1s from each species**	**Eukaryotic Classes**	**Eukaryotic Phyla**
**Nucleotide L1 sequences**			
**CROSS-PHYLUM clusters**			
c_220	52 Crassostrea gigas, 2 Lottia gigantea, 1 Saccoglossus kowalevskii, 14 Strongylocentrotus purpuratus	Bivalvia, Gastropoda, Enteropneusta, Echinoida	Mollusca, Hemichordata, Echinodermata
c_25	2 Anguilla anguilla, 8 Crassostrea gigas, 129 Danio rerio	Actinopterygii, Bivalvia	Chordata, Mollusca
**CROSS-CLASS clusters**			
c_26	6 Alligator mississippiensis, 3 Alligator sinensis, 11 Apalone spinifera, 7 Carcharhinus brachyurus, 159 Chelonia mydas, 184 Chrysemys picta, 3 Crocodylus porosus, 3 Gavialis gangeticus, 8 Latimeria chalumnae, 2 Lethenteron camtschaticum, 49 Pelodiscus sinensis, 1 Petromyzon marinus	Sauropsida, Chondrichthyes, Sarcopterygii, Hyperoartia	Chordata
c_41	40 Latimeria chalumnae, 1 Oryzias latipes	Sarcopterygii, Actinopterygii	Chordata
c_65	105 Anolis carolinensis, 11 Ophiophagus hannah, 9 Pogona vitticeps, 4 Vipera berus, 2 Xenopus laevis	Sauropsida, Amphibia	Chordata
**Interesting but likely contamination (short scaffolds)**			
c_14096	1 Drosophila ficusphila, 1 Lytechinus variegatus	Insecta, Echinoidea	Arthropoda, Echinodermata
c_162	1 Protopolystoma xenopodis, 41 Xenopus laevis, 35 Xenopus tropicalis	Amphibia, Monogenea	Chordata, Platyhelminthes
c_2488	1 Drosophila ficusphila, 2 Lytechinus variegatus	Insecta, Echinoidea	Arthropoda, Echinodermata
c_257	1 Cricetulus griseus, 16514 Mus musculus, 18274 Rattus norvegicus, 1 Schistosoma japonicum	Mammalia, Trematoda	Chordata, Platyhelminthes
c_5994	1 Drosophila ficusphila, 1 Lytechinus variegatus	Insecta, Echinoidea	Arthropoda, Echinodermata
c_8560	1 Drosophila ficusphila, 5 Strongylocentrotus purpuratus	Insecta, Echinoidea	Arthropoda, Echinodermata
**Nucleotide ORFs**			
**CROSS-PHYLUM clusters**			

Family	Number of L1s from each species	Eukaryotic Classes	Eukaryotic Phyla
o_374	99 Crassostrea gigas, 1 Lottia gigantea, 2 Priapulus caudatus	Bivalvia, Gastropoda, Priapulimorpha	Mollusca, Priapulida
o_666	26 Crassostrea gigas, 1 Priapulus caudatus	Bivalvia, Priapulimorpha	Mollusca, Priapulida
**CROSS-CLASS clusters**			
o_295	69 Alligator mississippiensis, 97 Alligator sinensis, 168 Apalone spinifera, 1098 Chelonia mydas, 1210 Chrysemys picta, 50 Crocodylus porosus, 70 Gavialis gangeticus, 17 Latimeria chalumnae, 338 Pelodiscus sinensis	Sauropsida, Sarcopterygii	Chordata
o_353	125 Crassostrea gigas, 2 Lottia gigantea	Bivalvia, Gastropoda	Mollusca
o_520	2 Lytechinus variegatus, 3 Patiria miniata, 7 Strongylocentrotus purpuratus	Echinoidea, Asteroidea	Echinodermata
o_559	1 Lytechinus variegatus, 2 Patiria miniata	Echinoidea, Asteroidea	Echinodermata
o_757	10 Crassostrea gigas, 1 Lottia gigantea	Bivalvia, Gastropoda	Mollusca
**Interesting but likely artifacts (no functional domains)**			
o_13226	1 Anopheles minimus, 1 Blattella germanica, 2 Caenorhabditis elegans	Insecta, Chromadorea	Arthropoda, Nematoda
o_13299	3 Anopheles arabiensis, 1 Anopheles merus, 1 Anopheles quadriannulatus, 1 Caenorhabditis 11	Insecta, Chromadorea	Arthropoda, Nematoda
o_15486	1 Anopheles albimanus, 1 Anopheles atroparvus, 2 Anopheles darlingi, 1 Anopheles funestus, 1 Anopheles sinensis, 1 Haemonchus contortus	Insecta, Chromadorea	Arthropoda, Nematoda
o_18254	1 Aedes aegypti, 1 Anopheles albimanus, 1 Caenorhabditis brenneri, 1 Caenorhabditis japonica, 1 Drosophila takahashii, 1 Drosophila virilis, 1 Manduca sexta, 1 Mesobuthus martensii, 1 Steinernema monticolum, 1 Trichogramma pretiosum	Insecta, Chromadorea, Arachnida	Arthropoda, Nematoda
o_21039	1 Caenorhabditis elegans, 2 Centruroides exilicauda	Chromadorea, Arachnida	Arthropoda, Nematoda
o_22604	2 Anopheles culicifacies, 2 Anopheles dirus, 1 Anopheles farauti, 1 Anopheles sinensis, 3 Caenorhabditis 11, 1 Caenorhabditis angaria, 1 Caenorhabditis brenneri, 1 Daphnia pulex, 1 Drosophila bipectinata, 1 Drosophila grimshawi, 1 Drosophila rhopaloa, 1 Melitaea cinxia	Insecta, Chromadorea, Branchiopoda	Arthropoda, Nematoda
o_27328	1 Anopheles arabiensis, 1 Anopheles farauti, 1 Caenorhabditis elegans, 1 Cephus cinctus	Insecta, Chromadorea	Arthropoda, Nematoda
o_4989	3 Drosophila biarmipes, 1 Necator americanus, 1 Strigamia maritima	Insecta, Chromadorea, Chilopoda	Arthropoda, Nematoda

Family	Number of L1s from each species	Eukaryotic Classes	Eukaryotic Phyla
o_18911	1 Anopheles arabiensis, 1 Anopheles epiroticus, 1 Anopheles gambiae, 1 Anopheles melas, 1 Anopheles merus, 1 Anopheles quadriannulatus, 1 Mesobuthus martensii	Insecta, Arachnida	Arthropoda

Interesting but likely contamination (short scaffolds)

o_294	1 Alligator mississippiensis, 6 Latimeria chalumnae, 1 Lethenteron camtschaticum, 2 Protopolystoma xenopodis, 130 Xenopus laevis, 59 Xenopus tropicalis	Sauropsida, Sarcopterygii, Hyperoartia, Amphibia, Monogenea	Chordata, Platy-helminthes
o_1103	2 Drosophila ficusphila, 1 Lytechinus variegatus, 3 Strongylocentrotus purpuratus	Insecta, Echinoidea	Arthropoda, Echinodermata
o_11511	1 Drosophila ficusphila, 6 Lytechinus variegatus	Insecta, Echinoidea	Arthropoda, Echinodermata
o_11634	1 Drosophila ficusphila, 1 Lytechinus variegatus	Insecta, Echinoidea	Arthropoda, Echinodermata
o_12034	1 Drosophila ficusphila, 3 Lytechinus variegatus	Insecta, Echinoidea	Arthropoda, Echinodermata
o_12376	1 Drosophila ficusphila, 2 Lytechinus variegatus	Insecta, Echinoidea	Arthropoda, Echinodermata
o_12900	1 Protopolystoma xenopodis, 1 Xenopus laevis	Amphibia, Monogenea	Chordata, Platy-helminthes
o_13327	1 Fusarium oxysporum, 1 Pteronotus parnellii	Sordari-omycetes, Mammalia	Ascomycota, Chordata
o_15461	1 Drosophila ficusphila, 3 Lytechinus variegatus, 8 Strongylocentrotus purpuratus	Insecta, Echinoidea	Arthropoda, Echinodermata
o_1822	2 Drosophila ficusphila, 1 Lytechinus variegatus, 25 Strongylocentrotus purpuratus	Insecta, Echinoidea	Arthropoda, Echinodermata
o_1886	1 Drosophila ficusphila, 6 Lytechinus variegatus, 3 Strongylocentrotus purpuratus	Insecta, Echinoidea	Arthropoda, Echinodermata
o_19358	1 Drosophila ficusphila, 3 Lytechinus variegatus	Insecta, Echinoidea	Arthropoda, Echinodermata
o_19697	1 Protopolystoma xenopodis, 6 Xenopus laevis	Amphibia, Monogenea	Chordata, Platy-helminthes
o_20044	1 Drosophila ficusphila, 3 Lytechinus variegatus	Insecta, Echinoidea	Arthropoda, Echinodermata
o_2101	2 Drosophila ficusphila, 2 Lytechinus variegatus, 12 Strongylocentrotus purpuratus	Insecta, Echinoidea	Arthropoda, Echinodermata
o_2734	2 Protopolystoma xenopodis, 80 Xenopus laevis, 34 Xenopus tropicalis	Amphibia, Monogenea	Chordata, Platy-helminthes
o_29	1 Protopolystoma xenopodis, 69 Xenopus laevis, 121 Xenopus tropicalis	Amphibia, Monogenea	Chordata, Platy-helminthes

Family	Number of L1s from each species	Eukaryotic Classes	Eukaryotic Phyla
o_3056	2 Drosophila ficusphila, 2 Lytechinus variegatus, 25 Strongylocentrotus purpuratus	Insecta, Echinoidea	Arthropoda, Echinodermata
o_423	2 Protopolystoma xenopodis, 14 Xenopus laevis, 64 Xenopus tropicalis	Amphibia, Monogenea	Chordata, Platy-helminthes
o_488	1 Drosophila ficusphila, 5 Lytechinus variegatus, 35 Strongylocentrotus purpuratus	Insecta, Echinoidea	Arthropoda, Echinodermata
o_504	7 Drosophila ficusphila, 8 Lytechinus variegatus, 69 Strongylocentrotus purpuratus	Insecta, Echinoidea	Arthropoda, Echinodermata
o_575	5 Protopolystoma xenopodis, 36 Xenopus laevis, 1 Xenopus tropicalis	Amphibia, Monogenea	Chordata, Platy-helminthes
o_651	1 Drosophila ficusphila, 10 Lytechinus variegatus, 13 Strongylocentrotus purpuratus	Insecta, Echinoidea	Arthropoda, Echinodermata
o_691	2 Drosophila ficusphila, 8 Lytechinus variegatus, 57 Strongylocentrotus purpuratus	Insecta, Echinoidea	Arthropoda, Echinodermata
o_7039	2 Fusarium oxysporum, 1 Pteronotus parnellii	Sordari-omycetes, Mammalia	Ascomycota, Chordata

Amino acid RT domains

CROSS-PHYLUM clusters

r_1348	3 Branchiostoma floridae, 2 Mnemiopsis leidyi	Cephalochor-data, Tentaculata	Chordata, Ctenophora
r_2916	1 Branchiostoma floridae, 4 Saccoglossus kowalevskii	Cephalochor-data, Enteropneusta	Chordata, Hemichordata

CROSS-CLASS clusters

r_1111	1 Fraxinus excelsior, 4 Phoenix dactylifera	Mesangiosper-mae, Liliopsida	Streptophyta
r_1410	4 Carcharhinus brachyurus, 1 Latimeria chalumnae	Chon-drichthyes, Sarcopterygii	Chordata
r_609	2 Lytechinus variegatus, 3 Patiria miniata, 3 Strongylocentrotus purpuratus	Echinoidea, Asteroidea	Echinodermata
r_67	10 Alligator mississippiensis, 14 Alligator sinensis, 9 Apalone spinifera, 60 Chelonia mydas, 141 Chrysemys picta, 4 Crocodylus porosus, 10 Gavialis gangeticus, 3 Latimeria chalumnae, 17 Pelodiscus sinensis, 1 Xenopus tropicalis	Sauropsida, Sarcopterygii, Amphibia	Chordata

Interesting but likely contamination (short scaffolds)

r_1013	1 Drosophila ficusphila, 2 Lytechinus variegatus, 4 Strongylocentrotus purpuratus	Insecta, Echinoidea	Arthropoda, Echinodermata

Family	Number of L1s from each species	Eukaryotic Classes	Eukaryotic Phyla
r_1179	2 Protopolystoma xenopodis, 11 Xenopus laevis, 20 Xenopus tropicalis	Amphibia, Monogenea	Chordata, Platy-helminthes
r_1417	4 Drosophila ficusphila, 32 Strongylocentrotus purpuratus	Insecta, Echinoidea	Arthropoda, Echinodermata
r_1779	1 Drosophila ficusphila, 1 Strongylocentrotus purpuratus	Insecta, Echinoidea	Arthropoda, Echinodermata
r_1979	1 Colletotrichum higginsianum, 1 Colletotrichum orchidophilum, 1 Fusarium oxysporum, 1 Parastagonospora nodorum, 1 Pteronotus parnellii	Sordari-omycetes, Doth-ideomycetes, Mammalia	Ascomycota, Chordata
r_2368	1 Drosophila ficusphila, 2 Lytechinus variegatus, 1 Strongylocentrotus purpuratus	Insecta, Echinoidea	Arthropoda, Echinodermata
r_3622	1 Drosophila ficusphila, 1 Lytechinus variegatus	Insecta, Echinoidea	Arthropoda, Echinodermata
r_3890	1 Fusarium oxysporum, 1 Pteronotus parnellii	Sordari-omycetes, Mammalia	Ascomycota, Chordata
r_4047	1 Drosophila ficusphila, 1 Lytechinus variegatus	Insecta, Echinoidea	Arthropoda, Echinodermata
r_430	1 Drosophila ficusphila, 3 Lytechinus variegatus, 11 Strongylocentrotus purpuratus	Insecta, Echinoidea	Arthropoda, Echinodermata
r_4915	1 Drosophila ficusphila, 1 Lytechinus variegatus	Insecta, Echinoidea	Arthropoda, Echinodermata
r_498	1 Protopolystoma xenopodis, 1 Xenopus laevis, 23 Xenopus tropicalis	Amphibia, Monogenea	Chordata, Platy-helminthes
r_500	2 Protopolystoma xenopodis, 8 Xenopus laevis, 2 Xenopus tropicalis	Amphibia, Monogenea	Chordata, Platy-helminthes
r_58	46 Cricetulus griseus, 35 Mesocricetus auratus, 9 Microtus ochrogaster, 11274 Mus musculus, 1 Pan troglodytes, 16 Peromyscus maniculatus, 6522 Rattus norvegicus, 1 Schistosoma mansoni, 1 Schistosoma rodhaini, 2 Tetraodon nigroviridis	Mammalia, Trematoda, Actinopterygii	Chordata, Platy-helminthes
r_681	1 Drosophila ficusphila, 17 Strongylocentrotus purpuratus	Insecta, Echinoidea	Arthropoda, Echinodermata
r_917	1 Drosophila ficusphila, 1 Lytechinus variegatus, 13 Strongylocentrotus purpuratus	Insecta, Echinoidea	Arthropoda, Echinodermata
