# Supplementary material for: Horizontal transfer of BovB and L1 retrotransposons in eukaryotes
Source: Genome Biol. 2018 Jul 9;19:85. doi: 10.1186/s13059-018-1456-7 (PMC6036668; doi:10.1186/s13059-018-1456-7)
Supplement: Supplementary file 2 — Figures S1. and S2. show additional discordant L1 clusters. Figures S3–S54. show Kimura divergence plots for species containing both L1 and BovB elements. Figure S55 shows the location of the chimeric L1-BovB element in the cow genome (Bos taurus), showing that there is little evidence of transcription. (PDF 823 kb) [file 13059_2018_1456_MOESM2_ESM.pdf]

Supplementary Figures

Figures S1-2: L1 discordant phylogenies 2

Figures S3-54: Kimura divergence plots for BovB and L1 4

Marsupialia . . . 5
Afrotheria . . . 7
Chiroptera . . . 10
Perissodactyla . . . 16
Bovidae . . . 18
Squamata . . . 22
Amphibia . . . 26
Neopterygii . . . 27
Other . . . 29

Figure S55: Chimeric L1-BovB 31

Figures S1-2: L1 discordant phylogenies

Potential L1 HT clusters were checked using both neighbour-joining and maximum likelihood methods to confirm that the tree topology differed from expected species relationships. The best supported cross-Phylum L1 phylogenies are shown in the main text; the remaining cross-Phylum clusters are shown here. Clusters are described in detail in Table S6.

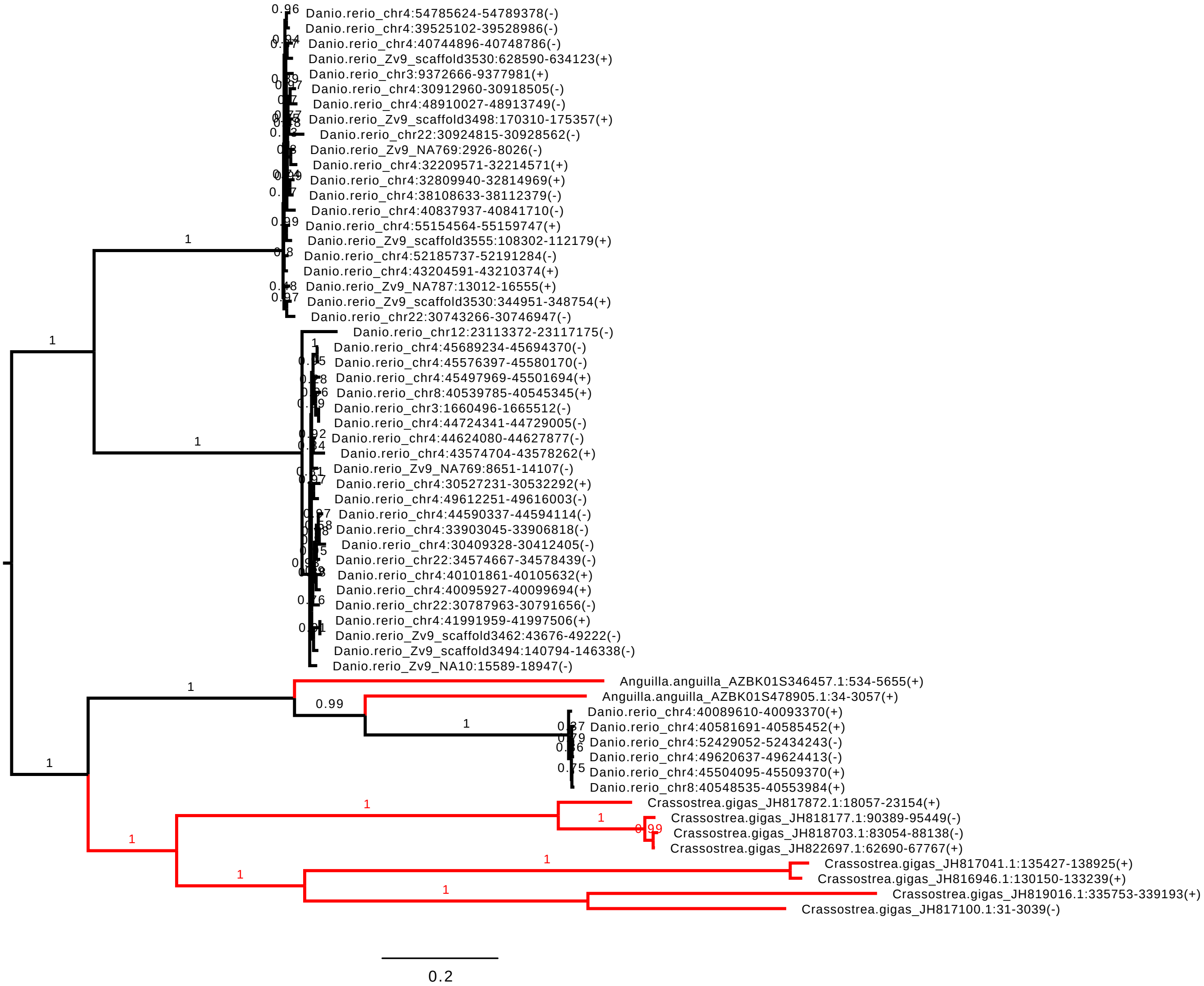

Figure S1: **L1 cluster c_25**

L1 nucleotide ORFs

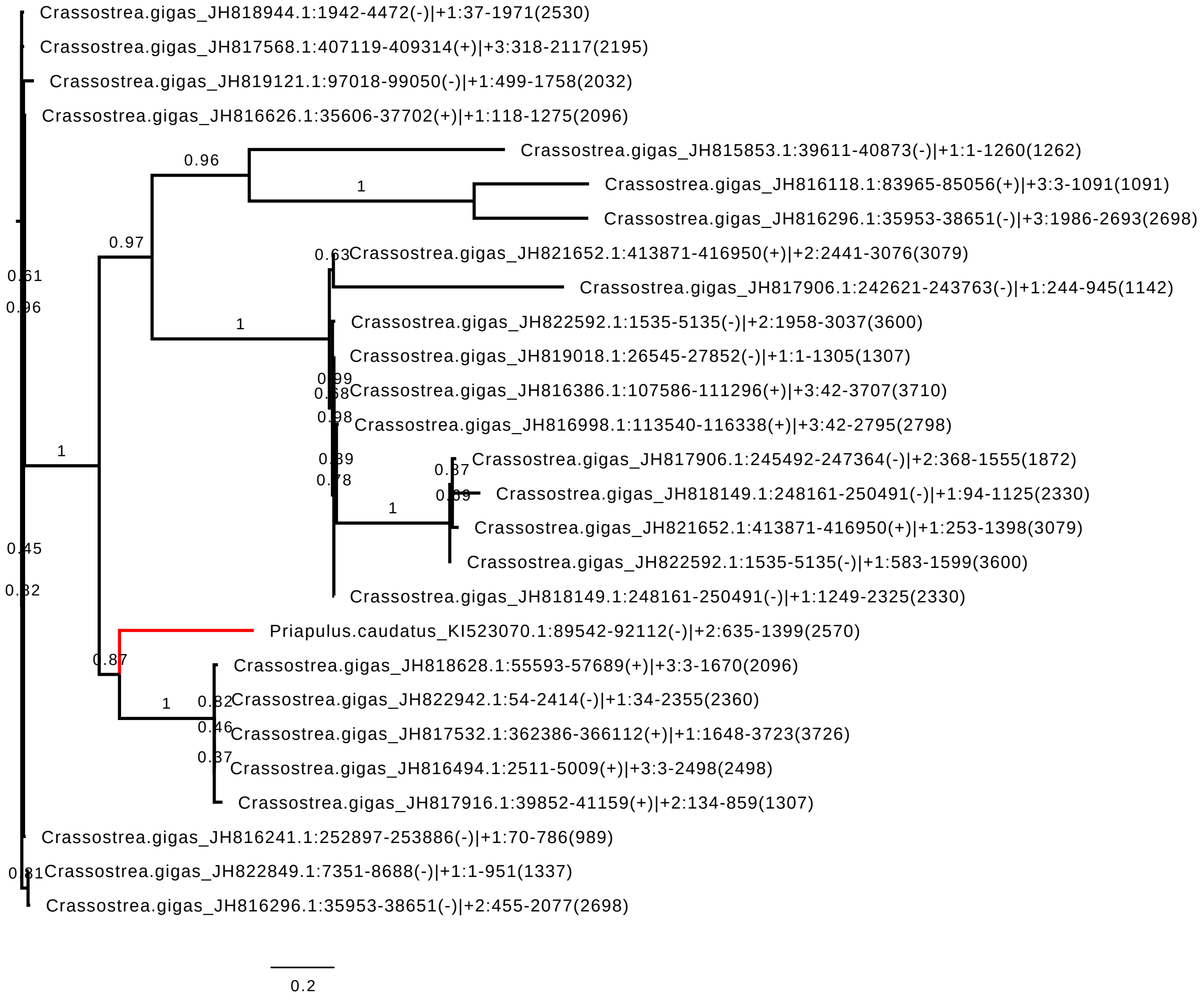

Figure S2: **L1 cluster o_666**

Figures S3-54: Kimura divergence plots for BovB and L1

RepeatMasker divergence plots represent Kimura substitution levels of TEs against the RepBase super consensus library. For example, Figure 5b in the main text shows the RepeatMasker divergence plot for the cow (*Bos taurus*), illustrating recent bursts of BovB and L1 activity in the genome with many copies sharing high identity to young, currently active elements.

The L1 superfamily includes both mammalian L1 elements (dark blue) and more diverse, frog-like Tx elements (light blue). Tx are typically found in fish, frogs and primitive eukaryotes (e.g. sea urchin *Strongylocentrotus purpuratus*). BovB elements are coloured in orange.

Typically, species within a clade show consistent divergence patterns of both TEs (particularly if there has been little recent activity - see Chiroptera). Recently TE-active species, on the other hand, are likely to show bursts of seemingly random activity. Consider the plots for the two lizard species, *Pogona vitticeps* and *Anolis carolinensis*. *Pogona* is implicated in many of the BovB HT events listed in Table S5, and this is supported by the huge burst of recent BovB activity shown in Figure S38. This is also seen in all four snake species. In contrast, the *Anolis* plot (Figure S39) indicates that L1s have become the dominant TE lineage in the genome.

By estimating TE divergence from super consensus sequences, we can visualise the contrasting (and sometimes competing) dynamics of BovB and L1 elements over time. This is particularly important for species where BovB or L1 (or both) have taken off and accumulated quickly within the genome.

Marsupialia

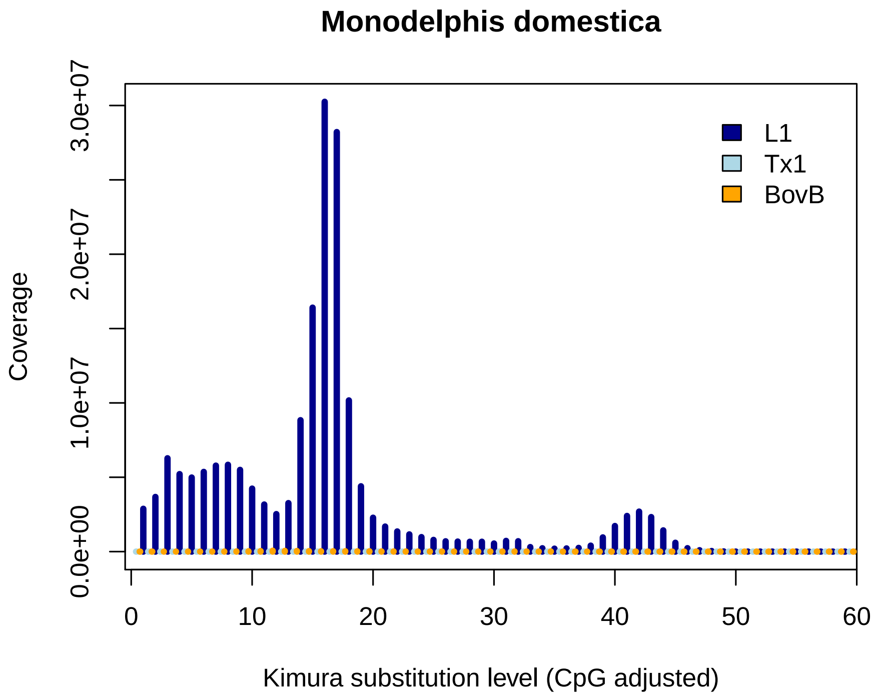

Figure S3

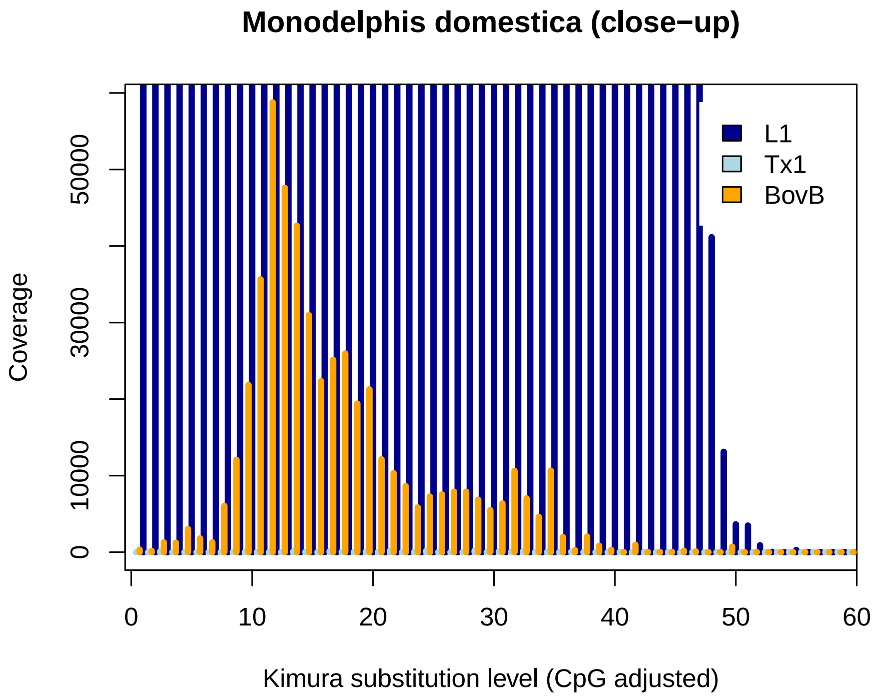

Figure S4

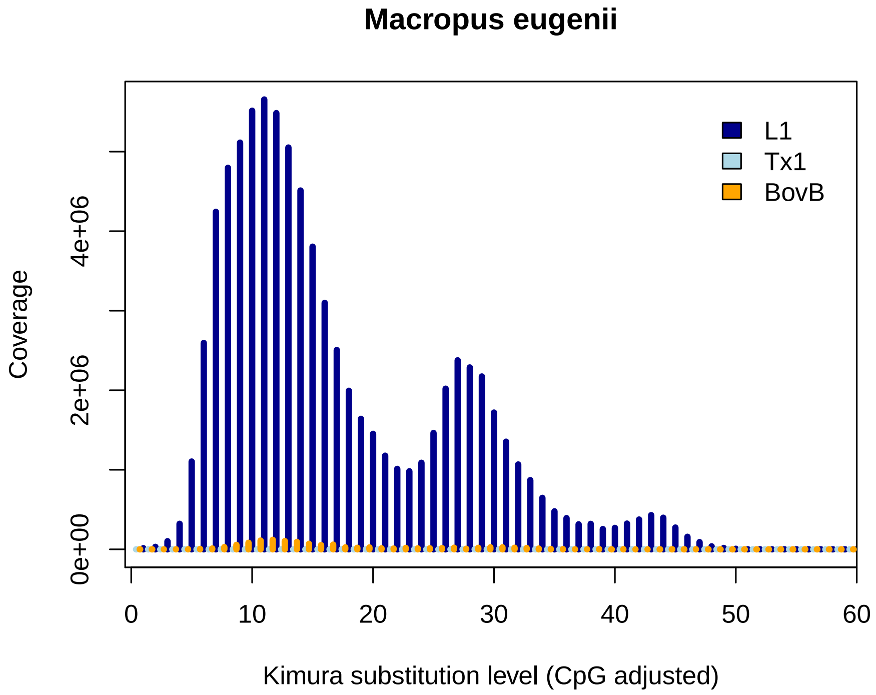

Figure S5

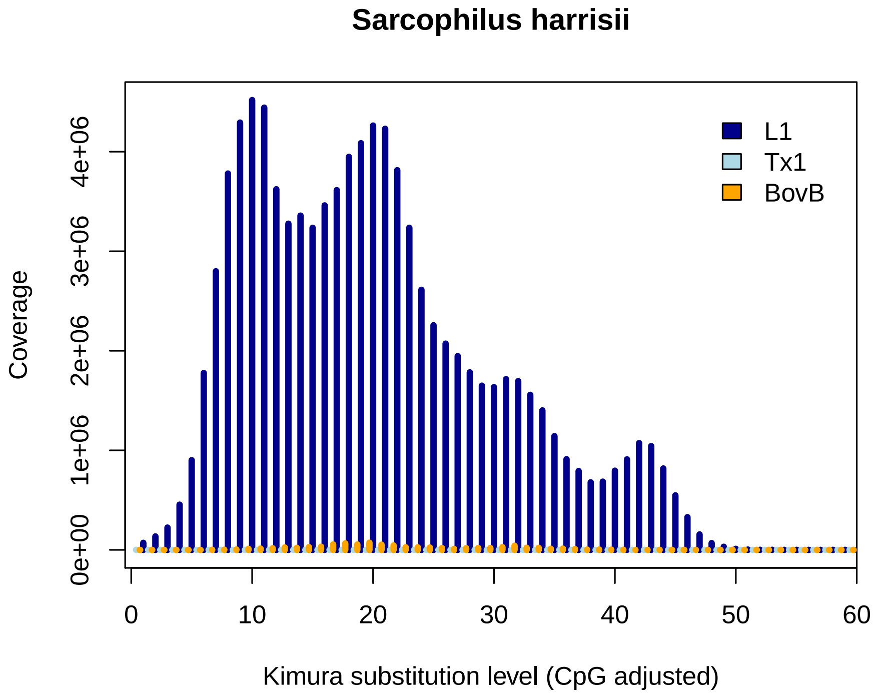

Figure S6

Afrotheria

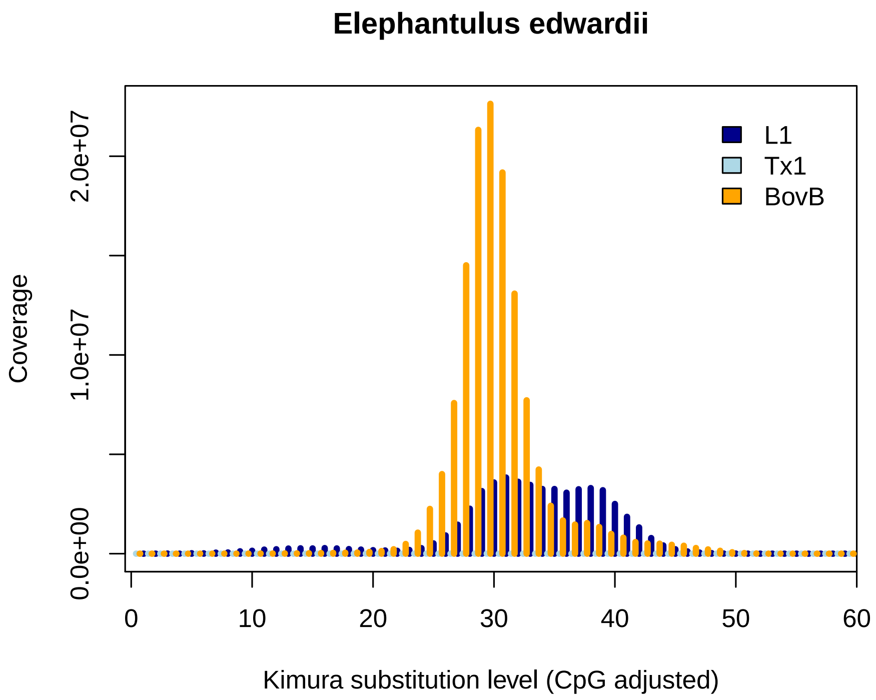

Figure S7

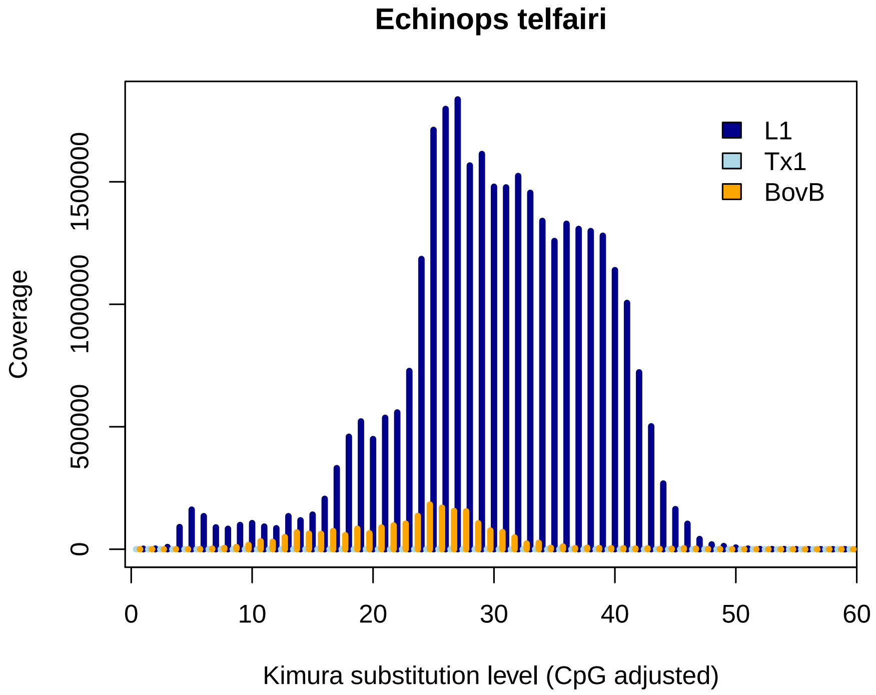

Figure S8

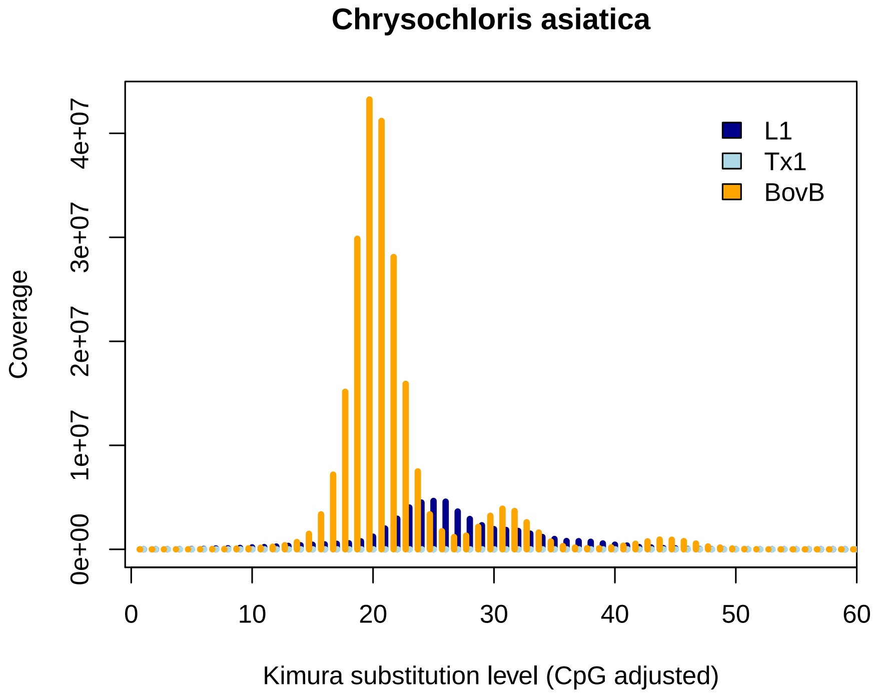

Figure S9

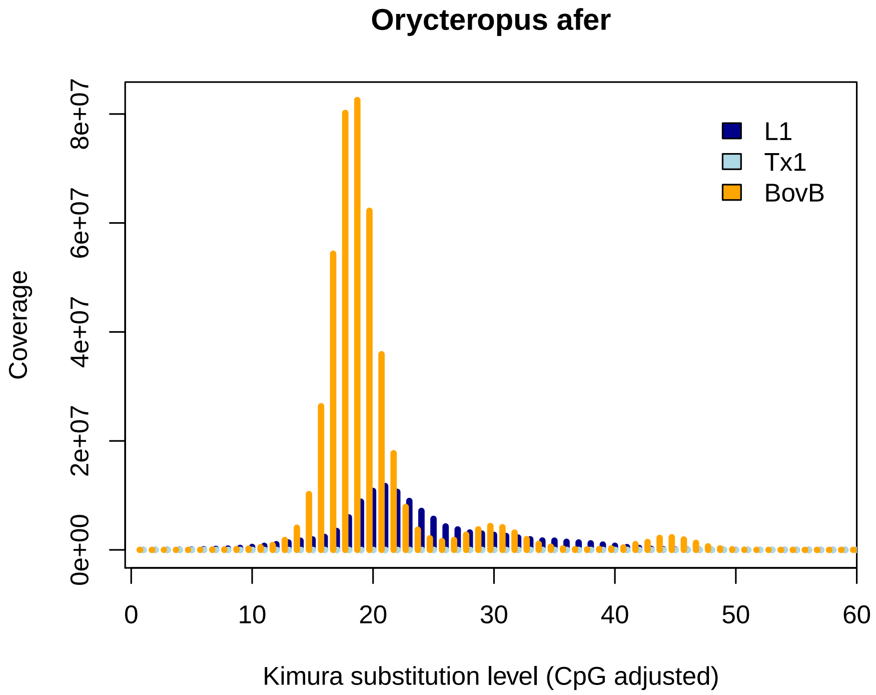

Figure S10

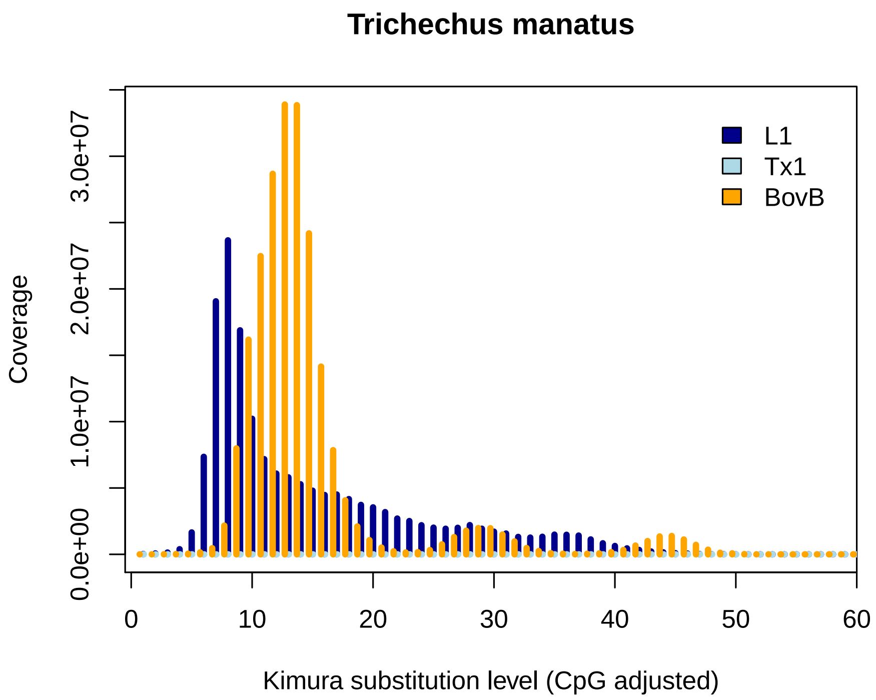

Figure S11

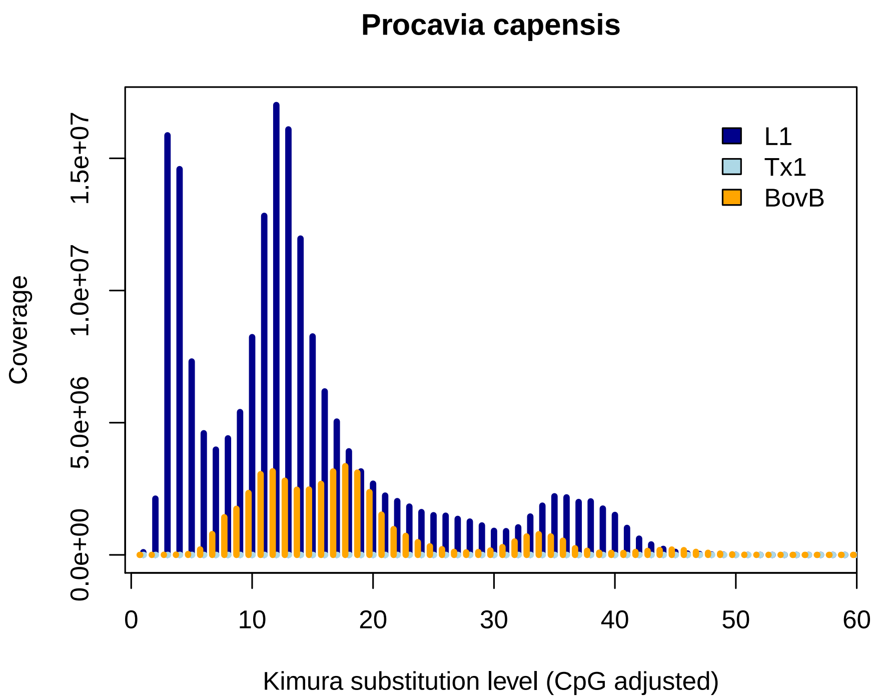

Figure S12

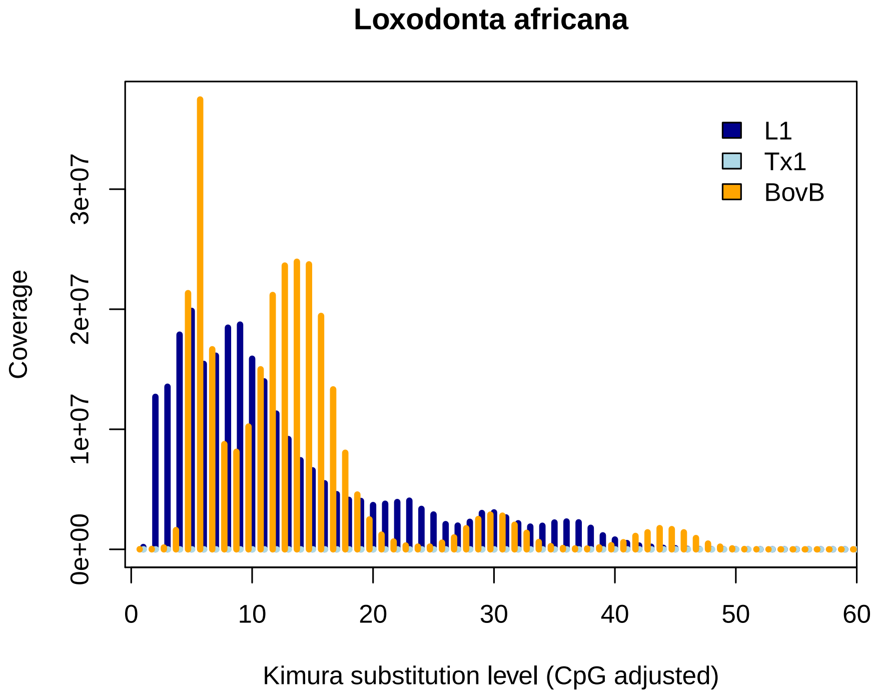

Figure S13

Chiroptera

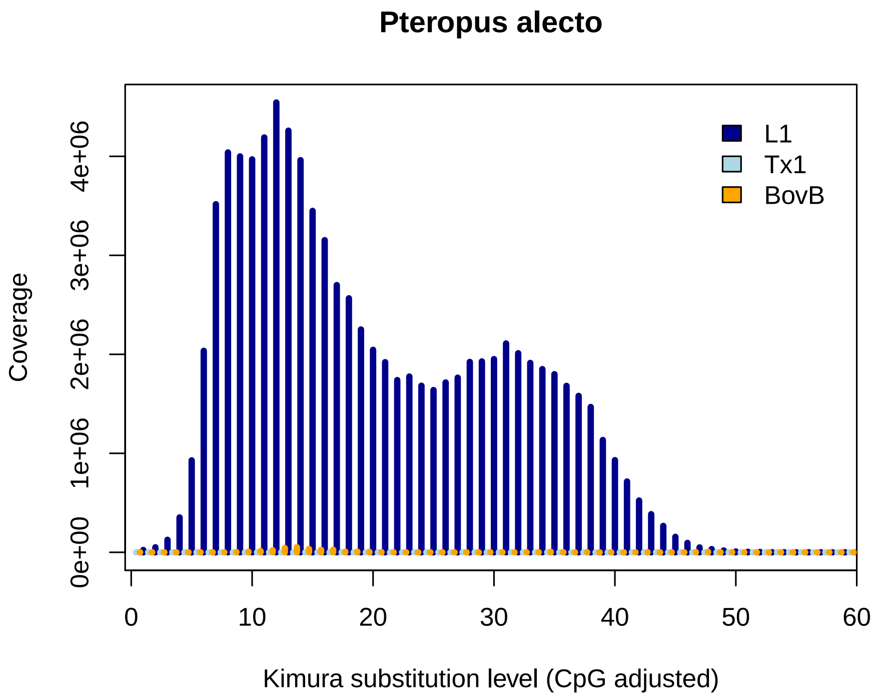

Figure S14

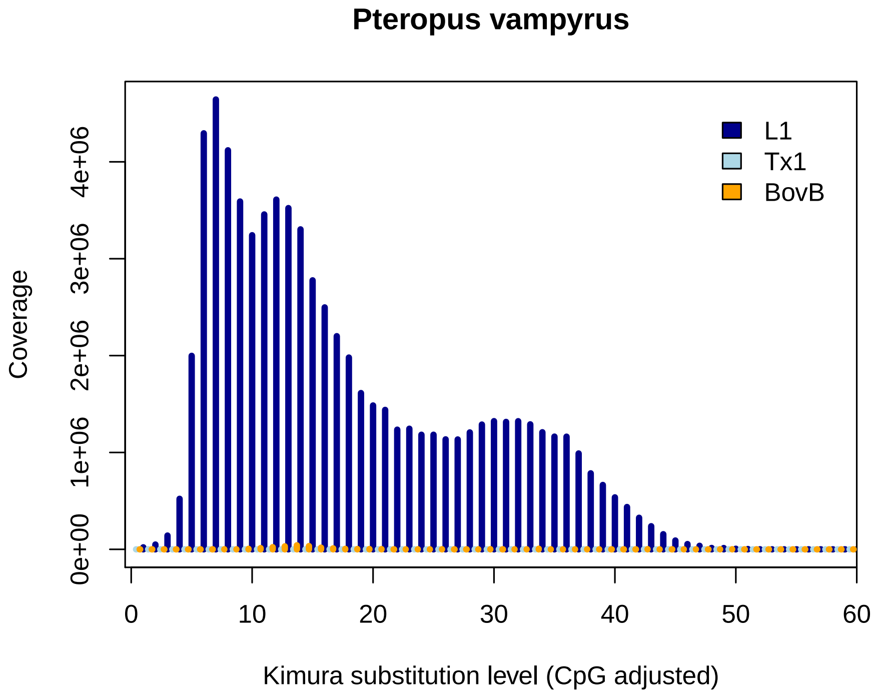

Figure S15

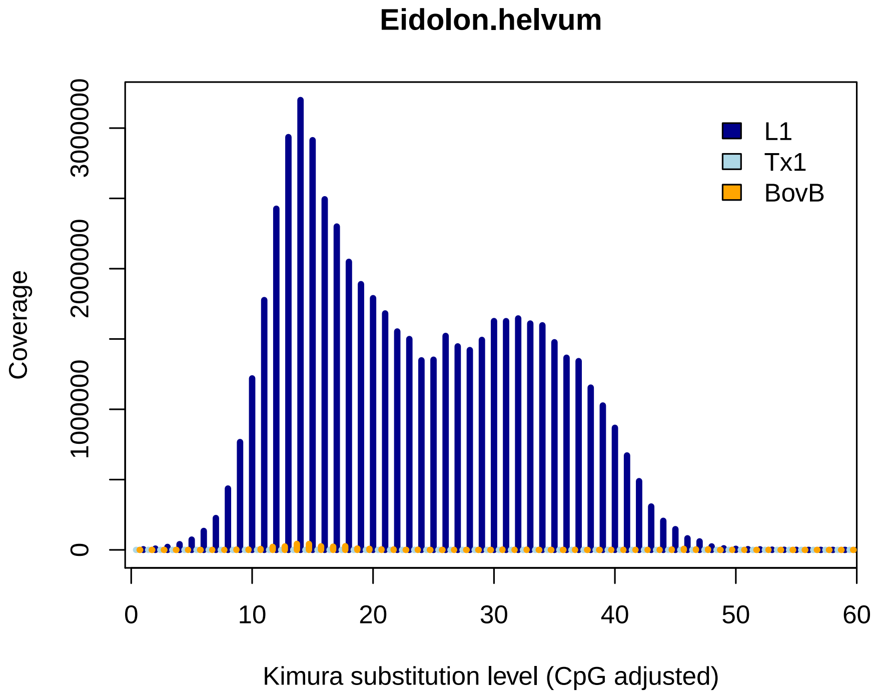

Figure S16

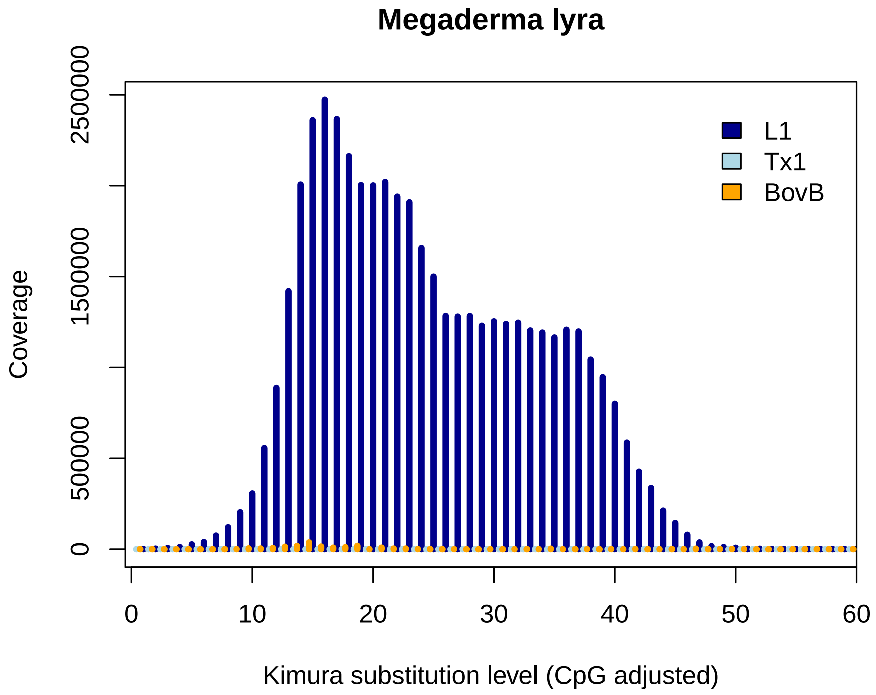

Figure S17

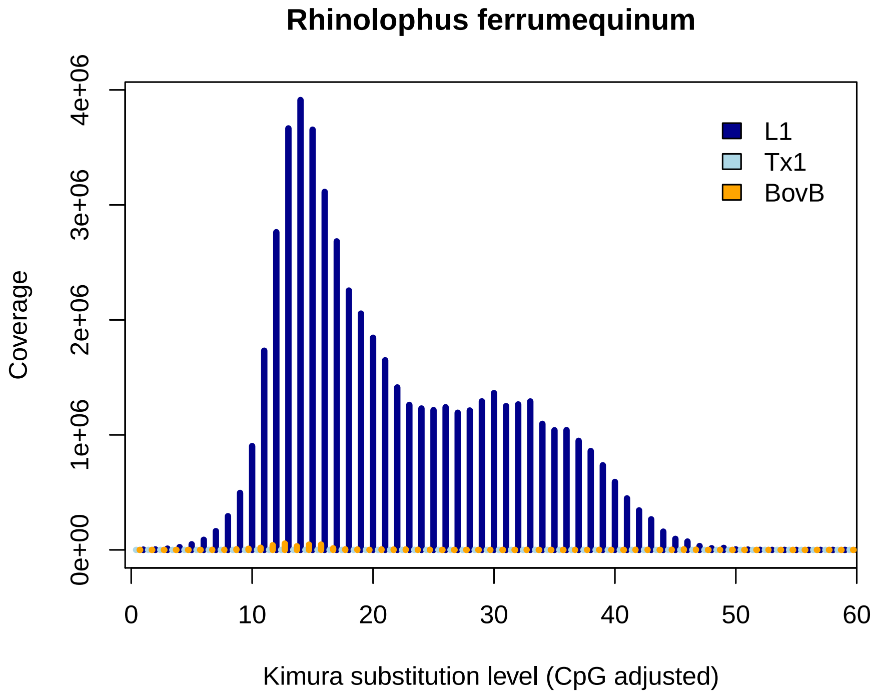

Figure S18

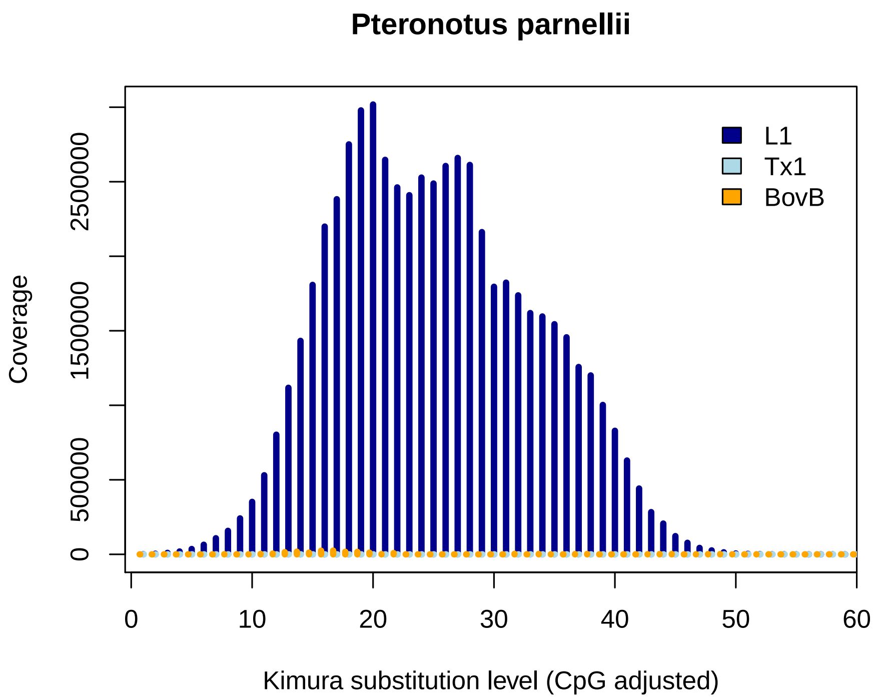

Figure S19

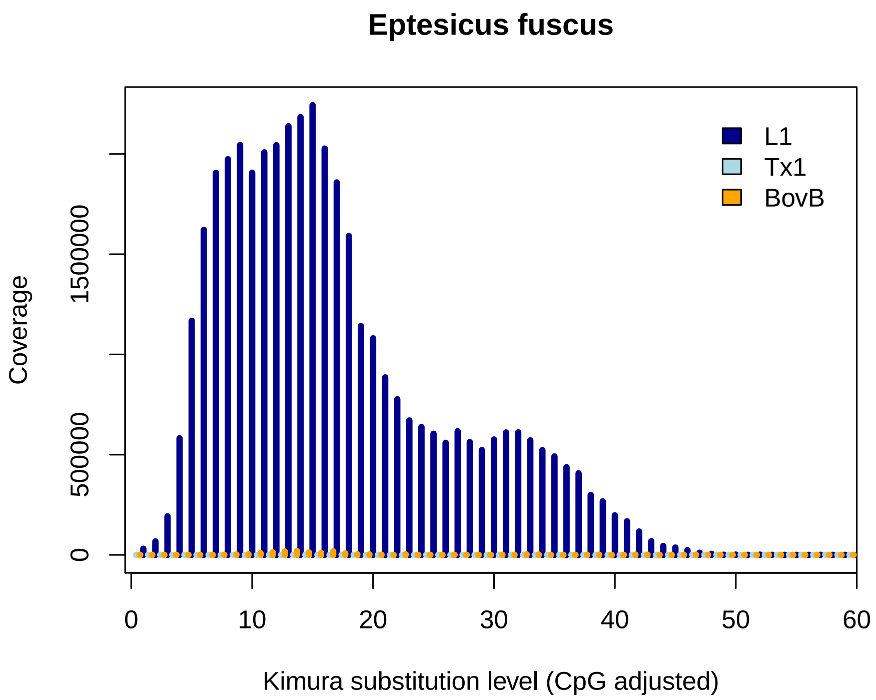

Figure S20

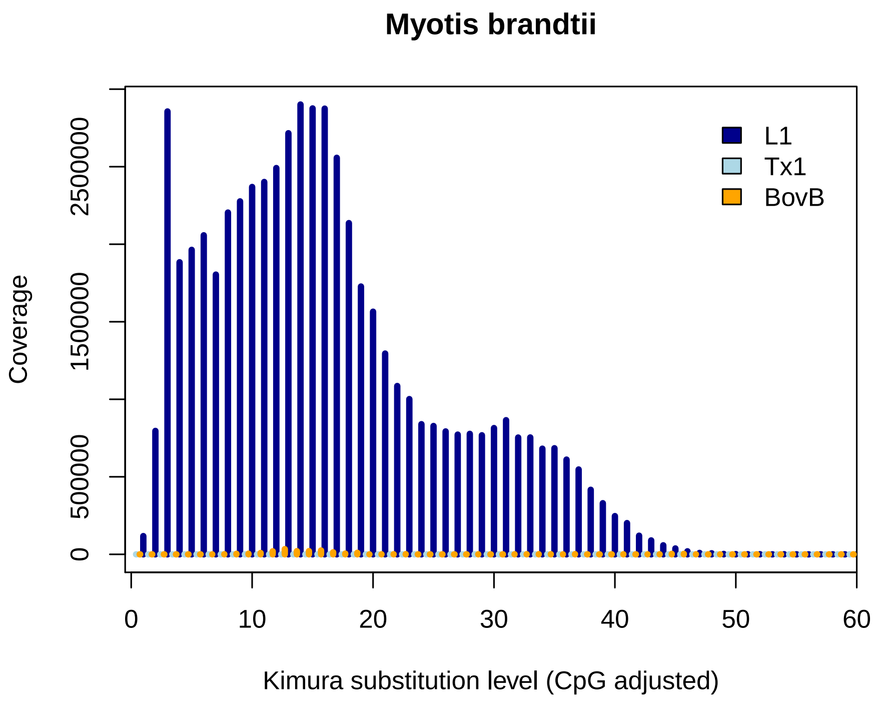

Figure S21

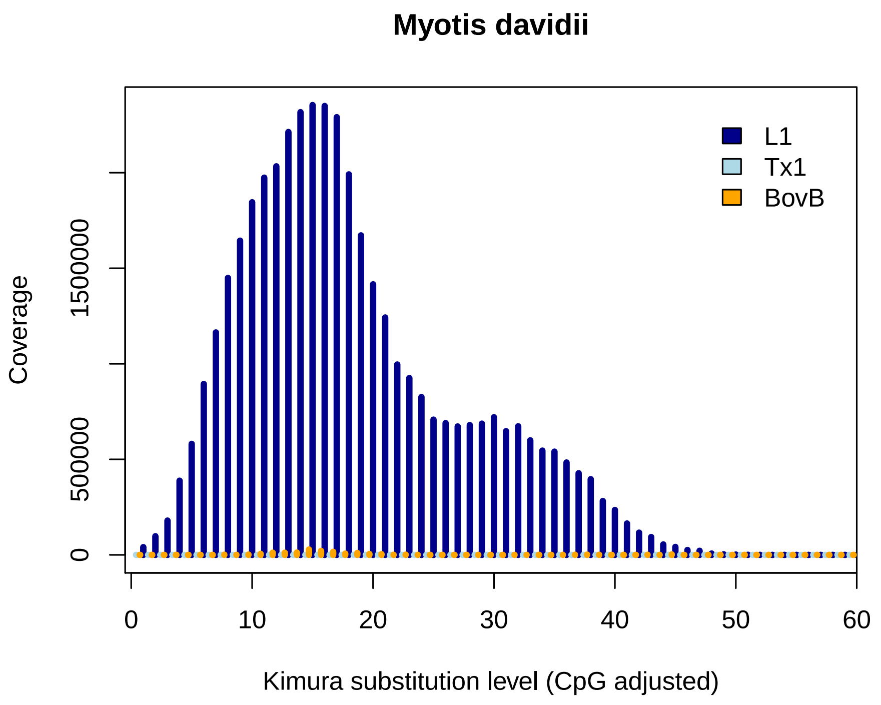

Figure S22

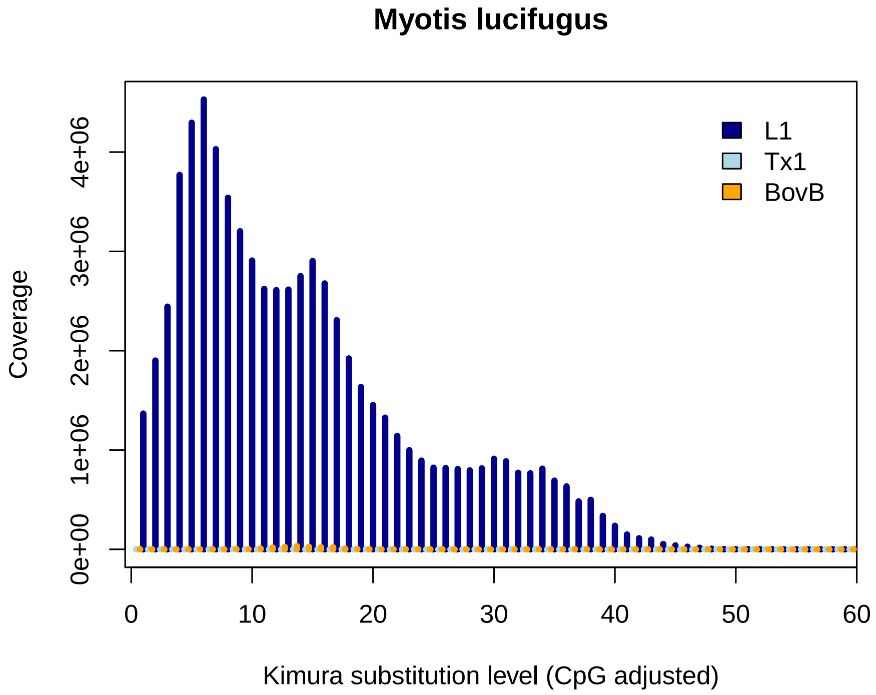

Figure S23

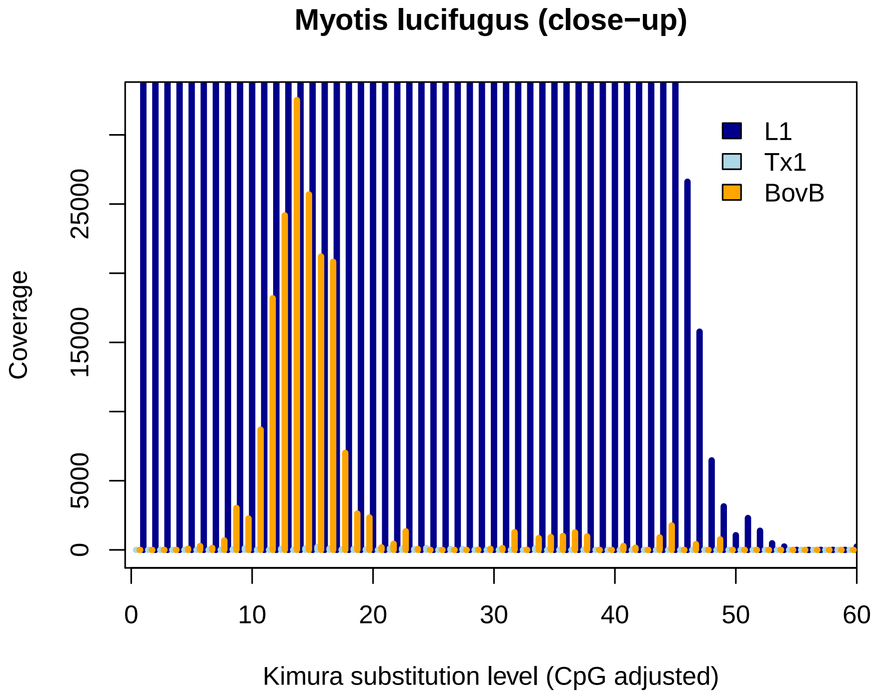

Figure S24

Perissodactyla

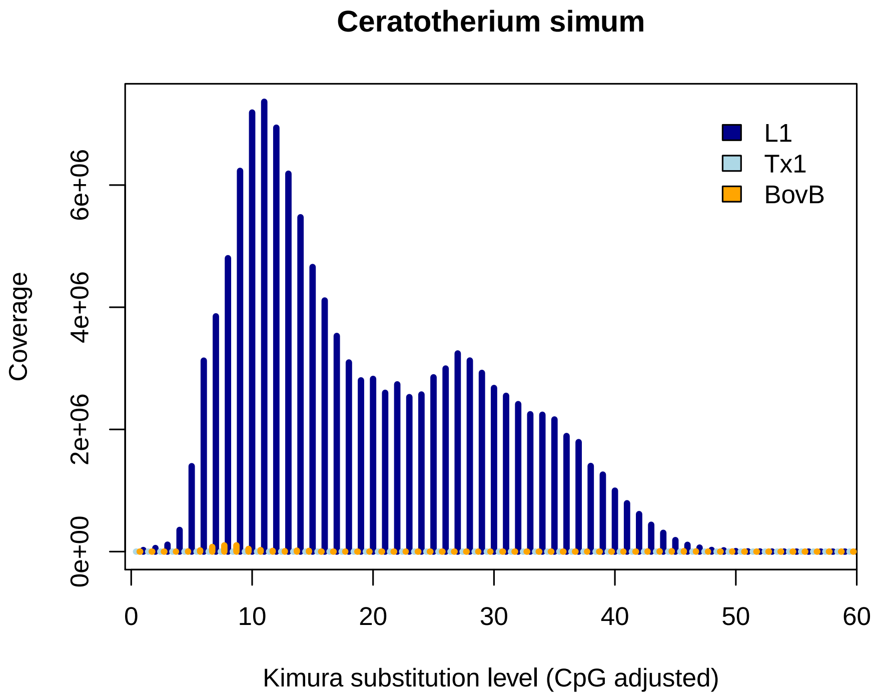

Figure S25

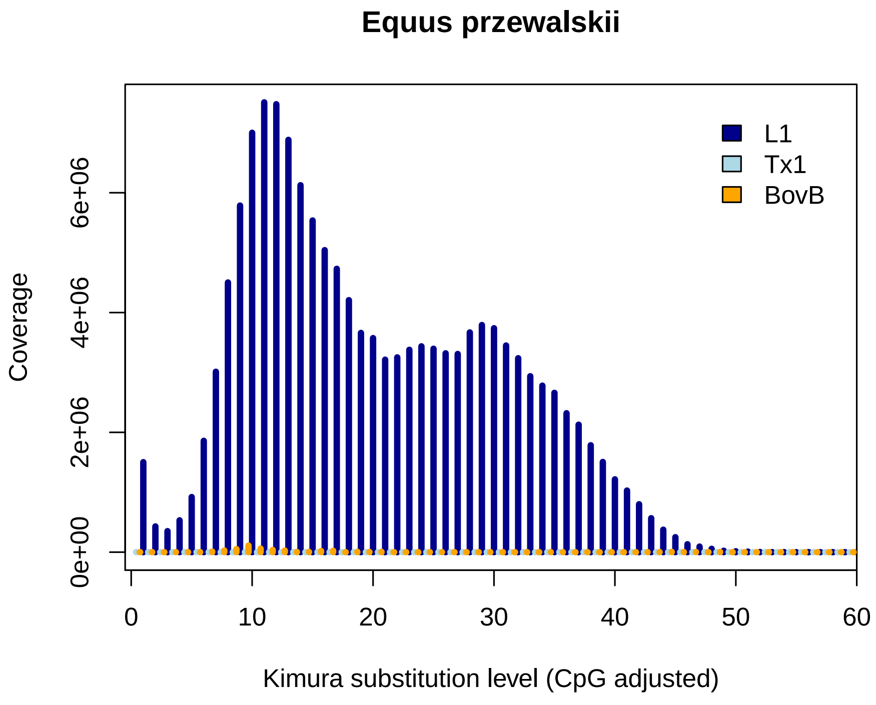

Figure S26

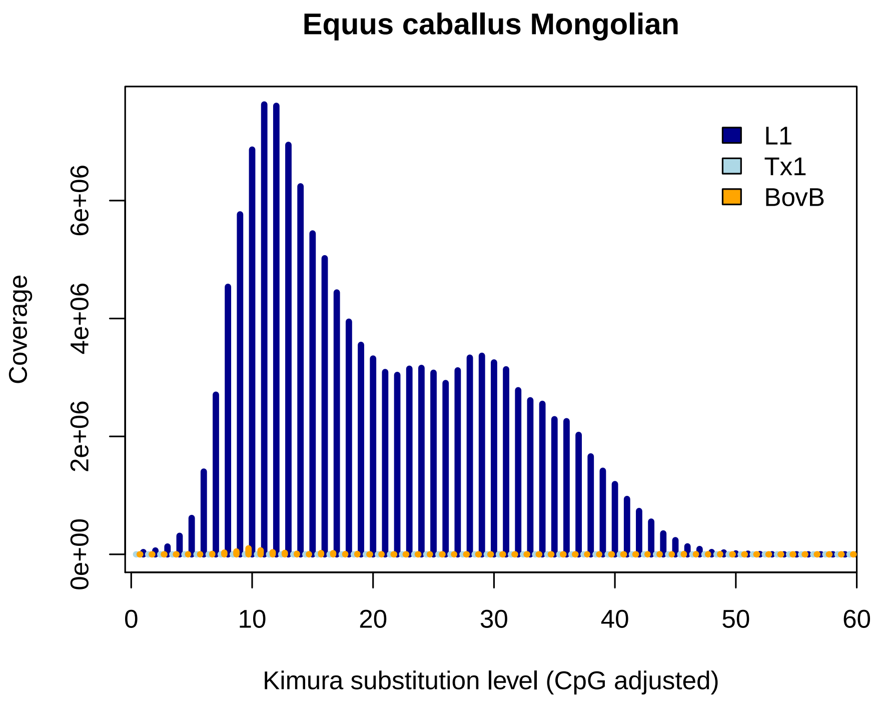

Figure S27

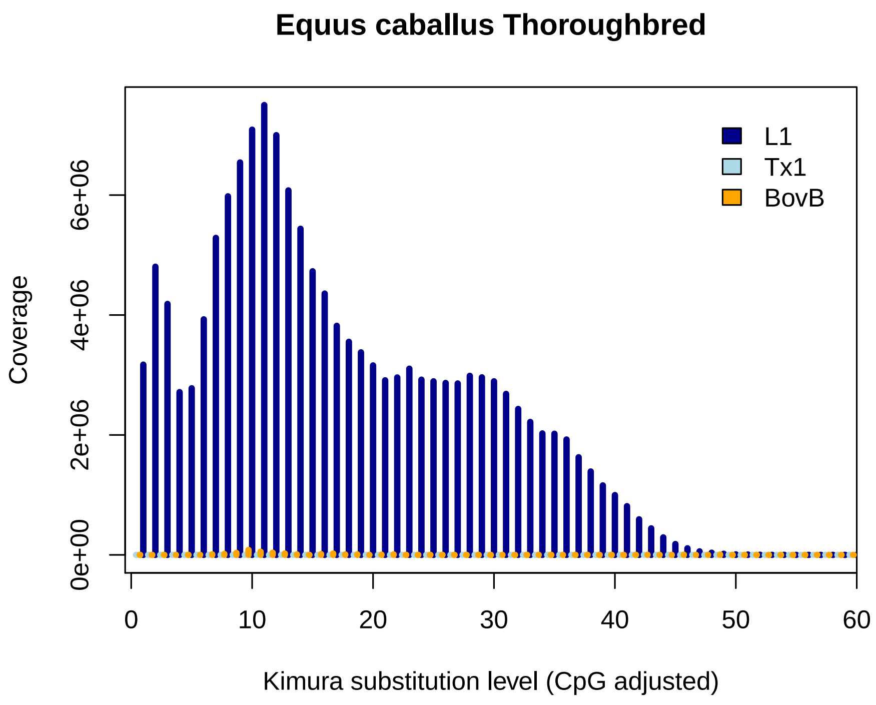

Figure S28

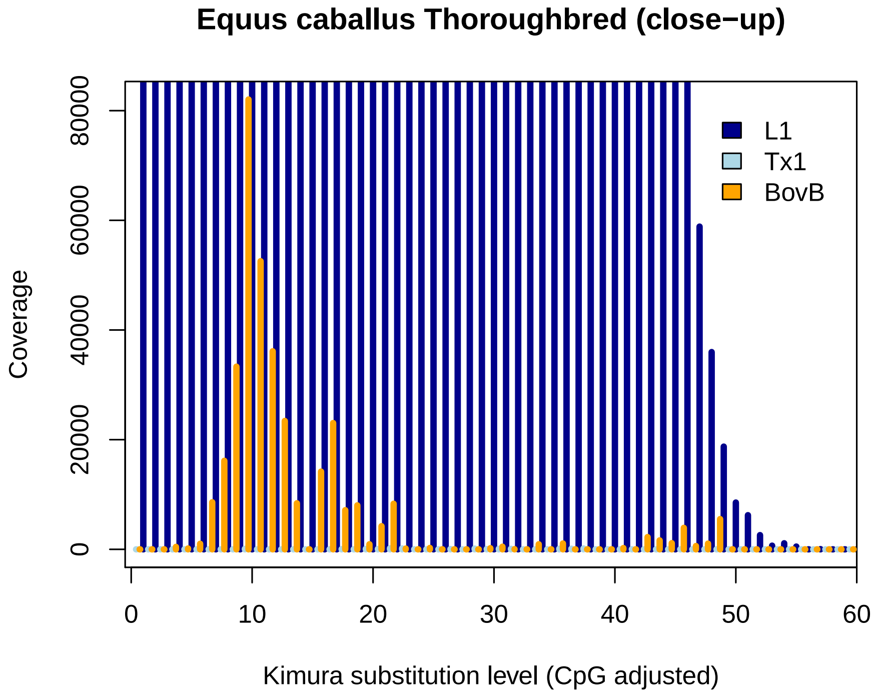

Figure S29

Bovidae

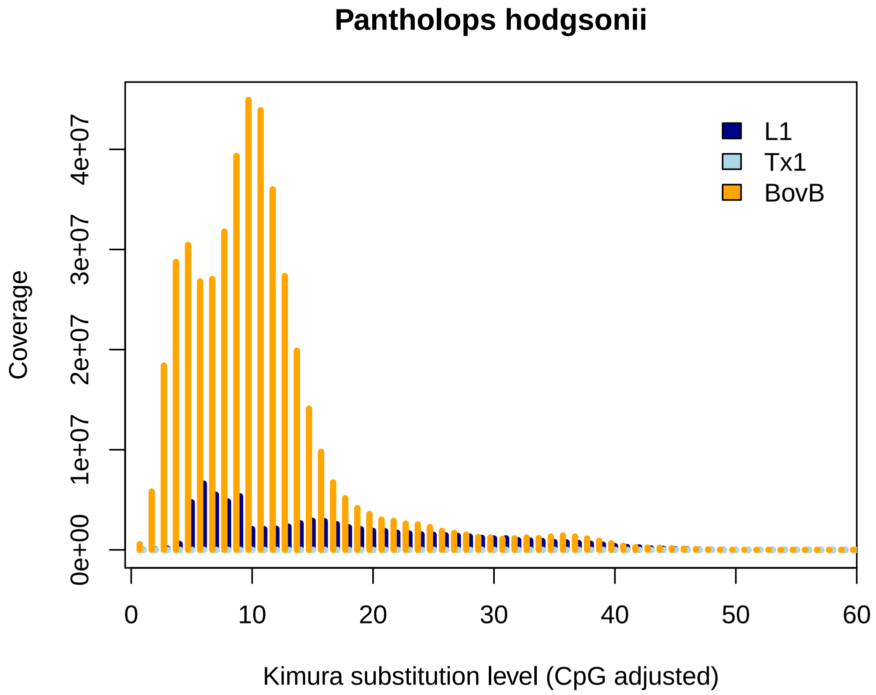

Figure S30

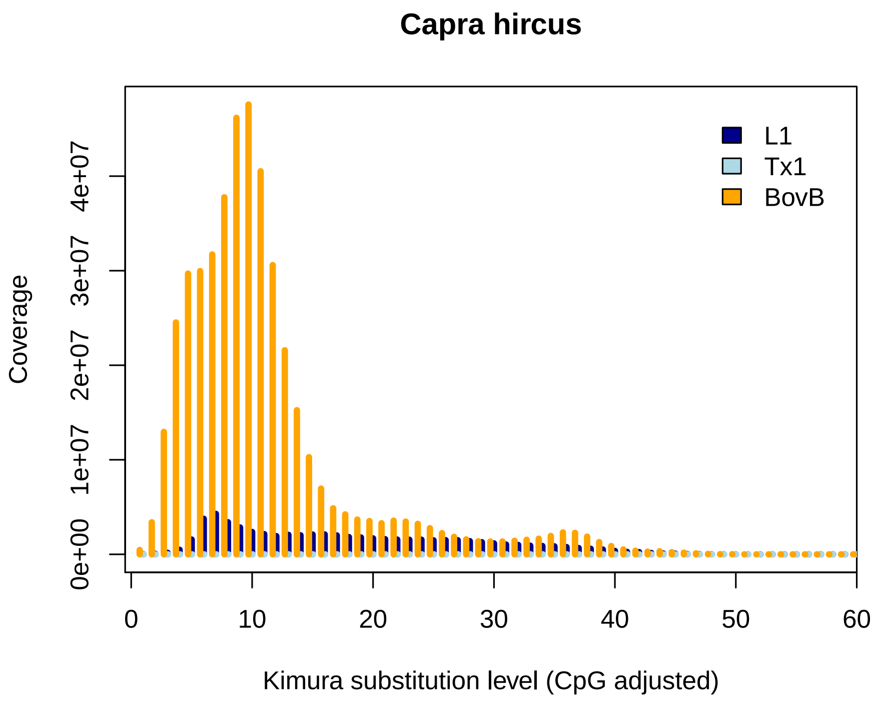

Figure S31

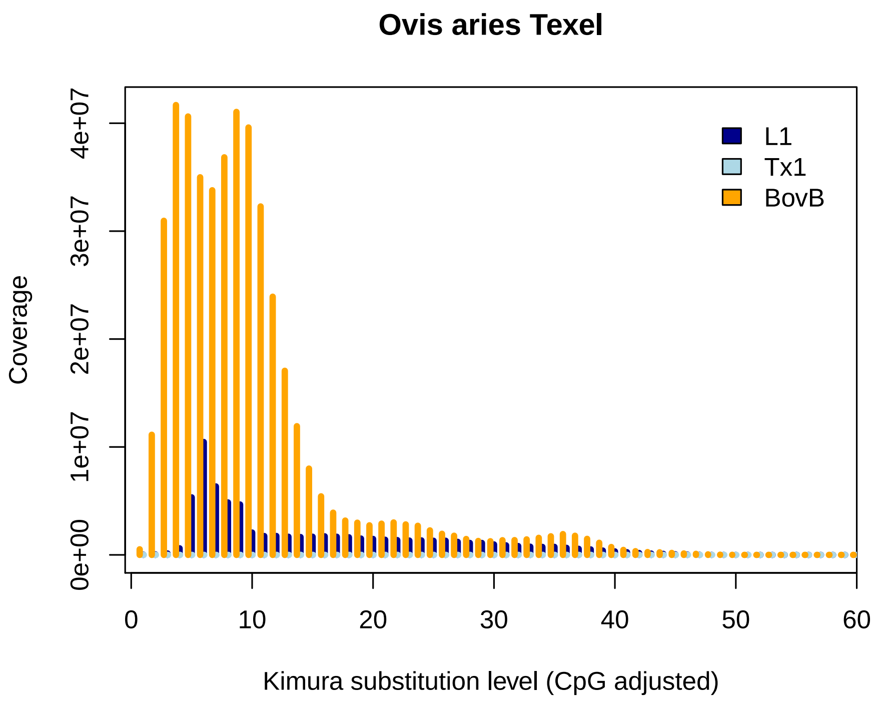

Figure S32

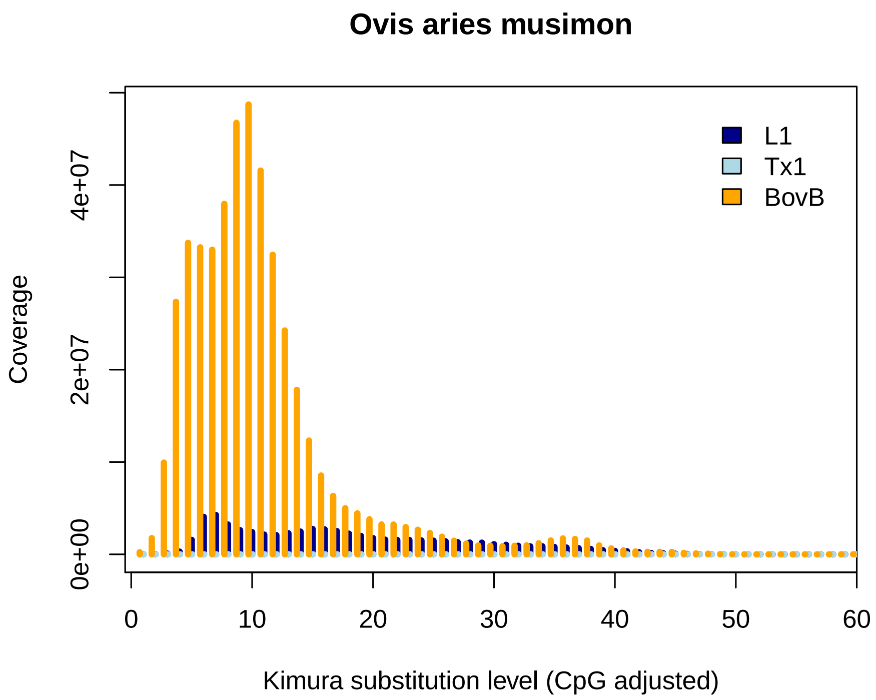

Figure S33

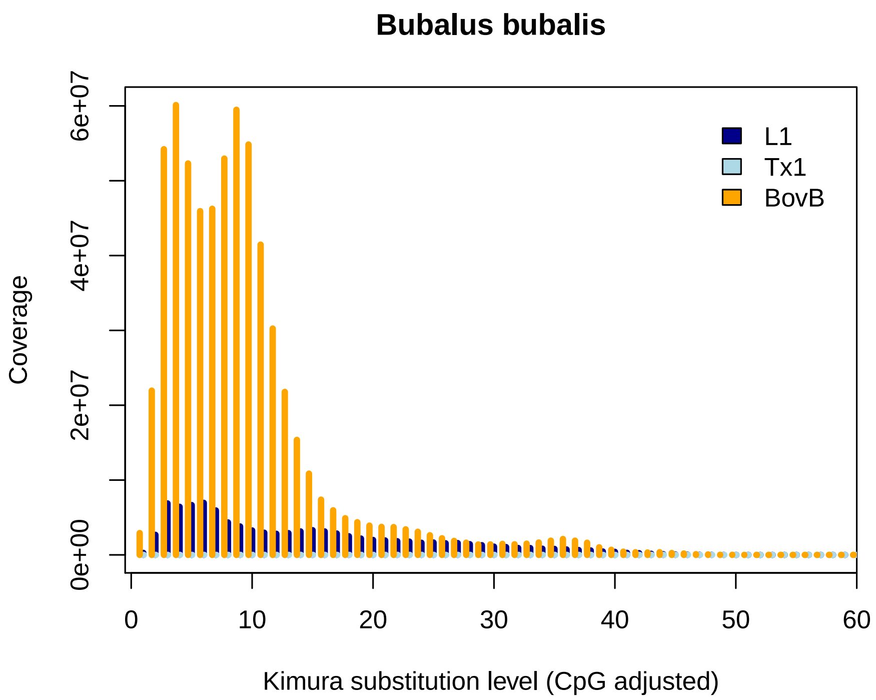

Figure S34

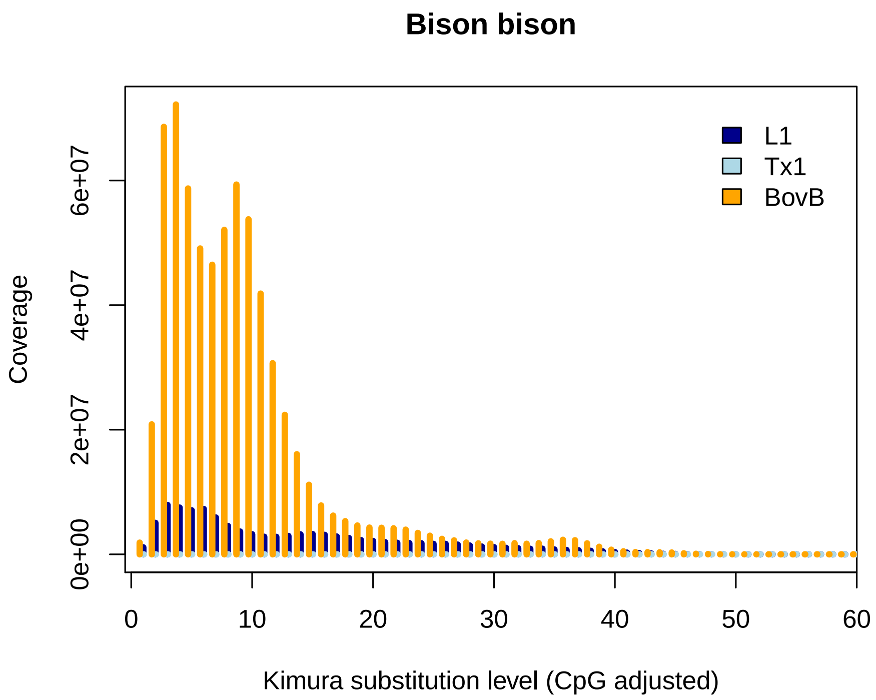

Figure S35

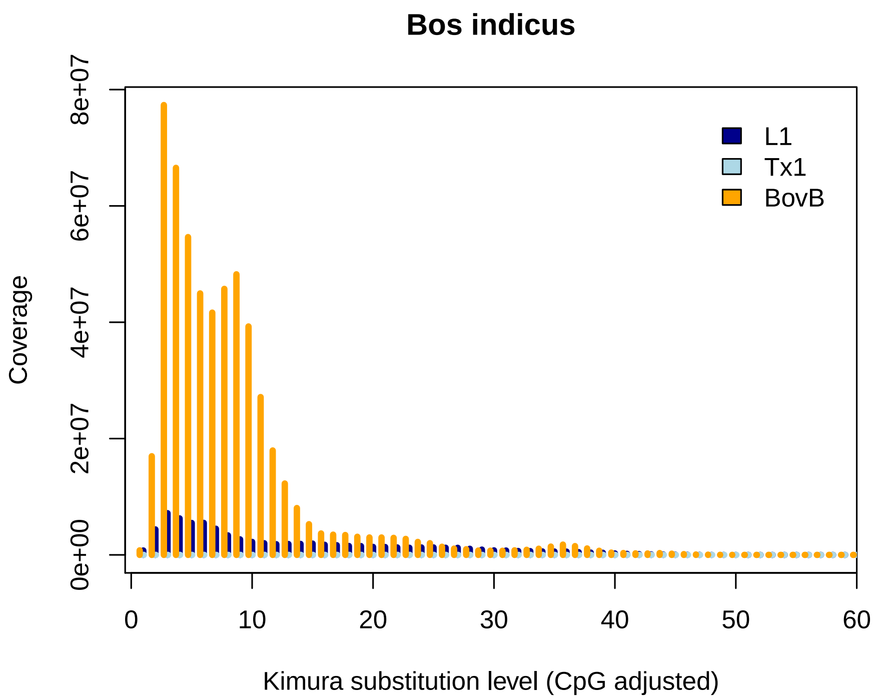

Figure S36

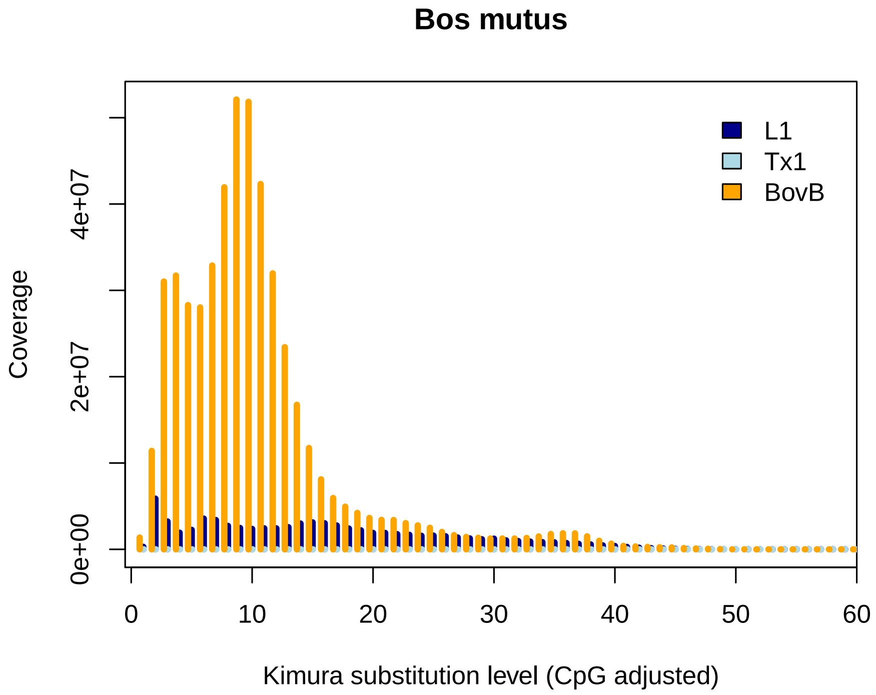

Figure S37

Squamata

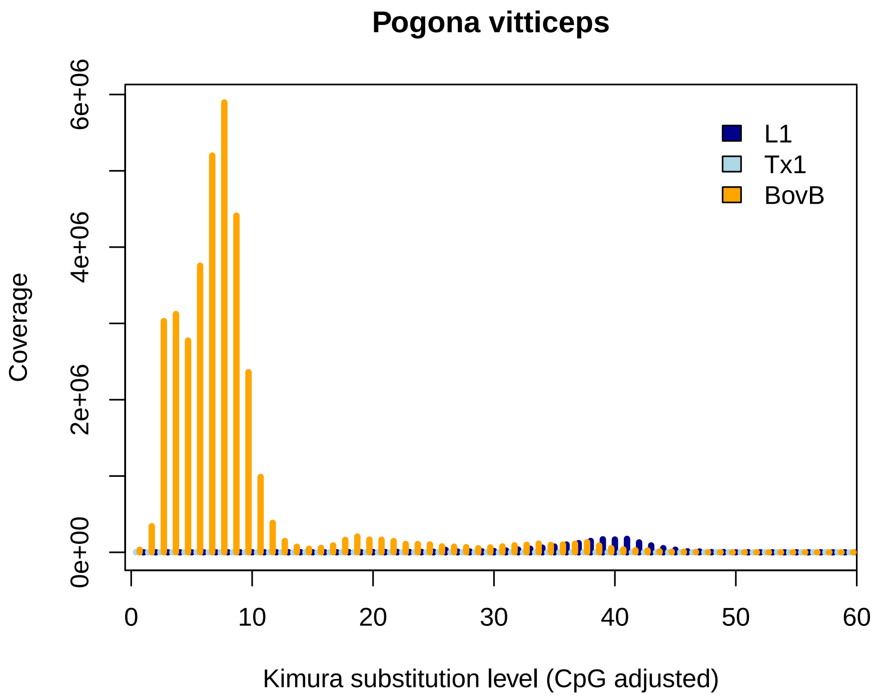

Figure S38

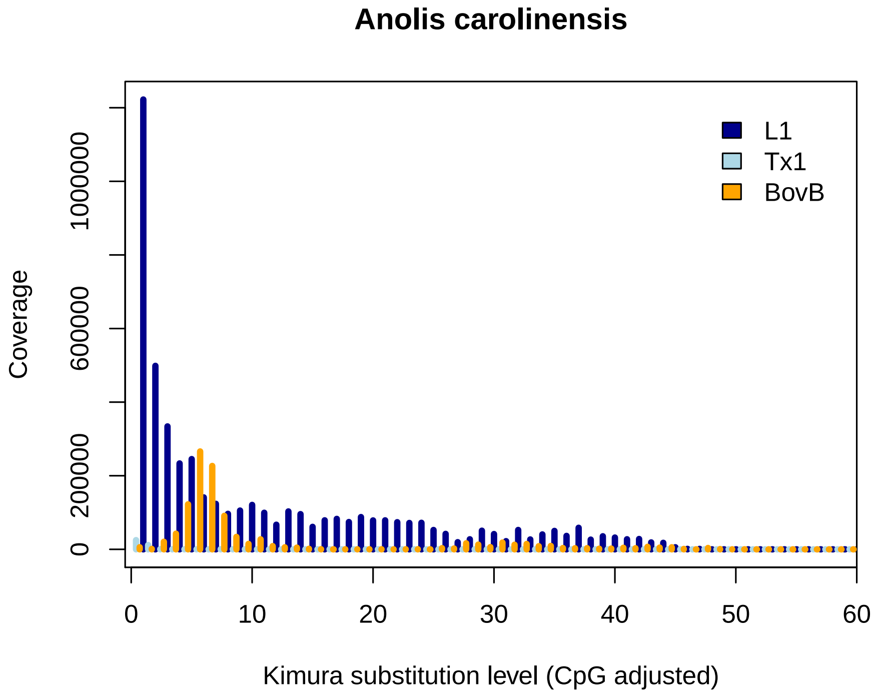

Figure S39

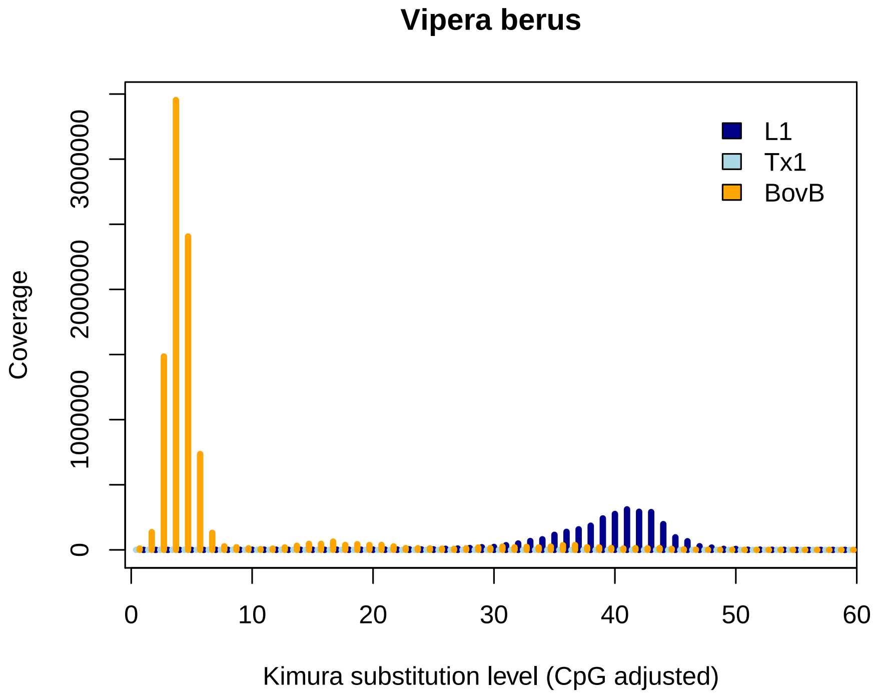

Figure S40

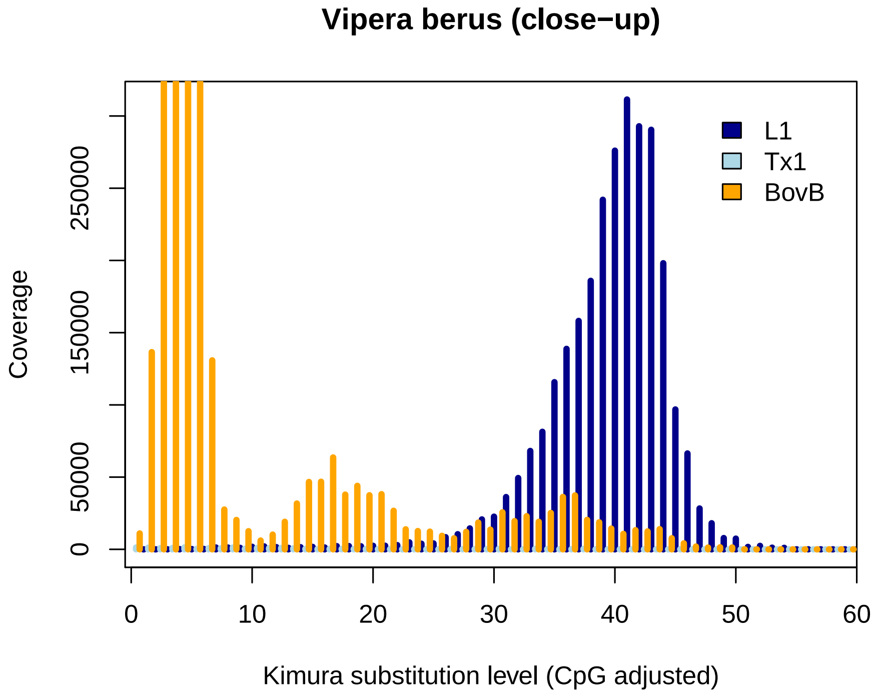

Figure S41

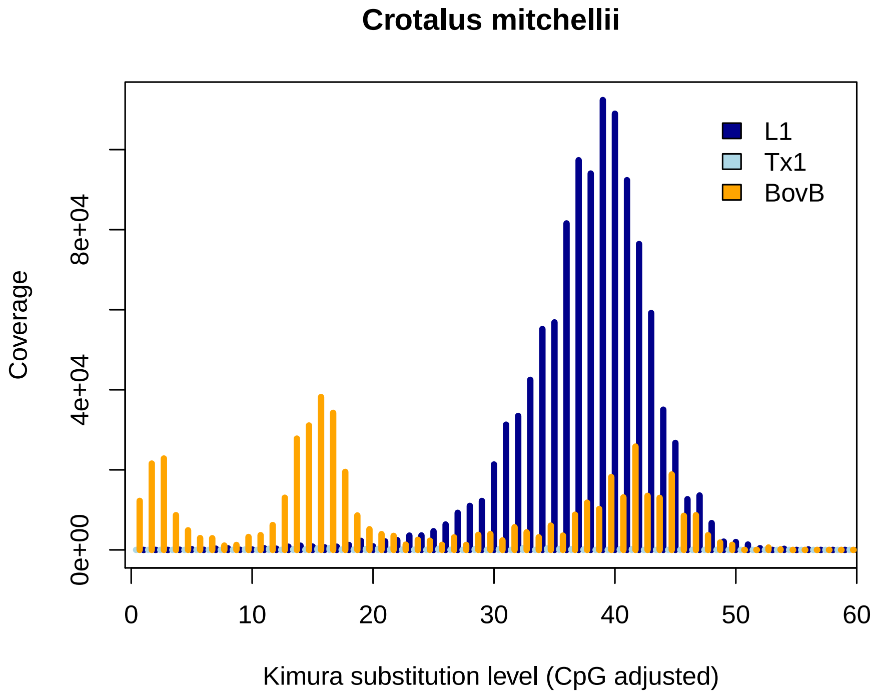

Figure S42

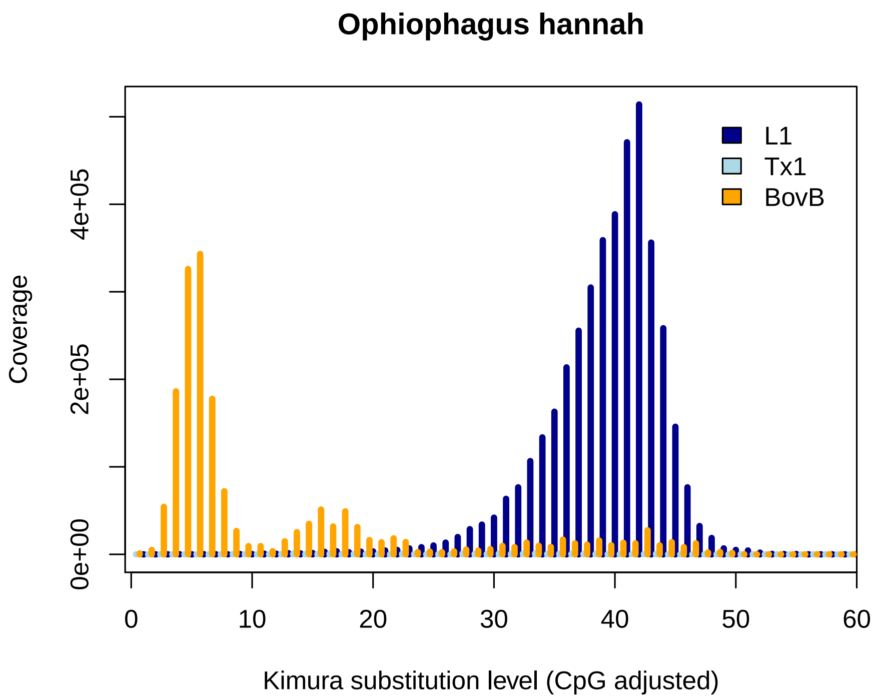

Figure S43

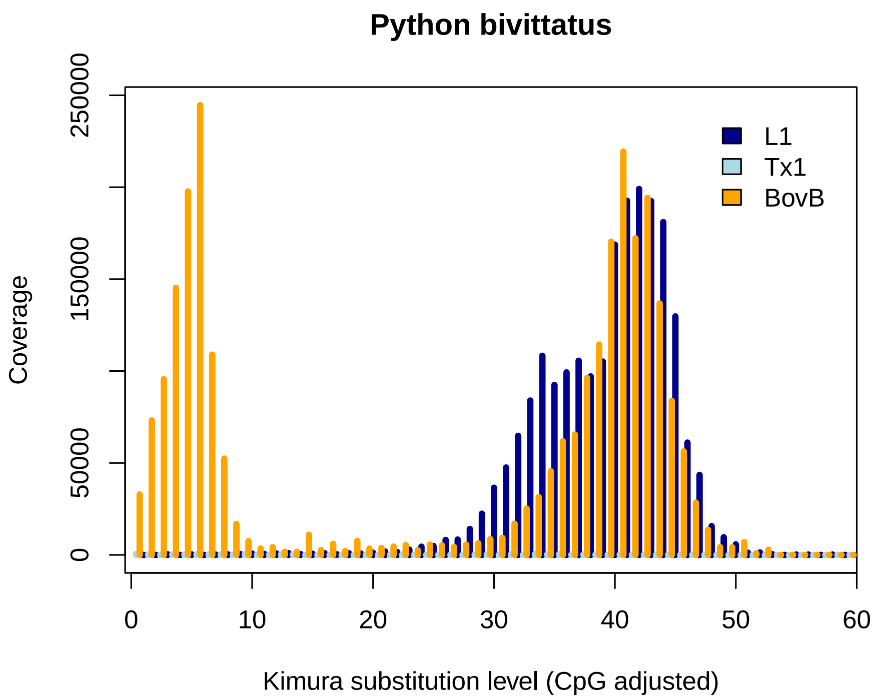

Figure S44

Amphibia

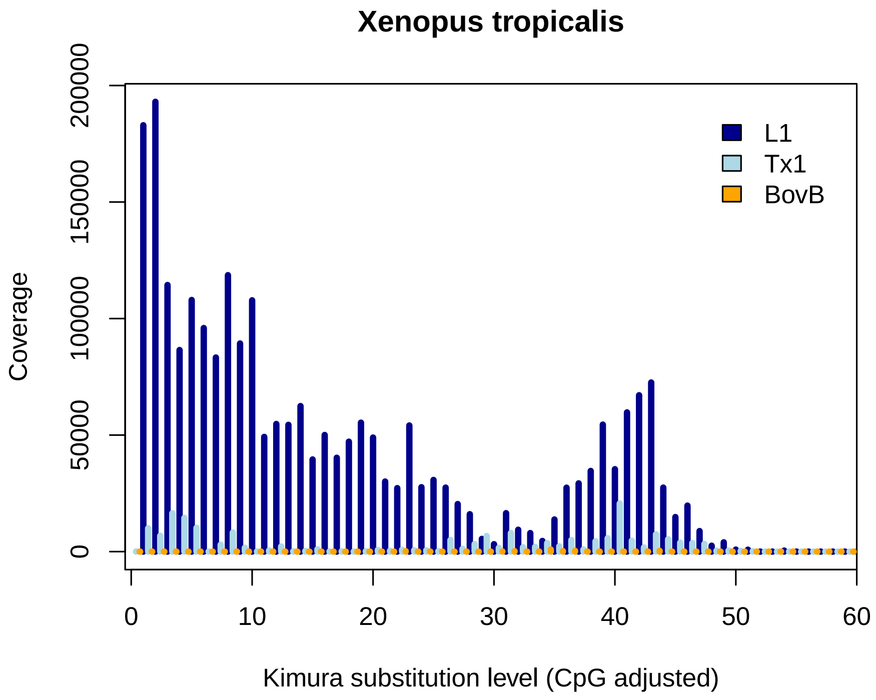

Figure S45

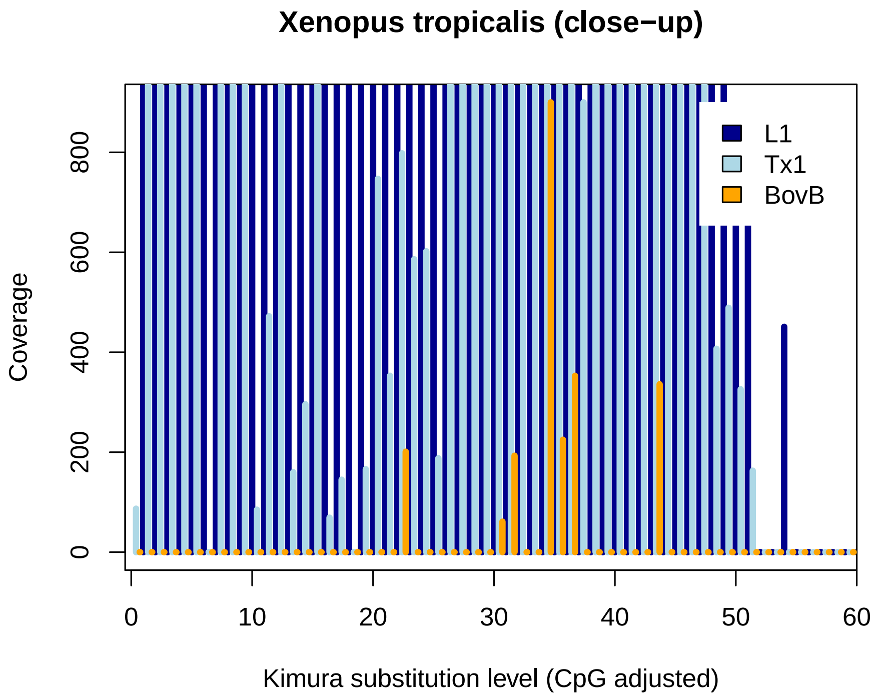

Figure S46

Neopterygii

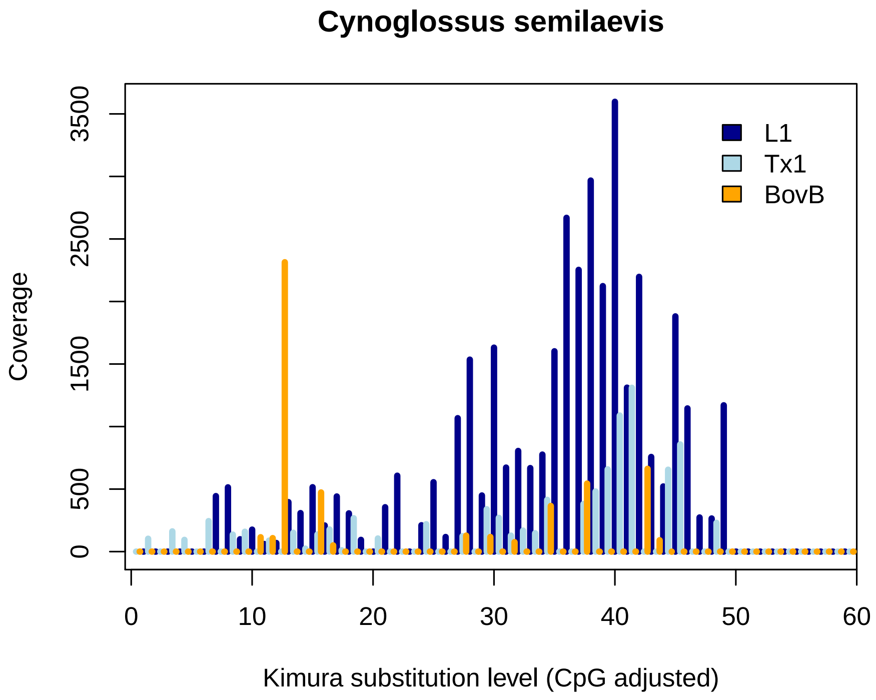

Figure S47

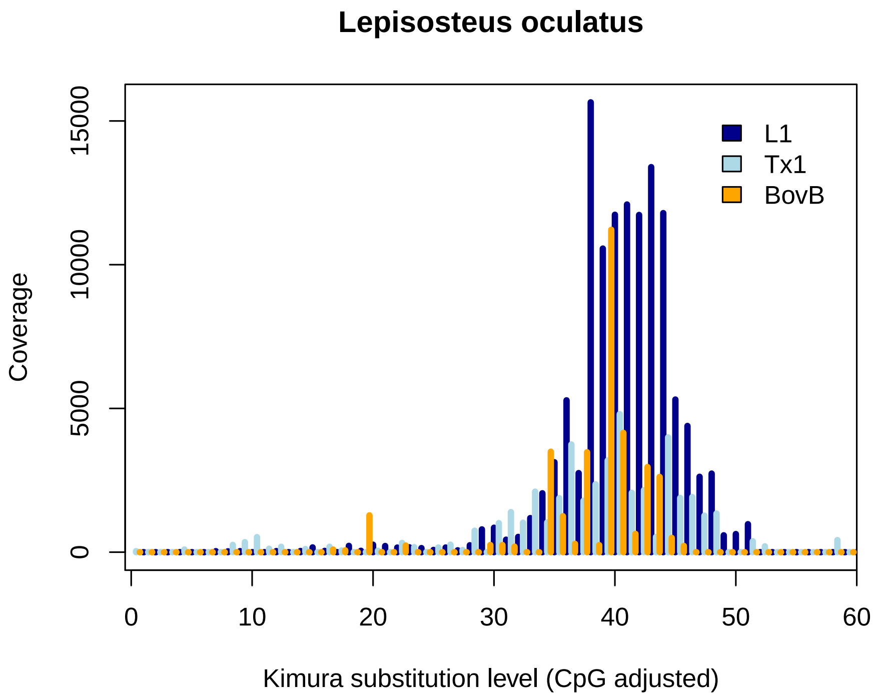

Figure S48

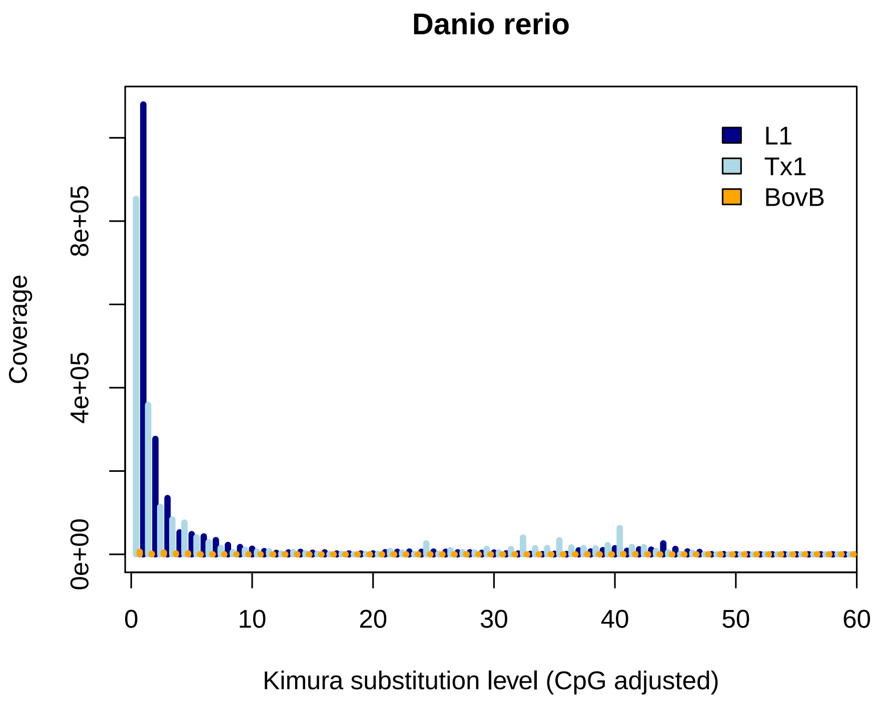

Figure S49

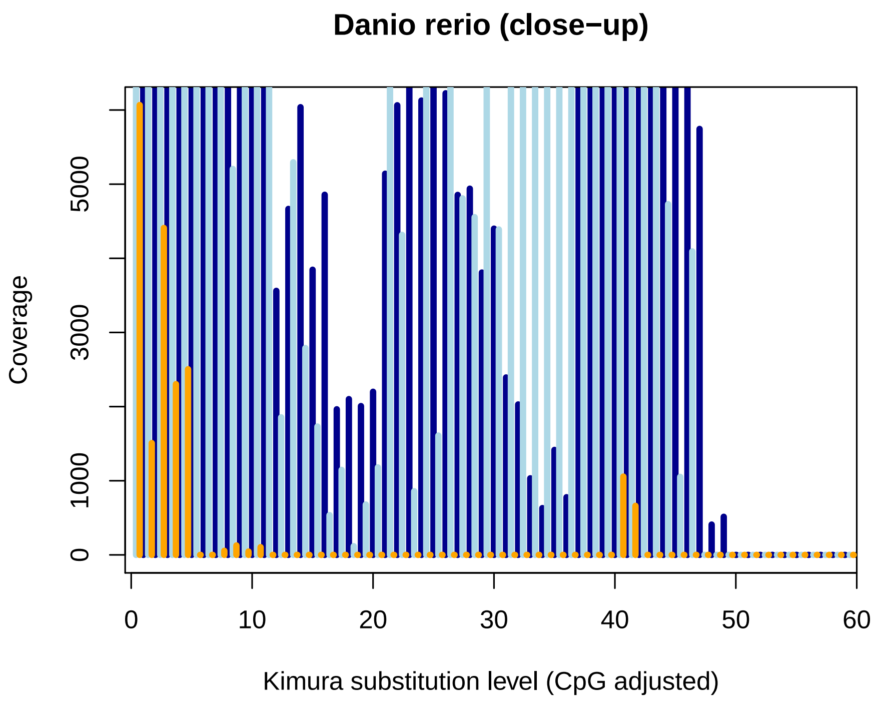

Figure S50

Other

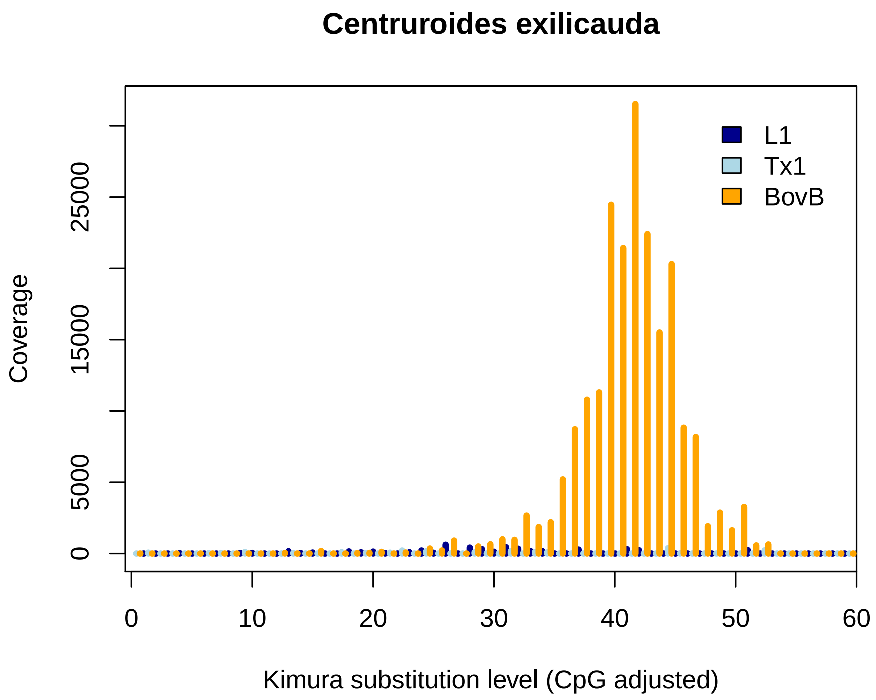

Figure S51

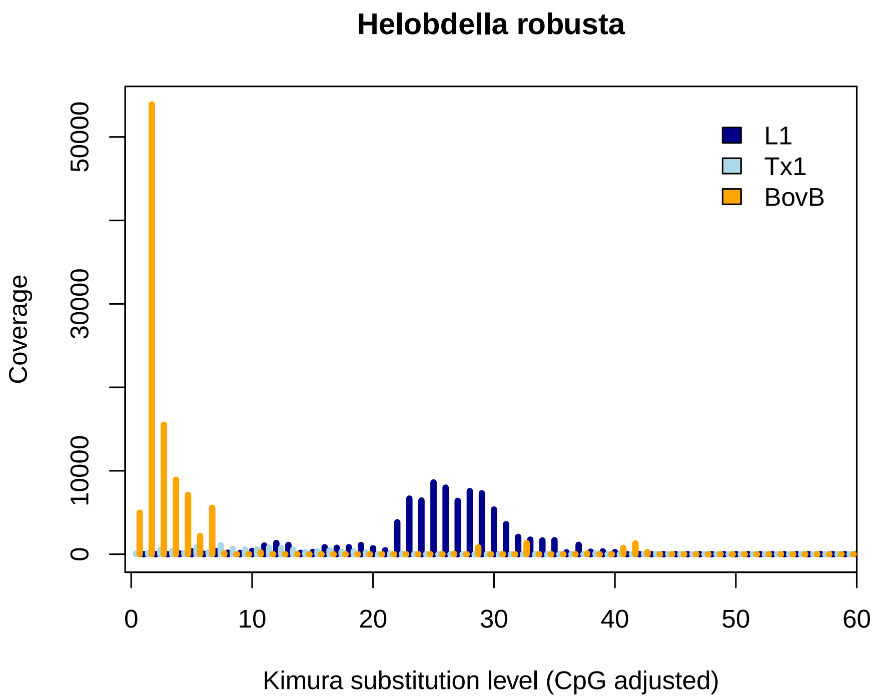

Figure S52

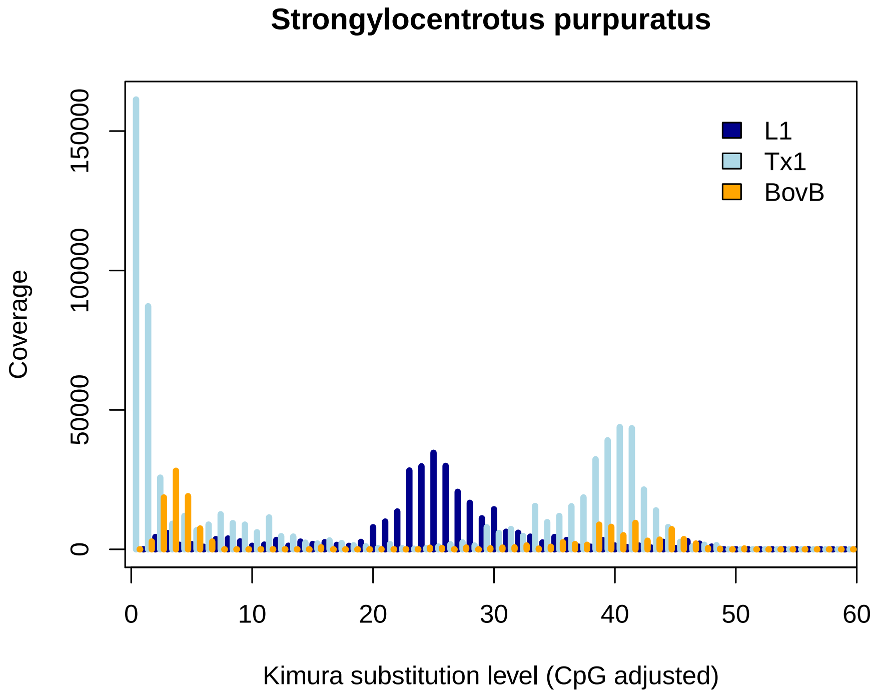

Figure S53

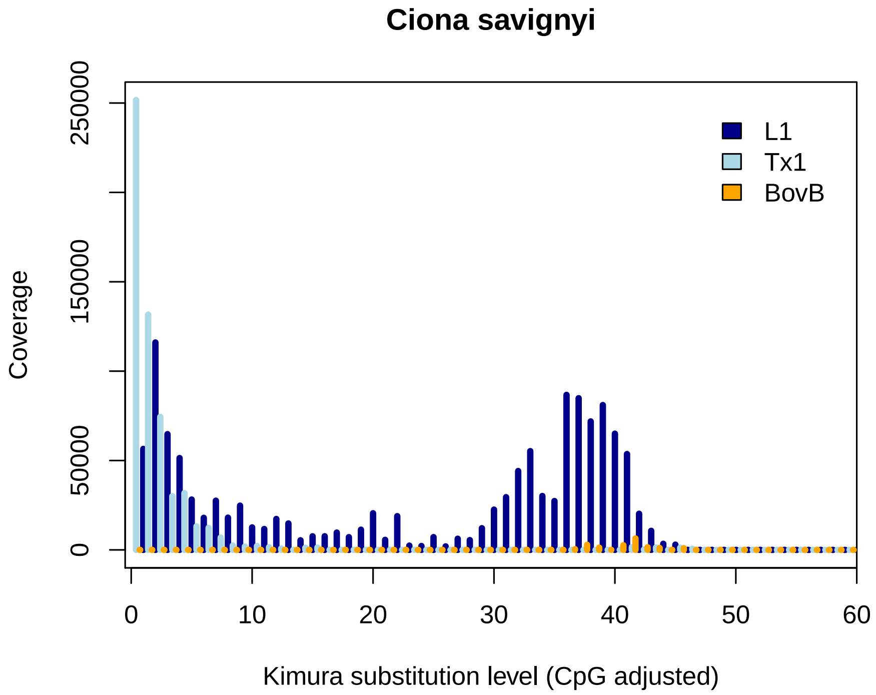

Figure S54

Figure S55: Chimeric L1-BovB

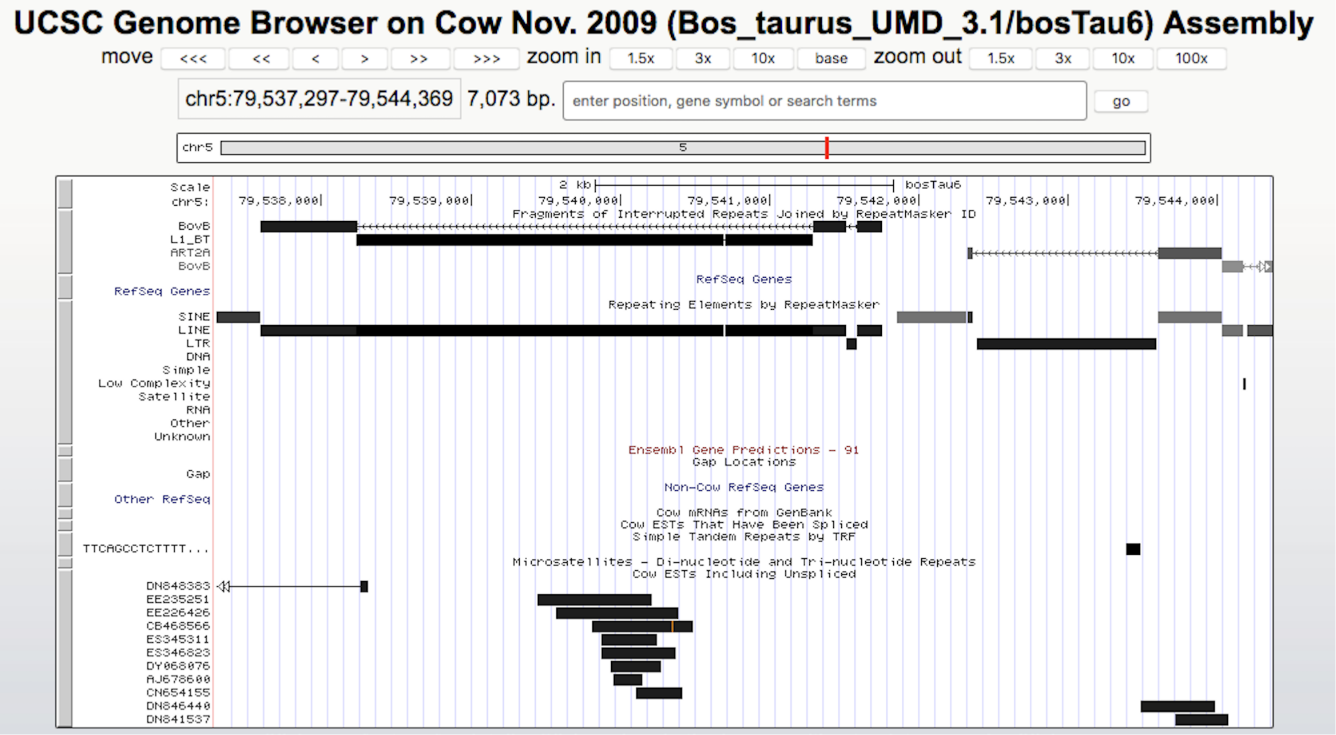

Figure S55: **Chimeric L1-BovB in cattle genomes.** Several cow ESTs overlap the L1 reverse transcriptase domain, but these may be artifacts/mismapped. No strong evidence to suggest transcription.
